# A worldwide model for boundaries of urban settlements

**DOI:** 10.1098/rsos.180468

**Published:** 2018-05-23

**Authors:** Erneson A. Oliveira, Vasco Furtado, José S. Andrade, Hernán A. Makse

**Affiliations:** 1 Programa de Pós Graduação em Informática Aplicada, Universidade de Fortaleza, 60811-905 Fortaleza, Ceará, Brazil; 2 Mestrado Profissional em Ciências da Cidade, Universidade de Fortaleza, 60811-905 Fortaleza, Ceará, Brazil; 3 Departamento de Física, Universidade Federal do Ceará, Campus do Pici, 60451-970 Fortaleza, Ceará, Brazil; 4 Levich Institute and Physics Department, City College of New York, New York, NY 10031, USA

**Keywords:** science of cities, urban settlements, city clustering algorithm, Zipf’s law

## Abstract

The shape of urban settlements plays a fundamental role in their sustainable planning. Properly defining the boundaries of cities is challenging and remains an open problem in the science of cities. Here, we propose a worldwide model to define urban settlements beyond their administrative boundaries through a bottom-up approach that takes into account geographical biases intrinsically associated with most societies around the world, and reflected in their different regional growing dynamics. The generality of the model allows one to study the scaling laws of cities at all geographical levels: countries, continents and the entire world. Our definition of cities is robust and holds to one of the most famous results in social sciences: Zipf’s law. According to our results, the largest cities in the world are not in line with what was recently reported by the United Nations. For example, we find that the largest city in the world is an agglomeration of several small settlements close to each other, connecting three large settlements: Alexandria, Cairo and Luxor. Our definition of cities opens the doors to the study of the economy of cities in a systematic way independently of arbitrary definitions that employ administrative boundaries.

## 1. Introduction

What are cities? In *The Death and Life of the Great American Cities*, Jacobs argues that human relations can be seen as a proxy for places within cities [1]. A modern view of cities establishes that they can be defined by the interactions among several types of networks [2,3], from infrastructure networks to social networks. In recent years, an increasing number of studies have been proposed to define cities through consistent mathematical models [4–15] and to investigate urban indicators at inter- and intra-city scales, in order to shed some light on problems faced by decision-makers [16–31]. Despite the efforts of such studies, properly defining the boundaries of urban settlements remains an open problem in the science of cities. A minimum criterion of acceptability for any model of cities seems to be the one that retrieves a conspicuous scaling law found for USA, UK and other countries, known as Zipf’s law [6,7,32–42]. In 1949, Zipf [43] observed that the frequency of words used in the English language obeys a natural and robust power law behaviour, i.e. a few words are used many times, while many words are used just a few times. Zipf’s law can be represented generically by the following relationship between the size *S* of objects from a given set and its rank *R*:

1.1 $$R\propto S^{-\zeta },$$

where *ζ*=1 is Zipf’s exponent. The size of objects is, in the original context, the frequency of used words. On the other hand, if such objects are cities, then the sizes stand for the population of each city, taking into account Zipf’s law and reflecting the fact that there are more small towns than metropolises in the world. We emphasize that it is not straightforward that Zipf’s law, despite its robustness, should hold independently of the city definition, as other scaling relations are not, such as the allometric exponents for CO_2_ emissions and light pollution [24,31]. Many other man-made and natural phenomena also exhibit the same persistent result, e.g. earthquakes and incomes [44,45].

Here, we propose a worldwide model to define urban settlements beyond their usual administrative boundaries through a bottom-up approach that takes into account cultural, political and geographical biases naturally embedded in the population distribution of continental areas. After all, it is not surprising that two regions, e.g. one in western Europe and another one in eastern Asia, spatially contiguous in population or in commuting level have different cultural, political or geographical characteristics. Thus, it is also not surprising that such issues yield different stages of the same mechanics of growth. The main goal of our model is to be successful in defining cities even in large regions. Our conjecture is straightforward: there are hierarchical mechanisms, similar to those present in previous studies of cities in the UK [14] and brain networks [46], behind the growth and innovation of urban settlements. These mechanisms are ruled by a combination of general measures, such as the population and the area of each city, and intrinsic factors which are specific to each region, e.g. topographical heterogeneity, political and economic issues, and cultural customs and traditions. In other words, if political turmoil or economic recession plagues a metropolis for a long time, all of its satellites are affected too, i.e. the entire region ruled by the metropolis will be negatively impacted.

## 2. The models

### 2.1. City clustering algorithm

In 2008, Rozenfeld *et al.* [6] proposed a model to define cities beyond their usual administrative boundaries using a notion of spatial continuity of urban settlements, called the city clustering algorithm (CCA) [6–8,11,15,30,24,31]. The CCA is defined for discrete or continuous landscapes [7] by two parameters: a population density threshold D* and a distance threshold ℓ. These parameters describe the populated areas and the commuting distance between areas, respectively. Here, we adopt the following strategy to improve the discrete CCA performance. (i) Supposing a regular rectangular lattice *L*_*x*_×*L*_*y*_ of sites where the population density of the *k*th site is *D*_*k*_, we perform an initial agglomeration by D* to identify all clusters. If *D*_*k*_>D*, then the *k*th site is populated and we aggregate it with its populated nearest neighbours. Otherwise, the *k*th site is unpopulated. (ii) For each populated cluster, we define its *shell sites*, i.e. sites in the interface between populated and unpopulated areas. (iii) Lastly, we perform a final agglomeration by ℓ, taking into account only the shell sites. If *d*_*ij*_<ℓ, where *d*_*ij*_ is the distance between the *i*th and *j*th shell sites, and if they belong to different clusters, then the *i*th and *j*th sites belong to the same CCA cluster, even with spatial discontinuity. Otherwise, they indeed belong to different CCA clusters. This simple strategy improves the algorithm’s computational performance because the number of shell sites is proportional to *L*, where *L*=*L*_*x*_≈*L*_*y*_ is a linear measure of the lattice.

### 2.2. City local clustering algorithm

We propose a worldwide model based on the CCA, called the city local clustering algorithm (CLCA), not only to define cities beyond their usual administrative boundaries, but also to take into account the intrinsic cultural, political and geographical biases associated with most societies and reflected in their particular growing dynamics. The traditional CCA, with fixed ℓ and D*, when applied to a large population density map, can introduce biases defining a lot of clusters in some regions, while in others just a few. We present the CLCA with the aim of defining cities even in large regions in order to overcome such CCA weakness. Hence, it is possible that other models, such as the models based on street networks proposed by Masucci *et al.* [13] and Arcaute *et al.* [14], carry the same CCA burden and that local adaptations are necessary for their applications into large regions.

**Figure 1.**
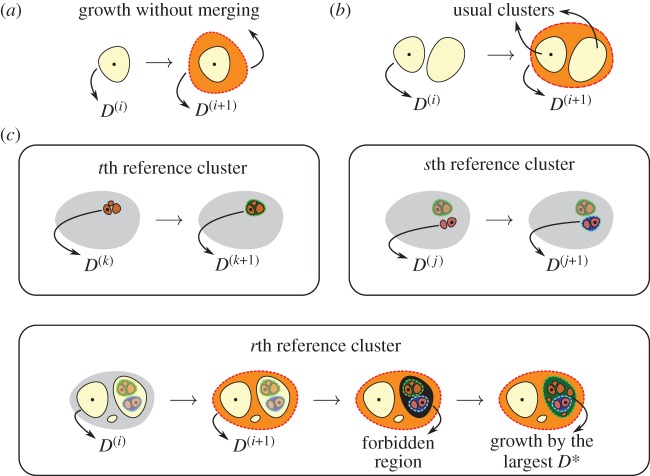
CLCA: representation of the conditions (ii) and (iii). (*a*) The growth of the reference cluster without the merging process. (*b*) The rising of the usual clusters. The usual clusters are the CCA clusters at the imminence of the merging process between *D*^(*i*)^ and *D*^(*i*+1)^. (*c*) For *t*th, *s*th and *r*th reference clusters (*t*th is prior to *s*th which is prior to *r*th), the merging processes are performed as described in (*b*), even though there are clusters already defined close to and within the current analysed region in the second and third case, respectively. In the latter, there is the emergence of a forbidden region. The forbidden regions are the complementary areas of the reference clusters already defined within the usual clusters. In order to define the clusters inside those areas, we force the region defined by the largest value of D* to grow to the limits of the forbidden region. Here, we suppose that *D*^(*j*)^>*D*^(*k*)^. The filled dots stand for the reference sites.

The main idea of our model is to analyse the change of the CCA clusters through the variation of D* under the perspective of different regions. First, we define a regular rectangular lattice *L*_*x*_×*L*_*y*_ of sites, where the population density of the *k*th site is *D*_*k*_. We sort all the sites in a list according to the population density, in descending order. Therefore, the site with the greatest population density is the first entry in this list, which we call the first *reference site*. The reference site can be considered as the current core of the analysed region. Second, we apply the CCA to the lattice, keeping a fixed value of ℓ, for a range of D* decreasing from a maximum value *D*^(max)^ to a minimum value *D*^(min)^ with a decrement *δ*. During the decreasing of D*, clusters are formed and they spread out to all regions of the lattice. Eventually, the cluster that contains the reference site (from now on the *reference cluster*), together with one or more of the other clusters, will merge from *D*^(*i*)^ to *D*^(*i*+1)^, where *D*^(*i*+1)^=*D*^(*i*)^−*δ*. In order to accept or deny the merging of these clusters, we introduce three conditions:

(i) If the area *A*_*r*_(*D*^(*i*)^) of the reference cluster *r*, i.e. the cluster that contains the *r*th reference site at *D*^(*i*)^, obeys
  2.1 $$A_{r}(D^{\left(i\right)})<A^{*},$$
  then the reference cluster *r* always merges with other clusters, because it is still considered very small. In this context, the area A* can be understood as the minimal area of a metropolis.
(ii) If the difference between the areas of the reference cluster *r* at *D*^(*i*+1)^ and *D*^(*i*)^ obeys
  2.2 $$A_{r}(D^{\left(i+1\right)})-A_{r}(D^{\left(i\right)})>H^{*}A_{r}(D^{\left(i\right)}),$$
  then the reference cluster *r* has grown without merging (figure 1*a*) or there is a merging of at least two large clusters (figure 1*b*). In the last case, we emphasize that if there are more than two clusters involved in the merging process, the reference cluster *r* may not be one of the largest. As the first case is not desirable, we can avoid it by reducing the value of *δ* and keeping the value of H* relatively high. The parameter H* can be understood as the percentage of the area of the reference cluster *r* at *D*^(*i*)^. If the second case happens, we consider the entire region inside of the reference cluster *r* at *D*^(*i*+1)^, but the clusters of this region (which we call the *usual clusters*) are defined by those at *D*^(*i*)^. The usual clusters are the CCA clusters at the imminence of the merging process between *D*^(*i*)^ and *D*^(*i*+1)^. This includes the reference cluster *r* itself and one or more of the other clusters before the merging (figure 1*b*). Furthermore, all of the sites of the reference cluster *r* at *D*^(*i*+1)^ are removed from the initial list of reference sites. This condition is necessary because we should not merge two large metropolises.
(iii) In condition (ii), when a reference cluster *r* is merging with another cluster that covers one or more regions already defined by previous reference clusters at different values of D*, there is a strong likelihood of the emergence of a *forbidden region* within that cluster. In this case, we force the region already defined by the largest value of D* to grow to the limits of the forbidden region (figure 1*c*). The forbidden regions are the complementary areas of the reference clusters already defined within the usual clusters. As a consequence of this procedure, some CCA clusters that were hidden after the analysis of the previous reference cluster arise in this forbidden region. We justify this condition by the idea that a metropolis rules the growth of its satellites, as it plays a fundamental role in their socioeconomic relations.

We apply the same procedure to the second reference cluster, to the third reference cluster and so on. Finally, we also define the *isolated clusters* with the minimum value of D* for all the cases accepted in condition (ii). In order to make our model clearer, we chose the descending order to sort the population density for one reason: to favour the merging process of the high-density clusters that arose from the decreasing of D*. In practice, we run our revised discrete CCA just once for the entire range of input parameters and store all of the outputs in order to improve the performance of the model. The apparent simplicity of this task hides a RAM management problem of storing all of the outputs in a medium-performance computer. We overcome such a barrier through the *zram module* [47], available in the newest linux kernels. The zram module creates blocks which compress and store information dynamically in the RAM itself, at the cost of processing time.

## 3. The dataset

**Figure 2.**
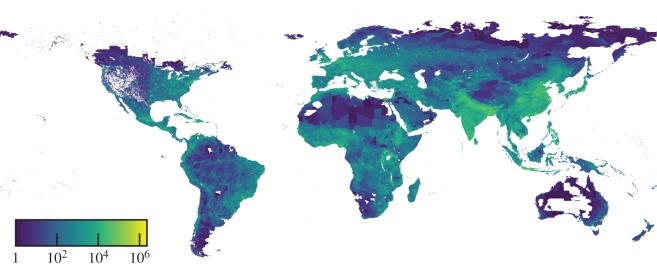
The Global Rural-Urban Mapping Project (GRUMPv1) dataset. The population map of the entire world from the GRUMPv1 dataset in logarithmic scale.

We use the GRUMPv1 [48], available from the Socioeconomic Data and Applications Center (SEDAC) at Columbia University, to apply the CLCA to a single global dataset. The GRUMPv1 dataset is composed of georeferenced rectangular population grids for 232 countries around the world in the year 2000 (figure 2). Such a dataset is a compilation of gridded census and satellite data for the populations of urban and rural areas. These data are provided at a high resolution of 30 arc-seconds, equivalent to 30/3600^°^ or a grid of 0.926×0.926 km at the Equator. We note that despite the heterogeneous population distributions that built the GRUMPv1, its overall resolution is tolerable to the CLCA, since we can identify well-defined clusters around all continents in the raw data.

We calculate the area of each site by the composition of two spherical triangles [49]. The area of a spherical triangle with edges *a*, *b* and *c* is given by

3.1 $$A=4R_{e}^{2}\tan^{-1}{\left[\tan\left(\frac{s}{2}\right)\tan\left(\frac{s_{a}}{2}\right)\tan\left(\frac{s_{b}}{2}\right)\tan\left(\frac{s_{c}}{2}\right)\right]}^{1/2},$$

where *s*=(*a*/*R*_*e*_+*b*/*R*_*e*_+*c*/*R*_*e*_)/2, *s*_*a*_=*s*−*a*/*R*_*e*_, *s*_*b*_=*s*−*b*/*R*_*e*_ and *s*_*c*_=*s*−*c*/*R*_*e*_. In this formalism, *R*_*e*_=6378.137 km is the Earth’s radius and the edge lengths are calculated by the great circle (geodesic) distance between two points *i* and *j* on the Earth’s surface:

3.2 $$d_{ij}=R_{e}\cos^{-1}[\sin(\phi_{i})\sin(\phi_{j})+\cos(\phi_{i})\cos(\phi_{j})\cos(\lambda_{j}-\lambda_{i})].$$

The values of *λ*_*i*_ (*λ*_*j*_) and *ϕ*_*i*_ (*ϕ*_*j*_), measured in radians, are the longitude and latitude, respectively, of the point *i* (*j*). Thus, we are able to define the population density for each site of the lattice, since its population and area are known.

**Figure 3.**
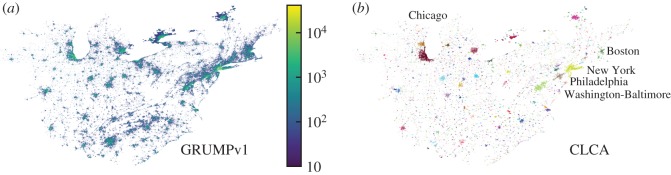
The largest cluster of regions for the USA. (*a*) The single population density cluster from the eastern USA is defined by the CCA with lower and upper bound parameters D*=50 people km^−2^ and ℓ=10 km, respectively. The population, provided by the GRUMPv1 dataset, is shown in logarithmic scale within each populated area. (*b*) Application of the CLCA for the cluster of regions of the eastern USA. The CLCA cities are represented in several colours, e.g. New York in mustard, Philadelphia in light brown, Washington-Baltimore in light green, Boston in green and Chicago in red. The CLCA parameters used were *D*^(min)^=100 people km^−2^, *D*^(max)^=1000 people km^−2^, *δ*= 10 people km^−2^, ℓ=3 km, A*=50 km^2^ and H*=0.05.

We also pre-process the GRUMPv1 dataset, dividing all countries and continents—and even the entire world—into large regions which we call *clusters of regions*, to apply our model in a feasible computational time using medium-performance computers. These regions are defined by the CCA with lower and upper bound parameters D*=50 people km^−2^ and ℓ=10 km, respectively. We believe that such large clusters can hold the socioeconomic and cultural relations among different urban settlements of a territory. Figure 3*a* shows the largest clusters of regions in the USA; as we can see, all of the eastern USA is considered a single cluster.

## 4. Results

To show the relevance of our model, we apply the CLCA to the GRUMPv1 dataset at three different geographical levels: countries, continents and the entire world. For each case, we consider only a single set of CLCA parameters. We justify our choices with the following assumptions: (i) *D*^(min)^=100 people km^−2^, a value slightly greater than the lower bound CCA parameter (D*=50 people km^−2^) used to define the regions of clusters; (ii) *D*^(max)^=1000 people km^−2^, a loosened value of $D^{\left(\max\right)}=\infty$; (iii) *δ*=10 people km^−2^, a small enough value to avoid the reference clusters growing without merging; (iv) ℓ=3 km, the critical distance threshold, already extensively analysed by previous CCA studies [6,7,24]; (v) A*=50 km^2^, the minimum area of a metropolis, as it is required that A* be reasonably greater than the minimum unit of area from the dataset and smaller than a metropolis’ area; and (vi) H*=0.05, a large enough value to favour the merging of clusters which are similar in size. Figure 3*b* shows the CLCA cities defined by the single set of CLCA parameters. For other regions, see the electronic supplementary material.

**Figure 4.**
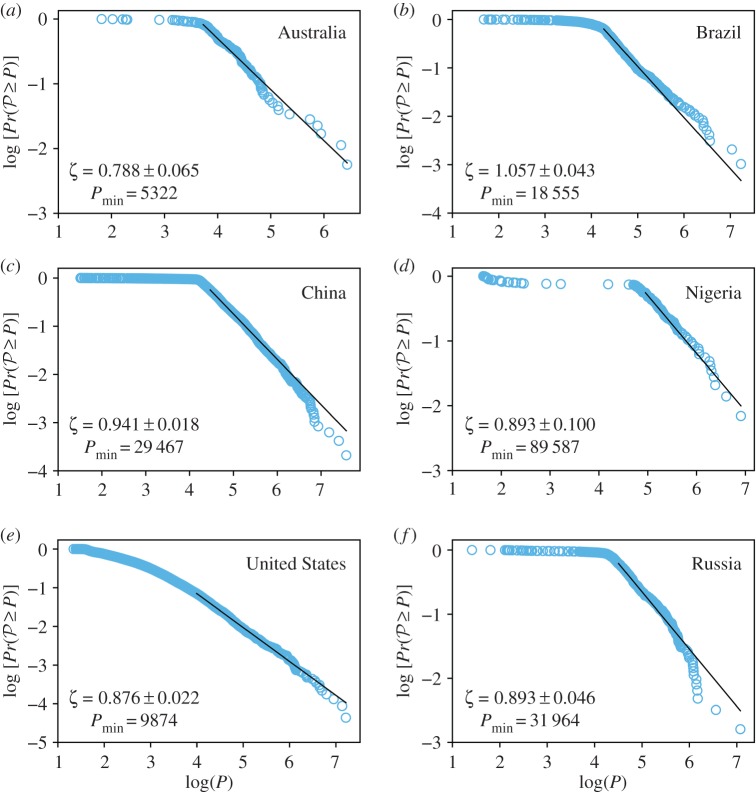
CDF Pr(P≥P) versus population *P*, in log–log scale, for the countries with the highest number of cities in each continent (for other countries, see the electronic supplementary material). (*a*–*f*) Cities proposed by the CLCA are represented by light blue circles. The solid black line is the maximum-likelihood power-law fit defined by the MLE [50]. The value of the lower bound $P_{\min}$ and the exponent *ζ* are also shown. The CLCA parameters used were *D*^(min)^=100 people km^−2^, *D*^(max)^= 1000 people km^−2^, *δ*=10 people km^−2^, ℓ= 3 km, A*=50 km^2^ and H*=0.05.

We study the population distribution using the maximum-likelihood estimator (MLE) proposed by Clauset *et al.* [50]. Their approach combines maximum-likelihood fitting methods with goodness-of-fit tests based on Kolmogorov–Smirnov statistic. Figure 4 shows the log–log behaviour of the cumulative distribution function (CDF) for the population of the CLCA cities, considering only the countries with the highest number of CLCA cities for each continent (for other countries, see the electronic supplementary material). The Pr(P≥P) represents the probability that a random population P takes on a value greater than or equal to the population *P*. In all CDF plots, we also show the maximum-likelihood power-law fit, as well as the value of the exponent *ζ*=*α*−1, where *α* is the MLE exponent, and the value of $P_{\min}$, the lower bound of the MLE.

**Figure 5.**
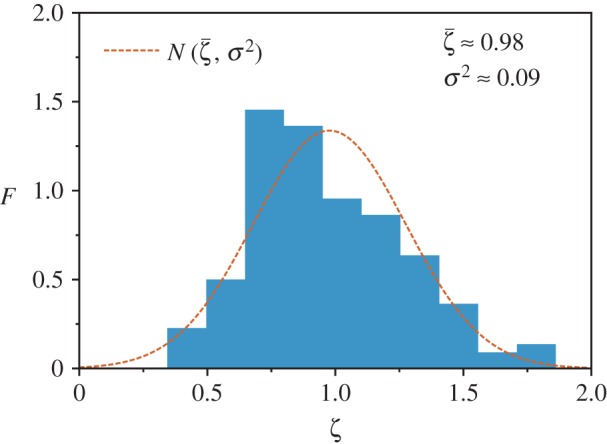
Normalized histogram, with frequency *F*, of the *ζ* exponent at the country level. The plot shows those countries (145 out of 232) with at least 10 cities defined by the CLCA in the region covered by the maximum-likelihood power-law fit. We find the mean value of the Zipf exponents $\bar{\zeta }=0.98$ and its variance *σ*^2^=0.09. The dashed red line stands for the normal distribution $N(\bar{\zeta },\sigma^{2})$. Therefore, Zipf’s law holds for most countries.

In figure 5, we show a normalized histogram, with frequency *F*, of the *ζ* exponents for all countries (145 out of 232) with at least 10 CLCA cities in the region covered by the maximum-likelihood power-law fit. The mean value of the *ζ* exponents is $\bar{\zeta }=0.98$, with variance *σ*^2^=0.09. The dashed red line stands for the normal distribution $N(\bar{\zeta },\sigma^{2})$. In spite of the *ζ* exponent heterogeneity illustrated by figure 5, Zipf’s law holds for most countries around the globe. We emphasize that such results corroborate with previous studies performed for one country or a small number of countries [6,7,32–42]. In particular, the figure 5 also endorses an astute meta-analysis performed by Cottineau [51]. Cottineau provided a comparison among Zipf’s law exponents found in 86 studies. Our results strongly corroborate those presented in such study, except that our exponents are ranged between 0 and 2.

**Figure 6.**
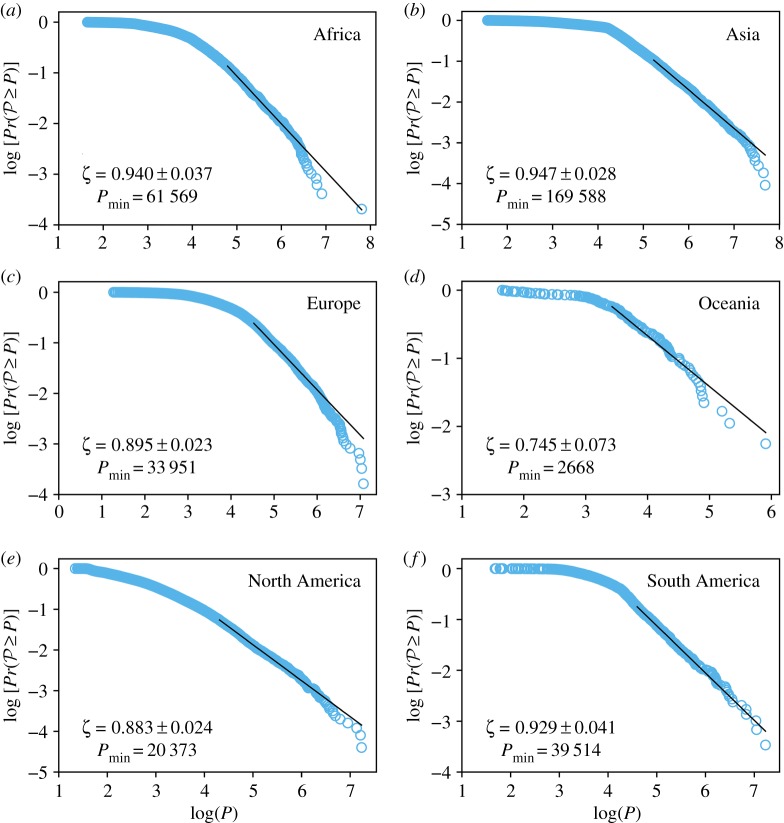
CDF Pr(P≥P) versus population *P*, in log–log scale, for the continents. (*a*–*f*) Cities proposed by the CLCA are represented by light blue circles. The solid black line is the maximum-likelihood power-law fit defined by the MLE [50]. The value of the lower bound $P_{\min}$ and the exponent *ζ* are also shown. The CLCA parameters used were *D*^(min)^=100 people km^−2^, *D*^(max)^= 1000 people km^−2^, *δ*=10 people km^−2^, ℓ= 3 km, A*=50 km^2^ and H*=0.05.

**Figure 7.**
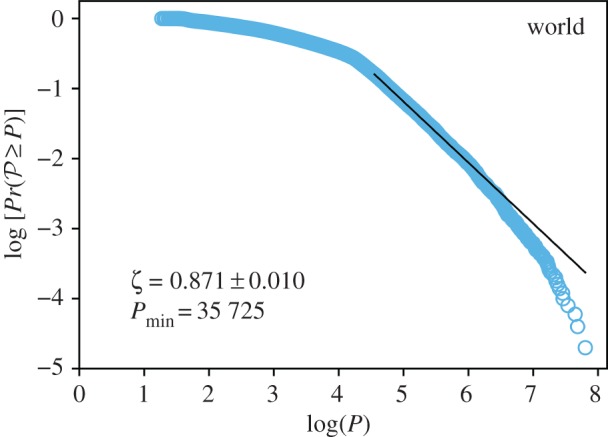
CDF Pr(P≥P) versus population *P*, in log–log scale, for the entire world. (*a*–*f*) Cities proposed by the CLCA are represented by light blue circles. The solid black line is the maximum likelihood power-law fit defined by the MLE [50]. The value of the lower bound $P_{\min}$ and the exponent *ζ* are also shown. The CLCA parameters used were *D*^(min)^=100 people km^−2^, *D*^(max)^= 1000 people km^−2^, *δ*=10 people km^−2^, ℓ= 3 km, A*=50 km^2^ and H*=0.05.

Furthermore, we challenge the robustness of our model at higher geographical levels: continents and the entire world. We performed the same analyses and find that our results persist on both scales, i.e. the CLCA cities follow Zipf’s law for continents and the entire world, as illustrated in figures 6 and 7.

We summarize our results in a set of seven tables: tables 1–6, for countries from Africa, Asia, Europe, North America, Oceania and South America, respectively. Table 7 contains similar information for all continents and the entire world. In all cases, we show the name of the considered region (country, continent or globe), the ISO 3166-1 alpha-3 code associated (only for countries), the number of cities obtained by the CLCA and those covered by the MLE, the lower bound $P_{\min}$ and the Zipf exponent *ζ*.

**Table 1.** *African countries.* We show the name, the ISO 3166-1 alpha-3 code, the number of cities obtained by the CLCA and the number of those covered by the maximum-likelihood power-law fit defined by the MLE [50] (represented by †), the lower bound $P_{\min}$, and the Zipf exponent *ζ*.

country	ISO	CLCA cities	CLCA cities^†^	$P_{\min}$	*ζ*
Angola	AGO	20	16	43 937	0.780 ± 0.195
Benin	BEN	40	30	12 607	0.780 ± 0.142
Burkina Faso	BFA	139	78	12 314	1.256 ± 0.142
Botswana	BWA	79	58	1674	0.785 ± 0.103
Central African Republic	CAF	37	11	14 868	1.230 ± 0.371
Ivory Coast	CIV	83	47	18 400	0.962 ± 0.140
Cameroon	CMR	143	93	7478	0.711 ± 0.074
Democratic Republic of the Congo	COD	191	47	25 996	0.764 ± 0.111
Congo	COG	21	18	17 673	1.050 ± 0.248
Comoros	COM	16	15	4167	0.922 ± 0.238
Cape Verde	CPV	16	11	5205	1.083 ± 0.327
Algeria	DZA	273	112	24 192	0.910 ± 0.086
Egypt	EGY	19	12	11 967	0.511 ± 0.147
Eritrea	ERI	27	12	6559	0.730 ± 0.211
Ethiopia	ETH	244	147	6638	0.688 ± 0.057
Gabon	GAB	33	27	3108	0.844 ± 0.162
Ghana	GHA	95	25	54 662	1.145 ± 0.229
Guinea	GIN	34	13	40 118	1.234 ± 0.342
Gambia	GMB	35	33	1186	0.610 ± 0.106
Guinea-Bissau	GNB	26	14	9148	1.139 ± 0.305
Kenya	KEN	179	20	72 756	1.383 ± 0.309
Liberia	LBR	42	19	6468	0.604 ± 0.139
Libyan Arab Jamahiriya	LBY	30	18	40 273	1.180 ± 0.278
Lesotho	LSO	14	11	1999	0.651 ± 0.196
Morocco (includes Western Sahara)	MAR	58	50	26 325	0.763 ± 0.108
Madagascar	MDG	138	74	14 867	1.340 ± 0.156
Mali	MLI	152	146	4463	1.161 ± 0.096
Mozambique	MOZ	127	14	128 214	1.861 ± 0.497
Malawi	MWI	179	72	4194	0.779 ± 0.092
Namibia	NAM	31	17	12 467	1.637 ± 0.397
Niger	NER	58	36	10 717	0.753 ± 0.126
Nigeria	NGA	144	80	89 587	0.893 ± 0.100
Sudan	SDN	77	56	39 764	1.031 ± 0.138
Senegal	SEN	42	34	13 475	0.798 ± 0.137
Sierra Leone	SLE	62	52	1899	0.612 ± 0.085
Chad	TCD	75	14	19 574	1.086 ± 0.290
Togo	TGO	54	11	82 964	1.667 ± 0.503
Tunisia	TUN	46	36	16 130	1.014 ± 0.169
United Republic of Tanzania	TZA	114	33	73 621	0.936 ± 0.163
Uganda	UGA	155	33	30 587	1.386 ± 0.241
South Africa	ZAF	1915	97	53 320	1.270 ± 0.129
Zambia	ZMB	55	34	7118	0.666 ± 0.114
Zimbabwe	ZWE	28	24	13 411	0.746 ± 0.152

**Table 2.** *Asian countries.* We show the name, the ISO 3166-1 alpha-3 code, the number of cities obtained by the CLCA and the number of those covered by the maximum-likelihood power-law fit defined by the MLE [50] (represented by †), the lower bound $P_{\min}$, and the Zipf exponent *ζ*.

country	ISO	CLCA cities	CLCA cities^†^	$P_{\min}$	*ζ*
Afghanistan	AFG	95	38	29 242	0.809 ± 0.131
Armenia	ARM	41	19	17 088	1.256 ± 0.288
Azerbaijan	AZE	34	21	17 169	0.776 ± 0.169
Bangladesh	BGD	103	58	26 586	0.581 ± 0.076
Bhutan	BTN	19	15	893	0.469 ± 0.121
China	CHN	4782	2706	29 467	0.941 ± 0.018
Cyprus	CYP	17	15	626	0.486 ± 0.126
Georgia	GEO	52	38	6526	0.765 ± 0.124
Indonesia	IDN	2416	542	12 876	0.894 ± 0.038
India	IND	1040	299	94 976	0.786 ± 0.045
Iran	IRN	169	56	100 763	1.194 ± 0.160
Israel	ISR	24	20	877	0.448 ± 0.100
Jordan	JOR	13	11	15 253	0.803 ± 0.242
Japan	JPN	270	33	289 039	1.011 ± 0.176
Kazakhstan	KAZ	77	22	103 289	1.505 ± 0.321
Kyrgyz Republic	KGZ	134	37	9117	0.991 ± 0.163
Cambodia	KHM	84	24	34 495	1.735 ± 0.354
Korea	KOR	131	23	126 819	0.750 ± 0.156
Lao People’s Democratic Republic	LAO	35	20	12 595	0.958 ± 0.214
Sri Lanka	LKA	23	20	8573	0.466 ± 0.104
Maldives	MDV	149	40	1498	1.799 ± 0.285
Myanmar	MMR	115	37	69 935	1.190 ± 0.196
Mongolia	MNG	24	19	13 179	1.419 ± 0.325
Malaysia	MYS	119	15	157 843	1.286 ± 0.332
Nepal	NPL	39	22	15 396	0.560 ± 0.119
Oman	OMN	28	12	34 956	1.519 ± 0.438
Pakistan	PAK	96	45	90 356	0.790 ± 0.118
Philippines	PHL	352	38	106 854	1.195 ± 0.194
Democratic People’s Republic of Korea	PRK	53	20	174 121	1.502 ± 0.336
Saudi Arabia	SAU	57	15	156 672	0.861 ± 0.222
Syrian Arab Republic	SYR	39	20	29 908	0.647 ± 0.145
Thailand	THA	100	24	23 482	0.718 ± 0.147
Tajikistan	TJK	39	13	17 660	0.740 ± 0.205
Turkmenistan	TKM	30	14	26 319	0.883 ± 0.236
East Timor	TLS	23	15	1220	0.547 ± 0.141
Turkey	TUR	338	244	18 389	0.926 ± 0.059
Taiwan	TWN	16	13	2186	0.344 ± 0.095
Uzbekistan	UZB	56	36	15 865	0.574 ± 0.096
Vietnam	VNM	345	72	35 980	0.876 ± 0.103
Yemen	YEM	46	22	38 276	1.059 ± 0.226

**Table 3.** *European countries.* We show the name, the ISO 3166-1 alpha-3 code, the number of cities obtained by the CLCA and the number of those covered by the maximum-likelihood power-law fit defined by the MLE [50] (represented by †), the lower bound $P_{\min}$ and the Zipf exponent *ζ*.

country	ISO	CLCA cities	CLCA cities^†^	$P_{\min}$	*ζ*
Albania	ALB	46	32	6030	0.783 ± 0.139
Austria	AUT	116	74	4383	0.754 ± 0.088
Belgium	BEL	43	31	9800	0.706 ± 0.127
Bulgaria	BGR	56	29	33 338	1.308 ± 0.243
Bosnia-Herzegovina	BIH	57	17	15 708	1.186 ± 0.288
Belarus	BLR	36	17	73 682	1.123 ± 0.272
Switzerland	CHE	71	15	55 878	1.167 ± 0.301
Czech Republic	CZE	206	33	41 254	1.393 ± 0.243
Germany	DEU	331	242	13 926	0.811 ± 0.052
Denmark	DNK	134	85	2248	0.682 ± 0.074
Spain	ESP	358	36	133 759	1.192 ± 0.199
Estonia	EST	51	13	14 041	1.178 ± 0.327
Finland	FIN	72	22	27 831	1.444 ± 0.308
France	FRA	1253	114	42 160	1.087 ± 0.102
United Kingdom	GBR	214	22	229 133	0.983 ± 0.210
Greece	GRC	320	93	7639	0.930 ± 0.096
Croatia	HRV	88	40	9672	1.085 ± 0.172
Hungary	HUN	143	25	34 474	1.189 ± 0.238
Ireland	IRL	189	62	4775	1.093 ± 0.139
Iceland	ISL	15	12	708	0.560 ± 0.162
Italy	ITA	400	157	19 724	0.885 ± 0.071
Lithuania	LTU	76	32	10 654	1.007 ± 0.178
Latvia	LVA	75	28	9276	1.107 ± 0.209
Republic of Moldova	MDA	31	23	6609	0.570 ± 0.119
Macedonia	MKD	45	23	11 001	0.981 ± 0.205
The Netherlands	NLD	69	16	112 058	1.288 ± 0.322
Norway	NOR	105	18	21 795	1.214 ± 0.286
Poland	POL	236	160	17 390	0.903 ± 0.071
Portugal	PRT	139	32	17 110	1.027 ± 0.182
Romania	ROU	522	385	3129	0.740 ± 0.038
Russia	RUS	622	384	31 964	0.893 ± 0.046
Serbia and Montenegro	SCG	60	27	38 415	1.340 ± 0.258
Slovakia	SVK	88	20	35 068	1.468 ± 0.328
Slovenia	SVN	88	32	3273	0.730 ± 0.129
Sweden	SWE	168	61	11 449	1.008 ± 0.129
Ukraine	UKR	164	107	36 515	0.833 ± 0.081

**Table 4.** *North American countries.* We show the name, the ISO 3166-1 alpha-3 code, the number of cities obtained by the CLCA and the number of those covered by the maximum-likelihood power-law fit defined by the MLE [50] (represented by †), the lower bound $P_{\min}$ and the Zipf exponent *ζ*.

country	ISO	CLCA cities	CLCA cities^†^	$P_{\min}$	*ζ*
Canada	CAN	1135	308	4879	0.815 ± 0.046
Costa Rica	CRI	14	11	20 751	1.195 ± 0.360
Cuba	CUB	113	46	34 673	1.327 ± 0.196
Guatemala	GTM	25	14	28 353	0.948 ± 0.253
Honduras	HND	236	35	17 120	1.290 ± 0.218
Haiti	HTI	23	18	21 953	0.897 ± 0.211
Mexico	MEX	474	284	11 992	0.726 ± 0.043
Nicaragua	NIC	31	28	9802	0.821 ± 0.155
Panama	PAN	40	12	17 717	1.089 ± 0.314
El Salvador	SLV	25	13	21 323	0.816 ± 0.226
United States	USA	22 893	1624	9874	0.876 ± 0.022

**Table 5.** *Oceanian countries.* We show the name, the ISO 3166-1 alpha-3 code, the number of cities obtained by the CLCA and the number of those covered by the maximum-likelihood power-law fit defined by the MLE [50] (represented by †), the lower bound $P_{\min}$ and the Zipf exponent *ζ*.

country	ISO	CLCA cities	CLCA cities^†^	$P_{\min}$	*ζ*
Australia	AUS	177	145	5332	0.788 ± 0.065
Fiji	FJI	15	14	936	0.807 ± 0.216
Marshall Islands	MHL	28	27	44	0.760 ± 0.146
New Zealand	NZL	108	79	3077	0.776 ± 0.087
Papua New Guinea	PNG	30	13	13 828	1.479 ± 0.410

**Table 6.** *South American countries.* We show the name, the ISO 3166-1 alpha-3 code, the number of cities obtained by the CLCA and the number of those covered by the maximum-likelihood power-law fit defined by the MLE [50] (represented by †), the lower bound $P_{\min}$ and the Zipf exponent *ζ*.

country	ISO	CLCA cities	CLCA cities^†^	$P_{\min}$	*ζ*
Argentina	ARG	749	227	10 880	0.994 ± 0.066
Bolivia	BOL	83	57	6729	0.841 ± 0.111
Brazil	BRA	966	613	18 555	1.057 ± 0.043
Chile	CHL	59	19	93 915	1.422 ± 0.326
Colombia	COL	402	163	12 890	0.886 ± 0.069
Ecuador	ECU	94	54	12 717	0.832 ± 0.113
Peru	PER	417	153	8279	0.867 ± 0.070
Paraguay	PRY	29	26	4928	0.700 ± 0.137
Uruguay	URY	79	16	23 346	1.310 ± 0.327
Venezuela	VEN	81	28	82 323	1.254 ± 0.237

**Table 7.** *Continents and the entire world.* We show the name, the number of cities obtained by the CLCA and the number of those covered by the maximum-likelihood power-law fit defined by the MLE [50] (represented by †), the lower bound $P_{\min}$ and the Zipf exponent *ζ*.

continent/globe	CLCA cities	CLCA cities^†^	$P_{\min}$	*ζ*
Africa	4860	660	61 569	0.940 ± 0.037
Asia	10 953	1167	169 588	0.947 ± 0.028
Europe	6118	1489	33 951	0.895 ± 0.023
Oceania	180	103	2668	0.745 ± 0.073
North America	24 919	1364	20 373	0.883 ± 0.024
South America	2934	522	39 514	0.929 ± 0.041
world (except Antarctica)	50 314	8019	35 725	0.871 ± 0.010

**Figure 8.**
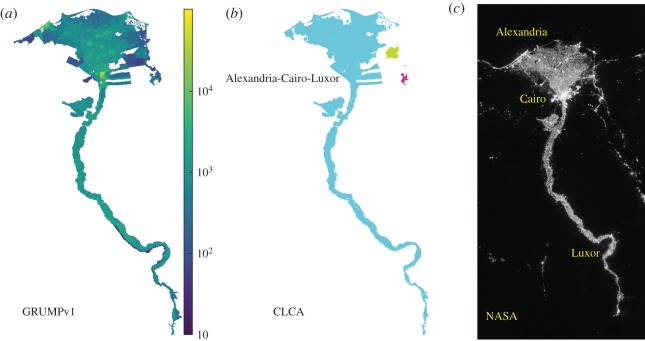
Northeastern region of Egypt. (*a*) The cluster of regions defined by the pre-processing of the GRUMPv1 dataset for the northeastern region of Egypt. (*b*) The largest city defined by the CLCA in the entire world is formed by several cities, including Alexandria, Cairo and Luxor. (*c*) Night-time lights of the northeast of Egypt provided by National Aeronautics and Space Administration (NASA). The CLCA cities found exhibit a remarkable similarity with the lights across the Nile.

It is remarkable that the top CLCA city, with a population of 63 585 039 people, is composed of three large urban settlements (Alexandria, Cairo and Luxor) connected by several small ones. Figure 8*a*–*c* shows the largest cluster of regions in Egypt for the GRUMPv1 dataset, CLCA cities and night-time lights from the National Aeronautics and Space Administration (NASA) [52], respectively. We believe the main reason for this finding has been present in the northeast of Africa since before the beginning of ancient civilization—namely, the Nile river. Actually, it is well known that almost the entire Egypt population lives in a strip along the Nile river, in the Nile delta and in the Suez canal on 4% of the total country area (10^6^ km^2^), where there are arable lands to produce food [53]. The river and delta regions are composed by some large cities and a lot of small villages, making them extremely dense. Therefore, our results raise the hypothesis that the cities and villages across the Nile can be seen as a kind of ‘megacity’, despite spatially non-contiguous, due to the socioeconomic relation, reflected in the high commuting levels, among close subregions.

Table 8 shows the top 25 CLCA cities in the entire world by population, and their associated areas. After the top CLCA city, Alexandria-Cairo-Luxor, we emphasize that the 13 next-largest CLCA cities are in Asia. Indeed, we can see that the shape of the tail end of the entire world population distribution (in figure 7) is roughly ruled by the greater CLCA city in Africa and several CLCA cities in Asia.

**Table 8.** *Top 25 cities, by population, in the world.* We emphasize that, after the top CLCA city (Alexandria-Cairo-Luxor), the 13 next-largest CLCA cities are in Asia. The largest United Nation city, Tokyo, is just the 4th according to our analyses.

CLCA city	country	CLCA population (people)	CLCA area (*km*^2^)
Alexandria-Cairo-Luxor	Egypt	63 585 039	34 434
Dhaka	Bangladesh	48 419 117	26 963
Guangzhou-Macau-Hong Kong	China	44 384 647	12 896
Tokyo	Japan	34 318 072	9189
Kolkota	India	28 876 910	10 408
Patna	India	28 484 380	18 670
Xi’an	China	25 370 875	39 736
Jakarta-Bekasi-Banten	Indonesia	23 814 197	5862
Hanoi-Hai Phong	Vietnam	22 480 083	19 128
New Delhi	India	22 136 675	6914
Seoul	South Korea	20 318 881	3610
Mumbai	India	18 431 960	2443
Manila	Philippines	17 591 794	4039
Mexico City	Mexico	17 190 725	2845
São Paulo	Brazil	16 984 627	2840
Kyoto-Osaka-Kobe	Japan	16 398 829	4608
New York City	USA	16 364 109	4471
Shangai	China	15 291 143	2529
Kochi-Kottayam-Kollam	India	14 551 809	8091
Surabaya-Gresik-Malang	Indonesia	14 289 547	6891
Los Angeles	USA	13 615 610	5167
Cirebon-Tegal-Kebumen	Indonesia	12 758 617	6818
Semarang-Klaten-Surakarta	Indonesia	12 456 408	6418
Moscow	Russia	11 894 034	1448
Buenos Aires	Argentina	11 132 081	2653

These facts are not in line with what was recently reported by the United Nations (UN) [54], e.g. the largest CLCA city, Alexandria-Cairo-Luxor, is just the 9th largest city according to the UN, and the largest UN city, Tokyo, is just the 4th largest according to our analyses.

## 5. Conclusion

We propose a model to define urban settlements through a bottom-up approach beyond their usual administrative boundaries, and moreover to account for the intrinsic cultural, political and geographical biases associated with most societies and reflected in their particular growing dynamics. We claim that such a property qualifies our model to be applied worldwide, without any regional restrictions. We also propose an alternative strategy to improve the computational performance of the discrete CCA. We emphasize that the CCA can still be used to define cities; however, it depends upon a different tuning of its parameters for each large region without direct socioeconomic and political relations. Furthermore, we show that the definition of cities proposed by our approach is robust and holds to one of the most famous results in social science, Zipf’s law, not only for previously studied countries, e.g. the USA, the UK or China, but for all countries (145 from 232 provided by GRUMPv1) around the world. We also find that Zipf’s law emerges at different geographical levels, such as continents and the entire world. Another highlight of our study is the fact that our model is applied upon one single dataset to define all cities. Furthermore, we find that the most populated cities are not the major players in the global economy (such as New York City, London or Tokyo). The largest CLCA city, with a population of 63 585 039 people, is an agglomeration of several small cities close to each other which connects three large cities: Alexandria, Cairo and Luxor. Finally, after the top CLCA city of Alexandria-Cairo-Luxor, we find that the next-largest 13 CLCA cities are in Asia. These facts are not in full agreement with a recent UN report [54]. According to our results, the largest CLCA city, Alexandria-Cairo-Luxor, is just the 9th largest city according to the UN, while the largest UN city, Tokyo, is just the 4th largest according to our analyses.

## Supplementary Material

Supplementary Information: A worldwide model for boundaries of urban settlements

## Data accessibility

The data supporting this article are available at http://sedac.ciesin.columbia.edu/data/collection/grump-v1. More specifically, the reader can click on ‘Data sets’ and, after that, on ‘Population Count Grid, v1 (1990,1995,2000)’. We also provide the codes for the proposed model that are available at https://doi.org/10.5061/dryad.968nq8n [55].
