# Supplementary material for: A worldwide model for boundaries of urban settlements
Source: R Soc Open Sci. 2018 May 23;5(5):180468. doi: 10.1098/rsos.180468 (PMC5990827; doi:10.1098/rsos.180468)
Supplement: Supplementary Information: A worldwide model for boundaries of urban settlements [file rsos180468supp1.pdf]

Royal Society Open Science

Supplementary Information:

A worldwide model for boundaries of urban settlements

Erneson A. Oliveira[1,2,3a], Vasco Furtado[1], José S. Andrade Jr.[3], Hernán A. Makse[3,4]

[1] *Programa de Pós Graduação em Informática Aplicada,*

Universidade de Fortaleza, 60811-905 Fortaleza, Ceará, Brasil.

[2] *Mestrado Profissional em Ciências da Cidade,*

Universidade de Fortaleza, 60811-905 Fortaleza, Ceará, Brasil.

[3] *Departamento de Física, Universidade Federal do Ceará,*

Campus do Pici, 60451-970 Fortaleza, Ceará, Brasil.

[4] *Levich Institute and Physics Department,*

City College of New York, New York, New York 10031, USA.

(Dated: March 31, 2018)

[a] Correspondence to: erneson@fisica.ufc.br

I. THE TOP 25 CLCA CITIES

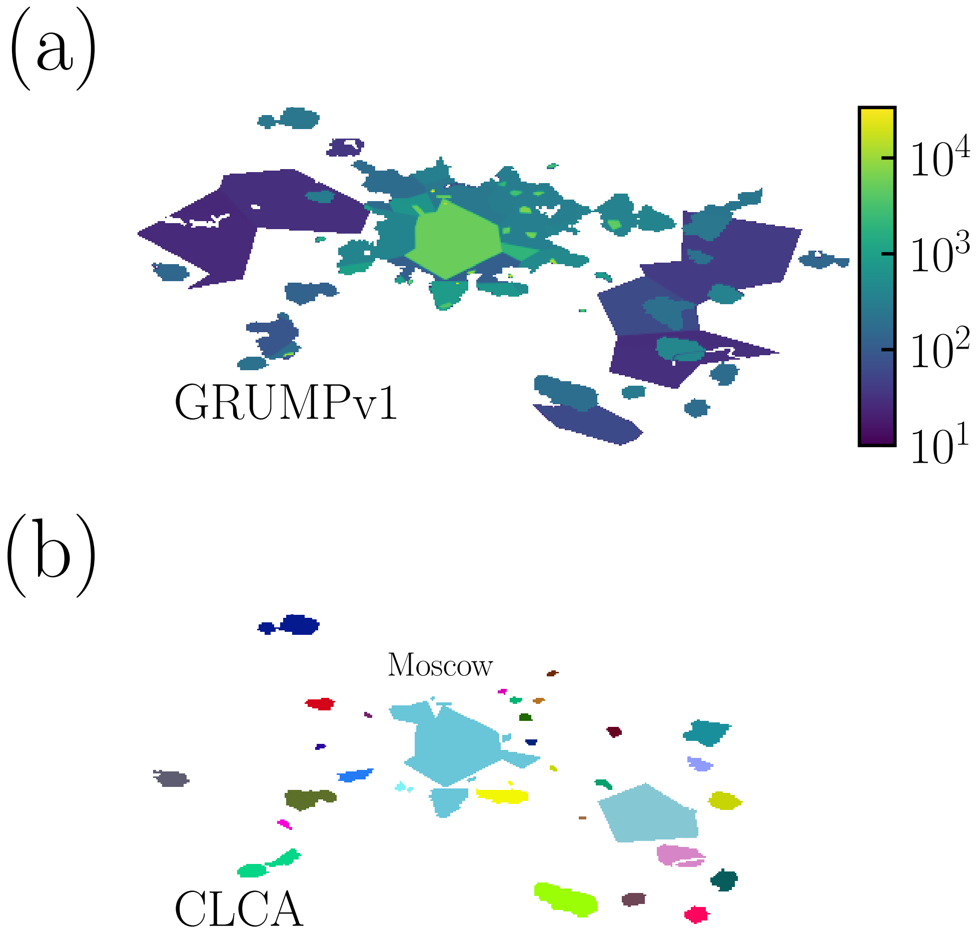

FIG. 1. **(Color online) CLCA cities.** (a) The population map of the cluster of regions. (b) Cities defined by the CLCA.

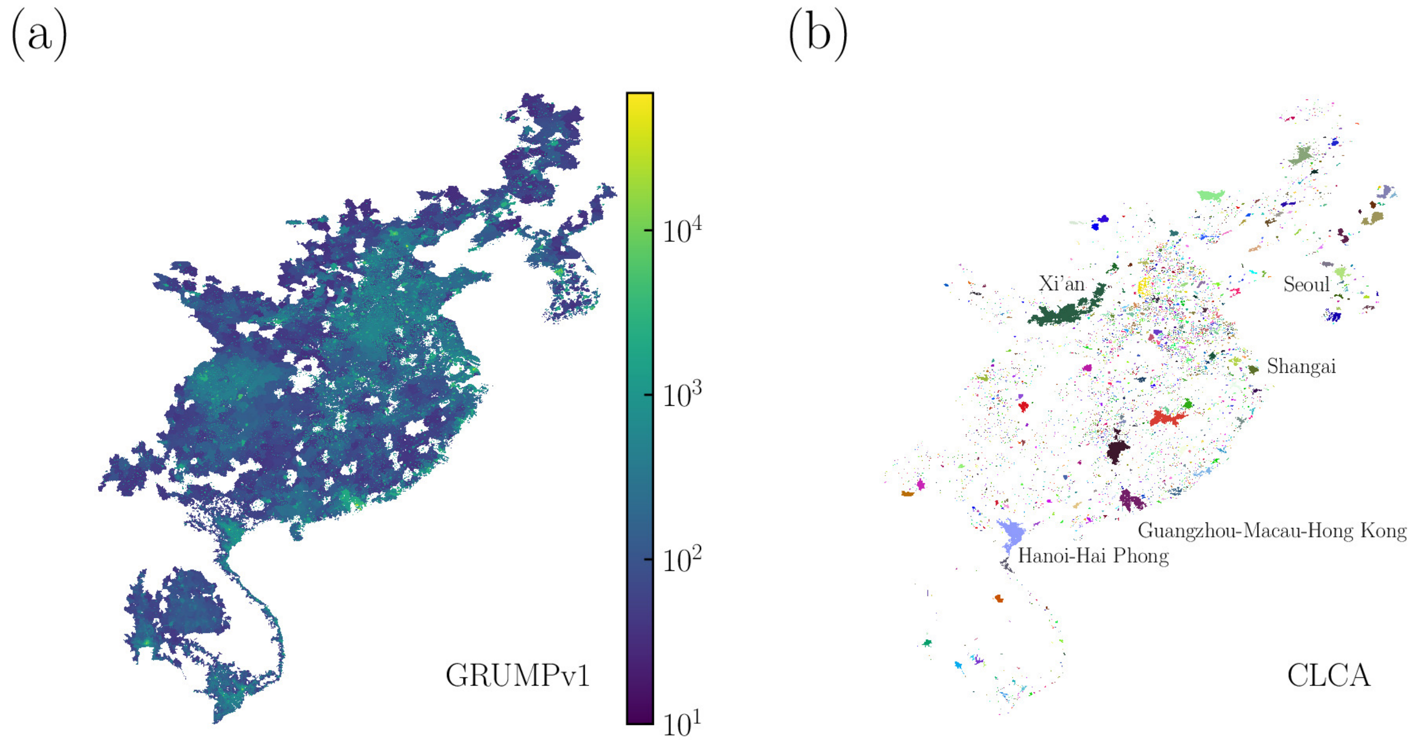

FIG. 2. **(Color online) CLCA cities.** (a) The population map of the cluster of regions. (b) Cities defined by the CLCA.

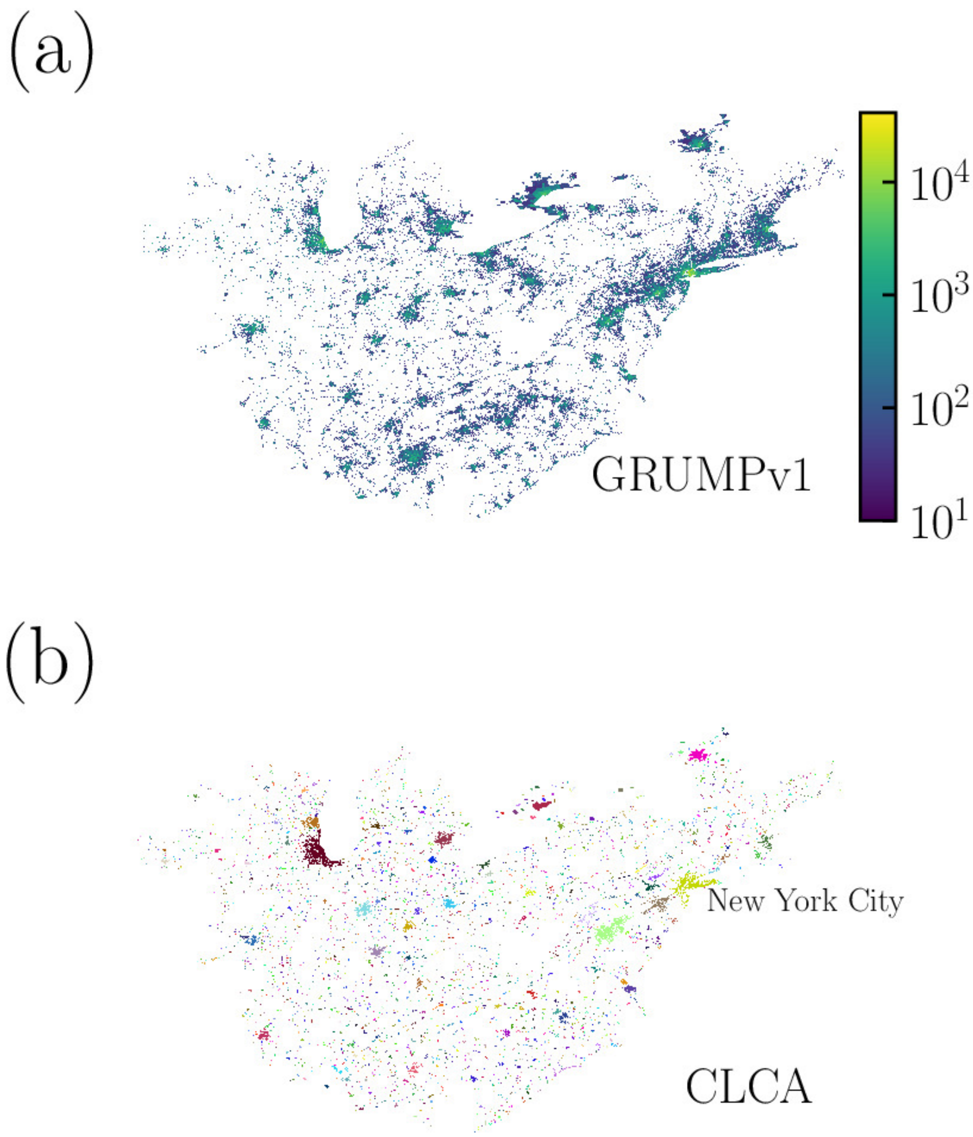

FIG. 3. **(Color online) CLCA cities.** (a) The population map of the cluster of regions. (b) Cities defined by the CLCA.

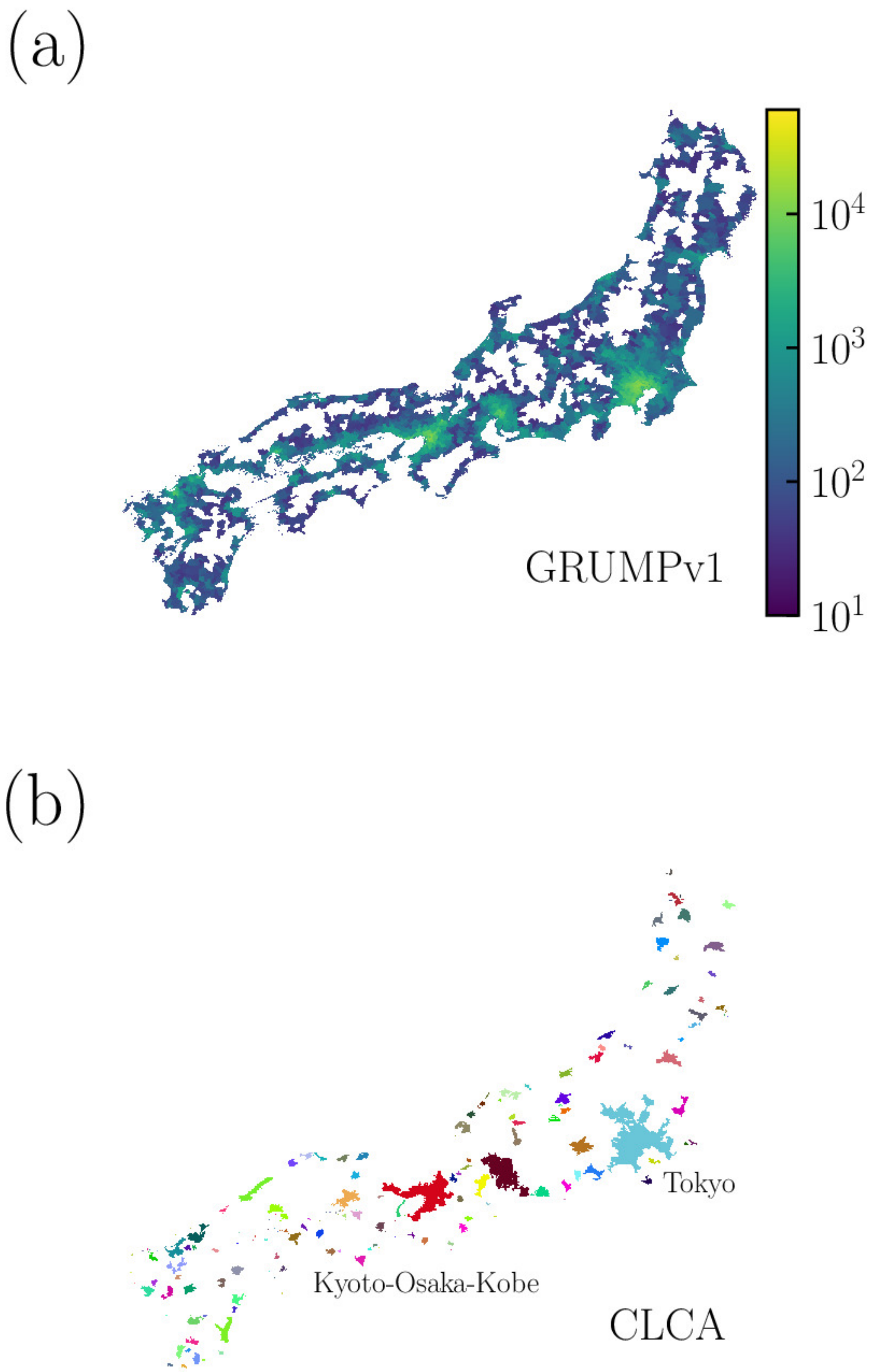

FIG. 4. **(Color online) CLCA cities.** (a) The population map of the cluster of regions. (b) Cities defined by the CLCA.

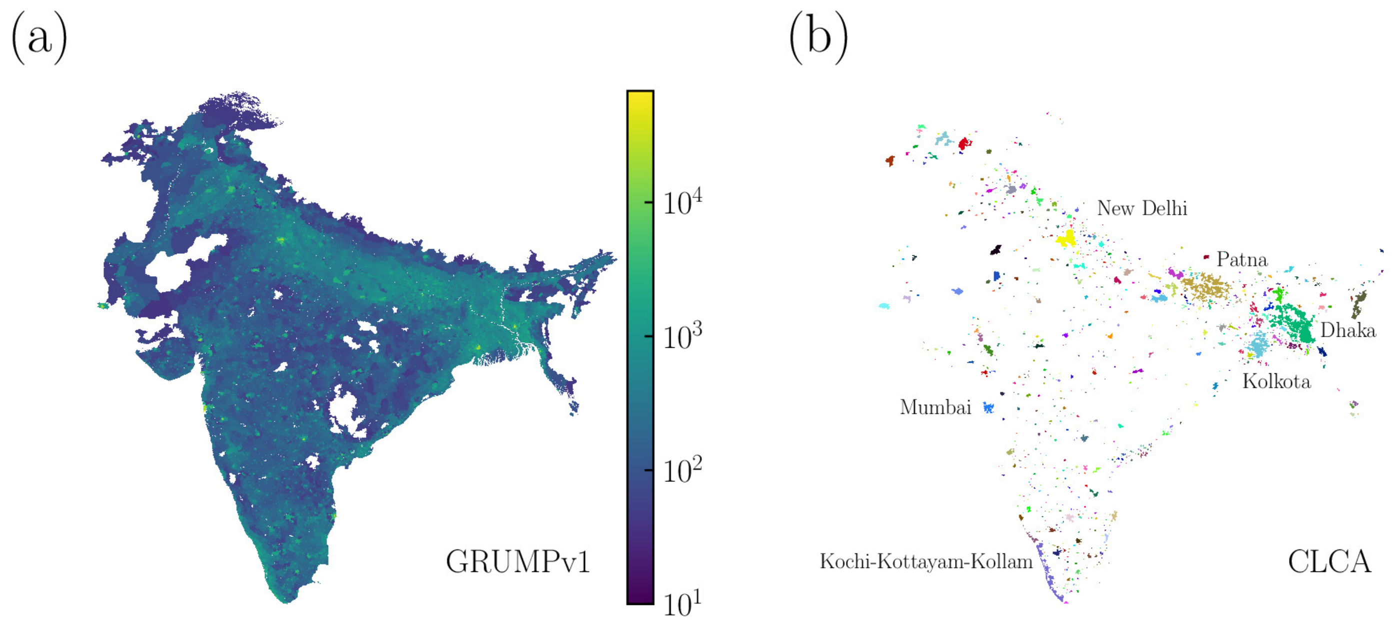

FIG. 5. **(Color online) CLCA cities.** (a) The population map of the cluster of regions. (b) Cities defined by the CLCA.

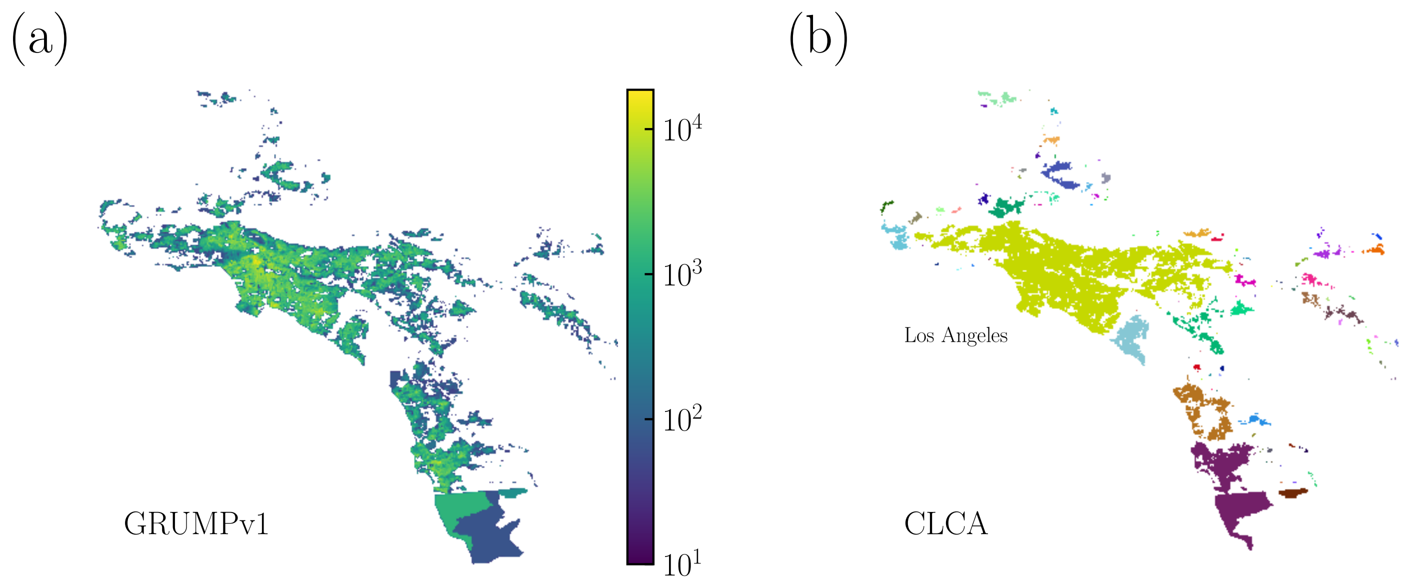

FIG. 6. **(Color online) CLCA cities.** (a) The population map of the cluster of regions. (b) Cities defined by the CLCA.

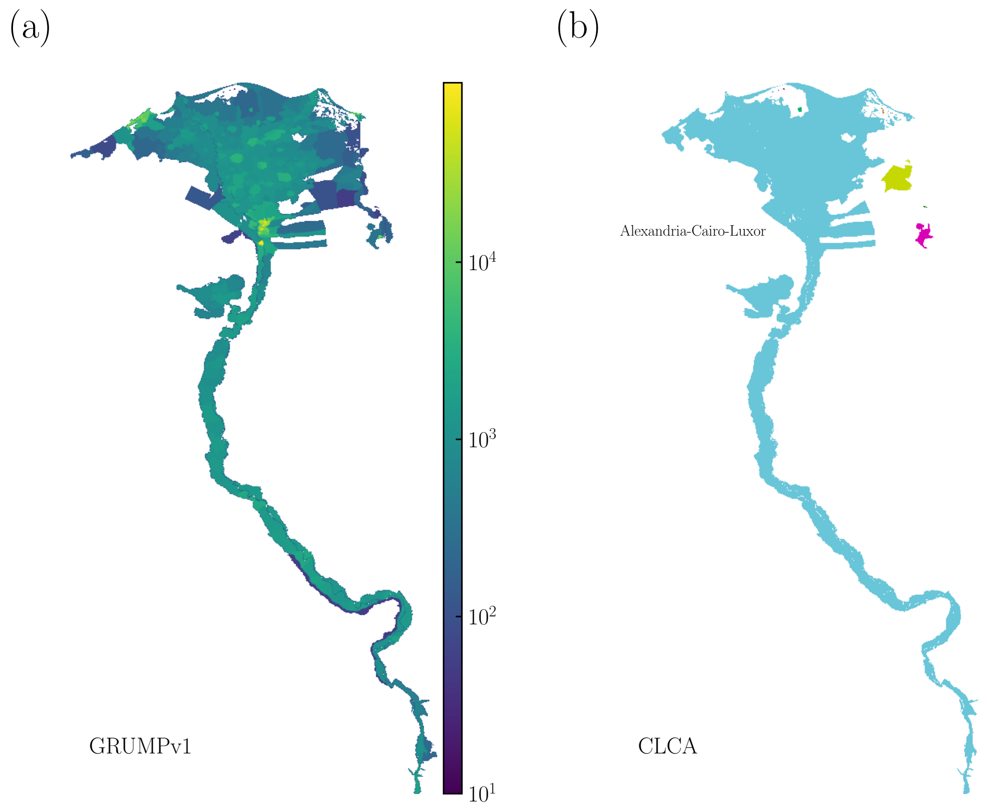

FIG. 7. **(Color online) CLCA cities.** (a) The population map of the cluster of regions. (b) Cities defined by the CLCA.

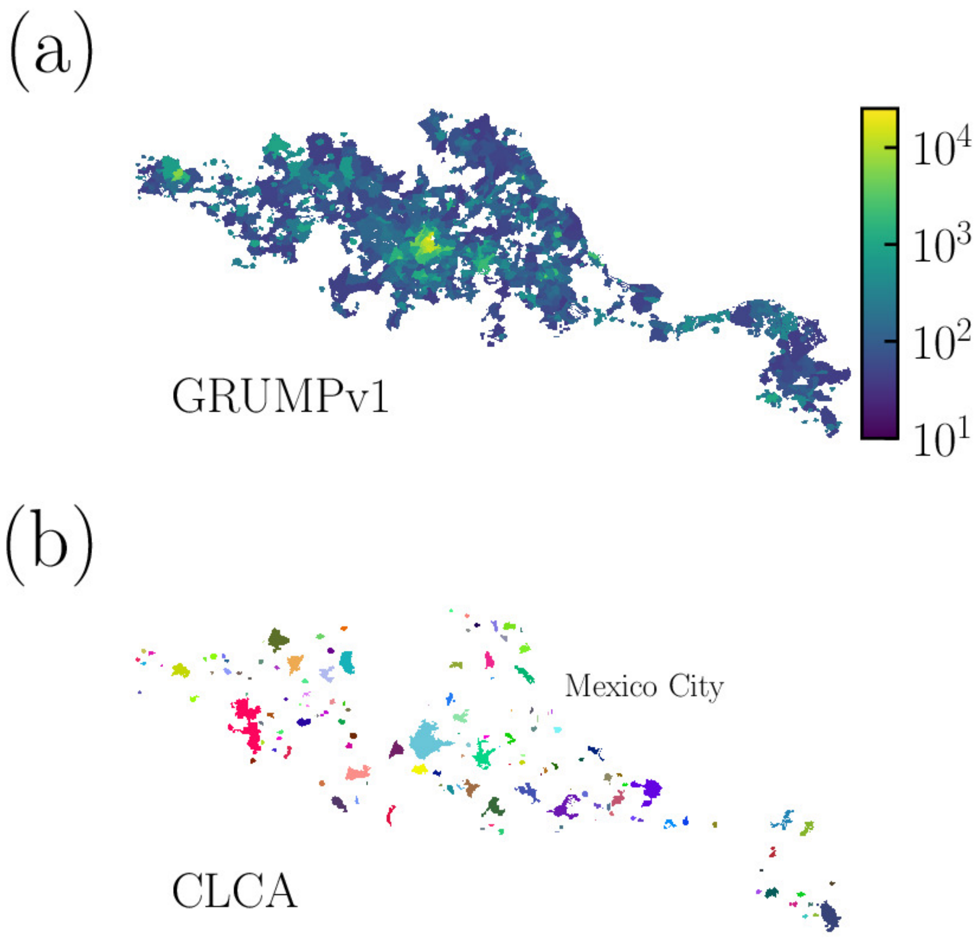

FIG. 8. **(Color online) CLCA cities.** (a) The population map of the cluster of regions. (b) Cities defined by the CLCA.

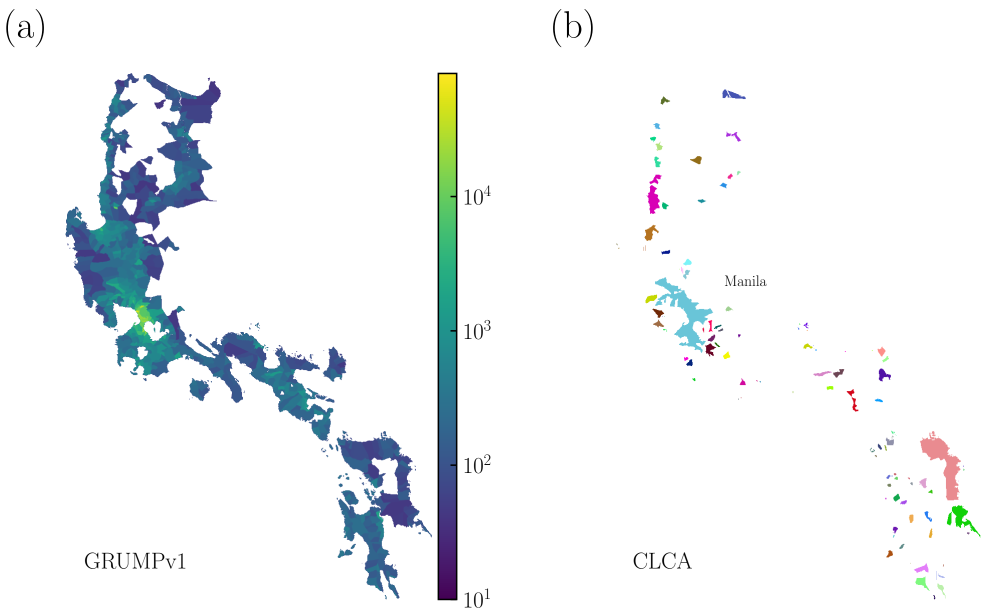

FIG. 9. **(Color online) CLCA cities.** (a) The population map of the cluster of regions. (b) Cities defined by the CLCA.

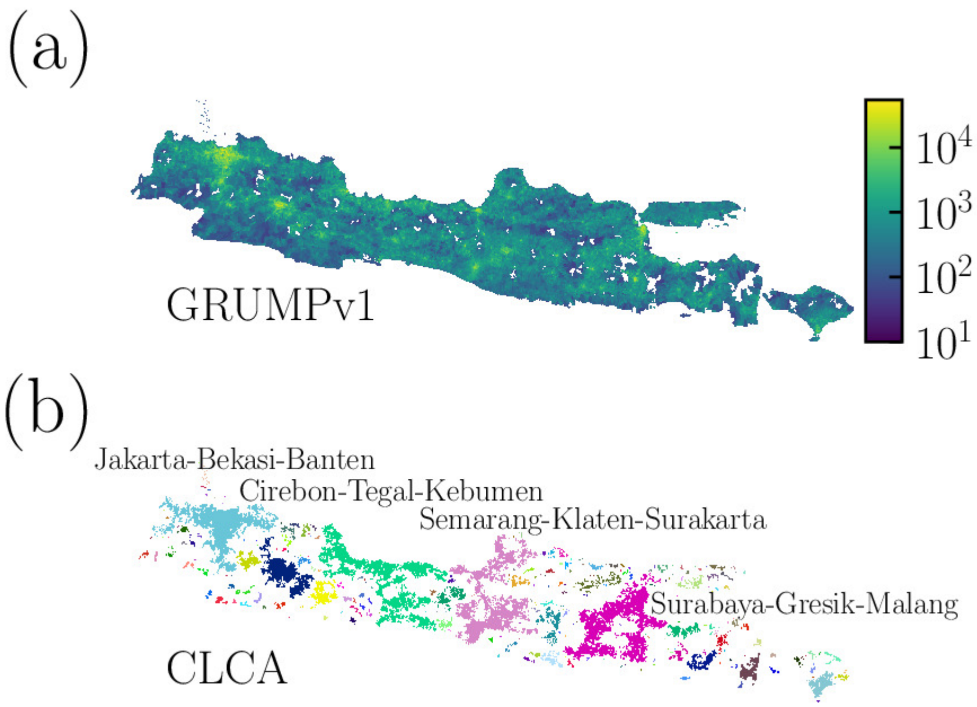

FIG. 10. **(Color online) CLCA cities.** (a) The population map of the cluster of regions. (b) Cities defined by the CLCA.

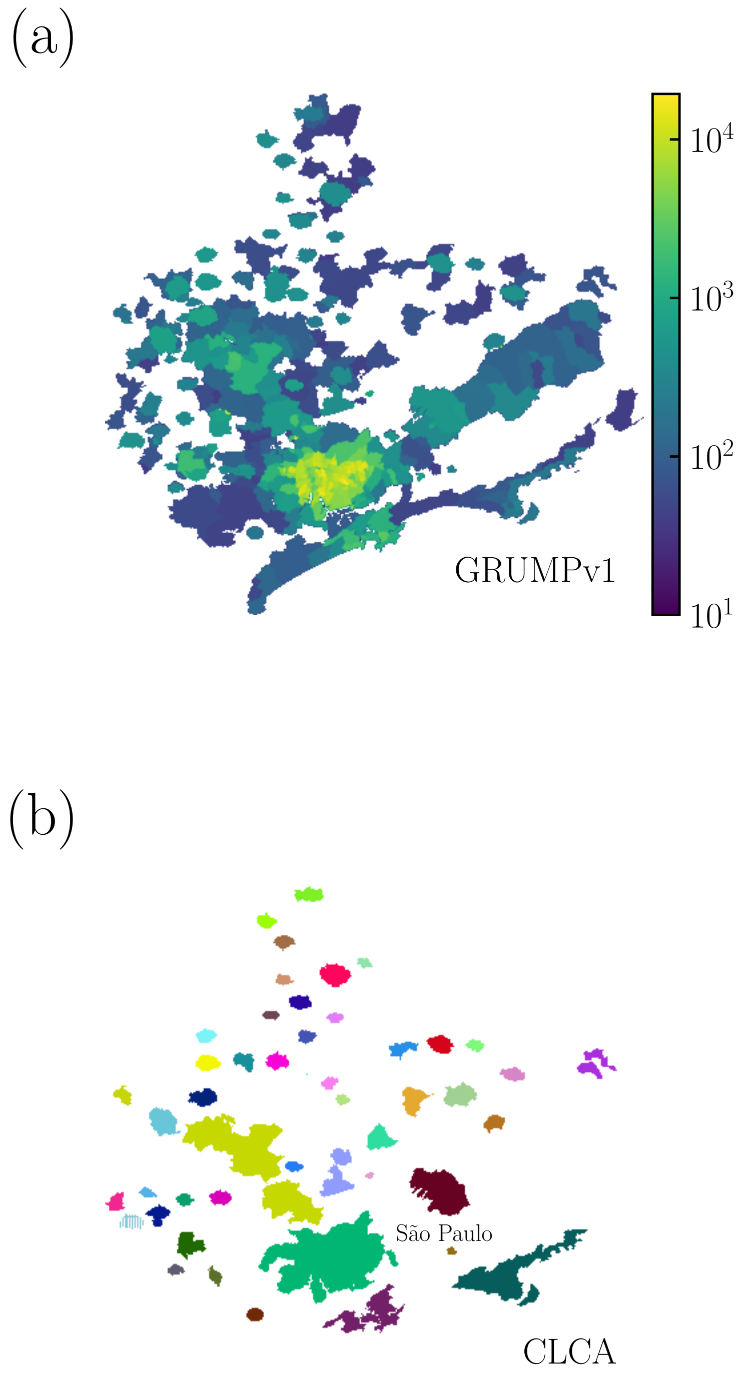

FIG. 11. **(Color online) CLCA cities.** (a) The population map of the cluster of regions. (b) Cities defined by the CLCA.

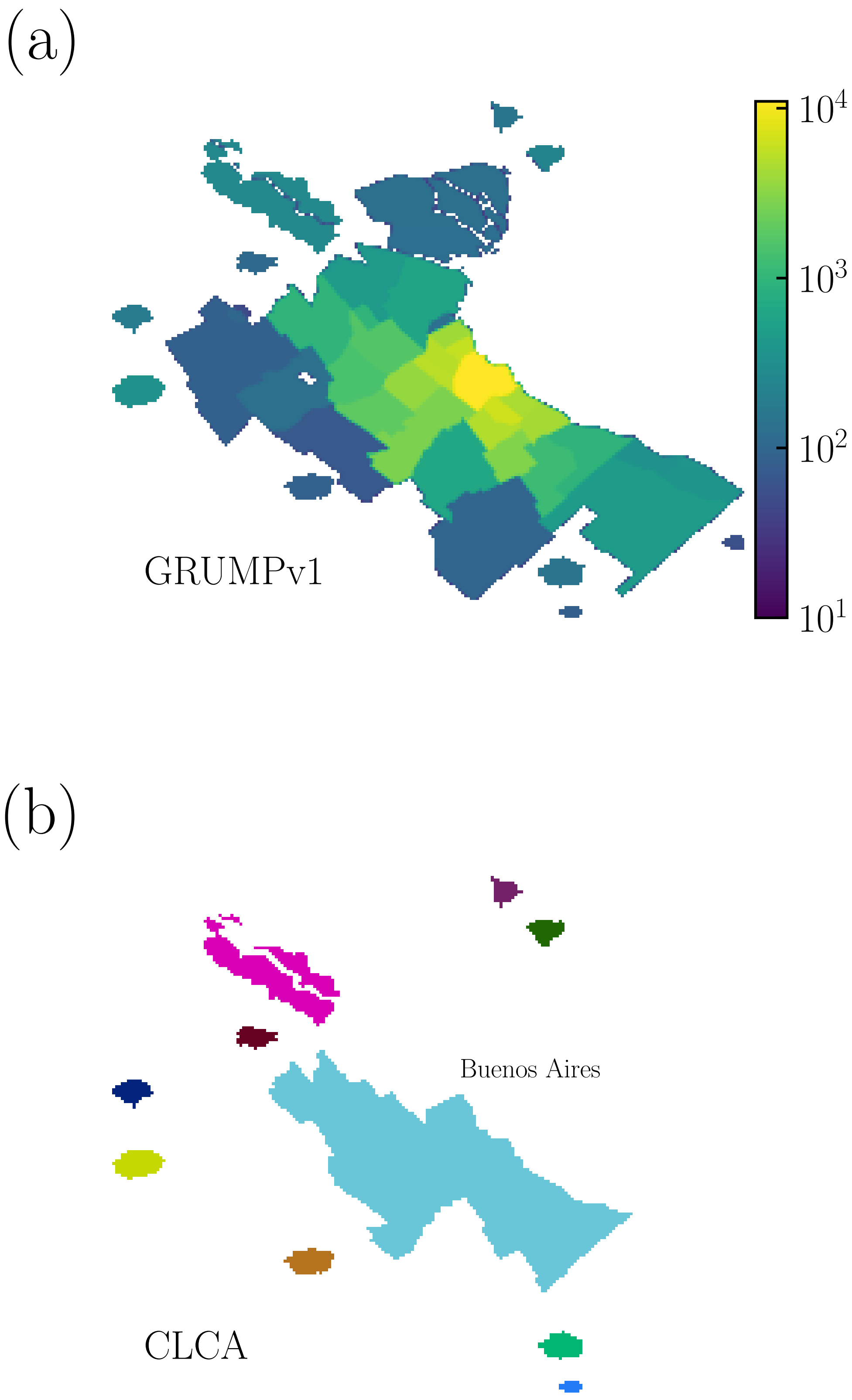

FIG. 12. **(Color online) CLCA cities.** (a) The population map of the cluster of regions. (b) Cities defined by the CLCA.

II. ISO 3166-1 ALPHA-3 CODES

Continent	Country	ISO
Africa	Algeria	DZA
Africa	Benin	BEN
Africa	Burkina Faso	BFA
Africa	Cameroon	CMR
Africa	Central African Republic	CAF
Africa	Democratic Republic of the Congo	COD
Africa	Ethiopia	ETH
Africa	Gabon	GAB
Africa	Ghana	GHA
Africa	Guinea	GIN
Africa	Ivory Coast	CIV
Africa	Kenya	KEN
Africa	Madagascar	MDG
Africa	Malawi	MWI
Africa	Mali	MLI
Africa	Mozambique	MOZ
Africa	Nigeria	NGA
Africa	Niger	NER
Africa	Senegal	SEN
Africa	South Africa	ZAF
Africa	Sudan	SDN
Africa	Togo	TGO
Africa	Tunisia	TUN
Africa	Uganda	UGA
Africa	United Republic of Tanzania	TZA
Africa	Zambia	ZMB
Africa	Zimbabwe	ZWE
Asia	Afghanistan	AFG

Asia	Armenia	ARM
Asia	Azerbaijan	AZE
Asia	Bangladesh	BGD
Asia	Cambodia	KHM
Asia	China	CHN
Asia	Democratic Peoples Republic of Korea	PRK
Asia	Georgia	GEO
Asia	India	IND
Asia	Indonesia	IDN
Asia	Iran	IRN
Asia	Israel	ISR
Asia	Japan	JPN
Asia	Kazakhstan	KAZ
Asia	Korea	KOR
Asia	Kyrgyz Republic	KGZ
Asia	Lao Peoples Democratic Republic	LAO
Asia	Malaysia	MYS
Asia	Myanmar	MMR
Asia	Nepal	NPL
Asia	Pakistan	PAK
Asia	Philippines	PHL
Asia	Syrian Arab Republic	SYR
Asia	Thailand	THA
Asia	Turkey	TUR
Asia	Uzbekistan	UZB
Asia	Viet Nam	VNM
Asia	Yemen	YEM
Europe	Albania	ALB
Europe	Austria	AUT
Europe	Belgium	BEL

Europe	Bosnia-Herzegovina	BIH
Europe	Bulgaria	BGR
Europe	Croatia	HRV
Europe	Czech Republic	CZE
Europe	Denmark	DNK
Europe	Finland	FIN
Europe	France	FRA
Europe	Germany	DEU
Europe	Greece	GRC
Europe	Hungary	HUN
Europe	Ireland	IRL
Europe	Italy	ITA
Europe	Latvia	LVA
Europe	Lithuania	LTU
Europe	Macedonia	MKD
Europe	Netherlands	NLD
Europe	Norway	NOR
Europe	Poland	POL
Europe	Portugal	PRT
Europe	Republic of Moldova	MDA
Europe	Romania	ROU
Europe	Russia	RUS
Europe	Serbia and Montenegro	SCG
Europe	Slovakia	SVK
Europe	Slovenia	SVN
Europe	Spain	ESP
Europe	Sweden	SWE
Europe	Ukraine	UKR
Europe	United Kingdom	GBR
N. America	Canada	CAN

N. America	Cuba	CUB
N. America	Honduras	HND
N. America	Mexico	MEX
N. America	Nicaragua	NIC
N. America	United States	USA
Oceania	Australia	AUS
Oceania	Marshall Islands	MHL
Oceania	New Zealand	NZL
S. America	Argentina	ARG
S. America	Bolivia	BOL
S. America	Brazil	BRA
S. America	Chile	CHL
S. America	Colombia	COL
S. America	Ecuador	ECU
S. America	Paraguay	PRY
S. America	Peru	PER
S. America	Uruguay	URY

III. CDF FOR ALL COUNTRIES

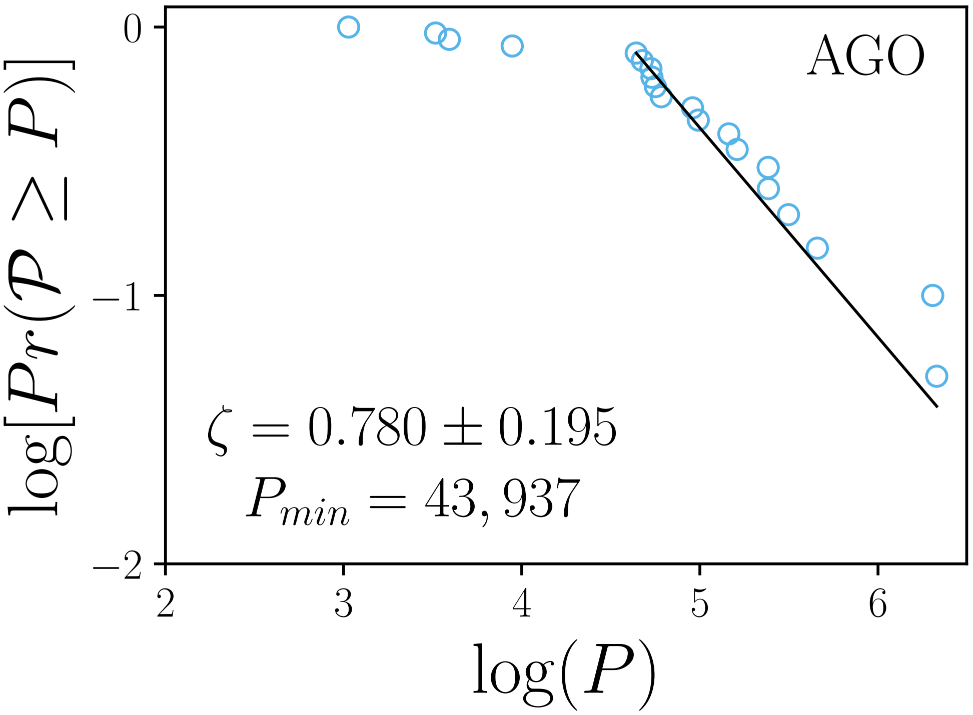

FIG. 13. **(Color online) Cumulative Distribution Function (CDF)** $Pr(\mathcal{P} \geq P)$ **versus population** P **in log-log scale.** Cities proposed by the City Local Clustering Algorithm (CLCA) are represented by light blue circles. The solid black line is the maximum likelihood power-law fit defined by the Maximum Likelihood Estimator (MLE) [50]. The value of the lower bound P_{min} and the exponent ζ are also shown. The CLCA parameters used were $D^{(min)} = 100$ people$/km^2$, $D^{(max)} = 1000$ people$/km^2$, $\delta = 10$ people$/km^2$, $\ell = 3$ km, $A^* = 50$ km^2 and $H^* = 0.50$.

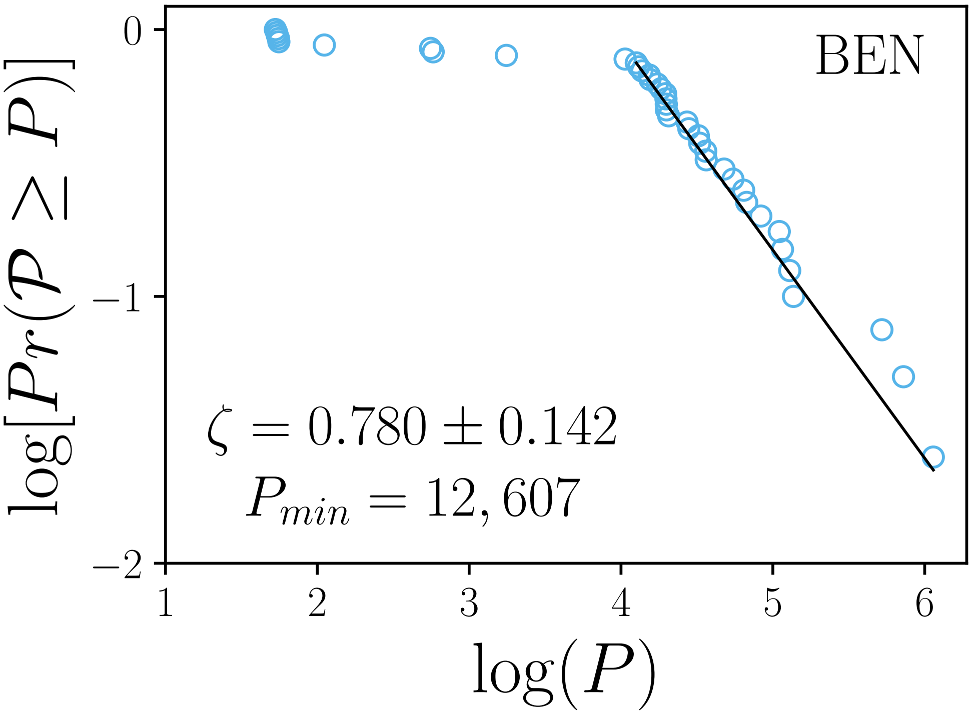

FIG. 14. **(Color online) Cumulative Distribution Function (CDF)** $Pr(\mathcal{P} \geq P)$ **versus population** P **in log-log scale.** Cities proposed by the City Local Clustering Algorithm (CLCA) are represented by light blue circles. The solid black line is the maximum likelihood power-law fit defined by the Maximum Likelihood Estimator (MLE) [50]. The value of the lower bound P_{min} and the exponent ζ are also shown. The CLCA parameters used were $D^{(min)} = 100$ people/km^2, $D^{(max)} = 1000$ people/km^2, $\delta = 10$ people/km^2, $\ell = 3$ km, $A^* = 50$ km^2 and $H^* = 0.50$.

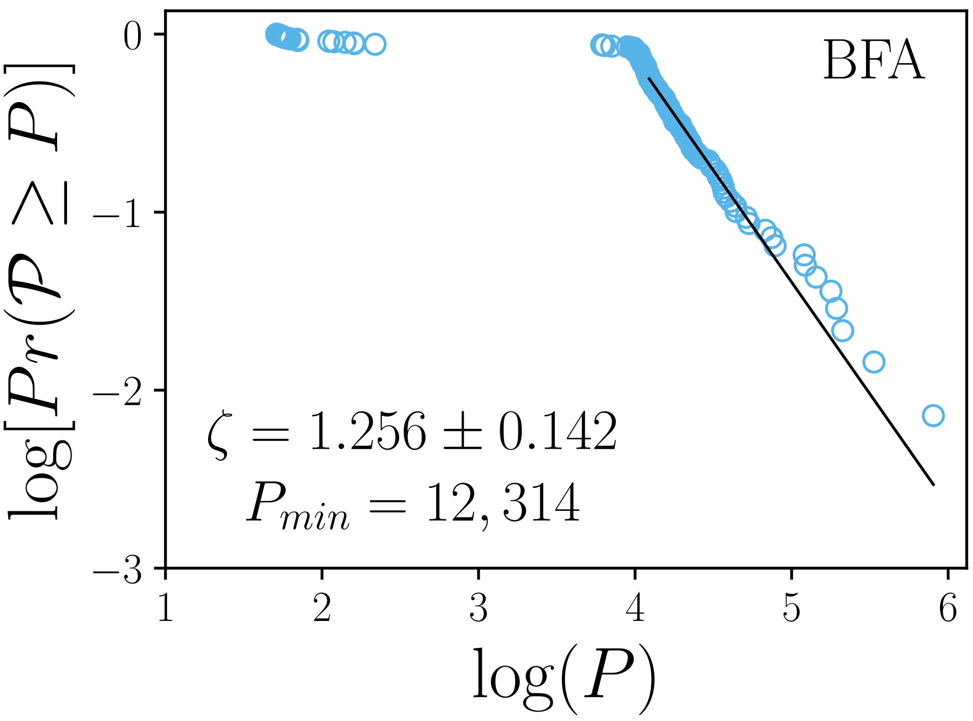

FIG. 15. **(Color online) Cumulative Distribution Function (CDF)** $Pr(\mathcal{P} \geq P)$ **versus population** P **in log-log scale.** Cities proposed by the City Local Clustering Algorithm (CLCA) are represented by light blue circles. The solid black line is the maximum likelihood power-law fit defined by the Maximum Likelihood Estimator (MLE) [50]. The value of the lower bound P_{min} and the exponent ζ are also shown. The CLCA parameters used were $D^{(min)} = 100$ people/km^2, $D^{(max)} = 1000$ people/km^2, $\delta = 10$ people/km^2, $\ell = 3$ km, $A^* = 50$ km^2 and $H^* = 0.50$.

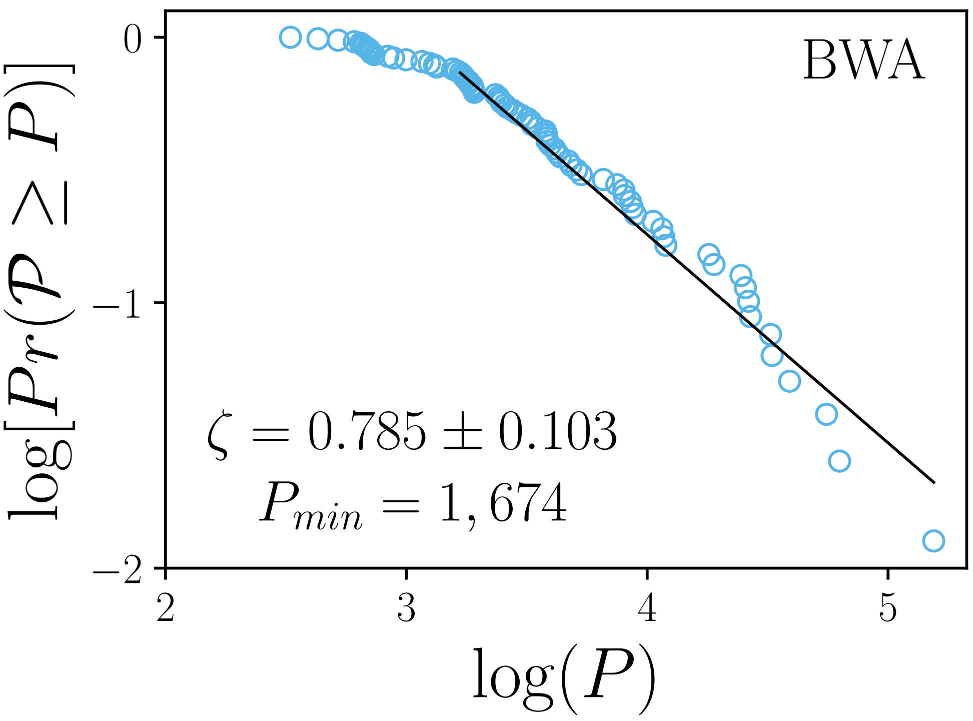

FIG. 16. **(Color online) Cumulative Distribution Function (CDF)** $Pr(\mathcal{P} \geq P)$ **versus population** P **in log-log scale.** Cities proposed by the City Local Clustering Algorithm (CLCA) are represented by light blue circles. The solid black line is the maximum likelihood power-law fit defined by the Maximum Likelihood Estimator (MLE) [50]. The value of the lower bound P_{min} and the exponent ζ are also shown. The CLCA parameters used were $D^{(min)} = 100$ people/km^2, $D^{(max)} = 1000$ people/km^2, $\delta = 10$ people/km^2, $\ell = 3$ km, $A^* = 50$ km^2 and $H^* = 0.50$.

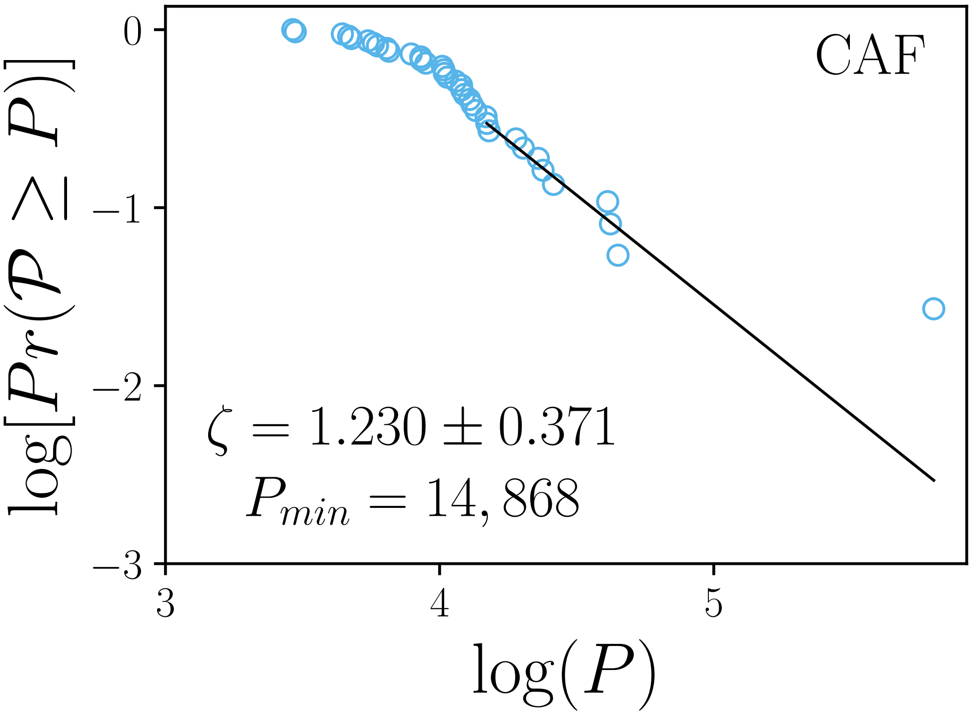

FIG. 17. **(Color online) Cumulative Distribution Function (CDF) $Pr(\mathcal{P} \geq P)$ versus population P in log-log scale.** Cities proposed by the City Local Clustering Algorithm (CLCA) are represented by light blue circles. The solid black line is the maximum likelihood power-law fit defined by the Maximum Likelihood Estimator (MLE) [50]. The value of the lower bound P_{min} and the exponent ζ are also shown. The CLCA parameters used were $D^{(min)} = 100$ people/km^2, $D^{(max)} = 1000$ people/km^2, $\delta = 10$ people/km^2, $\ell = 3$ km, $A^* = 50$ km^2 and $H^* = 0.50$.

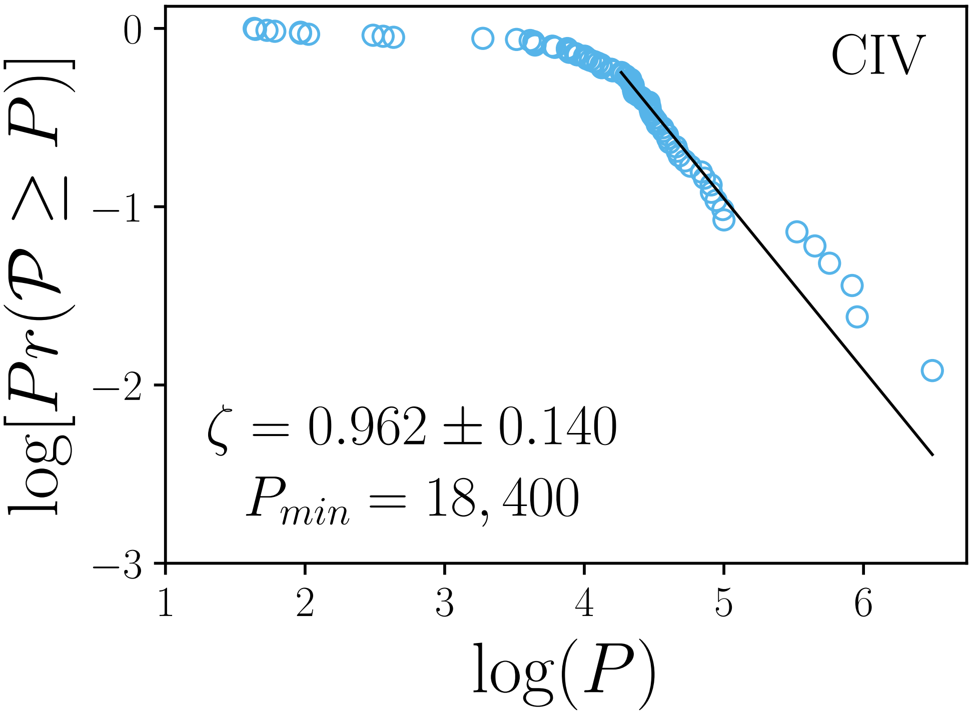

FIG. 18. **(Color online) Cumulative Distribution Function (CDF)** $Pr(\mathcal{P} \geq P)$ **versus population** P **in log-log scale.** Cities proposed by the City Local Clustering Algorithm (CLCA) are represented by light blue circles. The solid black line is the maximum likelihood power-law fit defined by the Maximum Likelihood Estimator (MLE) [50]. The value of the lower bound P_{min} and the exponent ζ are also shown. The CLCA parameters used were $D^{(min)} = 100$ people/km^2, $D^{(max)} = 1000$ people/km^2, $\delta = 10$ people/km^2, $\ell = 3$ km, $A^* = 50$ km^2 and $H^* = 0.50$.

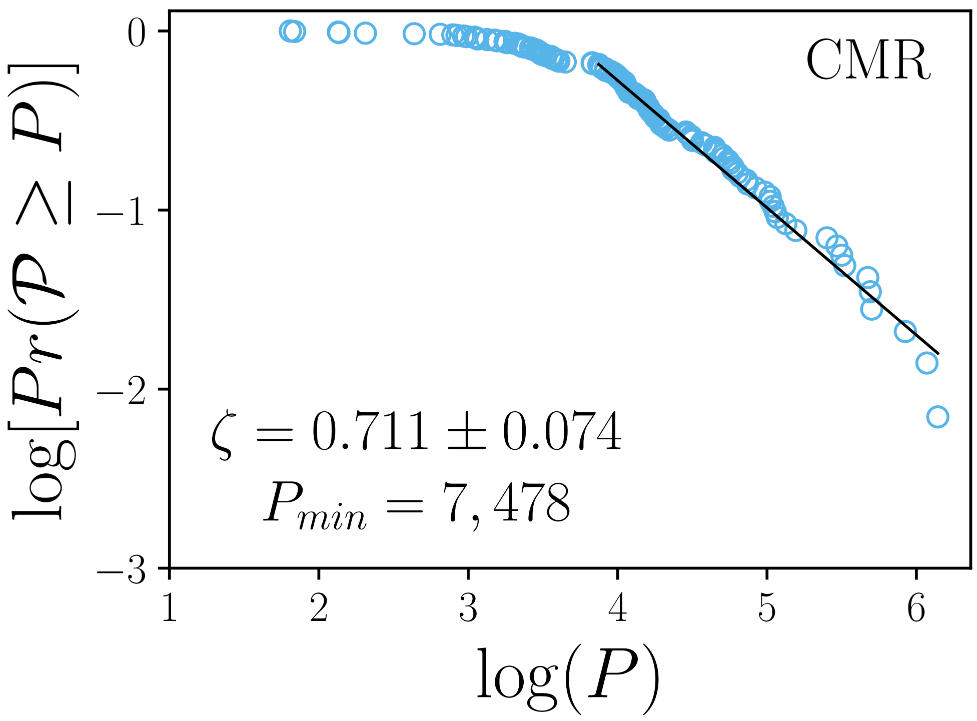

FIG. 19. **(Color online) Cumulative Distribution Function (CDF)** $Pr(\mathcal{P} \geq P)$ **versus population** P **in log-log scale.** Cities proposed by the City Local Clustering Algorithm (CLCA) are represented by light blue circles. The solid black line is the maximum likelihood power-law fit defined by the Maximum Likelihood Estimator (MLE) [50]. The value of the lower bound P_{min} and the exponent ζ are also shown. The CLCA parameters used were $D^{(min)} = 100$ people/km^2, $D^{(max)} = 1000$ people/km^2, $\delta = 10$ people/km^2, $\ell = 3$ km, $A^* = 50$ km^2 and $H^* = 0.50$.

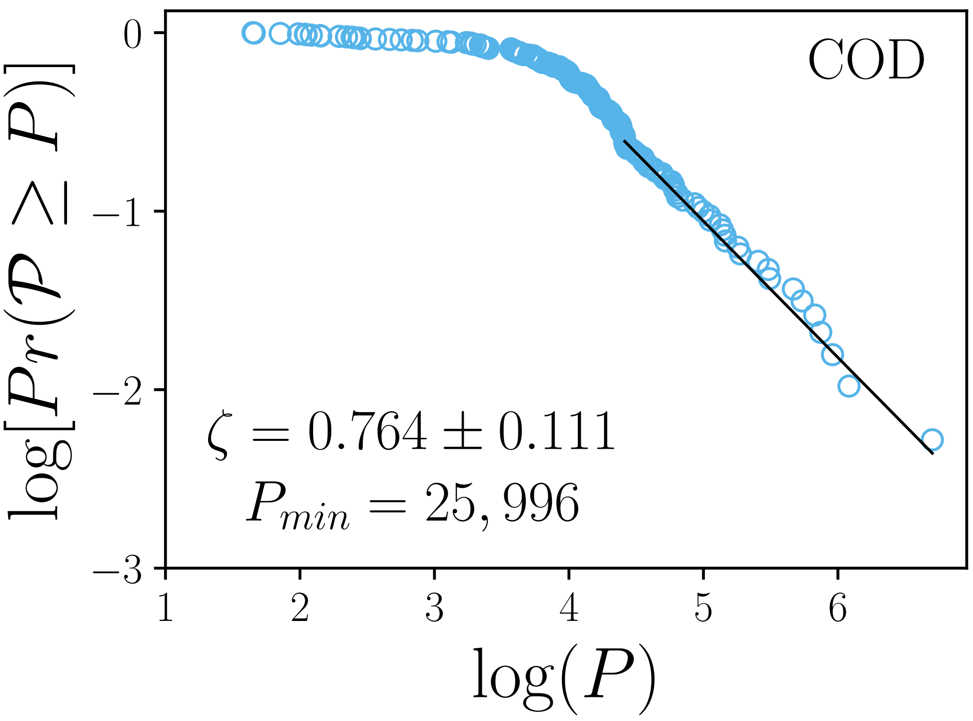

FIG. 20. **(Color online) Cumulative Distribution Function (CDF)** $Pr(\mathcal{P} \geq P)$ **versus population** P **in log-log scale.** Cities proposed by the City Local Clustering Algorithm (CLCA) are represented by light blue circles. The solid black line is the maximum likelihood power-law fit defined by the Maximum Likelihood Estimator (MLE) [50]. The value of the lower bound P_{min} and the exponent ζ are also shown. The CLCA parameters used were $D^{(min)} = 100$ people$/km^2$, $D^{(max)} = 1000$ people$/km^2$, $\delta = 10$ people$/km^2$, $\ell = 3$ km, $A^* = 50$ km^2 and $H^* = 0.50$.

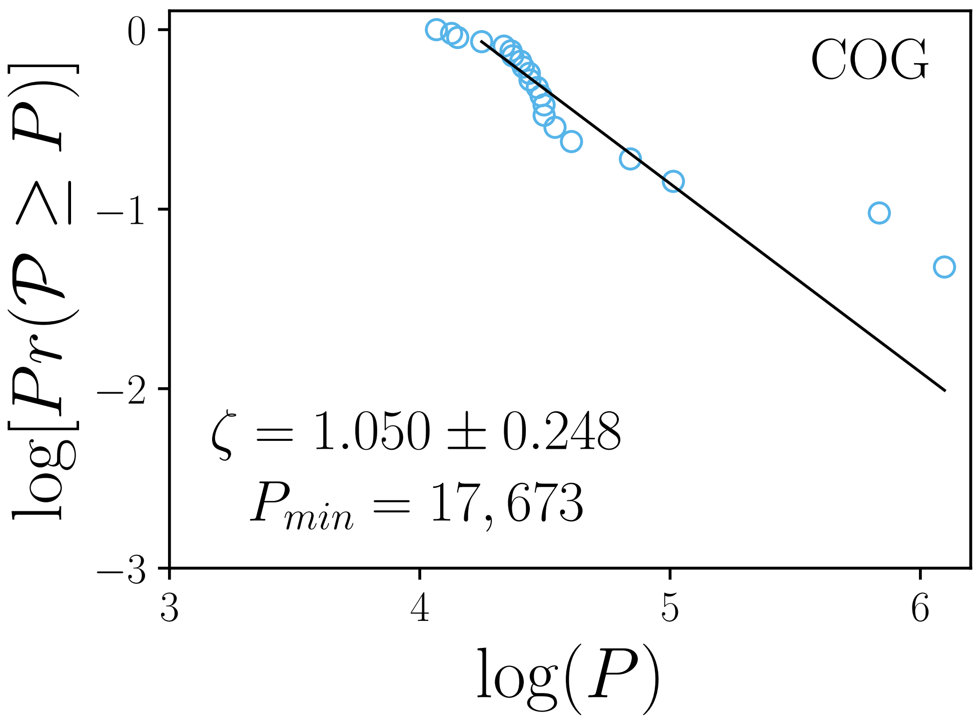

FIG. 21. **(Color online) Cumulative Distribution Function (CDF)** $Pr(\mathcal{P} \geq P)$ **versus population** P **in log-log scale.** Cities proposed by the City Local Clustering Algorithm (CLCA) are represented by light blue circles. The solid black line is the maximum likelihood power-law fit defined by the Maximum Likelihood Estimator (MLE) [50]. The value of the lower bound P_{min} and the exponent ζ are also shown. The CLCA parameters used were $D^{(min)} = 100$ people$/km^2$, $D^{(max)} = 1000$ people$/km^2$, $\delta = 10$ people$/km^2$, $\ell = 3\ km$, $A^* = 50\ km^2$ and $H^* = 0.50$.

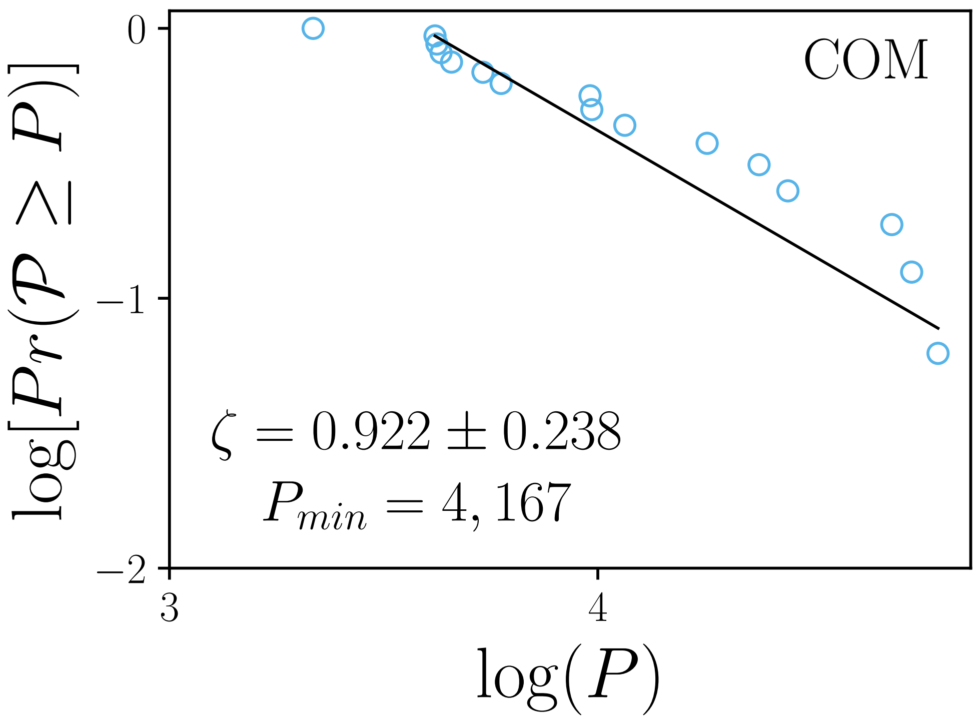

FIG. 22. **(Color online) Cumulative Distribution Function (CDF)** $Pr(\mathcal{P} \geq P)$ **versus population** P **in log-log scale.** Cities proposed by the City Local Clustering Algorithm (CLCA) are represented by light blue circles. The solid black line is the maximum likelihood power-law fit defined by the Maximum Likelihood Estimator (MLE) [50]. The value of the lower bound P_{min} and the exponent ζ are also shown. The CLCA parameters used were $D^{(min)} = 100$ people/km^2, $D^{(max)} = 1000$ people/km^2, $\delta = 10$ people/km^2, $\ell = 3$ km, $A^* = 50$ km^2 and $H^* = 0.50$.

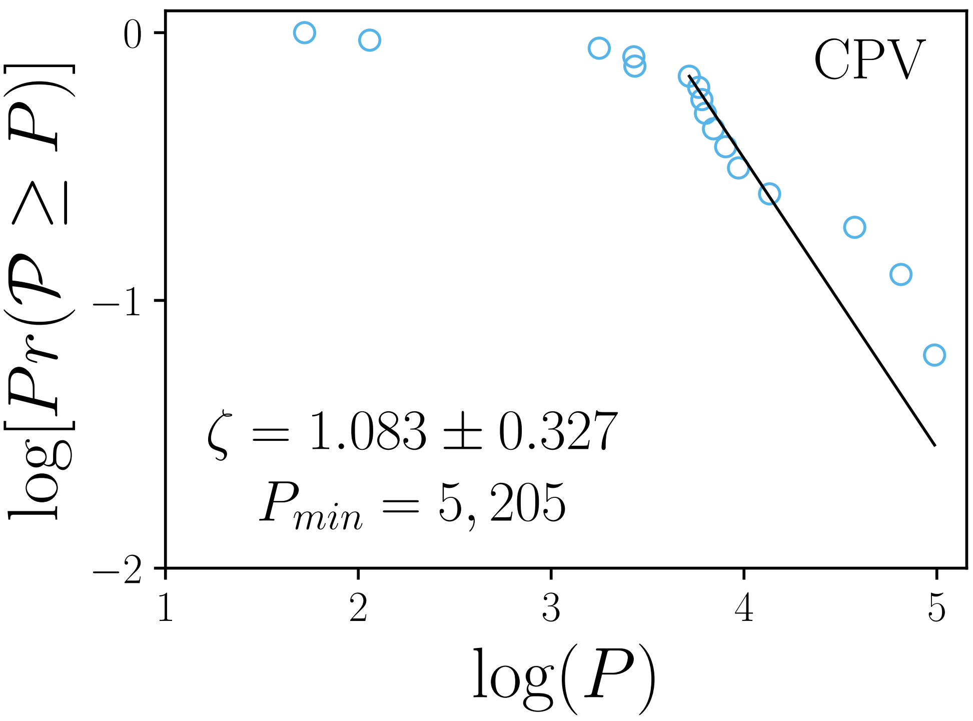

FIG. 23. **(Color online) Cumulative Distribution Function (CDF)** $Pr(\mathcal{P} \geq P)$ **versus population** P **in log-log scale.** Cities proposed by the City Local Clustering Algorithm (CLCA) are represented by light blue circles. The solid black line is the maximum likelihood power-law fit defined by the Maximum Likelihood Estimator (MLE) [50]. The value of the lower bound P_{min} and the exponent ζ are also shown. The CLCA parameters used were $D^{(min)} = 100$ people/km^2, $D^{(max)} = 1000$ people/km^2, $\delta = 10$ people/km^2, $\ell = 3$ km, $A^* = 50$ km^2 and $H^* = 0.50$.

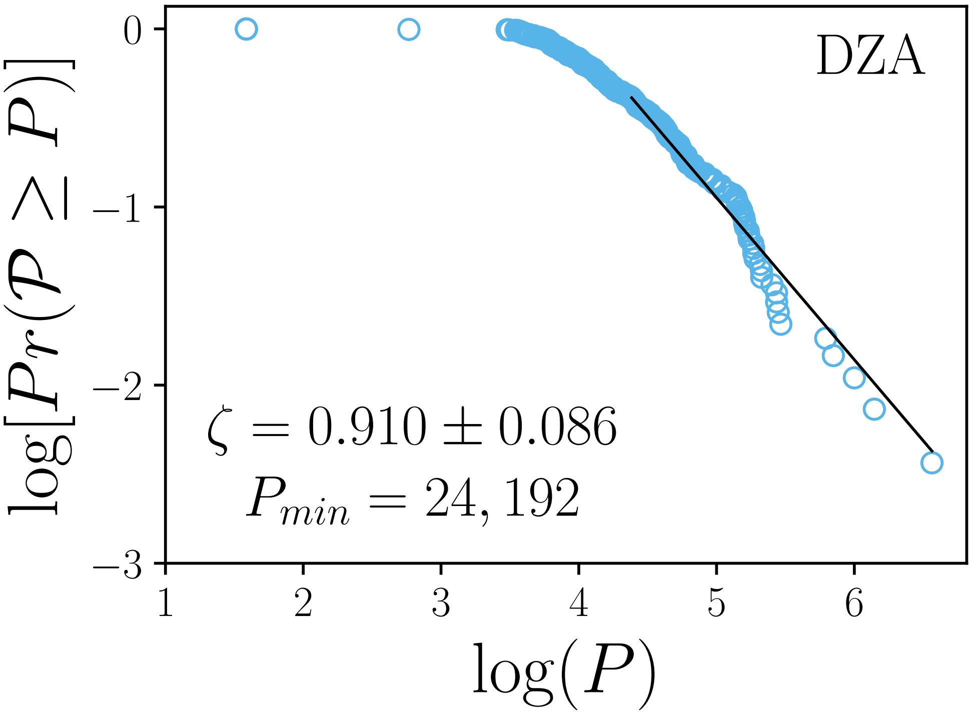

FIG. 24. **(Color online) Cumulative Distribution Function (CDF)** $Pr(\mathcal{P} \geq P)$ **versus population** P **in log-log scale.** Cities proposed by the City Local Clustering Algorithm (CLCA) are represented by light blue circles. The solid black line is the maximum likelihood power-law fit defined by the Maximum Likelihood Estimator (MLE) [50]. The value of the lower bound P_{min} and the exponent ζ are also shown. The CLCA parameters used were $D^{(min)} = 100$ people$/km^2$, $D^{(max)} = 1000$ people$/km^2$, $\delta = 10$ people$/km^2$, $\ell = 3\ km$, $A^* = 50\ km^2$ and $H^* = 0.50$.

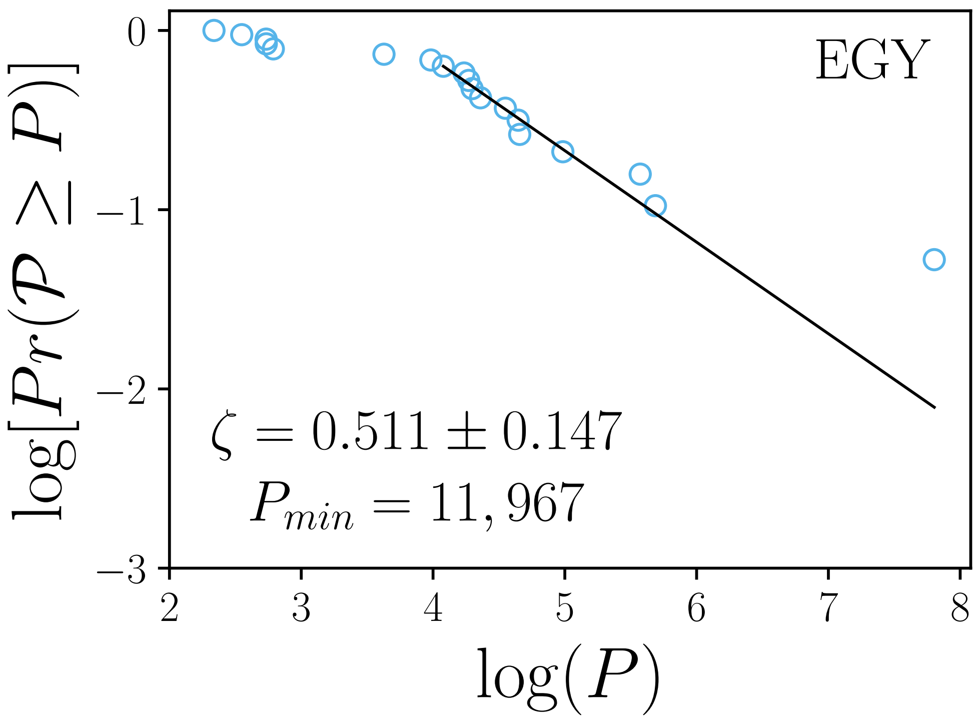

FIG. 25. **(Color online) Cumulative Distribution Function (CDF)** $Pr(\mathcal{P} \geq P)$ **versus population** P **in log-log scale.** Cities proposed by the City Local Clustering Algorithm (CLCA) are represented by light blue circles. The solid black line is the maximum likelihood power-law fit defined by the Maximum Likelihood Estimator (MLE) [50]. The value of the lower bound P_{min} and the exponent ζ are also shown. The CLCA parameters used were $D^{(min)} = 100$ people/km^2, $D^{(max)} = 1000$ people/km^2, $\delta = 10$ people/km^2, $\ell = 3$ km, $A^* = 50$ km^2 and $H^* = 0.50$.

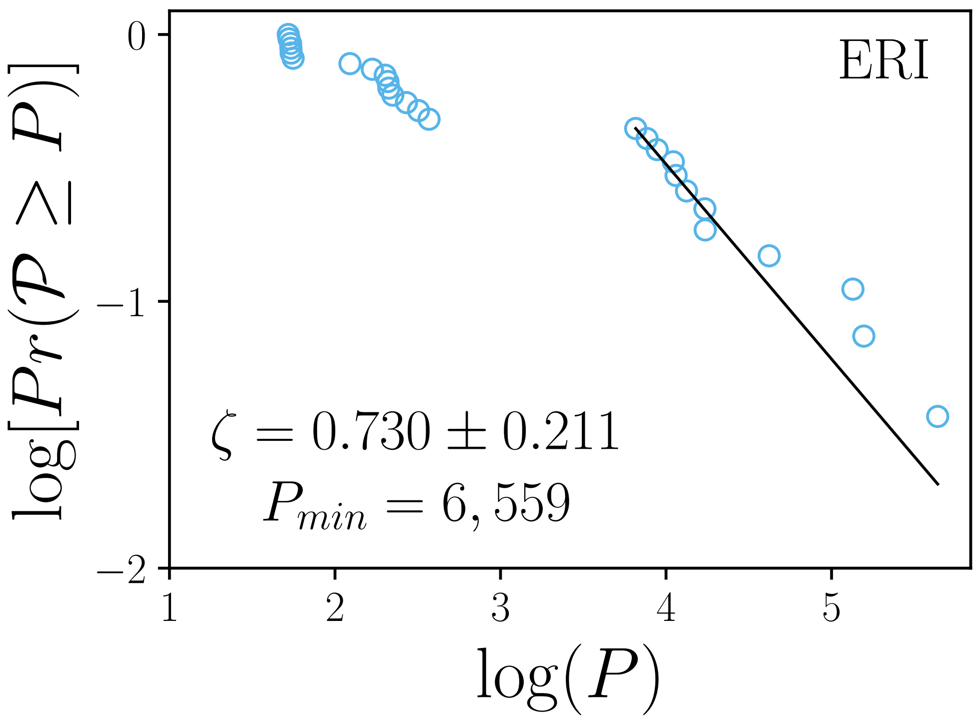

FIG. 26. **(Color online) Cumulative Distribution Function (CDF)** $Pr(\mathcal{P} \geq P)$ **versus population** P **in log-log scale.** Cities proposed by the City Local Clustering Algorithm (CLCA) are represented by light blue circles. The solid black line is the maximum likelihood power-law fit defined by the Maximum Likelihood Estimator (MLE) [50]. The value of the lower bound P_{min} and the exponent ζ are also shown. The CLCA parameters used were $D^{(min)} = 100$ people/km^2, $D^{(max)} = 1000$ people/km^2, $\delta = 10$ people/km^2, $\ell = 3$ km, $A^* = 50$ km^2 and $H^* = 0.50$.

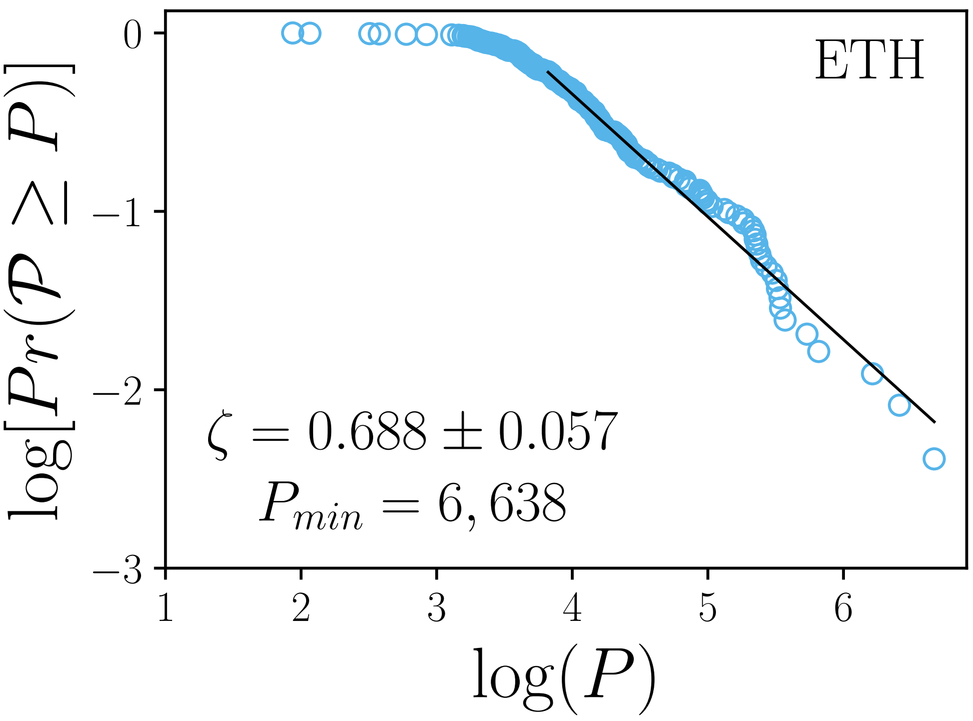

FIG. 27. **(Color online) Cumulative Distribution Function (CDF)** $Pr(\mathcal{P} \geq P)$ **versus population** P **in log-log scale.** Cities proposed by the City Local Clustering Algorithm (CLCA) are represented by light blue circles. The solid black line is the maximum likelihood power-law fit defined by the Maximum Likelihood Estimator (MLE) [50]. The value of the lower bound P_{min} and the exponent ζ are also shown. The CLCA parameters used were $D^{(min)} = 100$ people$/km^2$, $D^{(max)} = 1000$ people$/km^2$, $\delta = 10$ people$/km^2$, $\ell = 3$ km, $A^* = 50$ km^2 and $H^* = 0.50$.

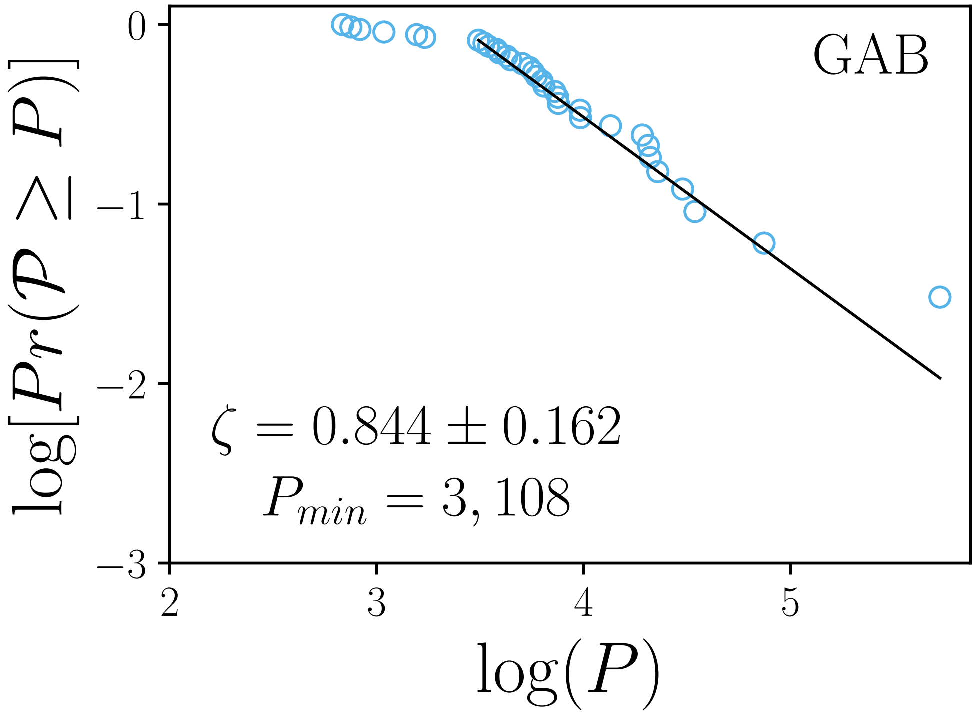

FIG. 28. **(Color online) Cumulative Distribution Function (CDF)** $Pr(\mathcal{P} \geq P)$ **versus population** P **in log-log scale.** Cities proposed by the City Local Clustering Algorithm (CLCA) are represented by light blue circles. The solid black line is the maximum likelihood power-law fit defined by the Maximum Likelihood Estimator (MLE) [50]. The value of the lower bound P_{min} and the exponent ζ are also shown. The CLCA parameters used were $D^{(min)} = 100$ people$/km^2$, $D^{(max)} = 1000$ people$/km^2$, $\delta = 10$ people$/km^2$, $\ell = 3\ km$, $A^* = 50\ km^2$ and $H^* = 0.50$.

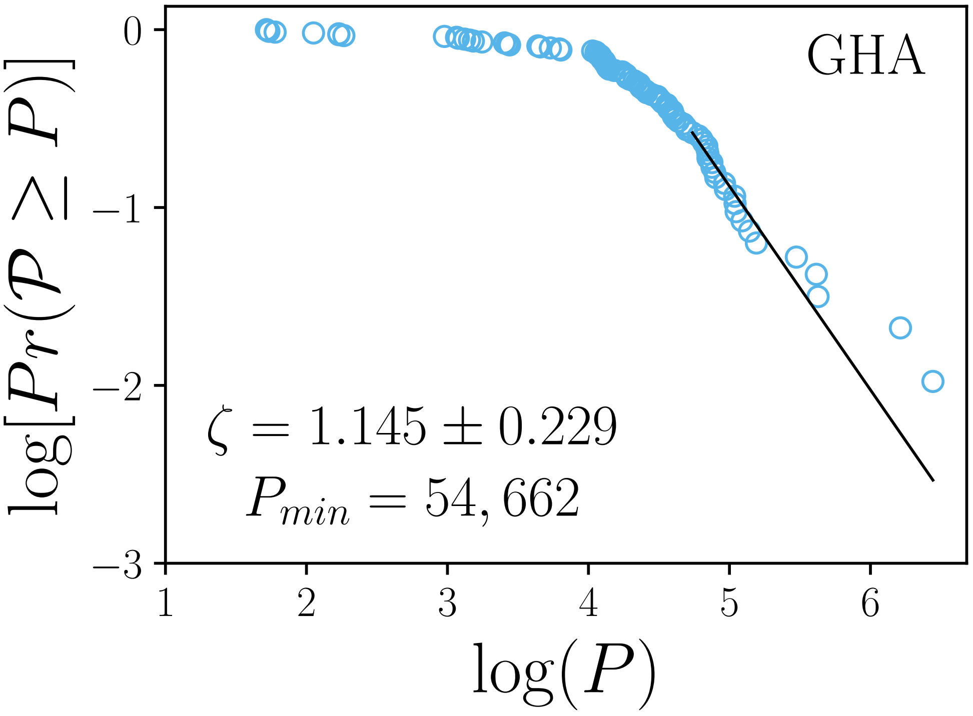

FIG. 29. **(Color online) Cumulative Distribution Function (CDF)** $Pr(\mathcal{P} \geq P)$ **versus population** P **in log-log scale.** Cities proposed by the City Local Clustering Algorithm (CLCA) are represented by light blue circles. The solid black line is the maximum likelihood power-law fit defined by the Maximum Likelihood Estimator (MLE) [50]. The value of the lower bound P_{min} and the exponent ζ are also shown. The CLCA parameters used were $D^{(min)} = 100$ people$/km^2$, $D^{(max)} = 1000$ people$/km^2$, $\delta = 10$ people$/km^2$, $\ell = 3$ km, $A^* = 50$ km^2 and $H^* = 0.50$.

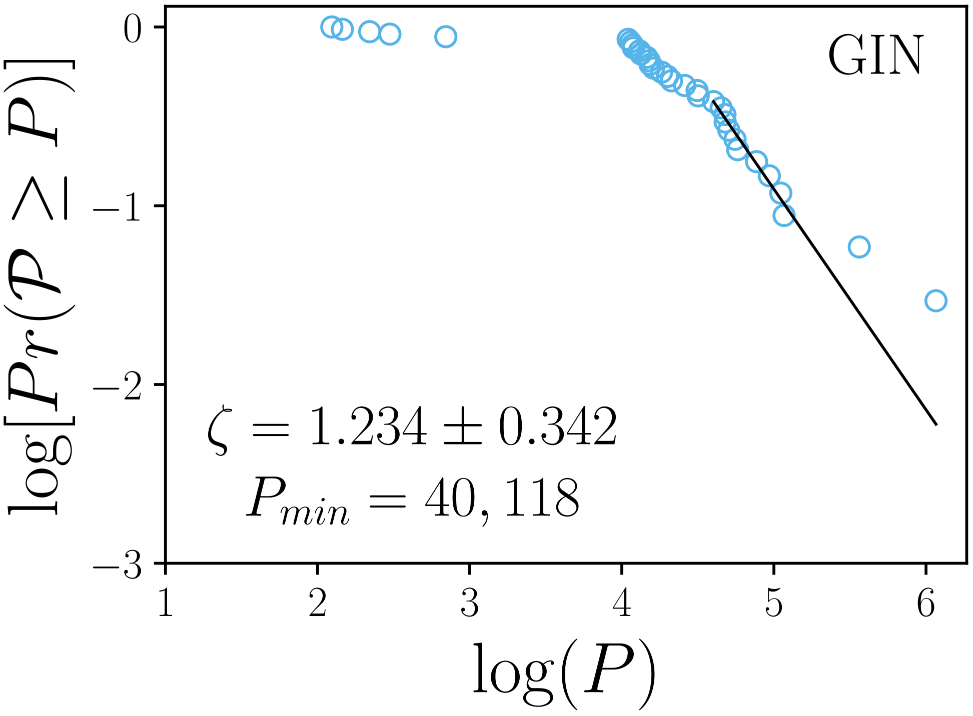

FIG. 30. **(Color online) Cumulative Distribution Function (CDF)** $Pr(\mathcal{P} \geq P)$ **versus population** P **in log-log scale.** Cities proposed by the City Local Clustering Algorithm (CLCA) are represented by light blue circles. The solid black line is the maximum likelihood power-law fit defined by the Maximum Likelihood Estimator (MLE) [50]. The value of the lower bound P_{min} and the exponent ζ are also shown. The CLCA parameters used were $D^{(min)} = 100$ people$/km^2$, $D^{(max)} = 1000$ people$/km^2$, $\delta = 10$ people$/km^2$, $\ell = 3$ km, $A^* = 50$ km^2 and $H^* = 0.50$.

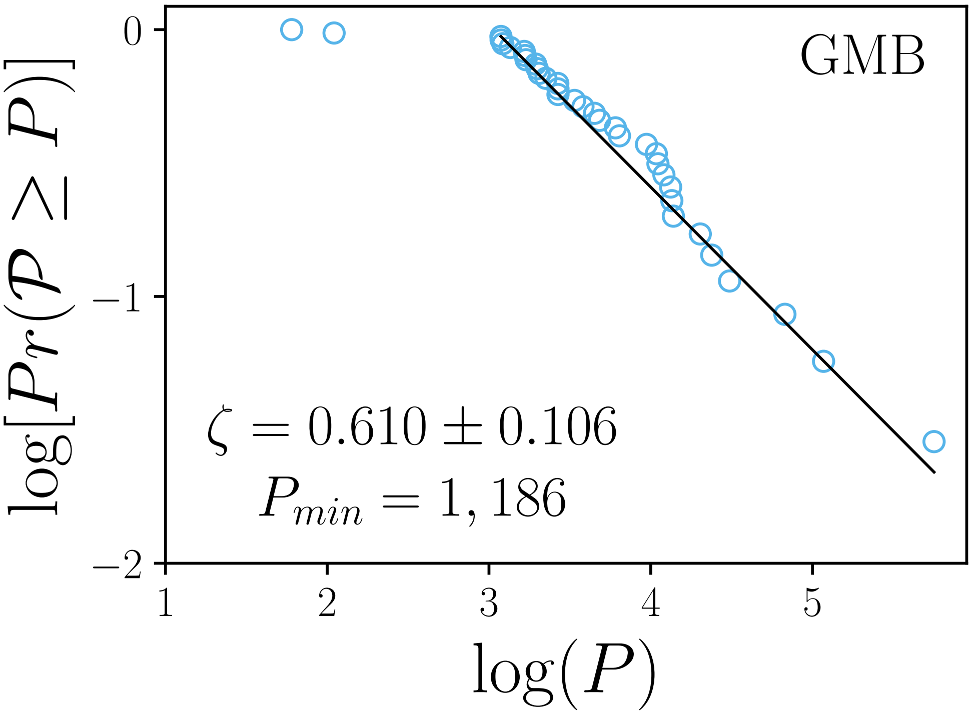

FIG. 31. **(Color online) Cumulative Distribution Function (CDF)** $Pr(\mathcal{P} \geq P)$ **versus population** P **in log-log scale.** Cities proposed by the City Local Clustering Algorithm (CLCA) are represented by light blue circles. The solid black line is the maximum likelihood power-law fit defined by the Maximum Likelihood Estimator (MLE) [50]. The value of the lower bound P_{min} and the exponent ζ are also shown. The CLCA parameters used were $D^{(min)} = 100$ people$/km^2$, $D^{(max)} = 1000$ people$/km^2$, $\delta = 10$ people$/km^2$, $\ell = 3$ km, $A^* = 50$ km^2 and $H^* = 0.50$.

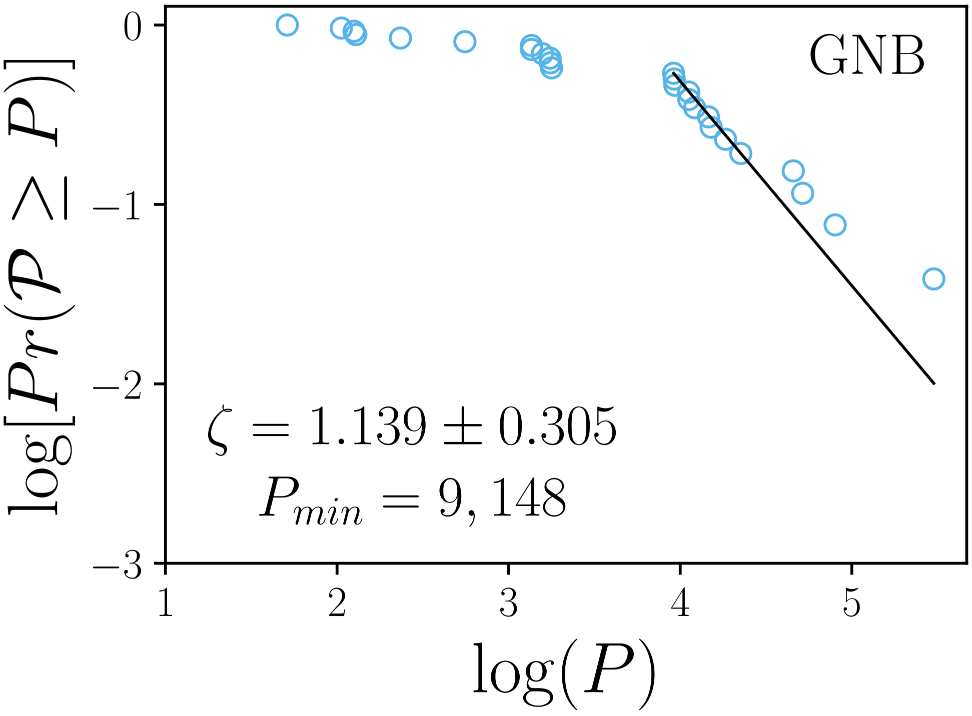

FIG. 32. **(Color online) Cumulative Distribution Function (CDF)** $Pr(\mathcal{P} \geq P)$ **versus population** P **in log-log scale.** Cities proposed by the City Local Clustering Algorithm (CLCA) are represented by light blue circles. The solid black line is the maximum likelihood power-law fit defined by the Maximum Likelihood Estimator (MLE) [50]. The value of the lower bound P_{min} and the exponent ζ are also shown. The CLCA parameters used were $D^{(min)} = 100$ people/km^2, $D^{(max)} = 1000$ people/km^2, $\delta = 10$ people/km^2, $\ell = 3$ km, $A^* = 50$ km^2 and $H^* = 0.50$.

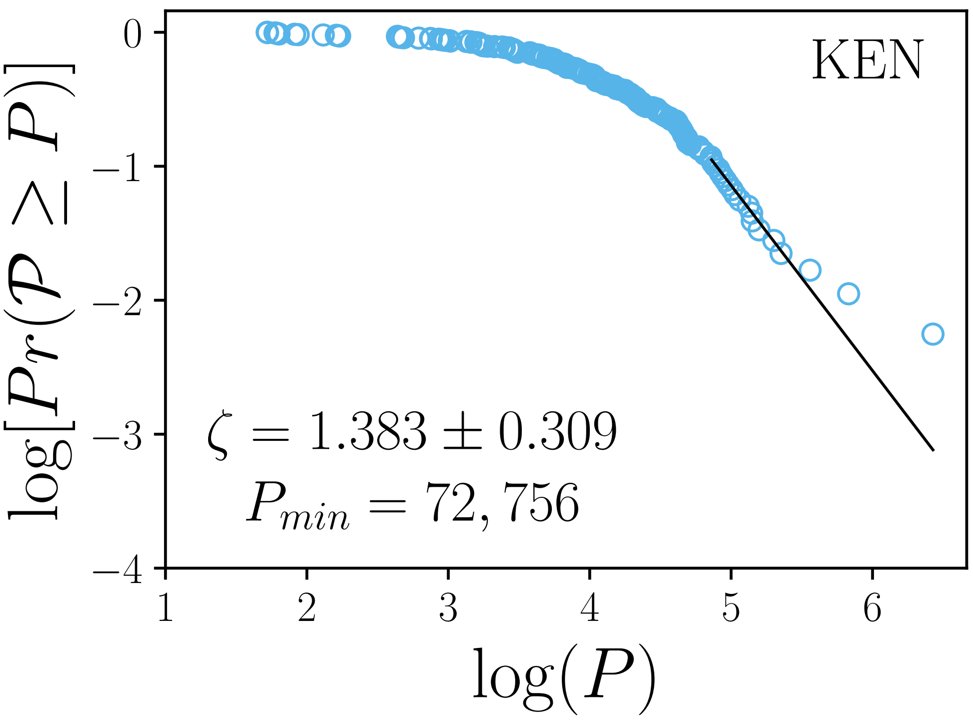

FIG. 33. **(Color online) Cumulative Distribution Function (CDF)** $Pr(\mathcal{P} \geq P)$ **versus population** P **in log-log scale.** Cities proposed by the City Local Clustering Algorithm (CLCA) are represented by light blue circles. The solid black line is the maximum likelihood power-law fit defined by the Maximum Likelihood Estimator (MLE) [50]. The value of the lower bound P_{min} and the exponent ζ are also shown. The CLCA parameters used were $D^{(min)} = 100$ people$/km^2$, $D^{(max)} = 1000$ people$/km^2$, $\delta = 10$ people$/km^2$, $\ell = 3\ km$, $A^* = 50\ km^2$ and $H^* = 0.50$.

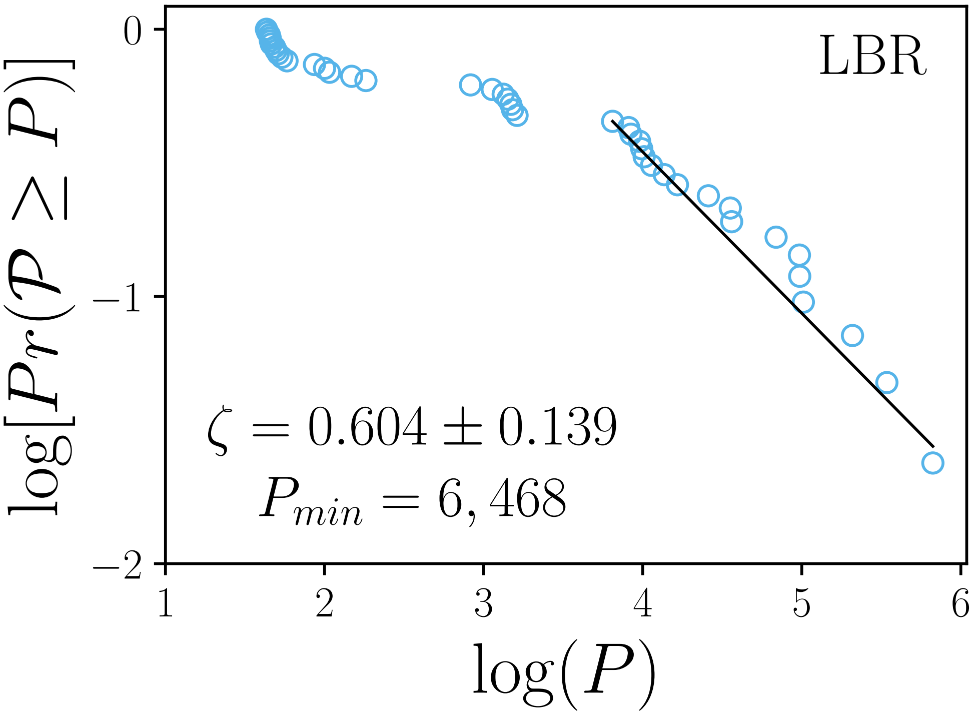

FIG. 34. **(Color online) Cumulative Distribution Function (CDF)** $Pr(\mathcal{P} \geq P)$ **versus population** P **in log-log scale.** Cities proposed by the City Local Clustering Algorithm (CLCA) are represented by light blue circles. The solid black line is the maximum likelihood power-law fit defined by the Maximum Likelihood Estimator (MLE) [50]. The value of the lower bound P_{min} and the exponent ζ are also shown. The CLCA parameters used were $D^{(min)} = 100$ people$/km^2$, $D^{(max)} = 1000$ people$/km^2$, $\delta = 10$ people$/km^2$, $\ell = 3$ km, $A^* = 50$ km^2 and $H^* = 0.50$.

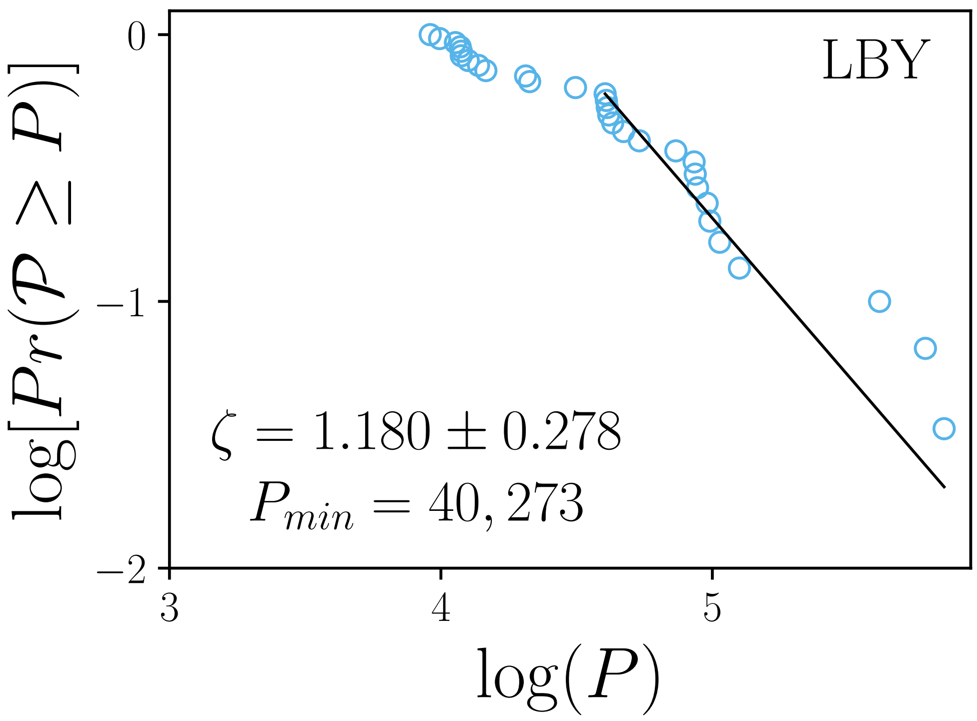

FIG. 35. **(Color online) Cumulative Distribution Function (CDF)** $Pr(\mathcal{P} \geq P)$ **versus population** P **in log-log scale.** Cities proposed by the City Local Clustering Algorithm (CLCA) are represented by light blue circles. The solid black line is the maximum likelihood power-law fit defined by the Maximum Likelihood Estimator (MLE) [50]. The value of the lower bound P_{min} and the exponent ζ are also shown. The CLCA parameters used were $D^{(min)} = 100$ people/km^2, $D^{(max)} = 1000$ people/km^2, $\delta = 10$ people/km^2, $\ell = 3$ km, $A^* = 50$ km^2 and $H^* = 0.50$.

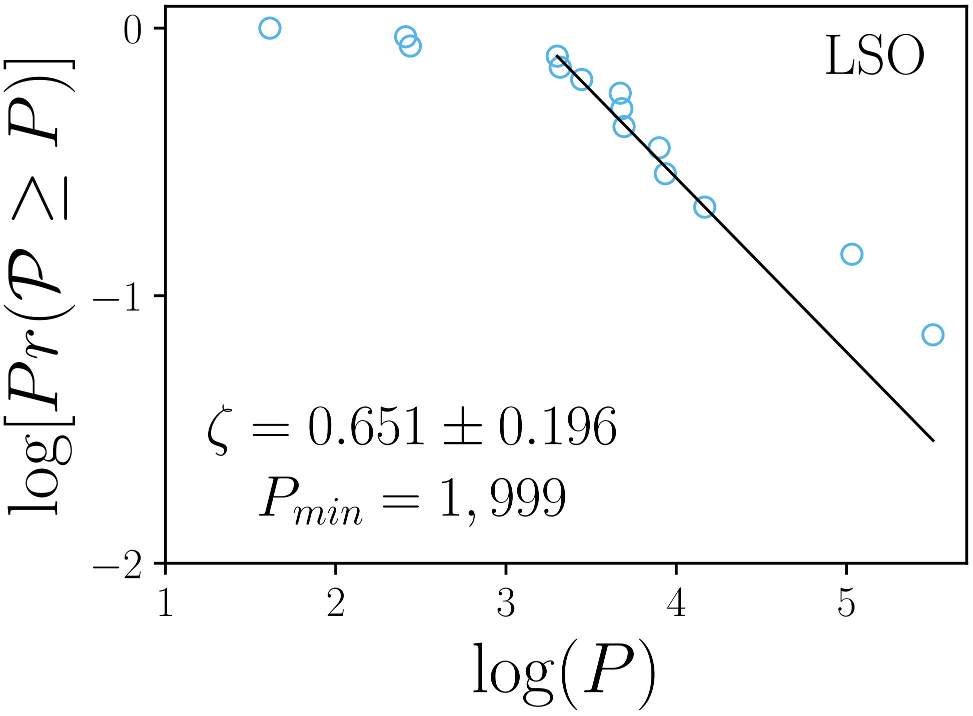

FIG. 36. **(Color online) Cumulative Distribution Function (CDF)** $Pr(\mathcal{P} \geq P)$ **versus population** P **in log-log scale.** Cities proposed by the City Local Clustering Algorithm (CLCA) are represented by light blue circles. The solid black line is the maximum likelihood power-law fit defined by the Maximum Likelihood Estimator (MLE) [50]. The value of the lower bound P_{min} and the exponent ζ are also shown. The CLCA parameters used were $D^{(min)} = 100$ people$/km^2$, $D^{(max)} = 1000$ people$/km^2$, $\delta = 10$ people$/km^2$, $\ell = 3\ km$, $A^* = 50\ km^2$ and $H^* = 0.50$.

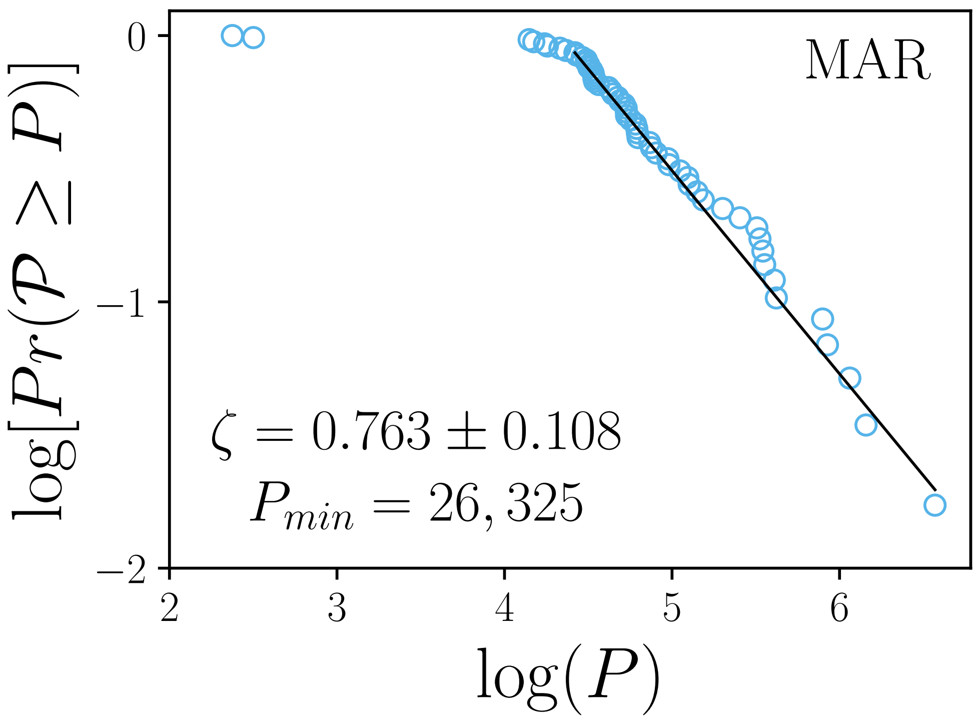

FIG. 37. **(Color online) Cumulative Distribution Function (CDF)** $Pr(\mathcal{P} \geq P)$ **versus population** P **in log-log scale.** Cities proposed by the City Local Clustering Algorithm (CLCA) are represented by light blue circles. The solid black line is the maximum likelihood power-law fit defined by the Maximum Likelihood Estimator (MLE) [50]. The value of the lower bound P_{min} and the exponent ζ are also shown. The CLCA parameters used were $D^{(min)} = 100$ people$/km^2$, $D^{(max)} = 1000$ people$/km^2$, $\delta = 10$ people$/km^2$, $\ell = 3\ km$, $A^* = 50\ km^2$ and $H^* = 0.50$.

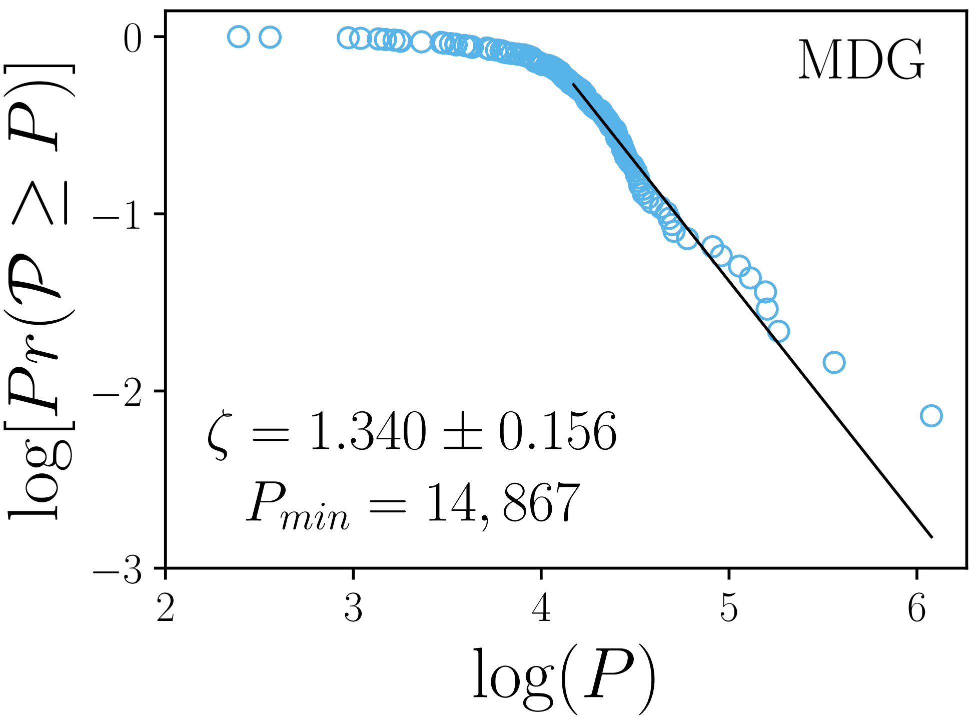

FIG. 38. **(Color online) Cumulative Distribution Function (CDF)** $Pr(\mathcal{P} \geq P)$ **versus population** P **in log-log scale.** Cities proposed by the City Local Clustering Algorithm (CLCA) are represented by light blue circles. The solid black line is the maximum likelihood power-law fit defined by the Maximum Likelihood Estimator (MLE) [50]. The value of the lower bound P_{min} and the exponent ζ are also shown. The CLCA parameters used were $D^{(min)} = 100$ people$/km^2$, $D^{(max)} = 1000$ people$/km^2$, $\delta = 10$ people$/km^2$, $\ell = 3\ km$, $A^* = 50\ km^2$ and $H^* = 0.50$.

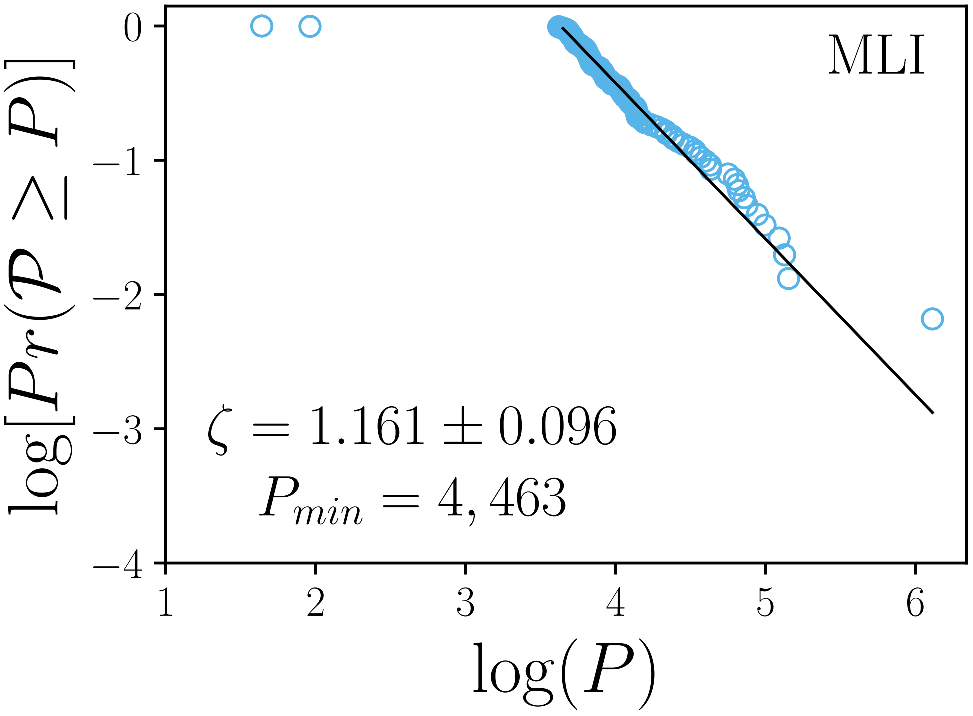

FIG. 39. **(Color online) Cumulative Distribution Function (CDF)** $Pr(\mathcal{P} \geq P)$ **versus population** P **in log-log scale.** Cities proposed by the City Local Clustering Algorithm (CLCA) are represented by light blue circles. The solid black line is the maximum likelihood power-law fit defined by the Maximum Likelihood Estimator (MLE) [50]. The value of the lower bound P_{min} and the exponent ζ are also shown. The CLCA parameters used were $D^{(min)} = 100$ people$/km^2$, $D^{(max)} = 1000$ people$/km^2$, $\delta = 10$ people$/km^2$, $\ell = 3$ km, $A^* = 50$ km^2 and $H^* = 0.50$.

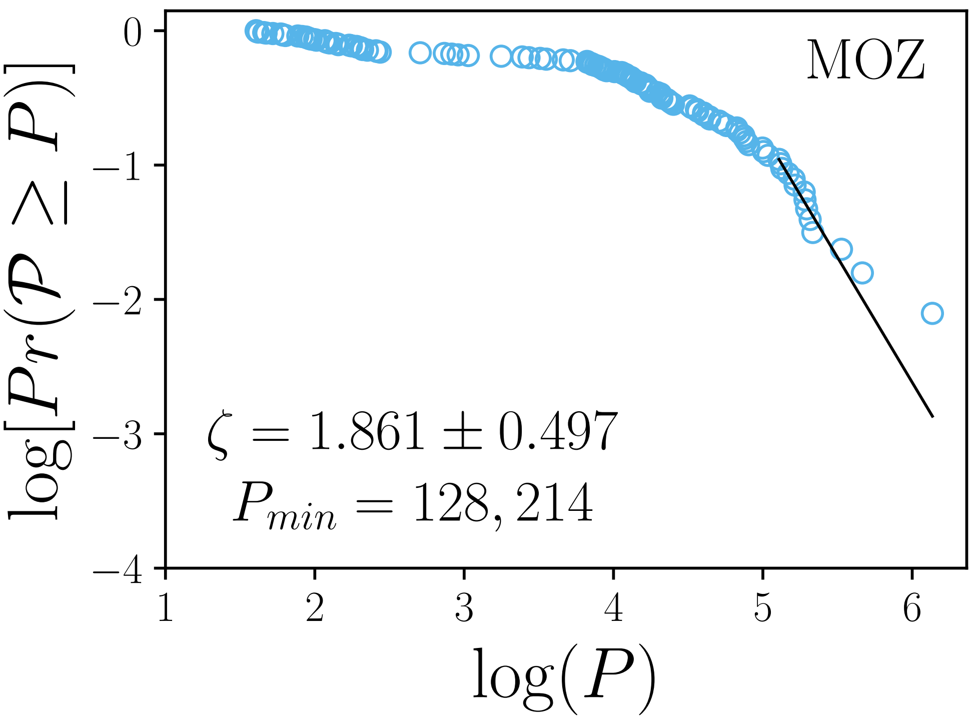

FIG. 40. **(Color online) Cumulative Distribution Function (CDF)** $Pr(\mathcal{P} \geq P)$ **versus population** P **in log-log scale.** Cities proposed by the City Local Clustering Algorithm (CLCA) are represented by light blue circles. The solid black line is the maximum likelihood power-law fit defined by the Maximum Likelihood Estimator (MLE) [50]. The value of the lower bound P_{min} and the exponent ζ are also shown. The CLCA parameters used were $D^{(min)} = 100$ people/km^2, $D^{(max)} = 1000$ people/km^2, $\delta = 10$ people/km^2, $\ell = 3$ km, $A^* = 50$ km^2 and $H^* = 0.50$.

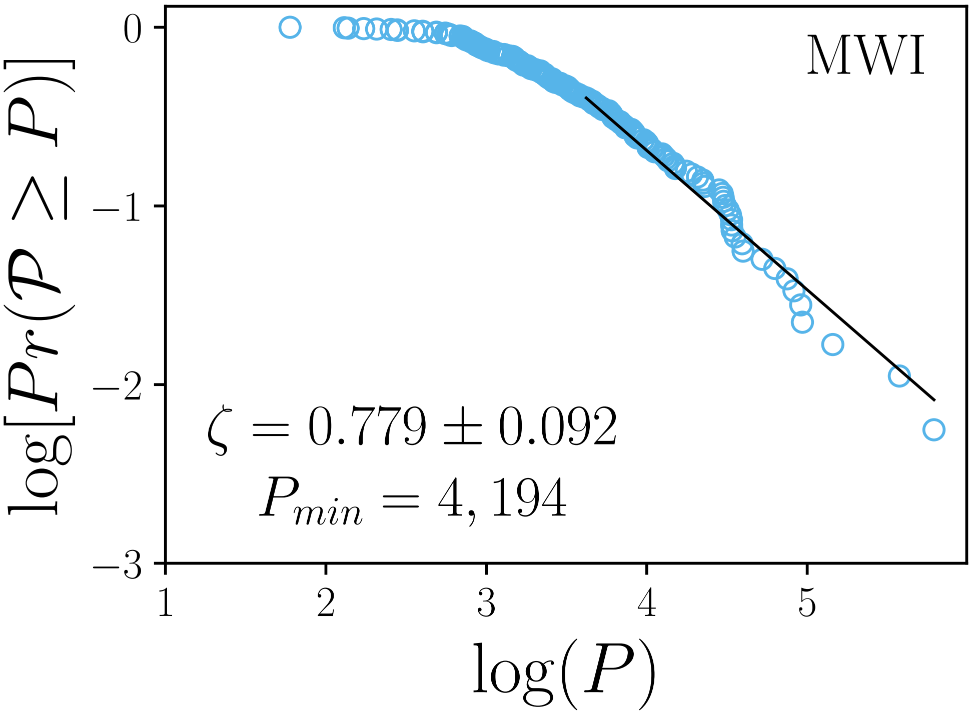

FIG. 41. **(Color online) Cumulative Distribution Function (CDF)** $Pr(\mathcal{P} \geq P)$ **versus population** P **in log-log scale.** Cities proposed by the City Local Clustering Algorithm (CLCA) are represented by light blue circles. The solid black line is the maximum likelihood power-law fit defined by the Maximum Likelihood Estimator (MLE) [50]. The value of the lower bound P_{min} and the exponent ζ are also shown. The CLCA parameters used were $D^{(min)} = 100$ people/km^2, $D^{(max)} = 1000$ people/km^2, $\delta = 10$ people/km^2, $\ell = 3$ km, $A^* = 50$ km^2 and $H^* = 0.50$.

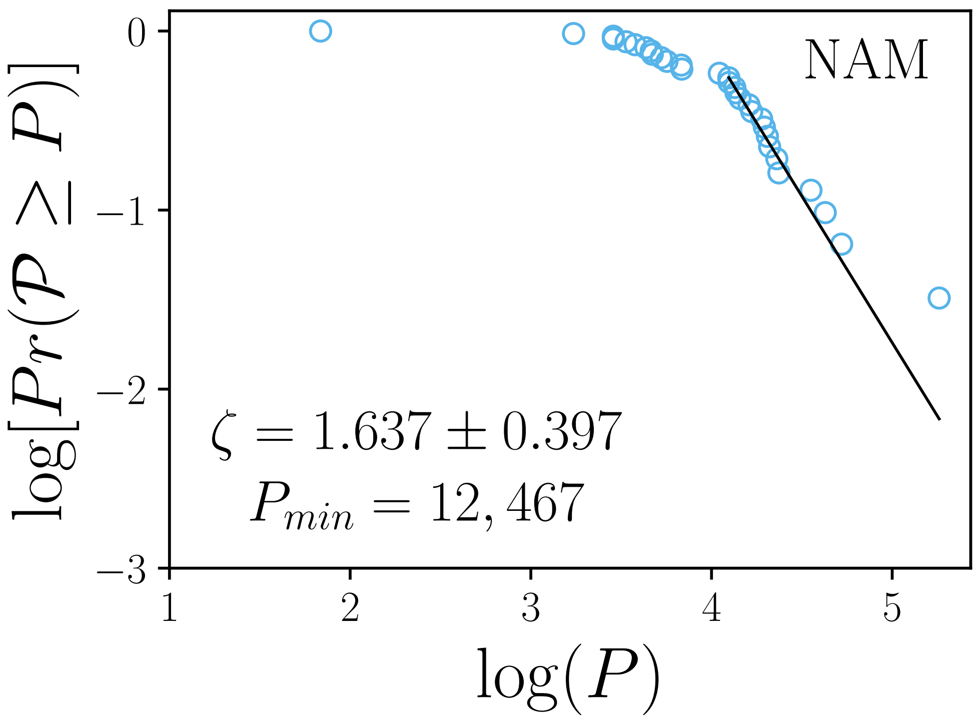

FIG. 42. **(Color online) Cumulative Distribution Function (CDF)** $Pr(\mathcal{P} \geq P)$ **versus population** P **in log-log scale.** Cities proposed by the City Local Clustering Algorithm (CLCA) are represented by light blue circles. The solid black line is the maximum likelihood power-law fit defined by the Maximum Likelihood Estimator (MLE) [50]. The value of the lower bound P_{min} and the exponent ζ are also shown. The CLCA parameters used were $D^{(min)} = 100$ people/km^2, $D^{(max)} = 1000$ people/km^2, $\delta = 10$ people/km^2, $\ell = 3$ km, $A^* = 50$ km^2 and $H^* = 0.50$.

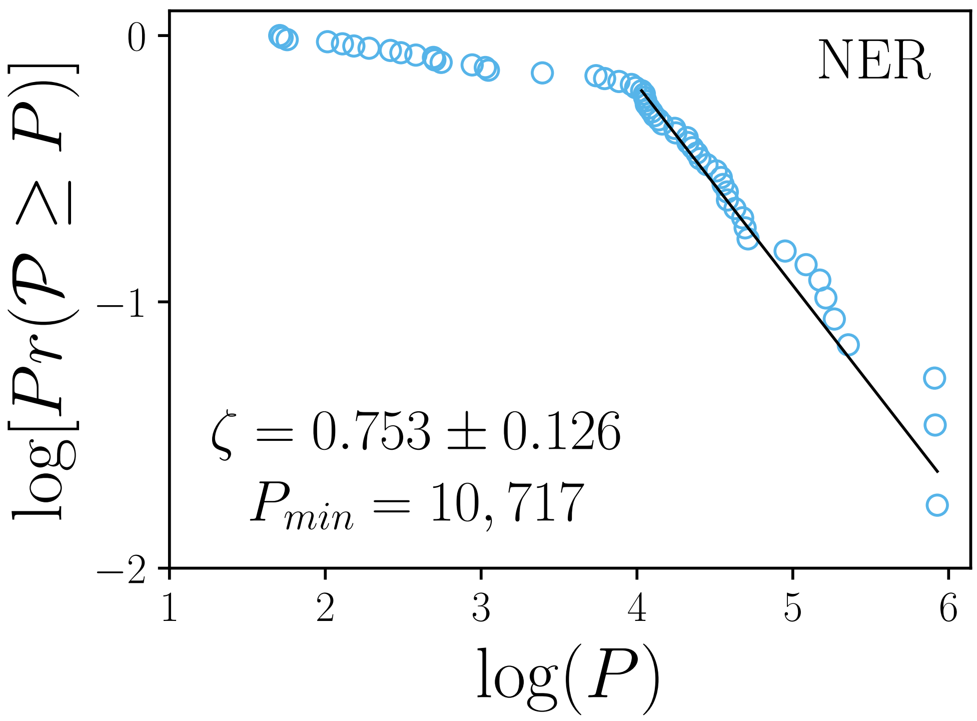

FIG. 43. **(Color online) Cumulative Distribution Function (CDF)** $Pr(\mathcal{P} \geq P)$ **versus population** P **in log-log scale.** Cities proposed by the City Local Clustering Algorithm (CLCA) are represented by light blue circles. The solid black line is the maximum likelihood power-law fit defined by the Maximum Likelihood Estimator (MLE) [50]. The value of the lower bound P_{min} and the exponent ζ are also shown. The CLCA parameters used were $D^{(min)} = 100$ people$/km^2$, $D^{(max)} = 1000$ people$/km^2$, $\delta = 10$ people$/km^2$, $\ell = 3\ km$, $A^* = 50\ km^2$ and $H^* = 0.50$.

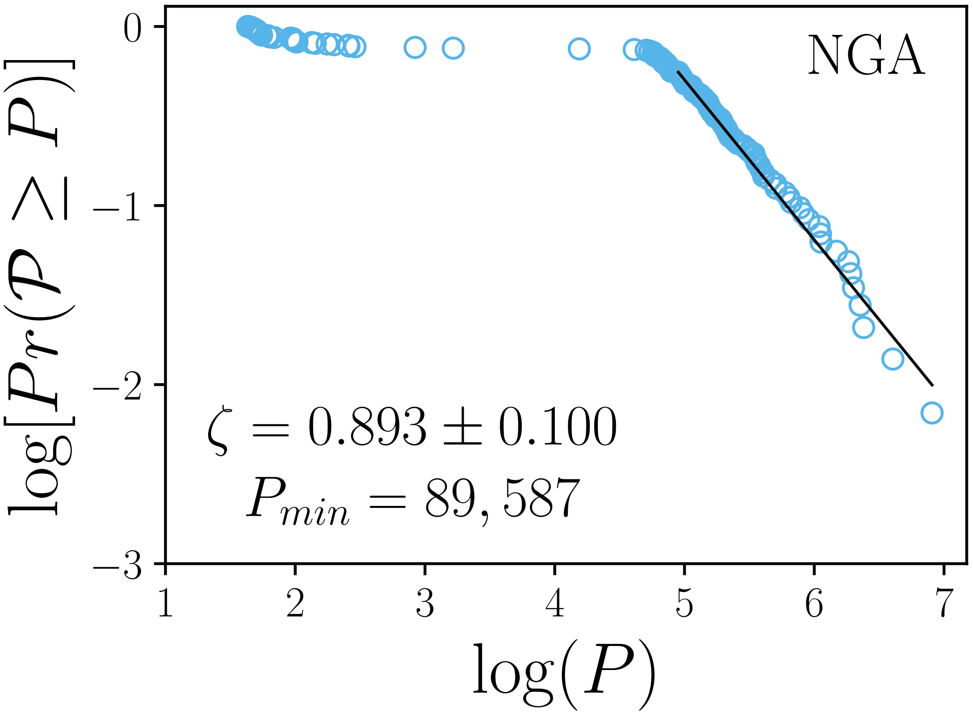

FIG. 44. **(Color online) Cumulative Distribution Function (CDF)** $Pr(\mathcal{P} \geq P)$ **versus population** P **in log-log scale.** Cities proposed by the City Local Clustering Algorithm (CLCA) are represented by light blue circles. The solid black line is the maximum likelihood power-law fit defined by the Maximum Likelihood Estimator (MLE) [50]. The value of the lower bound P_{min} and the exponent ζ are also shown. The CLCA parameters used were $D^{(min)} = 100$ people/km^2, $D^{(max)} = 1000$ people/km^2, $\delta = 10$ people/km^2, $\ell = 3$ km, $A^* = 50$ km^2 and $H^* = 0.50$.

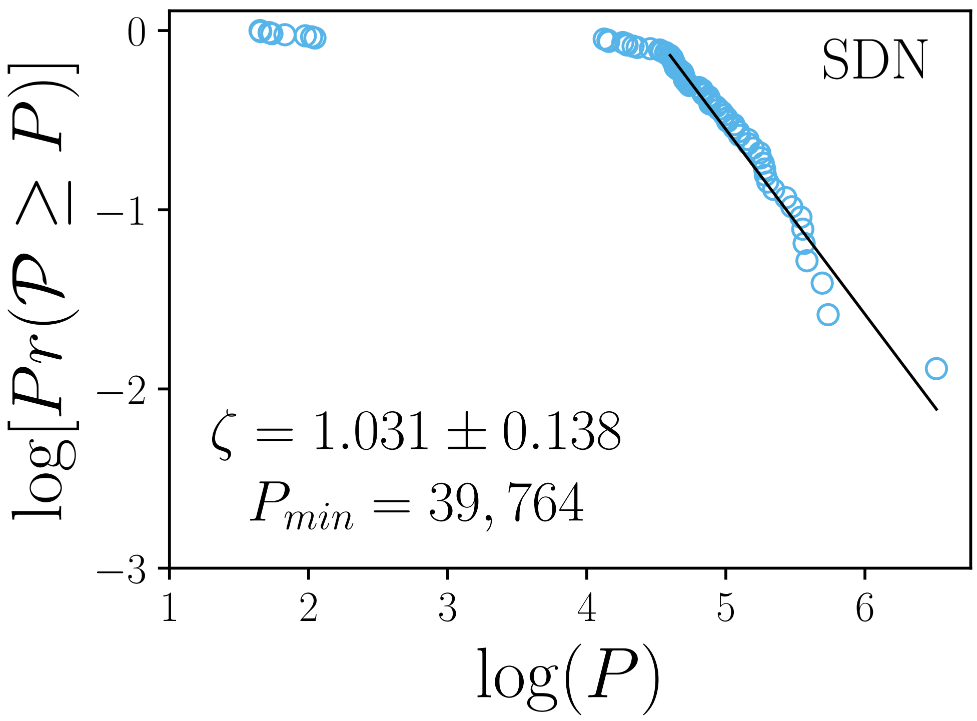

FIG. 45. **(Color online) Cumulative Distribution Function (CDF)** $Pr(\mathcal{P} \geq P)$ **versus population** P **in log-log scale.** Cities proposed by the City Local Clustering Algorithm (CLCA) are represented by light blue circles. The solid black line is the maximum likelihood power-law fit defined by the Maximum Likelihood Estimator (MLE) [50]. The value of the lower bound P_{min} and the exponent ζ are also shown. The CLCA parameters used were $D^{(min)} = 100$ people$/km^2$, $D^{(max)} = 1000$ people$/km^2$, $\delta = 10$ people$/km^2$, $\ell = 3\ km$, $A^* = 50\ km^2$ and $H^* = 0.50$.

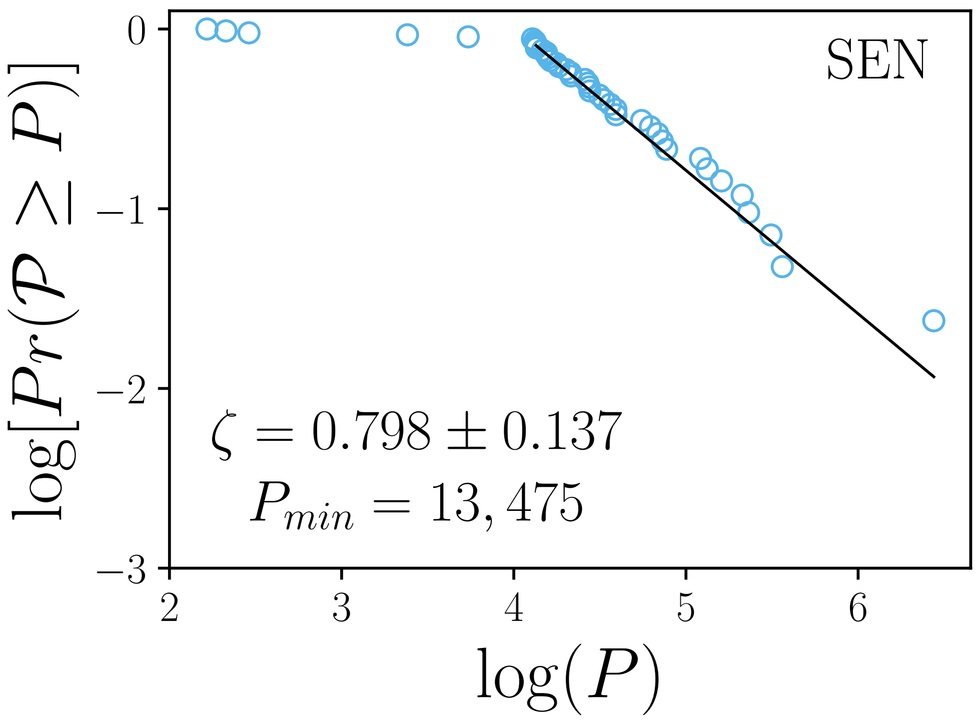

FIG. 46. **(Color online) Cumulative Distribution Function (CDF)** $Pr(\mathcal{P} \geq P)$ **versus population** P **in log-log scale.** Cities proposed by the City Local Clustering Algorithm (CLCA) are represented by light blue circles. The solid black line is the maximum likelihood power-law fit defined by the Maximum Likelihood Estimator (MLE) [50]. The value of the lower bound P_{min} and the exponent ζ are also shown. The CLCA parameters used were $D^{(min)} = 100$ people/km^2, $D^{(max)} = 1000$ people/km^2, $\delta = 10$ people/km^2, $\ell = 3$ km, $A^* = 50$ km^2 and $H^* = 0.50$.

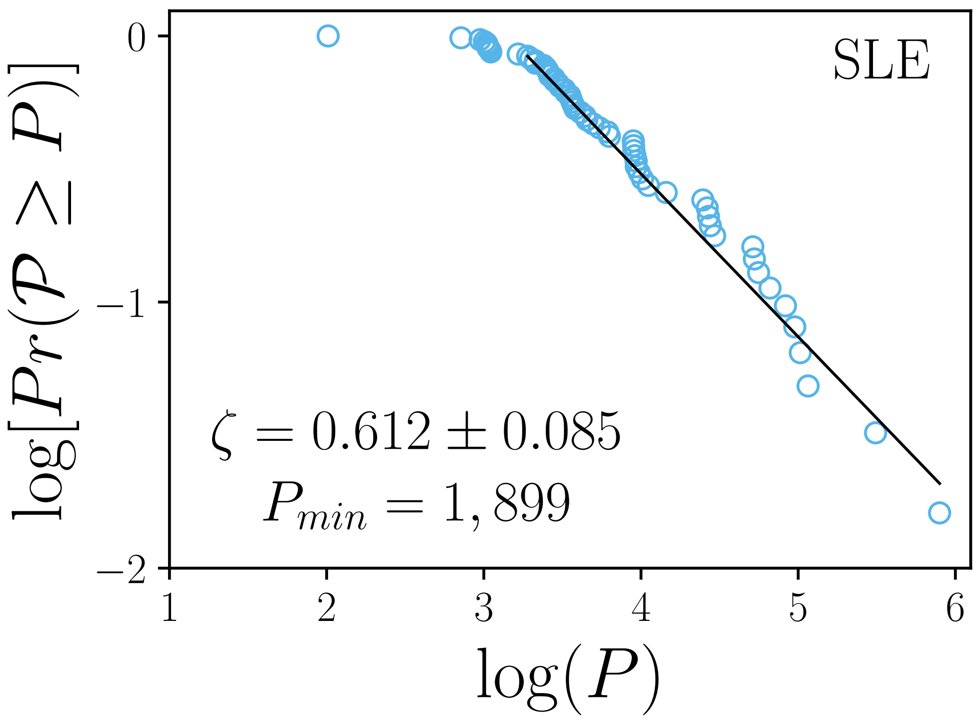

FIG. 47. **(Color online) Cumulative Distribution Function (CDF)** $Pr(\mathcal{P} \geq P)$ **versus population** P **in log-log scale.** Cities proposed by the City Local Clustering Algorithm (CLCA) are represented by light blue circles. The solid black line is the maximum likelihood power-law fit defined by the Maximum Likelihood Estimator (MLE) [50]. The value of the lower bound P_{min} and the exponent ζ are also shown. The CLCA parameters used were $D^{(min)} = 100$ people$/km^2$, $D^{(max)} = 1000$ people$/km^2$, $\delta = 10$ people$/km^2$, $\ell = 3\ km$, $A^* = 50\ km^2$ and $H^* = 0.50$.

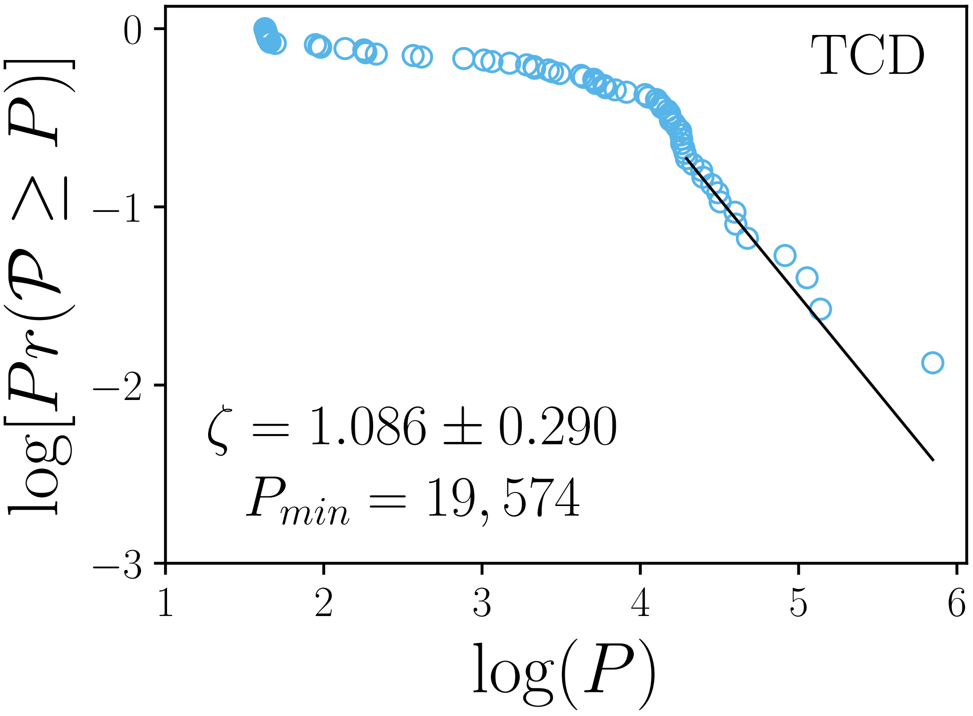

FIG. 48. **(Color online) Cumulative Distribution Function (CDF)** $Pr(\mathcal{P} \geq P)$ **versus population** P **in log-log scale.** Cities proposed by the City Local Clustering Algorithm (CLCA) are represented by light blue circles. The solid black line is the maximum likelihood power-law fit defined by the Maximum Likelihood Estimator (MLE) [50]. The value of the lower bound P_{min} and the exponent ζ are also shown. The CLCA parameters used were $D^{(min)} = 100$ people$/km^2$, $D^{(max)} = 1000$ people$/km^2$, $\delta = 10$ people$/km^2$, $\ell = 3\ km$, $A^* = 50\ km^2$ and $H^* = 0.50$.

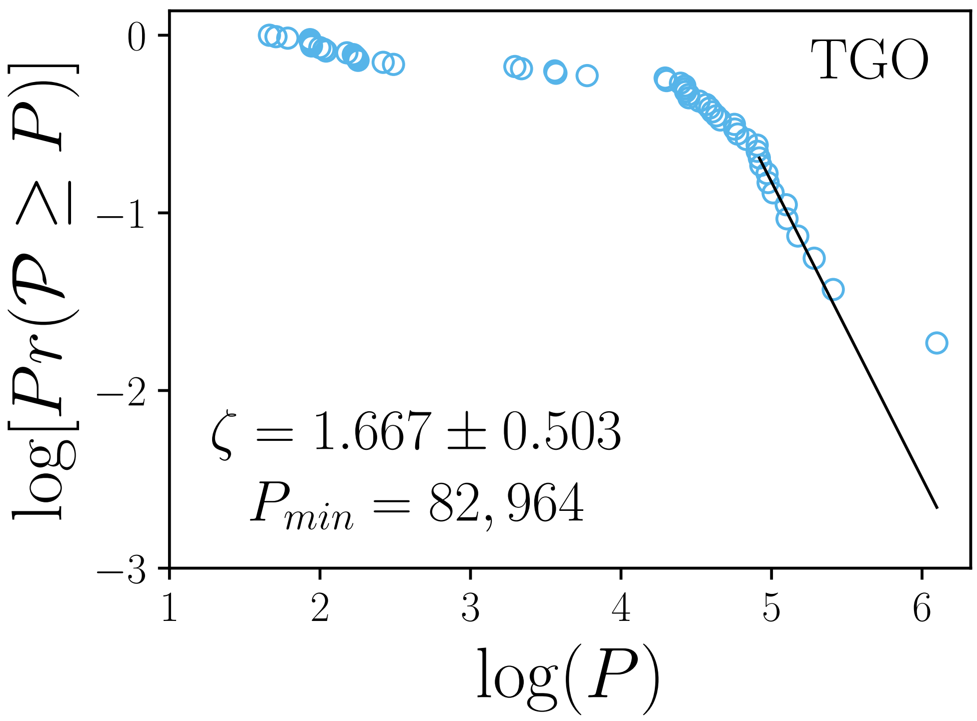

FIG. 49. **(Color online) Cumulative Distribution Function (CDF)** $Pr(\mathcal{P} \geq P)$ **versus population** P **in log-log scale.** Cities proposed by the City Local Clustering Algorithm (CLCA) are represented by light blue circles. The solid black line is the maximum likelihood power-law fit defined by the Maximum Likelihood Estimator (MLE) [50]. The value of the lower bound P_{min} and the exponent ζ are also shown. The CLCA parameters used were $D^{(min)} = 100$ people/km^2, $D^{(max)} = 1000$ people/km^2, $\delta = 10$ people/km^2, $\ell = 3$ km, $A^* = 50$ km^2 and $H^* = 0.50$.

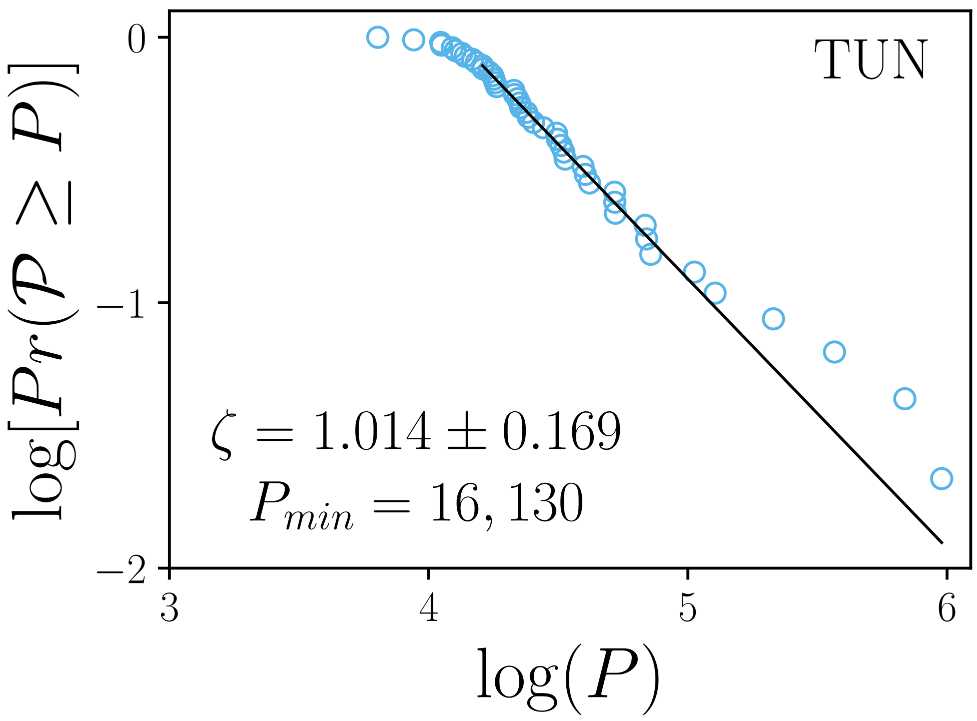

FIG. 50. **(Color online) Cumulative Distribution Function (CDF)** $Pr(\mathcal{P} \geq P)$ **versus population** P **in log-log scale.** Cities proposed by the City Local Clustering Algorithm (CLCA) are represented by light blue circles. The solid black line is the maximum likelihood power-law fit defined by the Maximum Likelihood Estimator (MLE) [50]. The value of the lower bound P_{min} and the exponent ζ are also shown. The CLCA parameters used were $D^{(min)} = 100$ people$/km^2$, $D^{(max)} = 1000$ people$/km^2$, $\delta = 10$ people$/km^2$, $\ell = 3$ km, $A^* = 50$ km^2 and $H^* = 0.50$.

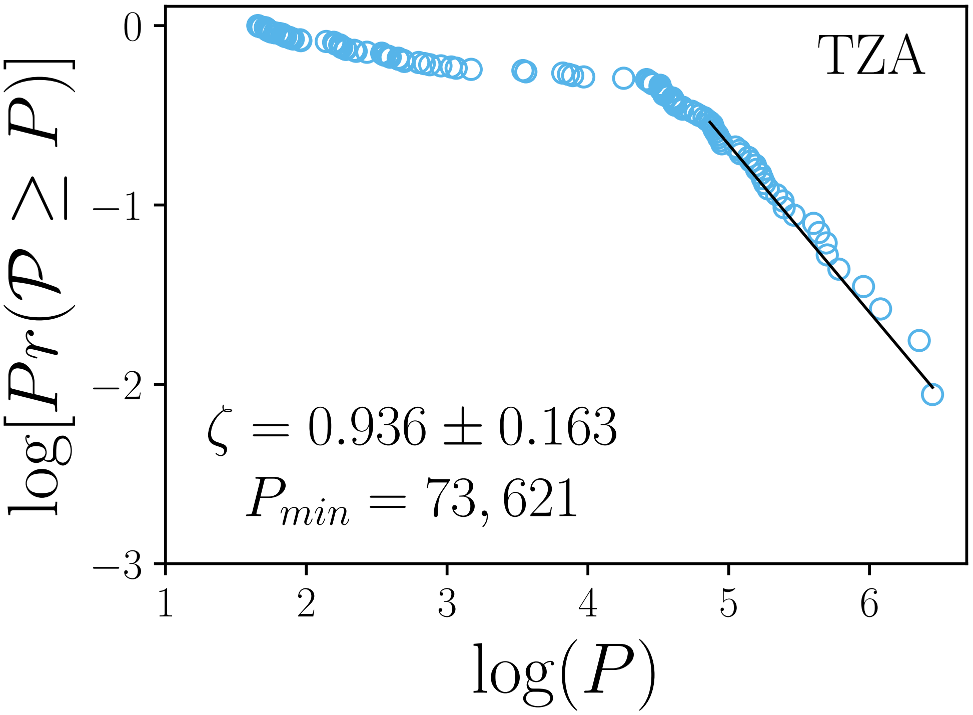

FIG. 51. **(Color online) Cumulative Distribution Function (CDF)** $Pr(\mathcal{P} \geq P)$ **versus population** P **in log-log scale.** Cities proposed by the City Local Clustering Algorithm (CLCA) are represented by light blue circles. The solid black line is the maximum likelihood power-law fit defined by the Maximum Likelihood Estimator (MLE) [50]. The value of the lower bound P_{min} and the exponent ζ are also shown. The CLCA parameters used were $D^{(min)} = 100$ people$/km^2$, $D^{(max)} = 1000$ people$/km^2$, $\delta = 10$ people$/km^2$, $\ell = 3\ km$, $A^* = 50\ km^2$ and $H^* = 0.50$.

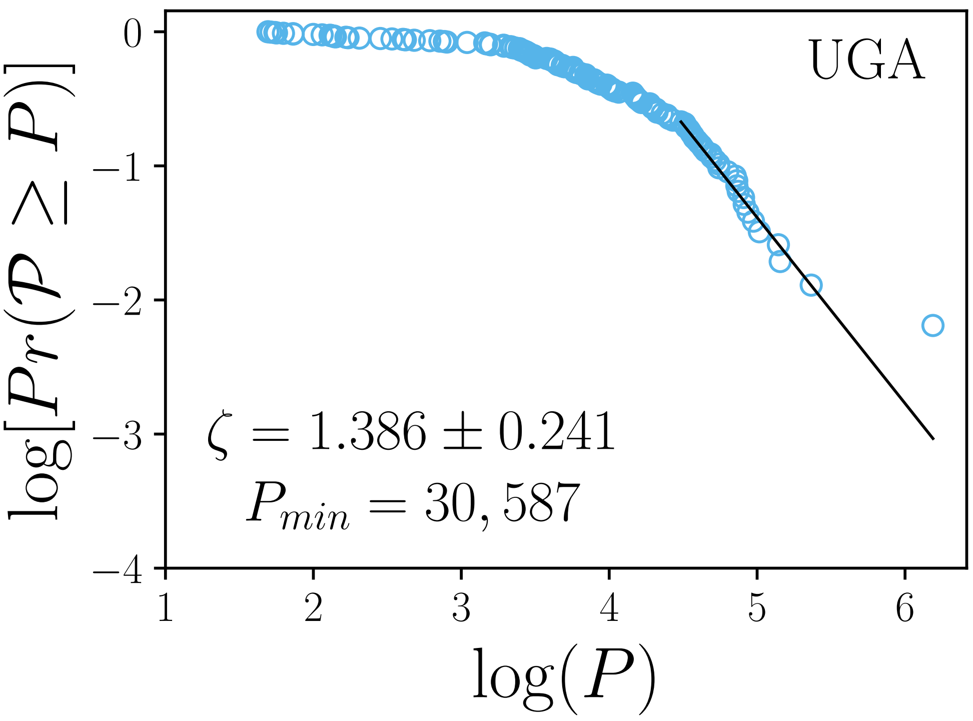

FIG. 52. **(Color online) Cumulative Distribution Function (CDF)** $Pr(\mathcal{P} \geq P)$ **versus population** P **in log-log scale.** Cities proposed by the City Local Clustering Algorithm (CLCA) are represented by light blue circles. The solid black line is the maximum likelihood power-law fit defined by the Maximum Likelihood Estimator (MLE) [50]. The value of the lower bound P_{min} and the exponent ζ are also shown. The CLCA parameters used were $D^{(min)} = 100$ people/km^2, $D^{(max)} = 1000$ people/km^2, $\delta = 10$ people/km^2, $\ell = 3$ km, $A^* = 50$ km^2 and $H^* = 0.50$.

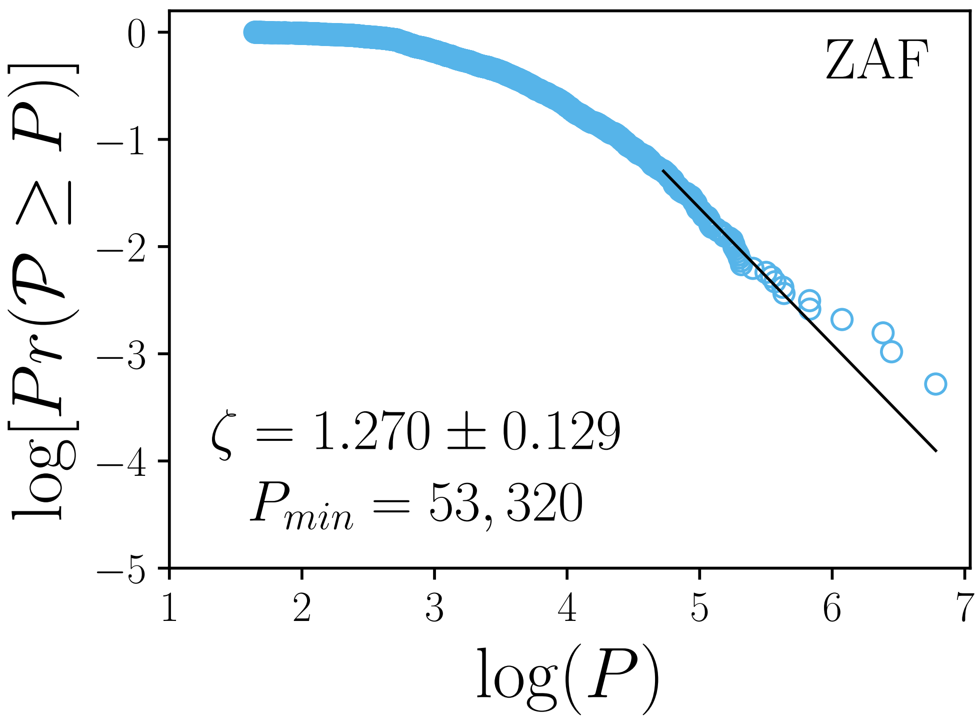

FIG. 53. **(Color online) Cumulative Distribution Function (CDF)** $Pr(\mathcal{P} \geq P)$ **versus population** P **in log-log scale.** Cities proposed by the City Local Clustering Algorithm (CLCA) are represented by light blue circles. The solid black line is the maximum likelihood power-law fit defined by the Maximum Likelihood Estimator (MLE) [50]. The value of the lower bound P_{min} and the exponent ζ are also shown. The CLCA parameters used were $D^{(min)} = 100$ people$/km^2$, $D^{(max)} = 1000$ people$/km^2$, $\delta = 10$ people$/km^2$, $\ell = 3\ km$, $A^* = 50\ km^2$ and $H^* = 0.50$.

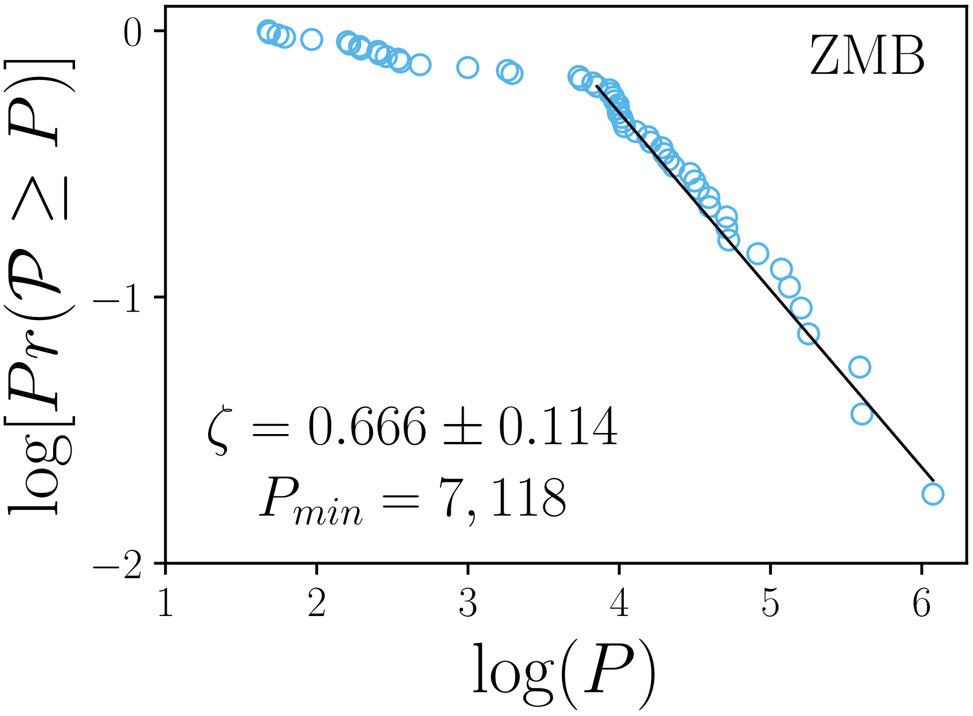

FIG. 54. **(Color online) Cumulative Distribution Function (CDF)** $Pr(\mathcal{P} \geq P)$ **versus population** P **in log-log scale.** Cities proposed by the City Local Clustering Algorithm (CLCA) are represented by light blue circles. The solid black line is the maximum likelihood power-law fit defined by the Maximum Likelihood Estimator (MLE) [50]. The value of the lower bound P_{min} and the exponent ζ are also shown. The CLCA parameters used were $D^{(min)} = 100$ people/km^2, $D^{(max)} = 1000$ people/km^2, $\delta = 10$ people/km^2, $\ell = 3$ km, $A^* = 50$ km^2 and $H^* = 0.50$.

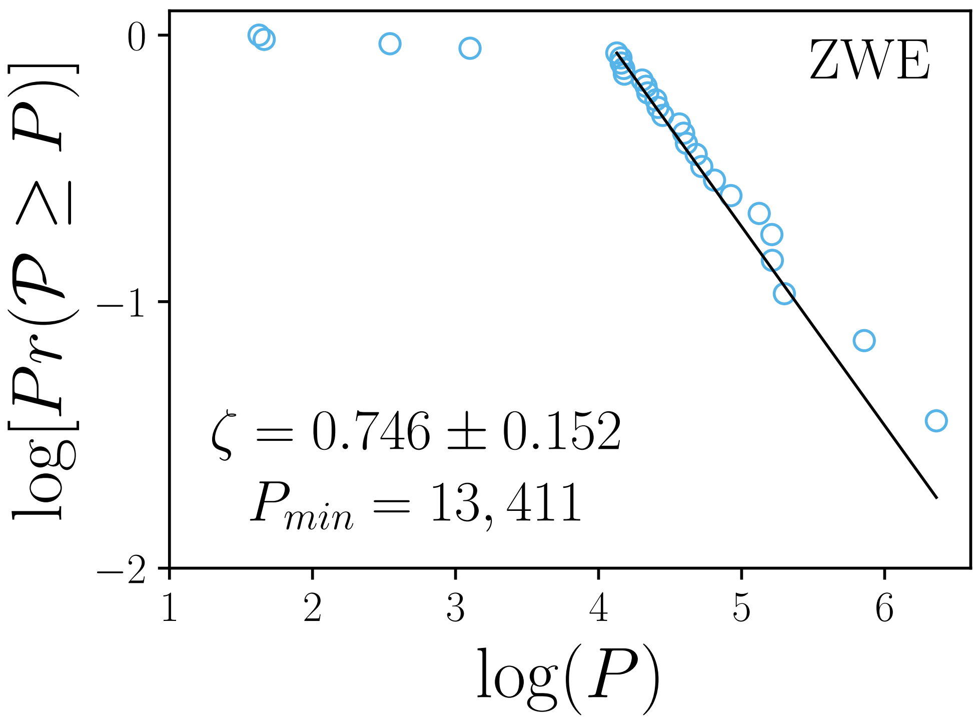

FIG. 55. **(Color online) Cumulative Distribution Function (CDF)** $Pr(\mathcal{P} \geq P)$ **versus population** P **in log-log scale.** Cities proposed by the City Local Clustering Algorithm (CLCA) are represented by light blue circles. The solid black line is the maximum likelihood power-law fit defined by the Maximum Likelihood Estimator (MLE) [50]. The value of the lower bound P_{min} and the exponent ζ are also shown. The CLCA parameters used were $D^{(min)} = 100$ people$/km^2$, $D^{(max)} = 1000$ people$/km^2$, $\delta = 10$ people$/km^2$, $\ell = 3\ km$, $A^* = 50\ km^2$ and $H^* = 0.50$.

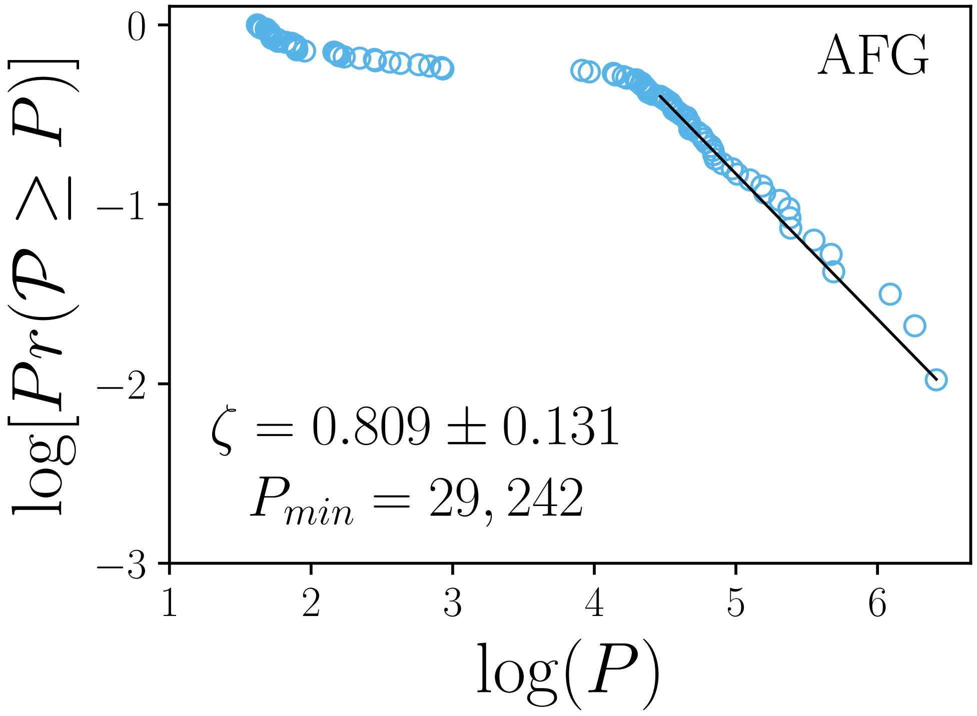

FIG. 56. **(Color online) Cumulative Distribution Function (CDF)** $Pr(\mathcal{P} \geq P)$ **versus population** P **in log-log scale.** Cities proposed by the City Local Clustering Algorithm (CLCA) are represented by light blue circles. The solid black line is the maximum likelihood power-law fit defined by the Maximum Likelihood Estimator (MLE) [50]. The value of the lower bound P_{min} and the exponent ζ are also shown. The CLCA parameters used were $D^{(min)} = 100$ people/km^2, $D^{(max)} = 1000$ people/km^2, $\delta = 10$ people/km^2, $\ell = 3$ km, $A^* = 50$ km^2 and $H^* = 0.50$.

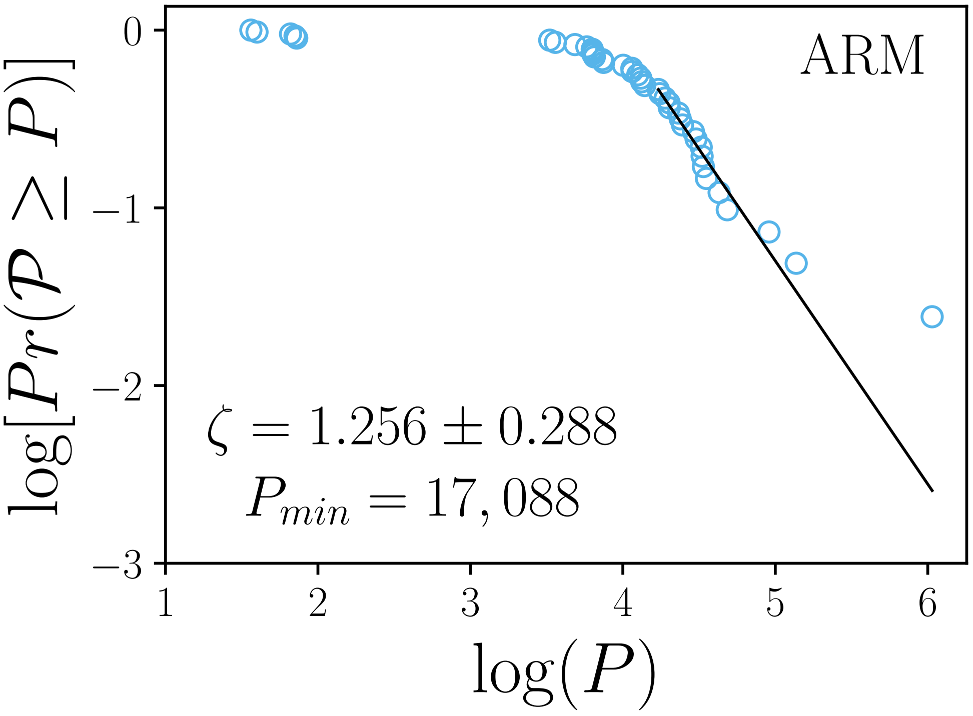

FIG. 57. **(Color online) Cumulative Distribution Function (CDF)** $Pr(\mathcal{P} \geq P)$ **versus population** P **in log-log scale.** Cities proposed by the City Local Clustering Algorithm (CLCA) are represented by light blue circles. The solid black line is the maximum likelihood power-law fit defined by the Maximum Likelihood Estimator (MLE) [50]. The value of the lower bound P_{min} and the exponent ζ are also shown. The CLCA parameters used were $D^{(min)} = 100$ people/km^2, $D^{(max)} = 1000$ people/km^2, $\delta = 10$ people/km^2, $\ell = 3$ km, $A^* = 50$ km^2 and $H^* = 0.50$.

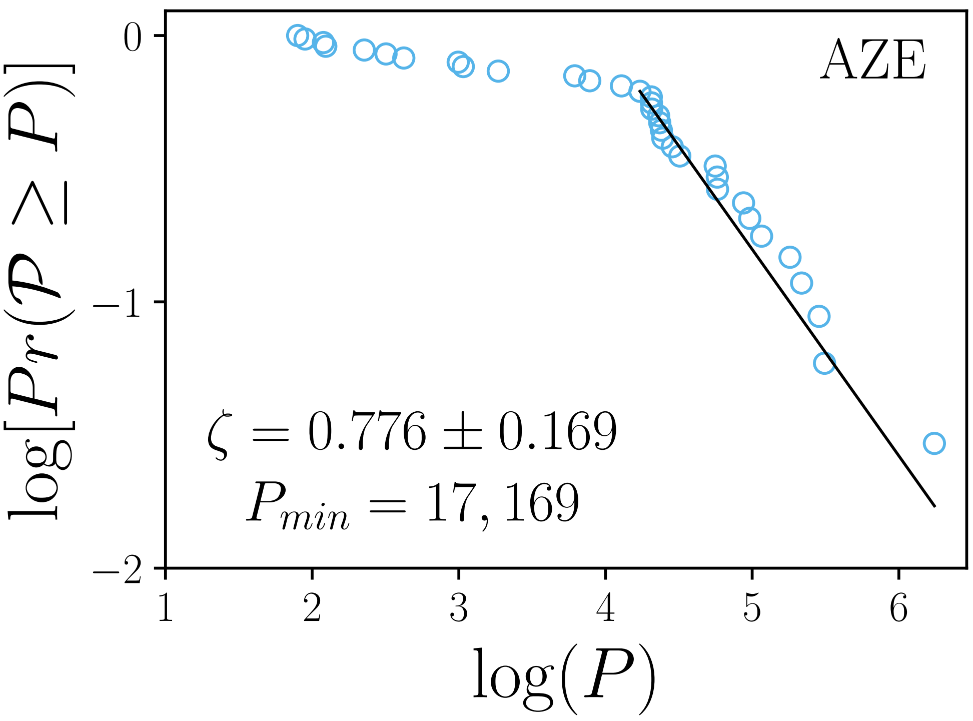

FIG. 58. **(Color online) Cumulative Distribution Function (CDF)** $Pr(\mathcal{P} \geq P)$ **versus population** P **in log-log scale.** Cities proposed by the City Local Clustering Algorithm (CLCA) are represented by light blue circles. The solid black line is the maximum likelihood power-law fit defined by the Maximum Likelihood Estimator (MLE) [50]. The value of the lower bound P_{min} and the exponent ζ are also shown. The CLCA parameters used were $D^{(min)} = 100$ people$/km^2$, $D^{(max)} = 1000$ people$/km^2$, $\delta = 10$ people$/km^2$, $\ell = 3$ km, $A^* = 50$ km^2 and $H^* = 0.50$.

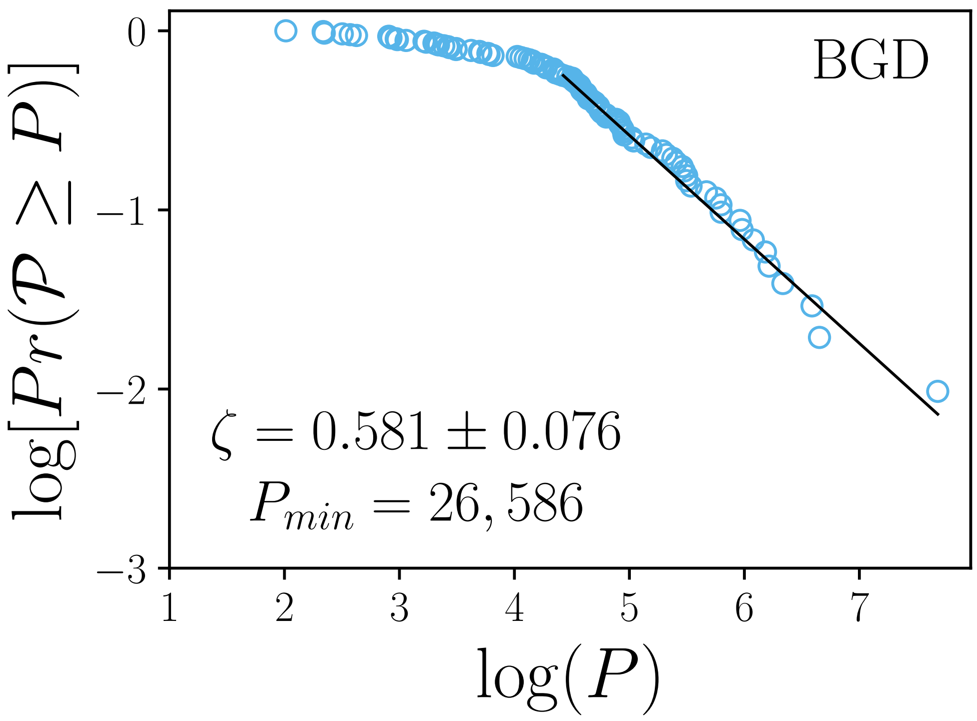

FIG. 59. **(Color online) Cumulative Distribution Function (CDF)** $Pr(\mathcal{P} \geq P)$ **versus population** P **in log-log scale.** Cities proposed by the City Local Clustering Algorithm (CLCA) are represented by light blue circles. The solid black line is the maximum likelihood power-law fit defined by the Maximum Likelihood Estimator (MLE) [50]. The value of the lower bound P_{min} and the exponent ζ are also shown. The CLCA parameters used were $D^{(min)} = 100$ people/km^2, $D^{(max)} = 1000$ people/km^2, $\delta = 10$ people/km^2, $\ell = 3$ km, $A^* = 50$ km^2 and $H^* = 0.50$.

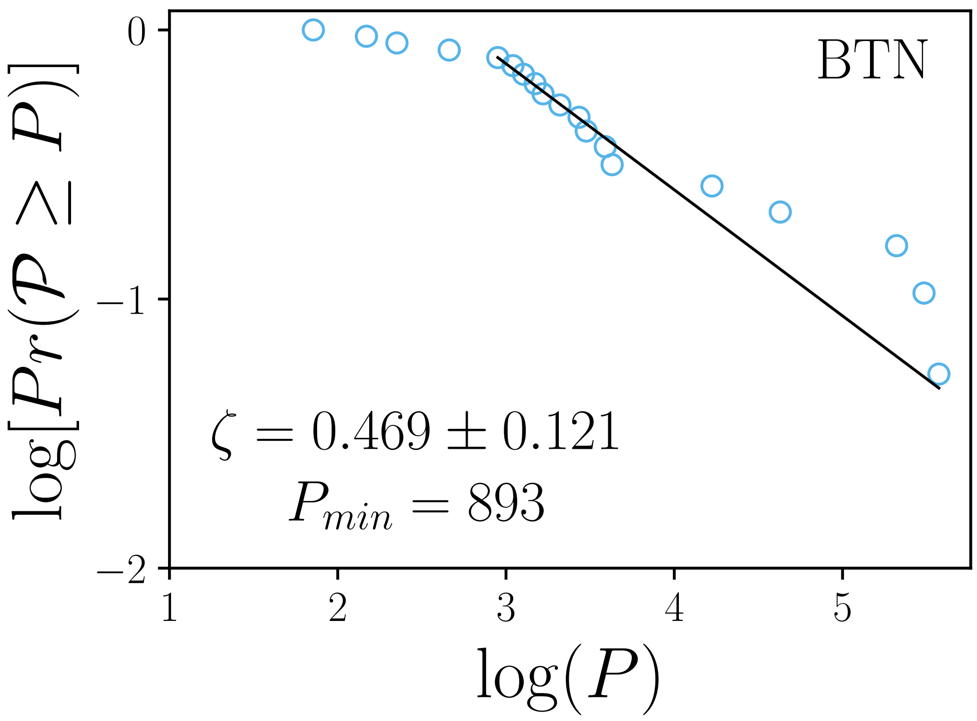

FIG. 60. **(Color online) Cumulative Distribution Function (CDF)** $Pr(\mathcal{P} \geq P)$ **versus population** P **in log-log scale.** Cities proposed by the City Local Clustering Algorithm (CLCA) are represented by light blue circles. The solid black line is the maximum likelihood power-law fit defined by the Maximum Likelihood Estimator (MLE) [50]. The value of the lower bound P_{min} and the exponent ζ are also shown. The CLCA parameters used were $D^{(min)} = 100$ people$/km^2$, $D^{(max)} = 1000$ people$/km^2$, $\delta = 10$ people$/km^2$, $\ell = 3\ km$, $A^* = 50\ km^2$ and $H^* = 0.50$.

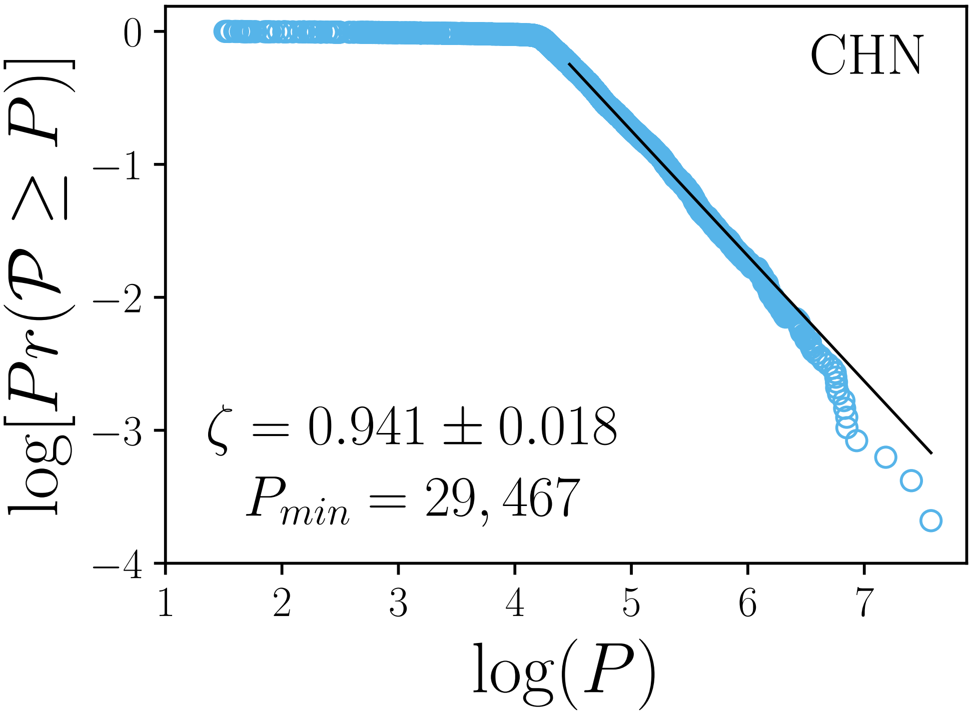

FIG. 61. **(Color online) Cumulative Distribution Function (CDF)** $Pr(\mathcal{P} \geq P)$ **versus population** P **in log-log scale.** Cities proposed by the City Local Clustering Algorithm (CLCA) are represented by light blue circles. The solid black line is the maximum likelihood power-law fit defined by the Maximum Likelihood Estimator (MLE) [50]. The value of the lower bound P_{min} and the exponent ζ are also shown. The CLCA parameters used were $D^{(min)} = 100$ people/km^2, $D^{(max)} = 1000$ people/km^2, $\delta = 10$ people/km^2, $\ell = 3$ km, $A^* = 50$ km^2 and $H^* = 0.50$.

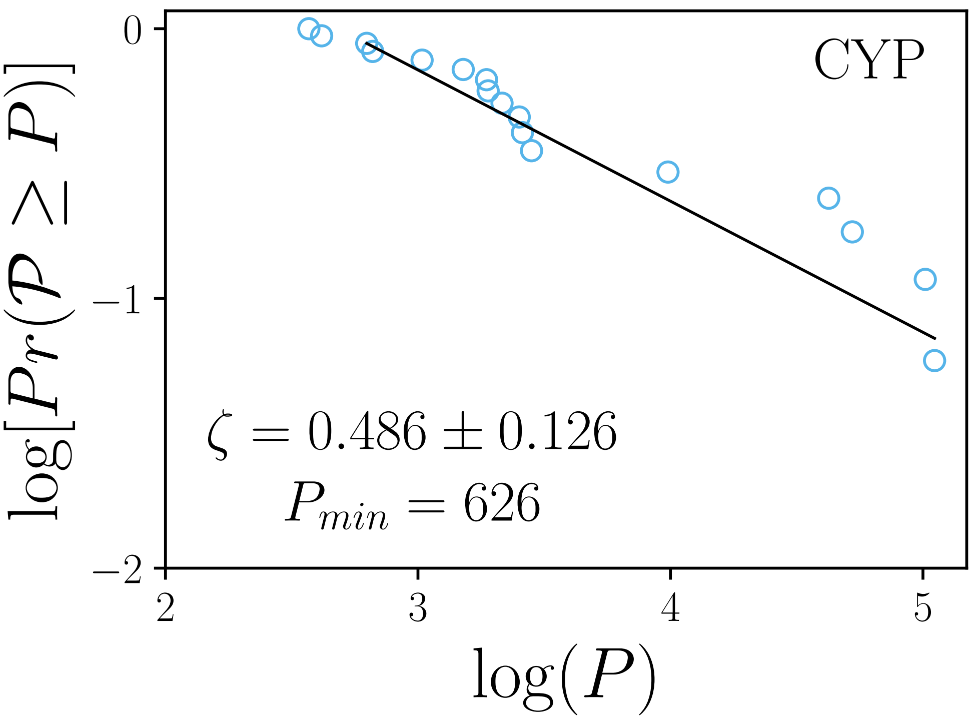

FIG. 62. **(Color online) Cumulative Distribution Function (CDF)** $Pr(\mathcal{P} \geq P)$ **versus population** P **in log-log scale.** Cities proposed by the City Local Clustering Algorithm (CLCA) are represented by light blue circles. The solid black line is the maximum likelihood power-law fit defined by the Maximum Likelihood Estimator (MLE) [50]. The value of the lower bound P_{min} and the exponent ζ are also shown. The CLCA parameters used were $D^{(min)} = 100$ people$/km^2$, $D^{(max)} = 1000$ people$/km^2$, $\delta = 10$ people$/km^2$, $\ell = 3\ km$, $A^* = 50\ km^2$ and $H^* = 0.50$.

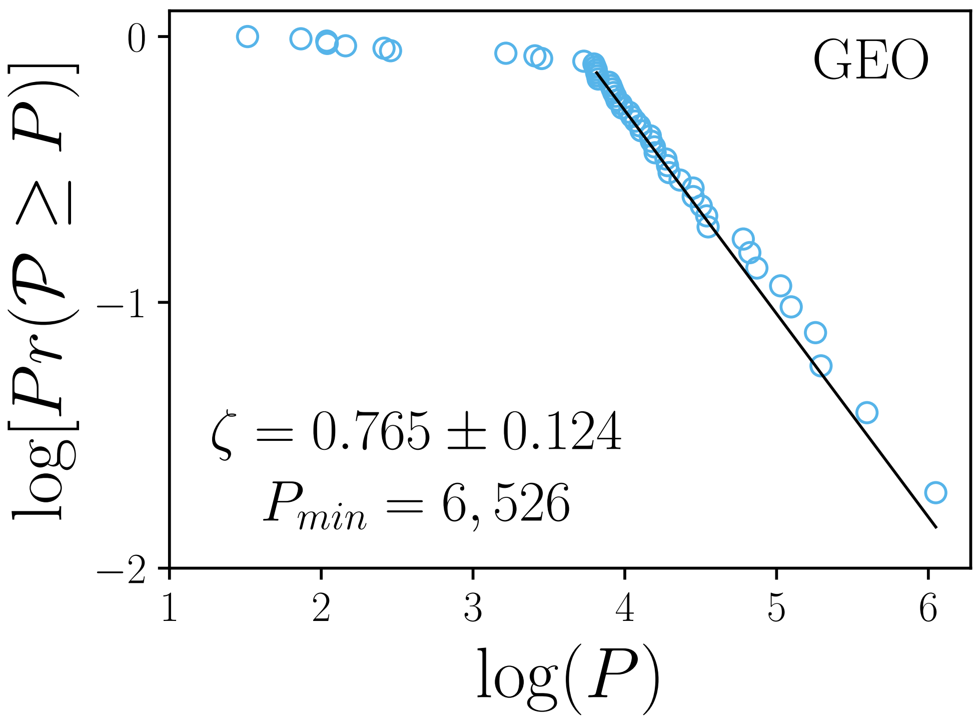

FIG. 63. **(Color online) Cumulative Distribution Function (CDF)** $Pr(\mathcal{P} \geq P)$ **versus population** P **in log-log scale.** Cities proposed by the City Local Clustering Algorithm (CLCA) are represented by light blue circles. The solid black line is the maximum likelihood power-law fit defined by the Maximum Likelihood Estimator (MLE) [50]. The value of the lower bound P_{min} and the exponent ζ are also shown. The CLCA parameters used were $D^{(min)} = 100$ people/km^2, $D^{(max)} = 1000$ people/km^2, $\delta = 10$ people/km^2, $\ell = 3$ km, $A^* = 50$ km^2 and $H^* = 0.50$.

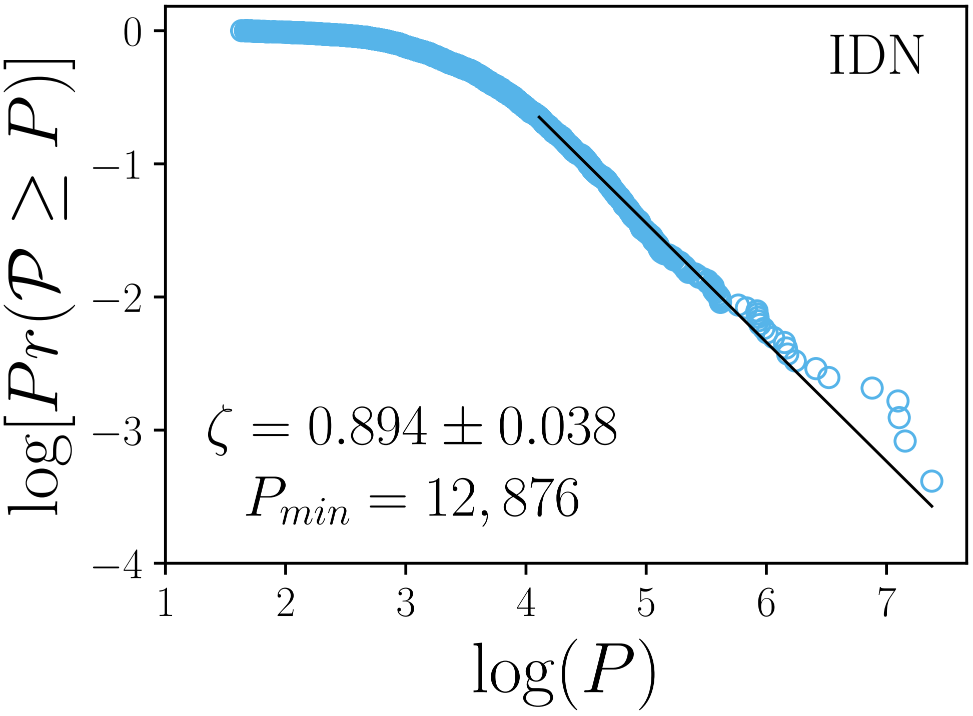

FIG. 64. **(Color online) Cumulative Distribution Function (CDF)** $Pr(\mathcal{P} \geq P)$ **versus population** P **in log-log scale.** Cities proposed by the City Local Clustering Algorithm (CLCA) are represented by light blue circles. The solid black line is the maximum likelihood power-law fit defined by the Maximum Likelihood Estimator (MLE) [50]. The value of the lower bound P_{min} and the exponent ζ are also shown. The CLCA parameters used were $D^{(min)} = 100$ people$/km^2$, $D^{(max)} = 1000$ people$/km^2$, $\delta = 10$ people$/km^2$, $\ell = 3\ km$, $A^* = 50\ km^2$ and $H^* = 0.50$.

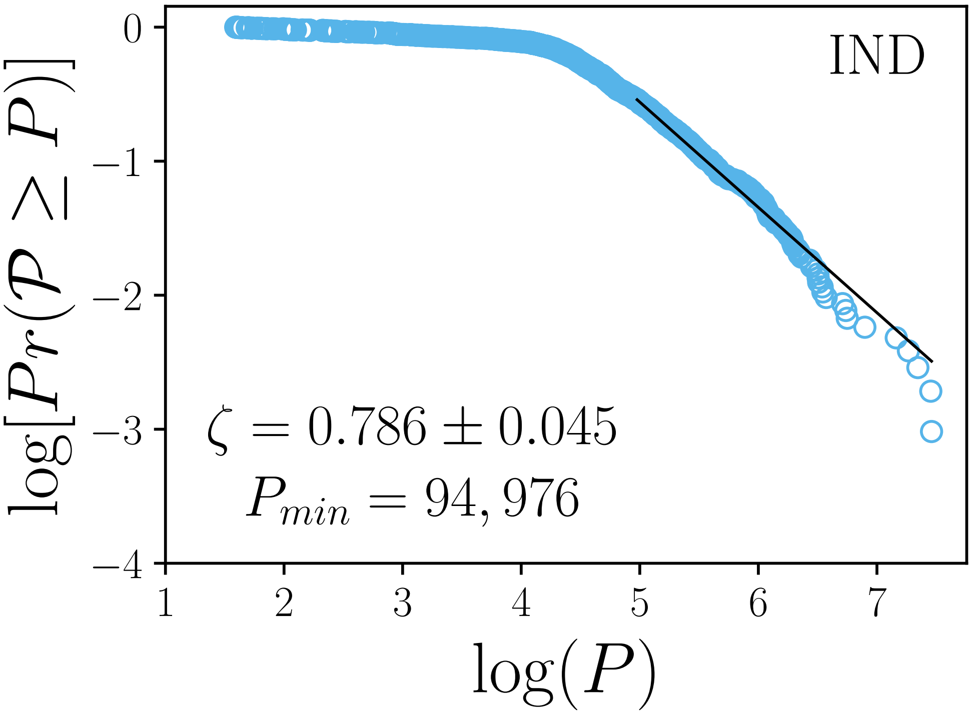

FIG. 65. **(Color online) Cumulative Distribution Function (CDF)** $Pr(\mathcal{P} \geq P)$ **versus population** P **in log-log scale.** Cities proposed by the City Local Clustering Algorithm (CLCA) are represented by light blue circles. The solid black line is the maximum likelihood power-law fit defined by the Maximum Likelihood Estimator (MLE) [50]. The value of the lower bound P_{min} and the exponent ζ are also shown. The CLCA parameters used were $D^{(min)} = 100$ people$/km^2$, $D^{(max)} = 1000$ people$/km^2$, $\delta = 10$ people$/km^2$, $\ell = 3\ km$, $A^* = 50\ km^2$ and $H^* = 0.50$.

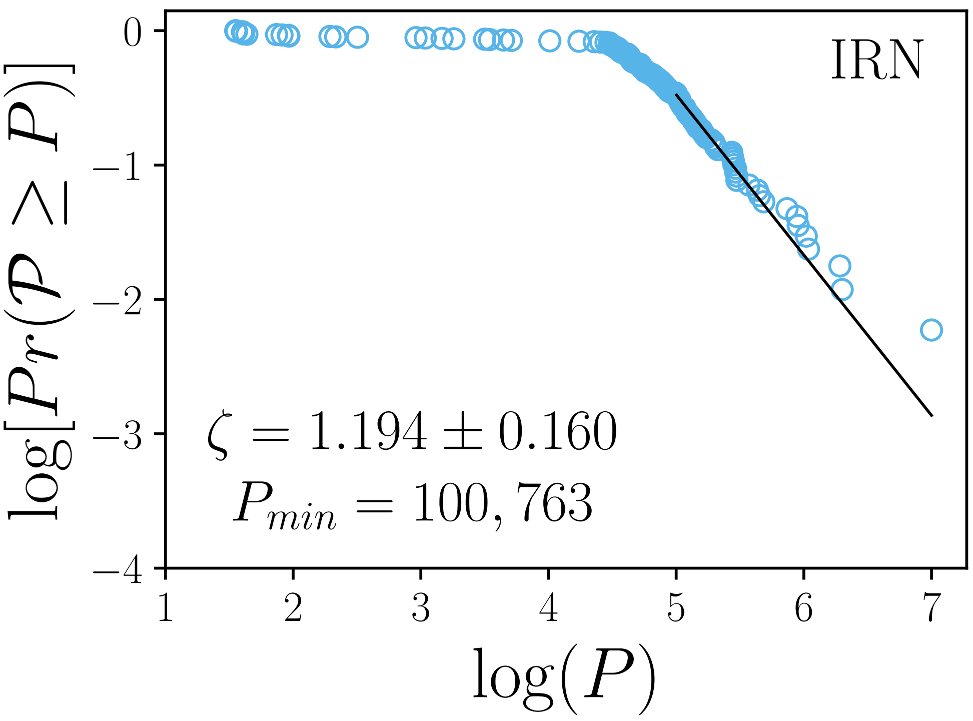

FIG. 66. **(Color online) Cumulative Distribution Function (CDF)** $Pr(\mathcal{P} \geq P)$ **versus population** P **in log-log scale.** Cities proposed by the City Local Clustering Algorithm (CLCA) are represented by light blue circles. The solid black line is the maximum likelihood power-law fit defined by the Maximum Likelihood Estimator (MLE) [50]. The value of the lower bound P_{min} and the exponent ζ are also shown. The CLCA parameters used were $D^{(min)} = 100$ people/km^2, $D^{(max)} = 1000$ people/km^2, $\delta = 10$ people/km^2, $\ell = 3$ km, $A^* = 50$ km^2 and $H^* = 0.50$.

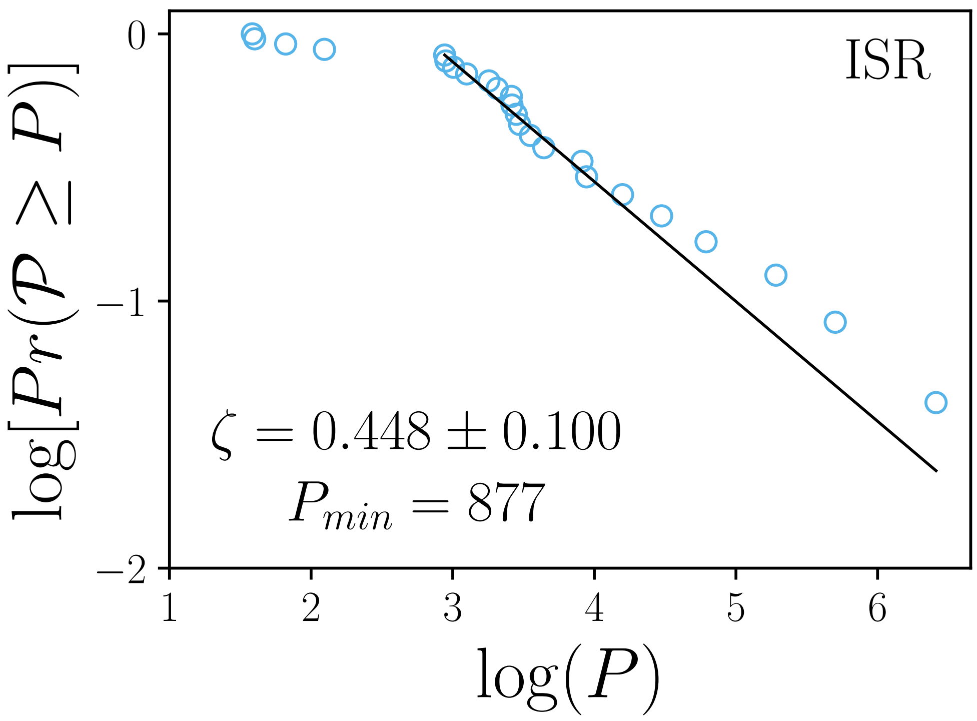

FIG. 67. **(Color online) Cumulative Distribution Function (CDF)** $Pr(\mathcal{P} \geq P)$ **versus population** P **in log-log scale.** Cities proposed by the City Local Clustering Algorithm (CLCA) are represented by light blue circles. The solid black line is the maximum likelihood power-law fit defined by the Maximum Likelihood Estimator (MLE) [50]. The value of the lower bound P_{min} and the exponent ζ are also shown. The CLCA parameters used were $D^{(min)} = 100$ people/km^2, $D^{(max)} = 1000$ people/km^2, $\delta = 10$ people/km^2, $\ell = 3$ km, $A^* = 50$ km^2 and $H^* = 0.50$.

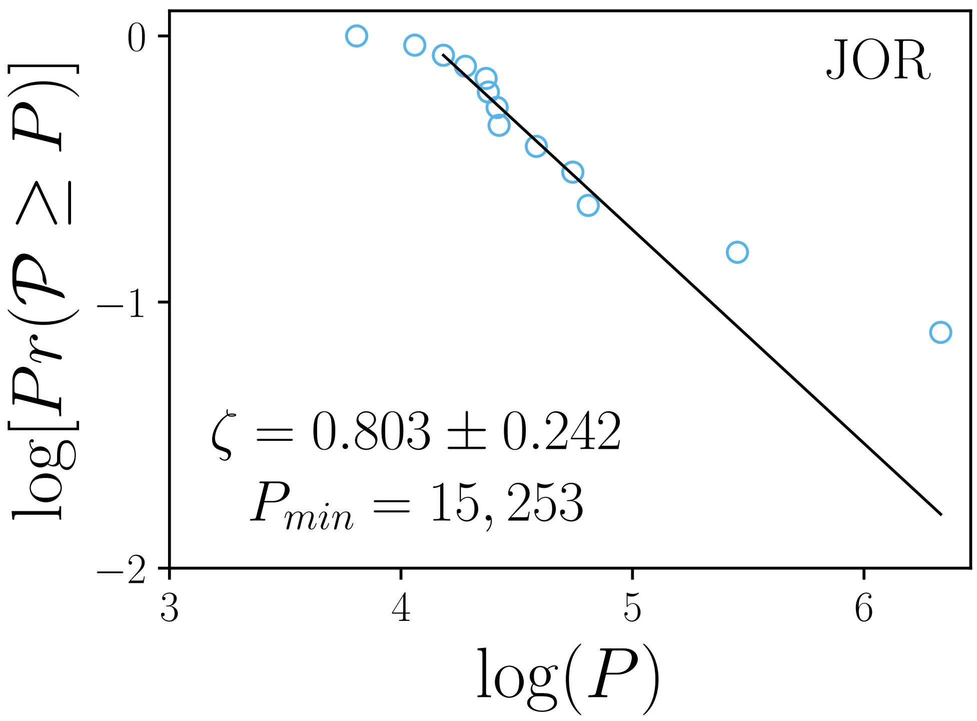

FIG. 68. **(Color online) Cumulative Distribution Function (CDF)** $Pr(\mathcal{P} \geq P)$ **versus population** P **in log-log scale.** Cities proposed by the City Local Clustering Algorithm (CLCA) are represented by light blue circles. The solid black line is the maximum likelihood power-law fit defined by the Maximum Likelihood Estimator (MLE) [50]. The value of the lower bound P_{min} and the exponent ζ are also shown. The CLCA parameters used were $D^{(min)} = 100$ people/km^2, $D^{(max)} = 1000$ people/km^2, $\delta = 10$ people/km^2, $\ell = 3$ km, $A^* = 50$ km^2 and $H^* = 0.50$.

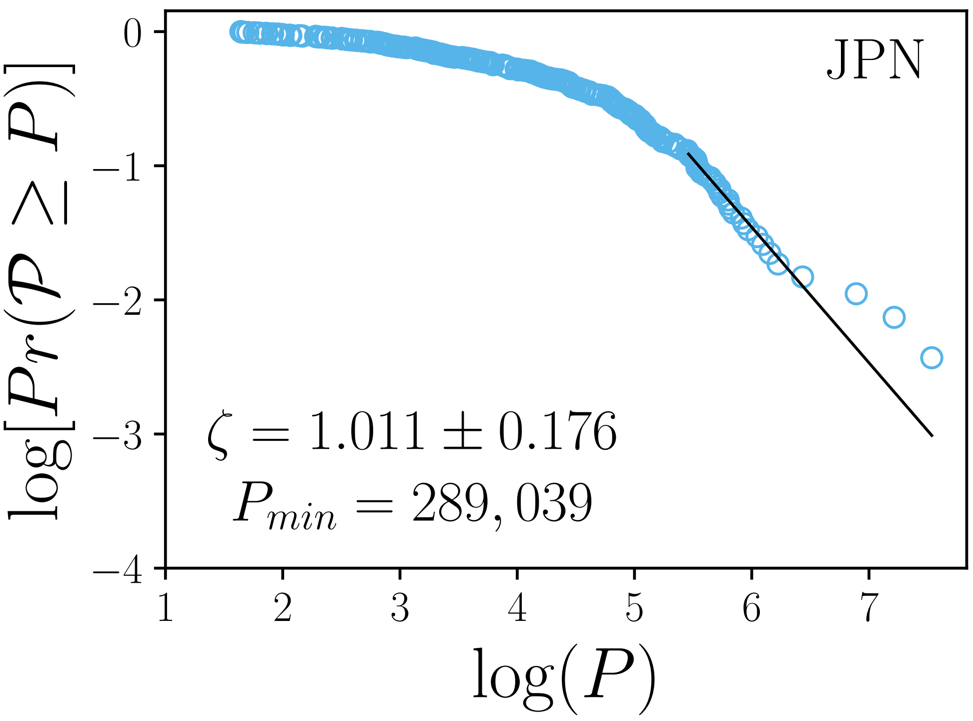

FIG. 69. **(Color online) Cumulative Distribution Function (CDF)** $Pr(\mathcal{P} \geq P)$ **versus population** P **in log-log scale.** Cities proposed by the City Local Clustering Algorithm (CLCA) are represented by light blue circles. The solid black line is the maximum likelihood power-law fit defined by the Maximum Likelihood Estimator (MLE) [50]. The value of the lower bound P_{min} and the exponent ζ are also shown. The CLCA parameters used were $D^{(min)} = 100$ people/km^2, $D^{(max)} = 1000$ people/km^2, $\delta = 10$ people/km^2, $\ell = 3$ km, $A^* = 50$ km^2 and $H^* = 0.50$.

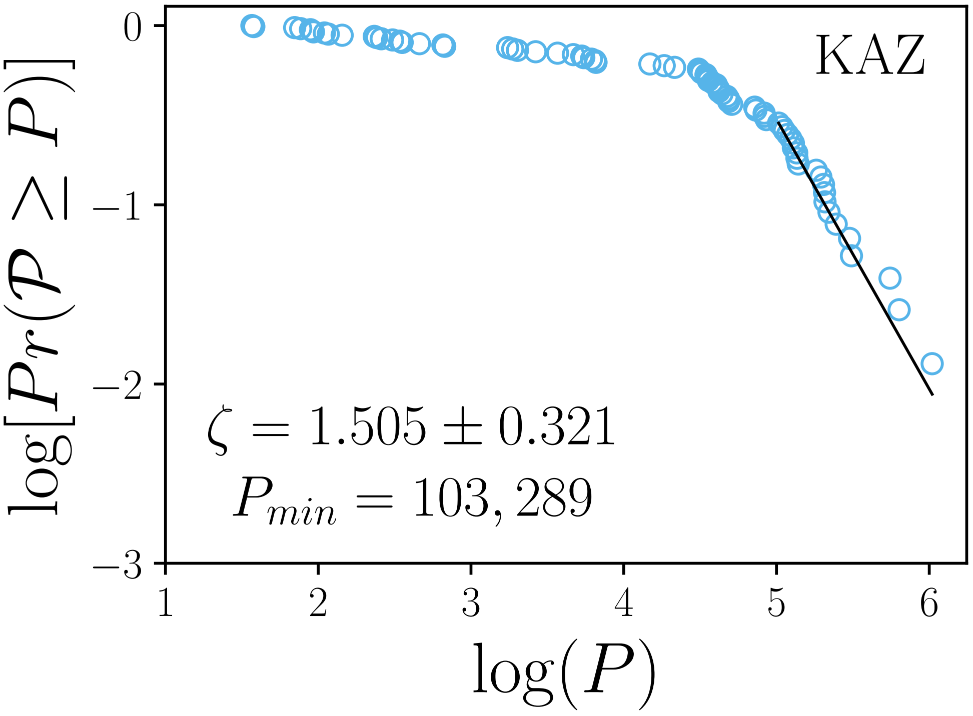

FIG. 70. **(Color online) Cumulative Distribution Function (CDF)** $Pr(\mathcal{P} \geq P)$ **versus population** P **in log-log scale.** Cities proposed by the City Local Clustering Algorithm (CLCA) are represented by light blue circles. The solid black line is the maximum likelihood power-law fit defined by the Maximum Likelihood Estimator (MLE) [50]. The value of the lower bound P_{min} and the exponent ζ are also shown. The CLCA parameters used were $D^{(min)} = 100$ people$/km^2$, $D^{(max)} = 1000$ people$/km^2$, $\delta = 10$ people$/km^2$, $\ell = 3$ km, $A^* = 50$ km^2 and $H^* = 0.50$.

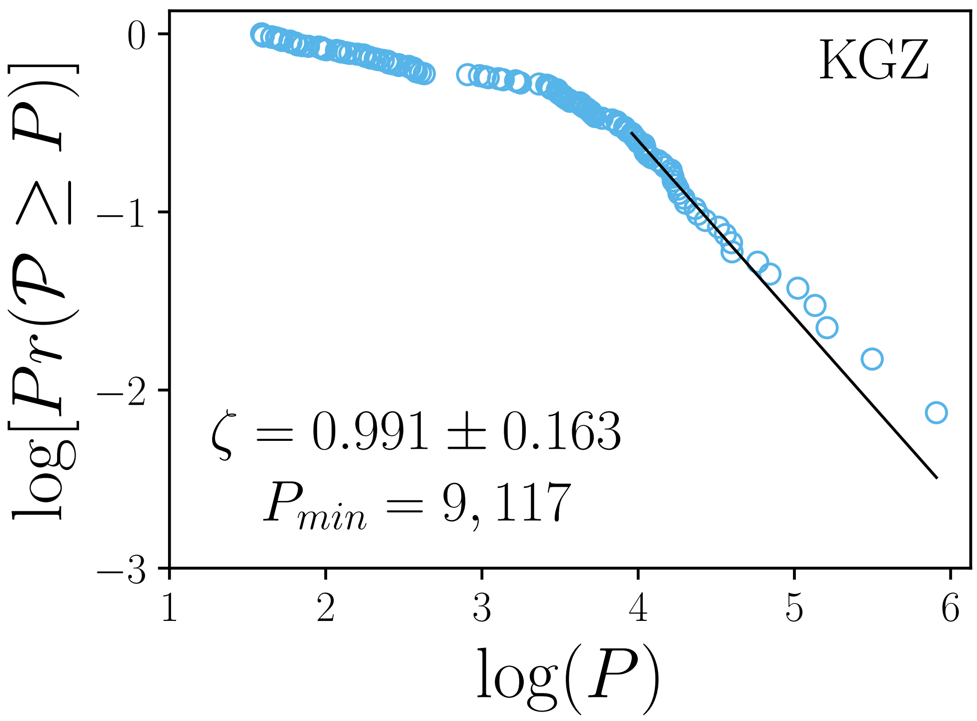

FIG. 71. **(Color online) Cumulative Distribution Function (CDF)** $Pr(\mathcal{P} \geq P)$ **versus population** P **in log-log scale.** Cities proposed by the City Local Clustering Algorithm (CLCA) are represented by light blue circles. The solid black line is the maximum likelihood power-law fit defined by the Maximum Likelihood Estimator (MLE) [50]. The value of the lower bound P_{min} and the exponent ζ are also shown. The CLCA parameters used were $D^{(min)} = 100$ people/km^2, $D^{(max)} = 1000$ people/km^2, $\delta = 10$ people/km^2, $\ell = 3$ km, $A^* = 50$ km^2 and $H^* = 0.50$.

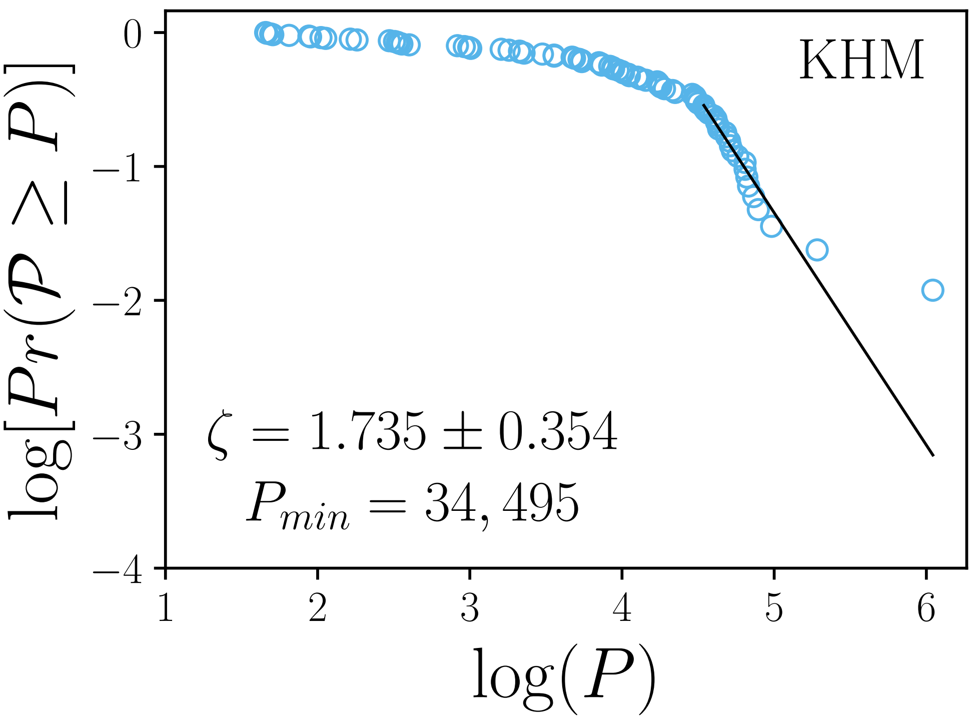

FIG. 72. **(Color online) Cumulative Distribution Function (CDF)** $Pr(\mathcal{P} \geq P)$ **versus population** P **in log-log scale.** Cities proposed by the City Local Clustering Algorithm (CLCA) are represented by light blue circles. The solid black line is the maximum likelihood power-law fit defined by the Maximum Likelihood Estimator (MLE) [50]. The value of the lower bound P_{min} and the exponent ζ are also shown. The CLCA parameters used were $D^{(min)} = 100$ people$/km^2$, $D^{(max)} = 1000$ people$/km^2$, $\delta = 10$ people$/km^2$, $\ell = 3$ km, $A^* = 50$ km^2 and $H^* = 0.50$.

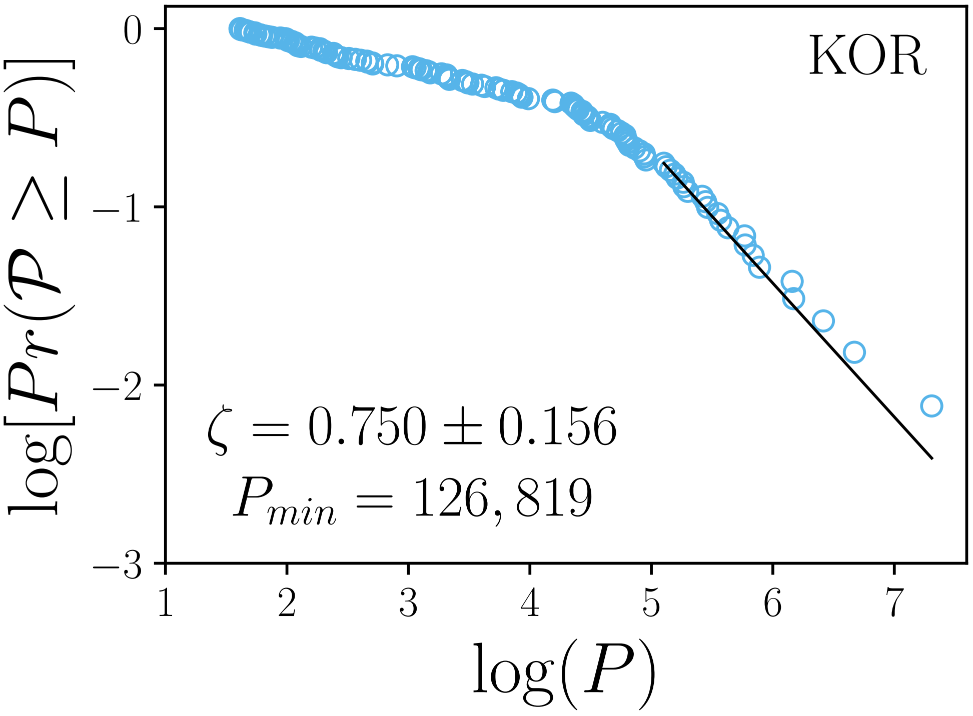

FIG. 73. **(Color online) Cumulative Distribution Function (CDF)** $Pr(\mathcal{P} \geq P)$ **versus population** P **in log-log scale.** Cities proposed by the City Local Clustering Algorithm (CLCA) are represented by light blue circles. The solid black line is the maximum likelihood power-law fit defined by the Maximum Likelihood Estimator (MLE) [50]. The value of the lower bound P_{min} and the exponent ζ are also shown. The CLCA parameters used were $D^{(min)} = 100$ people/km^2, $D^{(max)} = 1000$ people/km^2, $\delta = 10$ people/km^2, $\ell = 3$ km, $A^* = 50$ km^2 and $H^* = 0.50$.

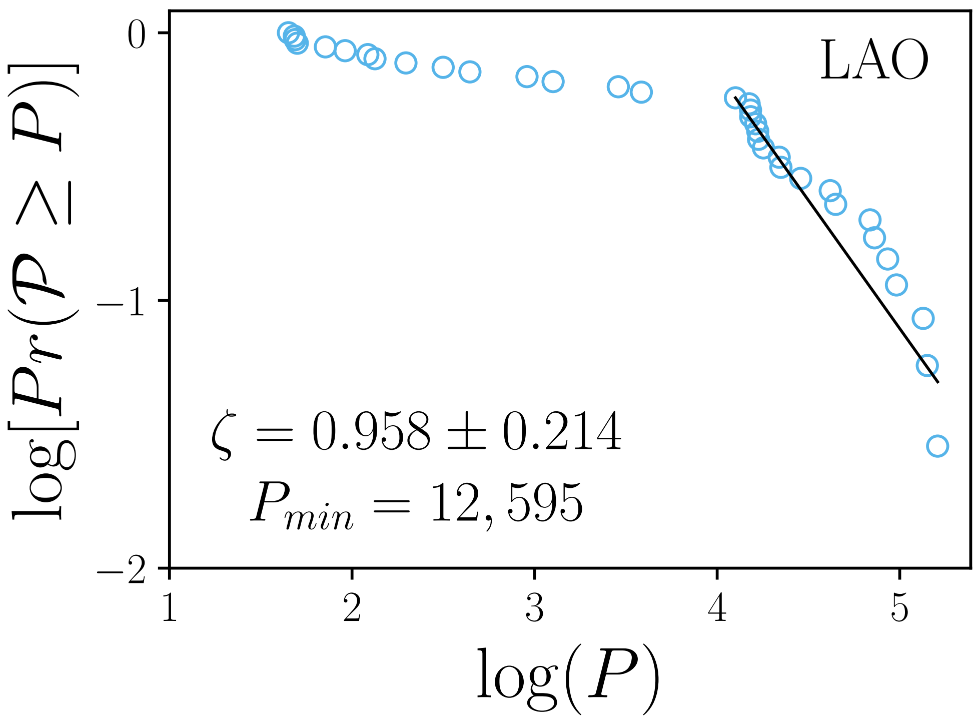

FIG. 74. **(Color online) Cumulative Distribution Function (CDF)** $Pr(\mathcal{P} \geq P)$ **versus population** P **in log-log scale.** Cities proposed by the City Local Clustering Algorithm (CLCA) are represented by light blue circles. The solid black line is the maximum likelihood power-law fit defined by the Maximum Likelihood Estimator (MLE) [50]. The value of the lower bound P_{min} and the exponent ζ are also shown. The CLCA parameters used were $D^{(min)} = 100$ people$/km^2$, $D^{(max)} = 1000$ people$/km^2$, $\delta = 10$ people$/km^2$, $\ell = 3$ km, $A^* = 50$ km^2 and $H^* = 0.50$.

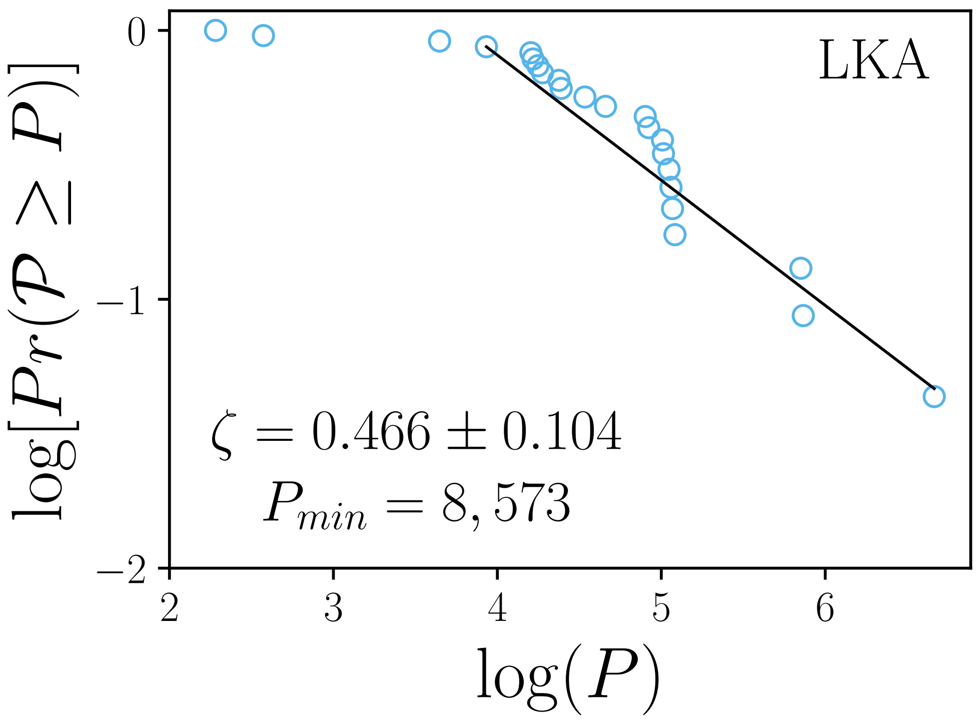

FIG. 75. **(Color online) Cumulative Distribution Function (CDF)** $Pr(\mathcal{P} \geq P)$ **versus population** P **in log-log scale.** Cities proposed by the City Local Clustering Algorithm (CLCA) are represented by light blue circles. The solid black line is the maximum likelihood power-law fit defined by the Maximum Likelihood Estimator (MLE) [50]. The value of the lower bound P_{min} and the exponent ζ are also shown. The CLCA parameters used were $D^{(min)} = 100$ people$/km^2$, $D^{(max)} = 1000$ people$/km^2$, $\delta = 10$ people$/km^2$, $\ell = 3$ km, $A^* = 50$ km^2 and $H^* = 0.50$.

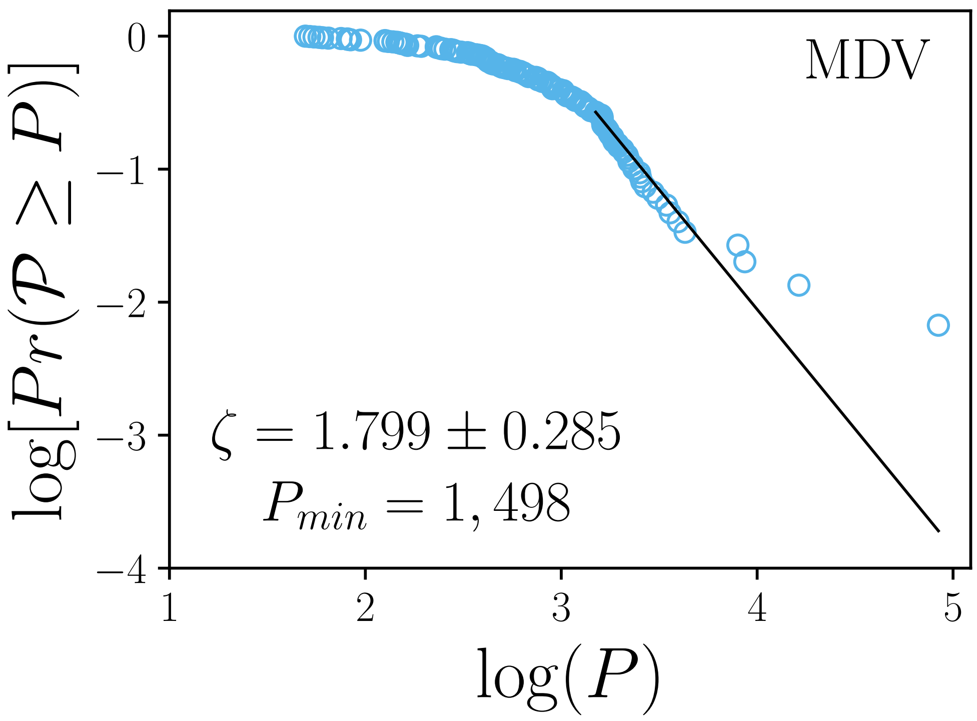

FIG. 76. **(Color online) Cumulative Distribution Function (CDF)** $Pr(\mathcal{P} \geq P)$ **versus population** P **in log-log scale.** Cities proposed by the City Local Clustering Algorithm (CLCA) are represented by light blue circles. The solid black line is the maximum likelihood power-law fit defined by the Maximum Likelihood Estimator (MLE) [50]. The value of the lower bound P_{min} and the exponent ζ are also shown. The CLCA parameters used were $D^{(min)} = 100$ people/km^2, $D^{(max)} = 1000$ people/km^2, $\delta = 10$ people/km^2, $\ell = 3$ km, $A^* = 50$ km^2 and $H^* = 0.50$.

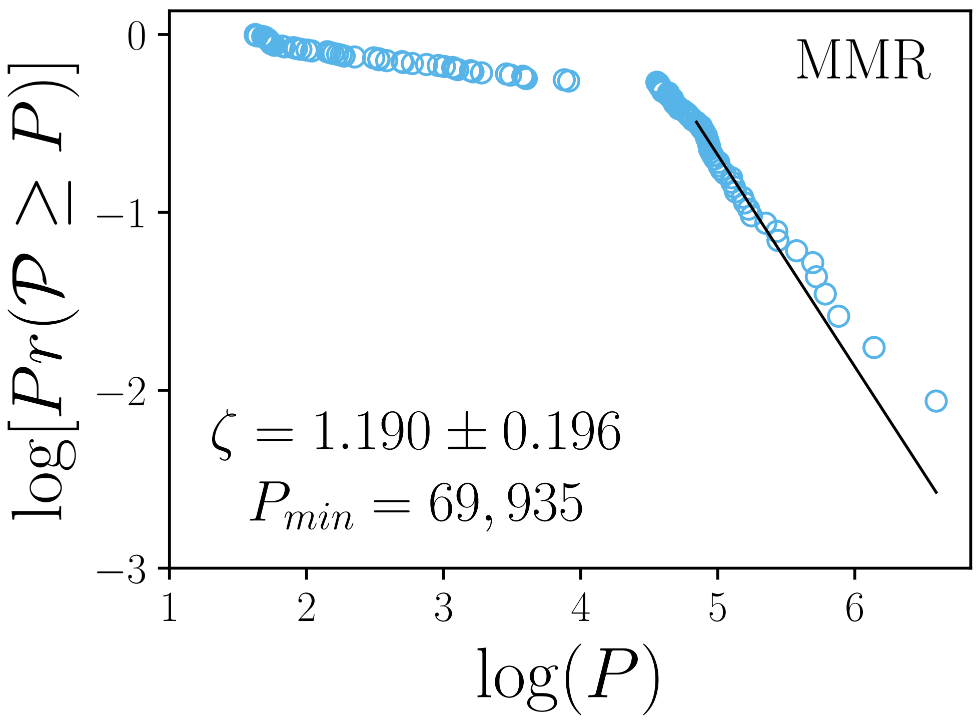

FIG. 77. **(Color online) Cumulative Distribution Function (CDF)** $Pr(\mathcal{P} \geq P)$ **versus population** P **in log-log scale.** Cities proposed by the City Local Clustering Algorithm (CLCA) are represented by light blue circles. The solid black line is the maximum likelihood power-law fit defined by the Maximum Likelihood Estimator (MLE) [50]. The value of the lower bound P_{min} and the exponent ζ are also shown. The CLCA parameters used were $D^{(min)} = 100$ people/km^2, $D^{(max)} = 1000$ people/km^2, $\delta = 10$ people/km^2, $\ell = 3$ km, $A^* = 50$ km^2 and $H^* = 0.50$.

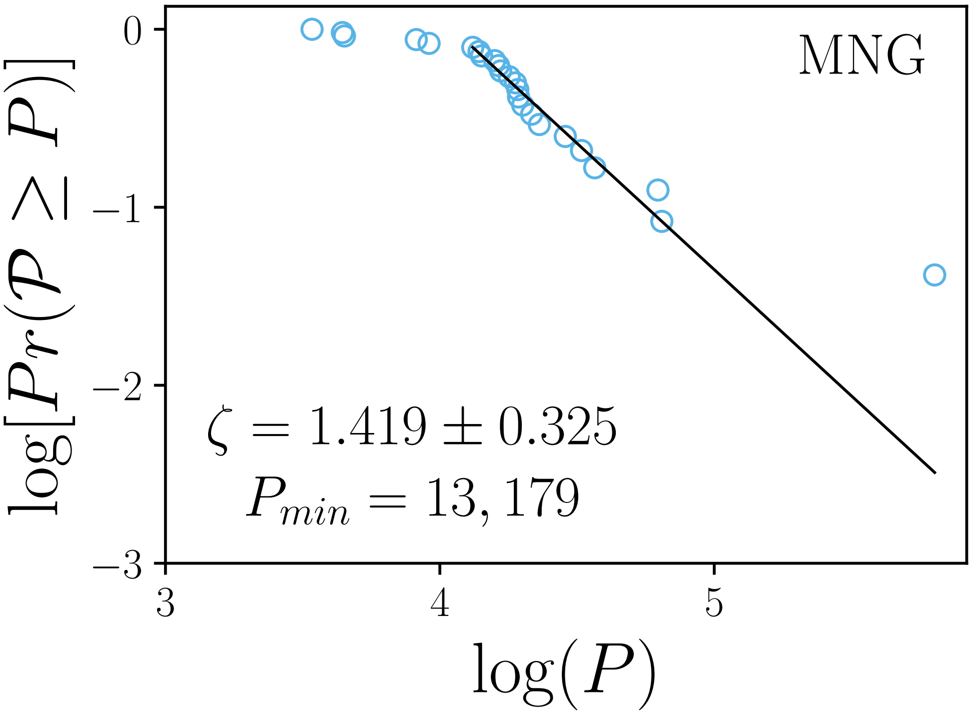

FIG. 78. **(Color online) Cumulative Distribution Function (CDF)** $Pr(\mathcal{P} \geq P)$ **versus population** P **in log-log scale.** Cities proposed by the City Local Clustering Algorithm (CLCA) are represented by light blue circles. The solid black line is the maximum likelihood power-law fit defined by the Maximum Likelihood Estimator (MLE) [50]. The value of the lower bound P_{min} and the exponent ζ are also shown. The CLCA parameters used were $D^{(min)} = 100$ people$/km^2$, $D^{(max)} = 1000$ people$/km^2$, $\delta = 10$ people$/km^2$, $\ell = 3$ km, $A^* = 50$ km^2 and $H^* = 0.50$.

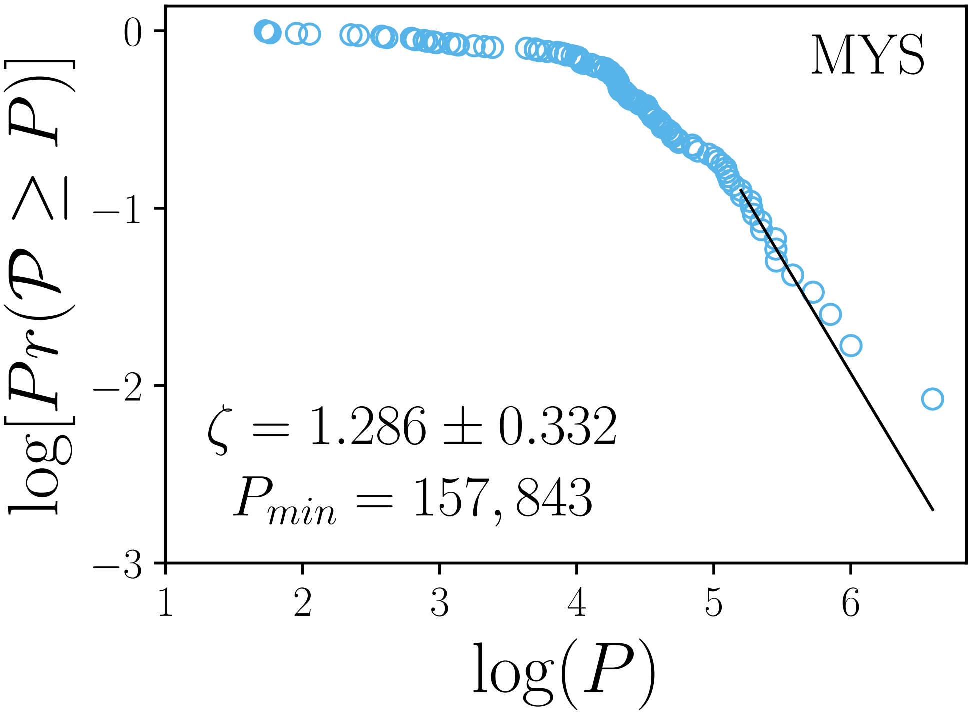

FIG. 79. **(Color online) Cumulative Distribution Function (CDF)** $Pr(\mathcal{P} \geq P)$ **versus population** P **in log-log scale.** Cities proposed by the City Local Clustering Algorithm (CLCA) are represented by light blue circles. The solid black line is the maximum likelihood power-law fit defined by the Maximum Likelihood Estimator (MLE) [50]. The value of the lower bound P_{min} and the exponent ζ are also shown. The CLCA parameters used were $D^{(min)} = 100$ people$/km^2$, $D^{(max)} = 1000$ people$/km^2$, $\delta = 10$ people$/km^2$, $\ell = 3$ km, $A^* = 50$ km^2 and $H^* = 0.50$.

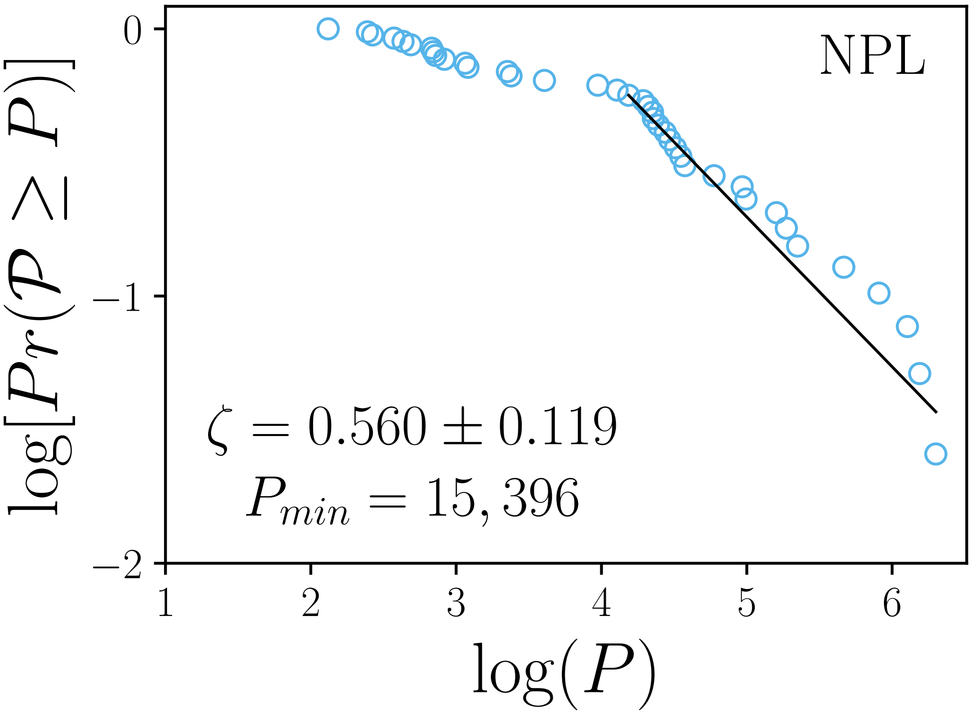

FIG. 80. **(Color online) Cumulative Distribution Function (CDF)** $Pr(\mathcal{P} \geq P)$ **versus population** P **in log-log scale.** Cities proposed by the City Local Clustering Algorithm (CLCA) are represented by light blue circles. The solid black line is the maximum likelihood power-law fit defined by the Maximum Likelihood Estimator (MLE) [50]. The value of the lower bound P_{min} and the exponent ζ are also shown. The CLCA parameters used were $D^{(min)} = 100$ people$/km^2$, $D^{(max)} = 1000$ people$/km^2$, $\delta = 10$ people$/km^2$, $\ell = 3\ km$, $A^* = 50\ km^2$ and $H^* = 0.50$.

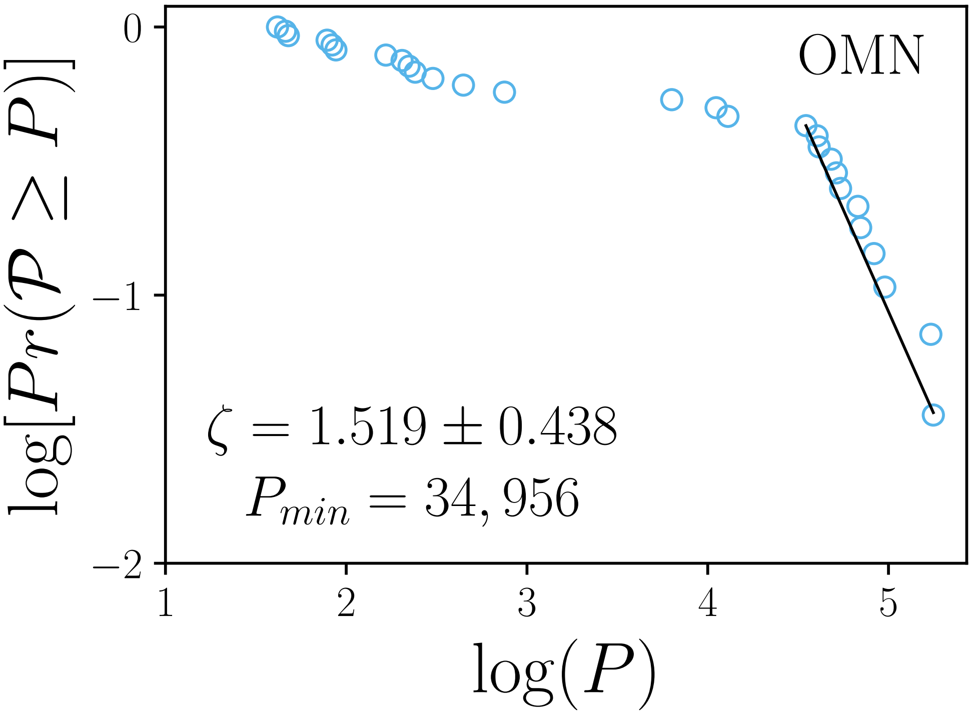

FIG. 81. **(Color online) Cumulative Distribution Function (CDF)** $Pr(\mathcal{P} \geq P)$ **versus population** P **in log-log scale.** Cities proposed by the City Local Clustering Algorithm (CLCA) are represented by light blue circles. The solid black line is the maximum likelihood power-law fit defined by the Maximum Likelihood Estimator (MLE) [50]. The value of the lower bound P_{min} and the exponent ζ are also shown. The CLCA parameters used were $D^{(min)} = 100$ people/km^2, $D^{(max)} = 1000$ people/km^2, $\delta = 10$ people/km^2, $\ell = 3$ km, $A^* = 50$ km^2 and $H^* = 0.50$.

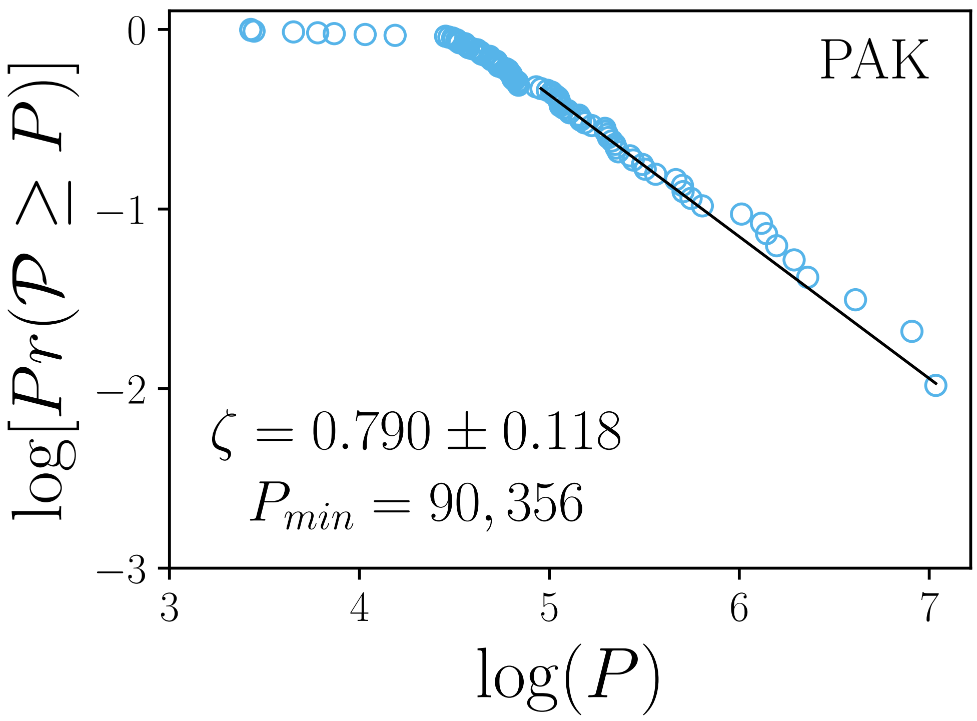

FIG. 82. **(Color online) Cumulative Distribution Function (CDF)** $Pr(\mathcal{P} \geq P)$ **versus population** P **in log-log scale.** Cities proposed by the City Local Clustering Algorithm (CLCA) are represented by light blue circles. The solid black line is the maximum likelihood power-law fit defined by the Maximum Likelihood Estimator (MLE) [50]. The value of the lower bound P_{min} and the exponent ζ are also shown. The CLCA parameters used were $D^{(min)} = 100$ people/km^2, $D^{(max)} = 1000$ people/km^2, $\delta = 10$ people/km^2, $\ell = 3$ km, $A^* = 50$ km^2 and $H^* = 0.50$.

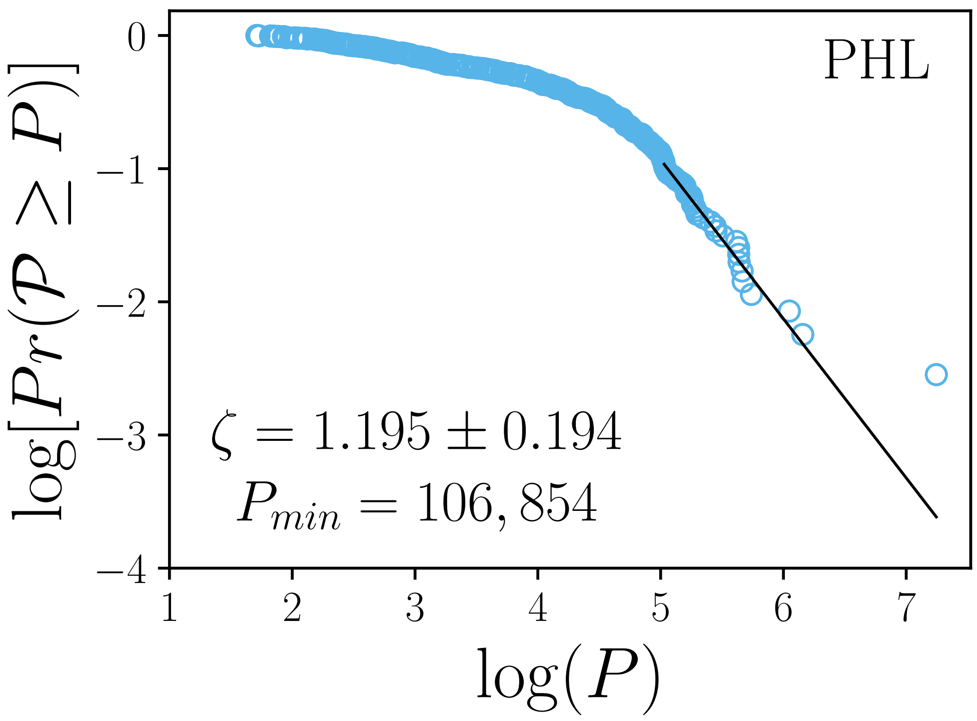

FIG. 83. **(Color online) Cumulative Distribution Function (CDF)** $Pr(\mathcal{P} \geq P)$ **versus population** P **in log-log scale.** Cities proposed by the City Local Clustering Algorithm (CLCA) are represented by light blue circles. The solid black line is the maximum likelihood power-law fit defined by the Maximum Likelihood Estimator (MLE) [50]. The value of the lower bound P_{min} and the exponent ζ are also shown. The CLCA parameters used were $D^{(min)} = 100$ people$/km^2$, $D^{(max)} = 1000$ people$/km^2$, $\delta = 10$ people$/km^2$, $\ell = 3\ km$, $A^* = 50\ km^2$ and $H^* = 0.50$.

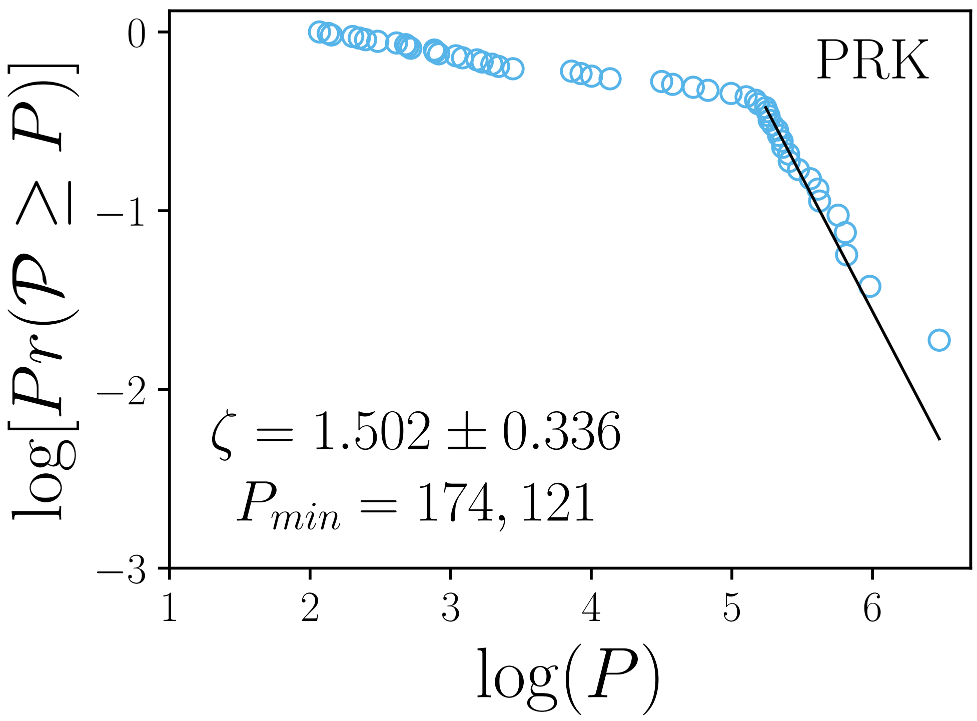

FIG. 84. **(Color online) Cumulative Distribution Function (CDF)** $Pr(\mathcal{P} \geq P)$ **versus population** P **in log-log scale.** Cities proposed by the City Local Clustering Algorithm (CLCA) are represented by light blue circles. The solid black line is the maximum likelihood power-law fit defined by the Maximum Likelihood Estimator (MLE) [50]. The value of the lower bound P_{min} and the exponent ζ are also shown. The CLCA parameters used were $D^{(min)} = 100$ people/km^2, $D^{(max)} = 1000$ people/km^2, $\delta = 10$ people/km^2, $\ell = 3$ km, $A^* = 50$ km^2 and $H^* = 0.50$.

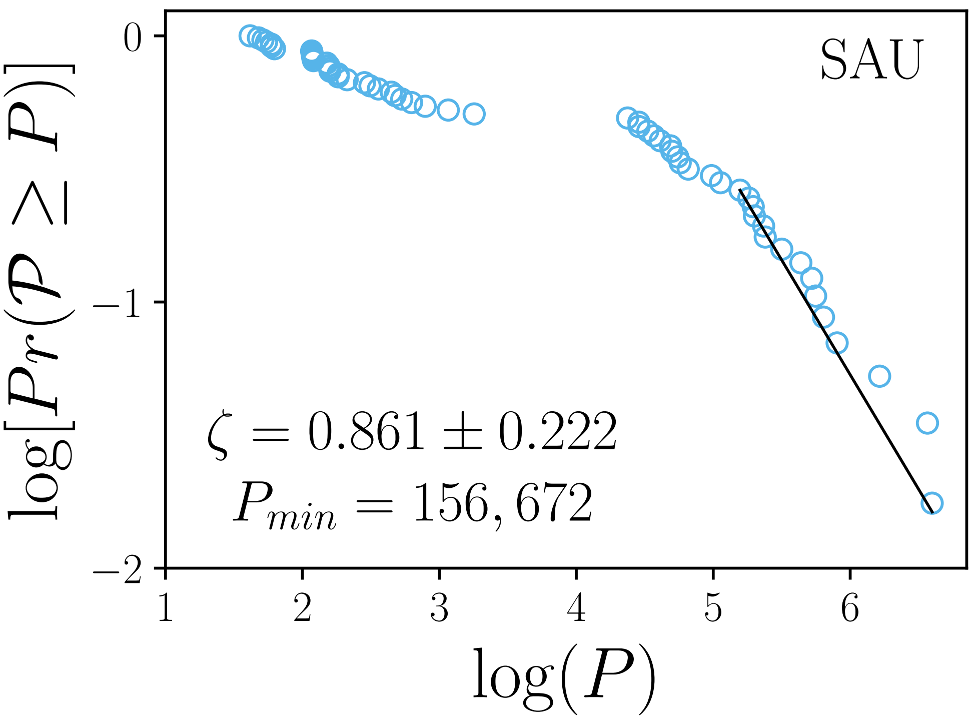

FIG. 85. **(Color online) Cumulative Distribution Function (CDF)** $Pr(\mathcal{P} \geq P)$ **versus population** P **in log-log scale.** Cities proposed by the City Local Clustering Algorithm (CLCA) are represented by light blue circles. The solid black line is the maximum likelihood power-law fit defined by the Maximum Likelihood Estimator (MLE) [50]. The value of the lower bound P_{min} and the exponent ζ are also shown. The CLCA parameters used were $D^{(min)} = 100$ people/km^2, $D^{(max)} = 1000$ people/km^2, $\delta = 10$ people/km^2, $\ell = 3$ km, $A^* = 50$ km^2 and $H^* = 0.50$.

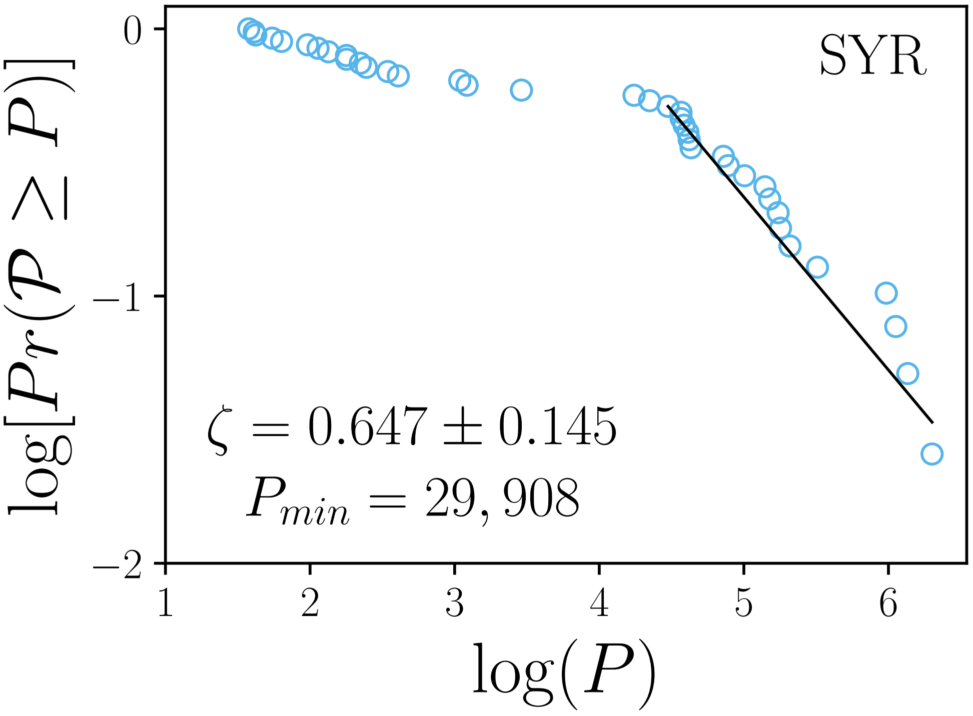

FIG. 86. **(Color online) Cumulative Distribution Function (CDF)** $Pr(\mathcal{P} \geq P)$ **versus population** P **in log-log scale.** Cities proposed by the City Local Clustering Algorithm (CLCA) are represented by light blue circles. The solid black line is the maximum likelihood power-law fit defined by the Maximum Likelihood Estimator (MLE) [50]. The value of the lower bound P_{min} and the exponent ζ are also shown. The CLCA parameters used were $D^{(min)} = 100$ people/km^2, $D^{(max)} = 1000$ people/km^2, $\delta = 10$ people/km^2, $\ell = 3$ km, $A^* = 50$ km^2 and $H^* = 0.50$.

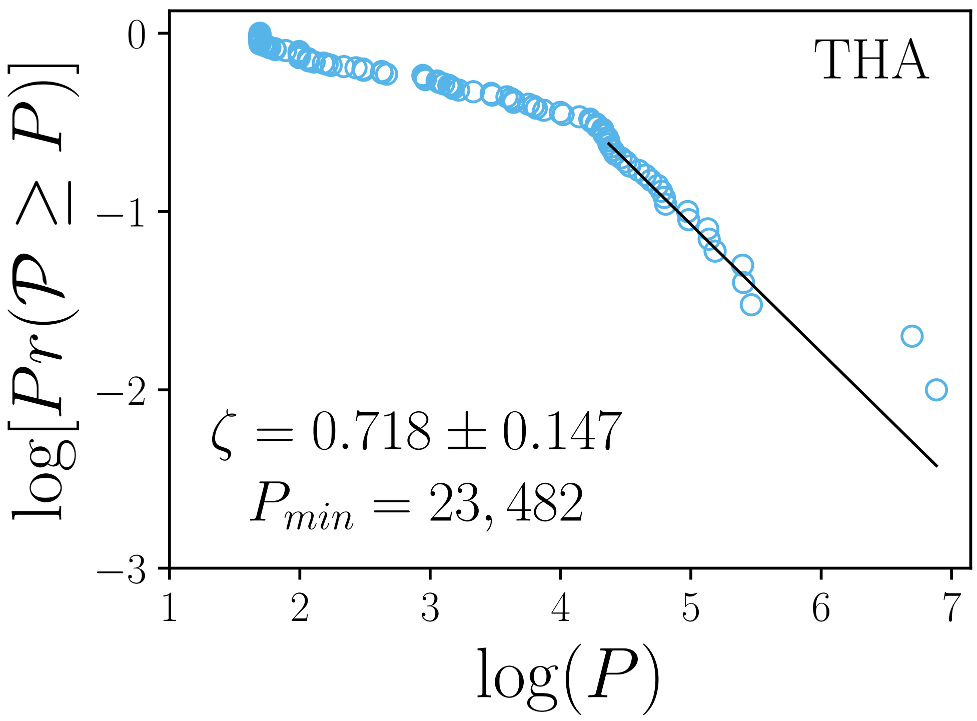

FIG. 87. **(Color online) Cumulative Distribution Function (CDF)** $Pr(\mathcal{P} \geq P)$ **versus population** P **in log-log scale.** Cities proposed by the City Local Clustering Algorithm (CLCA) are represented by light blue circles. The solid black line is the maximum likelihood power-law fit defined by the Maximum Likelihood Estimator (MLE) [50]. The value of the lower bound P_{min} and the exponent ζ are also shown. The CLCA parameters used were $D^{(min)} = 100$ people/km^2, $D^{(max)} = 1000$ people/km^2, $\delta = 10$ people/km^2, $\ell = 3$ km, $A^* = 50$ km^2 and $H^* = 0.50$.

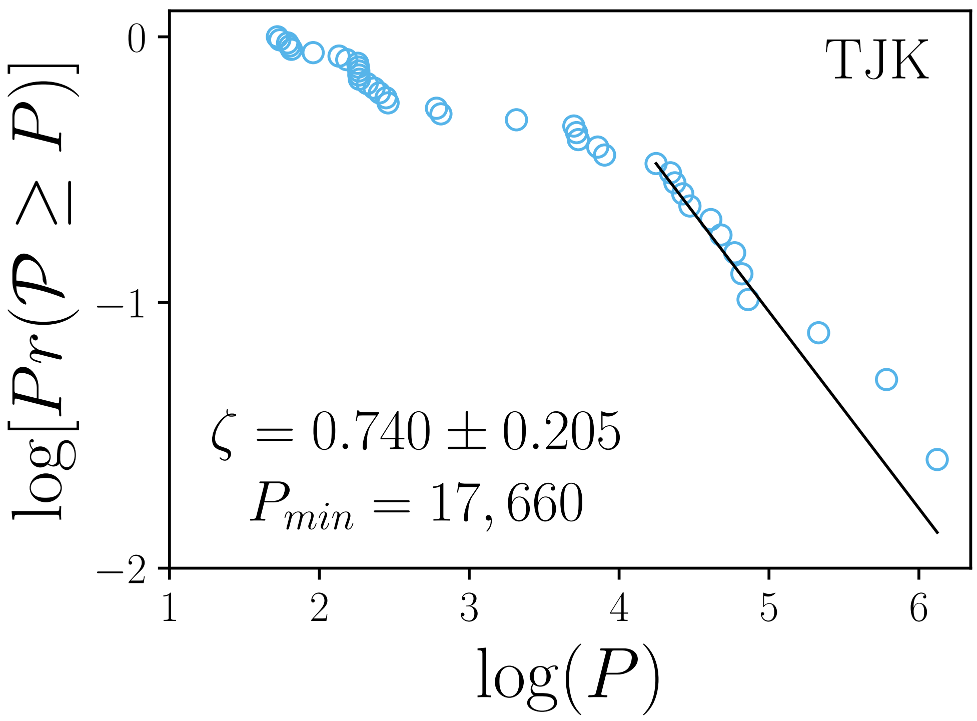

FIG. 88. **(Color online) Cumulative Distribution Function (CDF)** $Pr(\mathcal{P} \geq P)$ **versus population** P **in log-log scale.** Cities proposed by the City Local Clustering Algorithm (CLCA) are represented by light blue circles. The solid black line is the maximum likelihood power-law fit defined by the Maximum Likelihood Estimator (MLE) [50]. The value of the lower bound P_{min} and the exponent ζ are also shown. The CLCA parameters used were $D^{(min)} = 100$ people$/km^2$, $D^{(max)} = 1000$ people$/km^2$, $\delta = 10$ people$/km^2$, $\ell = 3$ km, $A^* = 50$ km^2 and $H^* = 0.50$.

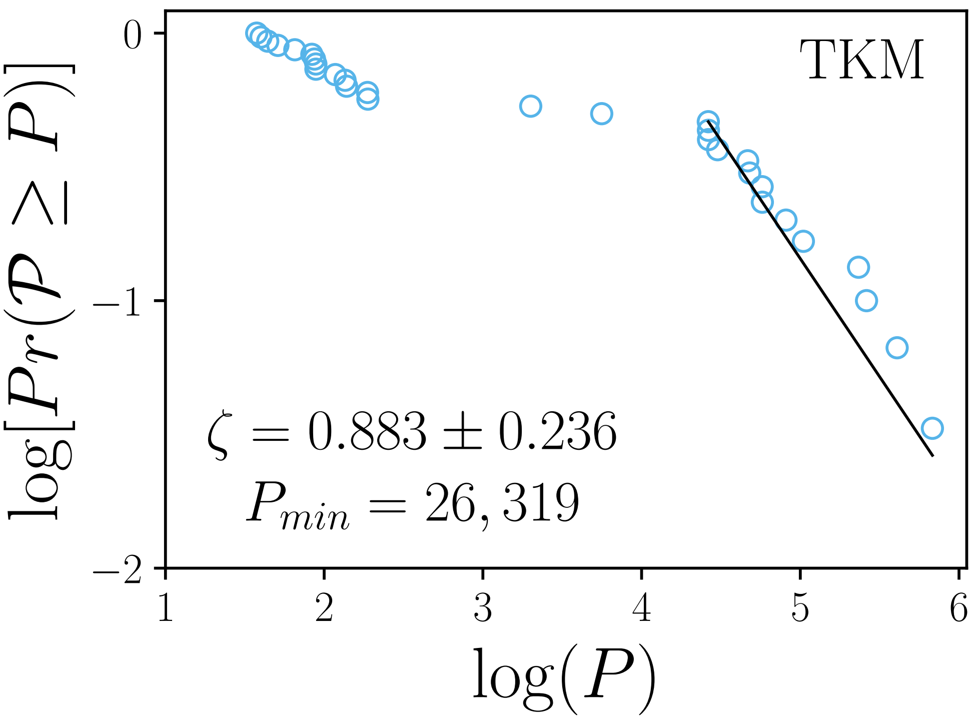

FIG. 89. **(Color online) Cumulative Distribution Function (CDF)** $Pr(\mathcal{P} \geq P)$ **versus population** P **in log-log scale.** Cities proposed by the City Local Clustering Algorithm (CLCA) are represented by light blue circles. The solid black line is the maximum likelihood power-law fit defined by the Maximum Likelihood Estimator (MLE) [50]. The value of the lower bound P_{min} and the exponent ζ are also shown. The CLCA parameters used were $D^{(min)} = 100$ people/km^2, $D^{(max)} = 1000$ people/km^2, $\delta = 10$ people/km^2, $\ell = 3$ km, $A^* = 50$ km^2 and $H^* = 0.50$.

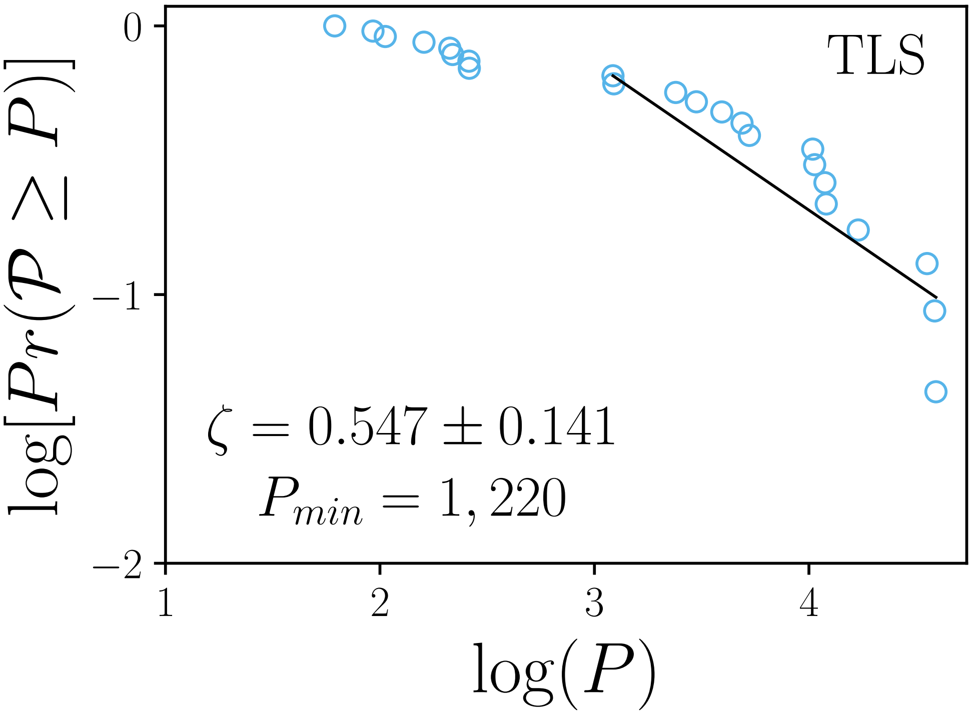

FIG. 90. **(Color online) Cumulative Distribution Function (CDF)** $Pr(\mathcal{P} \geq P)$ **versus population** P **in log-log scale.** Cities proposed by the City Local Clustering Algorithm (CLCA) are represented by light blue circles. The solid black line is the maximum likelihood power-law fit defined by the Maximum Likelihood Estimator (MLE) [50]. The value of the lower bound P_{min} and the exponent ζ are also shown. The CLCA parameters used were $D^{(min)} = 100$ people$/km^2$, $D^{(max)} = 1000$ people$/km^2$, $\delta = 10$ people$/km^2$, $\ell = 3$ km, $A^* = 50$ km^2 and $H^* = 0.50$.

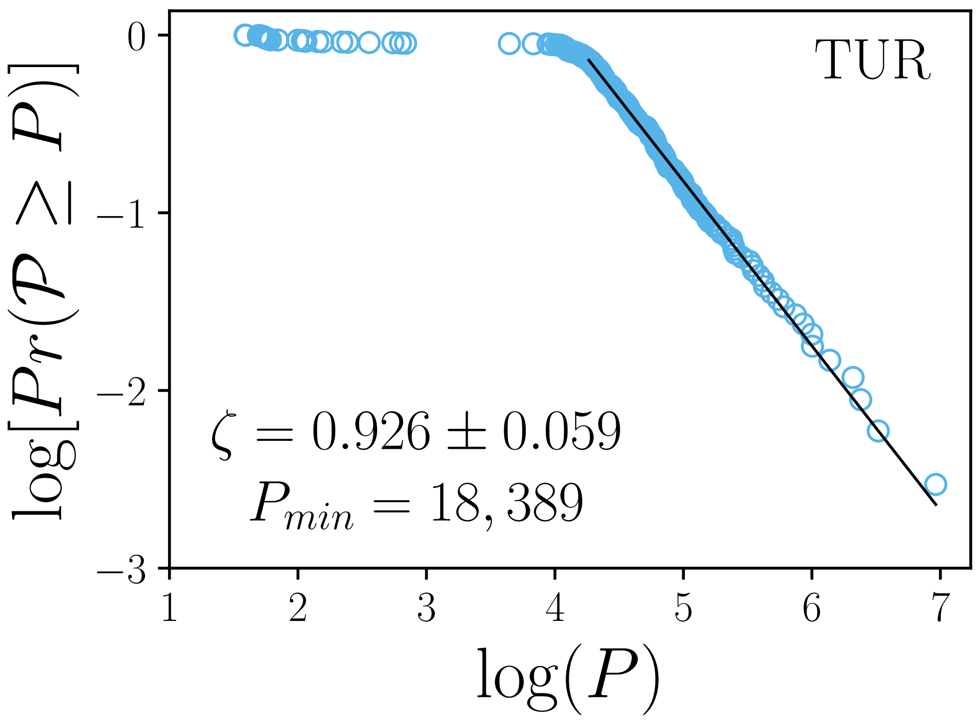

FIG. 91. **(Color online) Cumulative Distribution Function (CDF)** $Pr(\mathcal{P} \geq P)$ **versus population** P **in log-log scale.** Cities proposed by the City Local Clustering Algorithm (CLCA) are represented by light blue circles. The solid black line is the maximum likelihood power-law fit defined by the Maximum Likelihood Estimator (MLE) [50]. The value of the lower bound P_{min} and the exponent ζ are also shown. The CLCA parameters used were $D^{(min)} = 100$ people/km^2, $D^{(max)} = 1000$ people/km^2, $\delta = 10$ people/km^2, $\ell = 3$ km, $A^* = 50$ km^2 and $H^* = 0.50$.

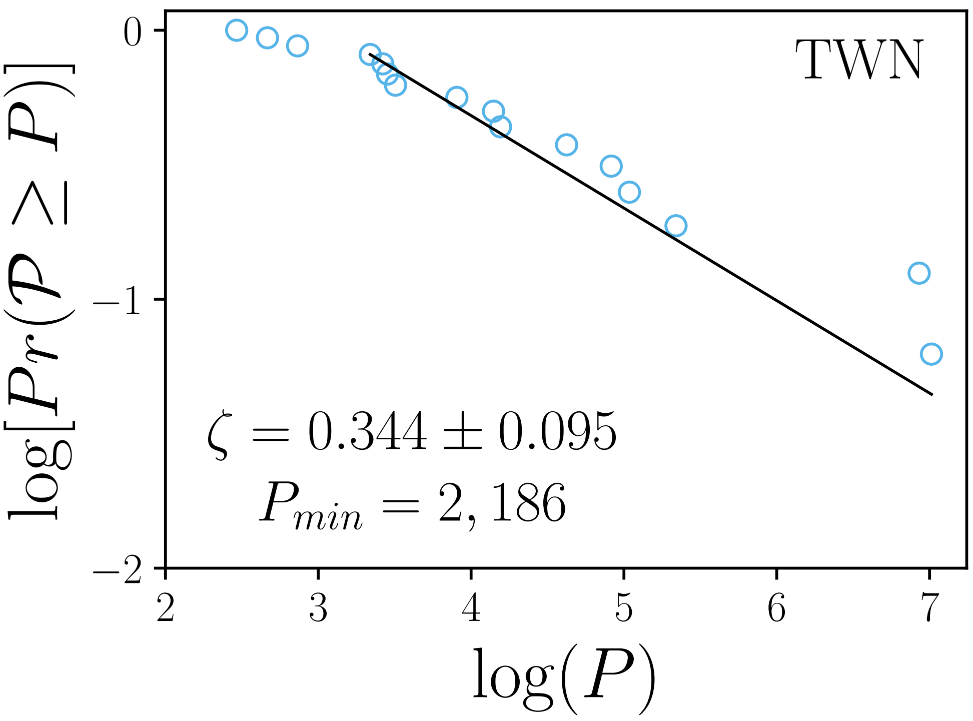

FIG. 92. **(Color online) Cumulative Distribution Function (CDF)** $Pr(\mathcal{P} \geq P)$ **versus population** P **in log-log scale.** Cities proposed by the City Local Clustering Algorithm (CLCA) are represented by light blue circles. The solid black line is the maximum likelihood power-law fit defined by the Maximum Likelihood Estimator (MLE) [50]. The value of the lower bound P_{min} and the exponent ζ are also shown. The CLCA parameters used were $D^{(min)} = 100$ people/km^2, $D^{(max)} = 1000$ people/km^2, $\delta = 10$ people/km^2, $\ell = 3$ km, $A^* = 50$ km^2 and $H^* = 0.50$.

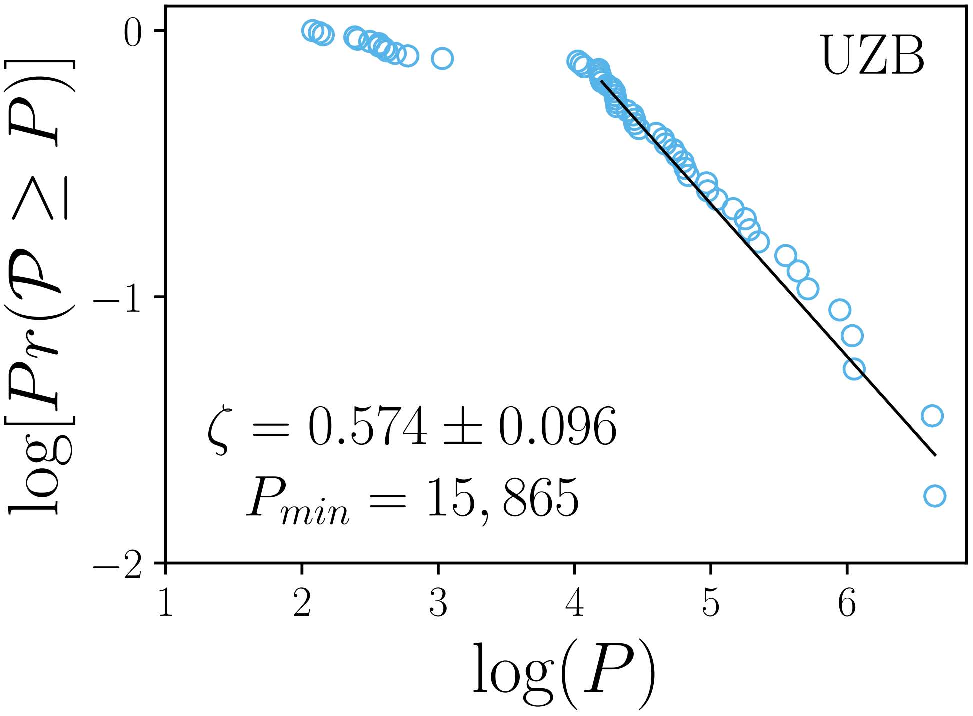

FIG. 93. **(Color online) Cumulative Distribution Function (CDF)** $Pr(\mathcal{P} \geq P)$ **versus population** P **in log-log scale.** Cities proposed by the City Local Clustering Algorithm (CLCA) are represented by light blue circles. The solid black line is the maximum likelihood power-law fit defined by the Maximum Likelihood Estimator (MLE) [50]. The value of the lower bound P_{min} and the exponent ζ are also shown. The CLCA parameters used were $D^{(min)} = 100$ people$/km^2$, $D^{(max)} = 1000$ people$/km^2$, $\delta = 10$ people$/km^2$, $\ell = 3$ km, $A^* = 50$ km^2 and $H^* = 0.50$.

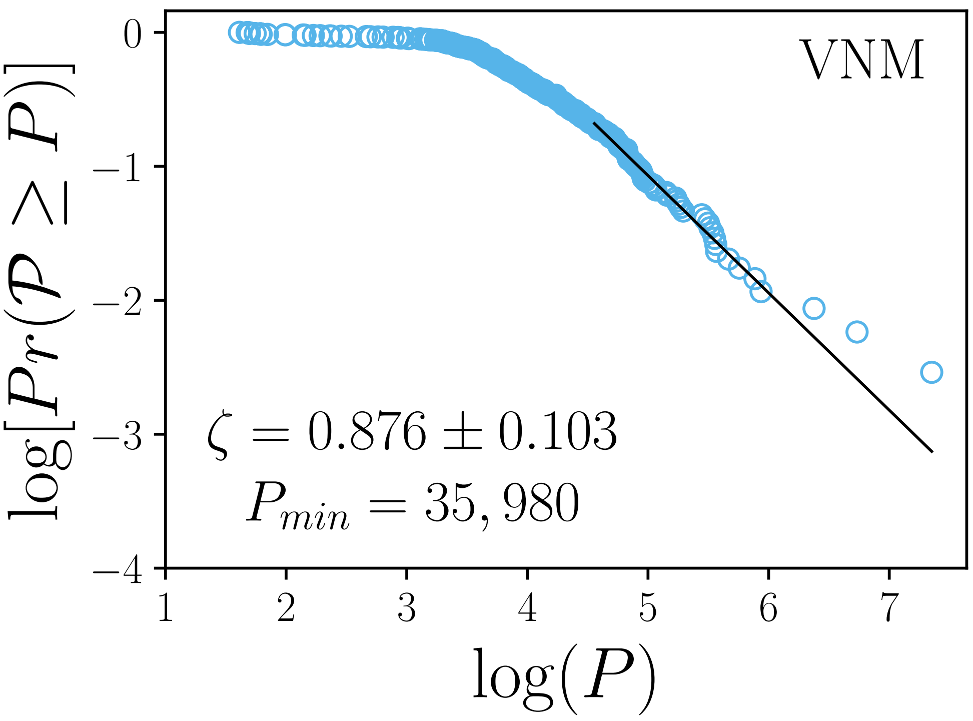

FIG. 94. **(Color online) Cumulative Distribution Function (CDF)** $Pr(\mathcal{P} \geq P)$ **versus population** P **in log-log scale.** Cities proposed by the City Local Clustering Algorithm (CLCA) are represented by light blue circles. The solid black line is the maximum likelihood power-law fit defined by the Maximum Likelihood Estimator (MLE) [50]. The value of the lower bound P_{min} and the exponent ζ are also shown. The CLCA parameters used were $D^{(min)} = 100$ people$/km^2$, $D^{(max)} = 1000$ people$/km^2$, $\delta = 10$ people$/km^2$, $\ell = 3\ km$, $A^* = 50\ km^2$ and $H^* = 0.50$.

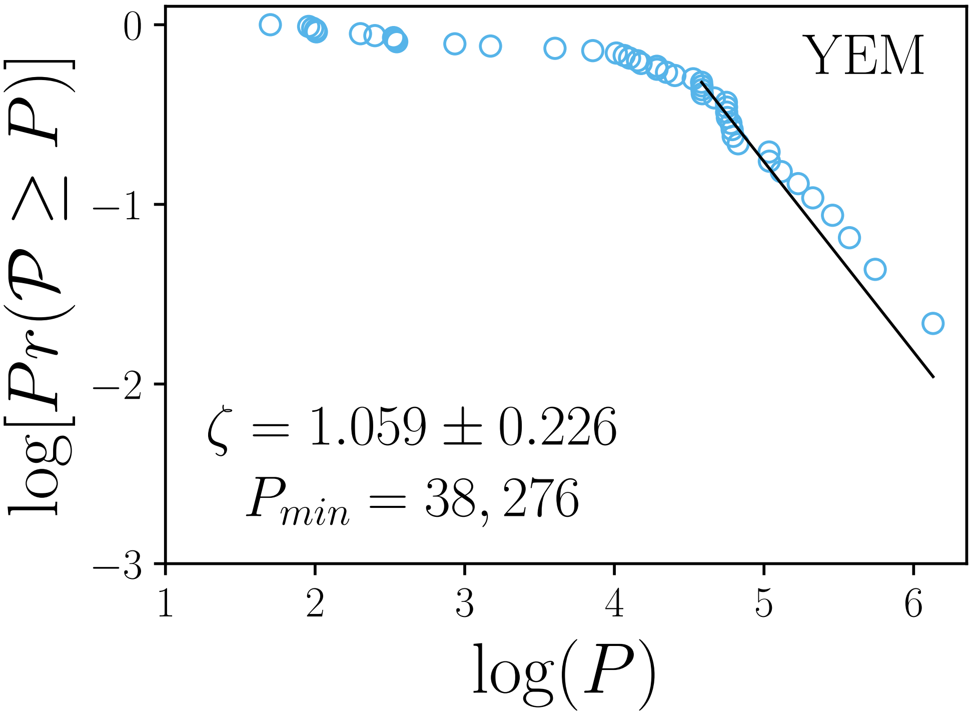

FIG. 95. **(Color online) Cumulative Distribution Function (CDF)** $Pr(\mathcal{P} \geq P)$ **versus population** P **in log-log scale.** Cities proposed by the City Local Clustering Algorithm (CLCA) are represented by light blue circles. The solid black line is the maximum likelihood power-law fit defined by the Maximum Likelihood Estimator (MLE) [50]. The value of the lower bound P_{min} and the exponent ζ are also shown. The CLCA parameters used were $D^{(min)} = 100$ people/km^2, $D^{(max)} = 1000$ people/km^2, $\delta = 10$ people/km^2, $\ell = 3$ km, $A^* = 50$ km^2 and $H^* = 0.50$.

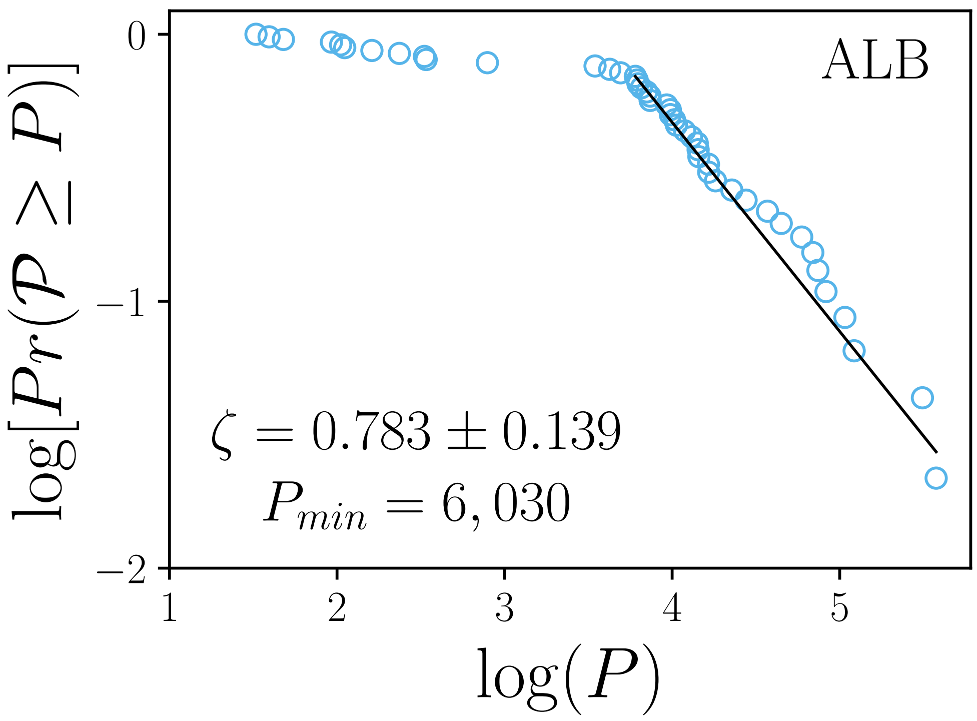

FIG. 96. **(Color online) Cumulative Distribution Function (CDF)** $Pr(\mathcal{P} \geq P)$ **versus population** P **in log-log scale.** Cities proposed by the City Local Clustering Algorithm (CLCA) are represented by light blue circles. The solid black line is the maximum likelihood power-law fit defined by the Maximum Likelihood Estimator (MLE) [50]. The value of the lower bound P_{min} and the exponent ζ are also shown. The CLCA parameters used were $D^{(min)} = 100$ people$/km^2$, $D^{(max)} = 1000$ people$/km^2$, $\delta = 10$ people$/km^2$, $\ell = 3\ km$, $A^* = 50\ km^2$ and $H^* = 0.50$.

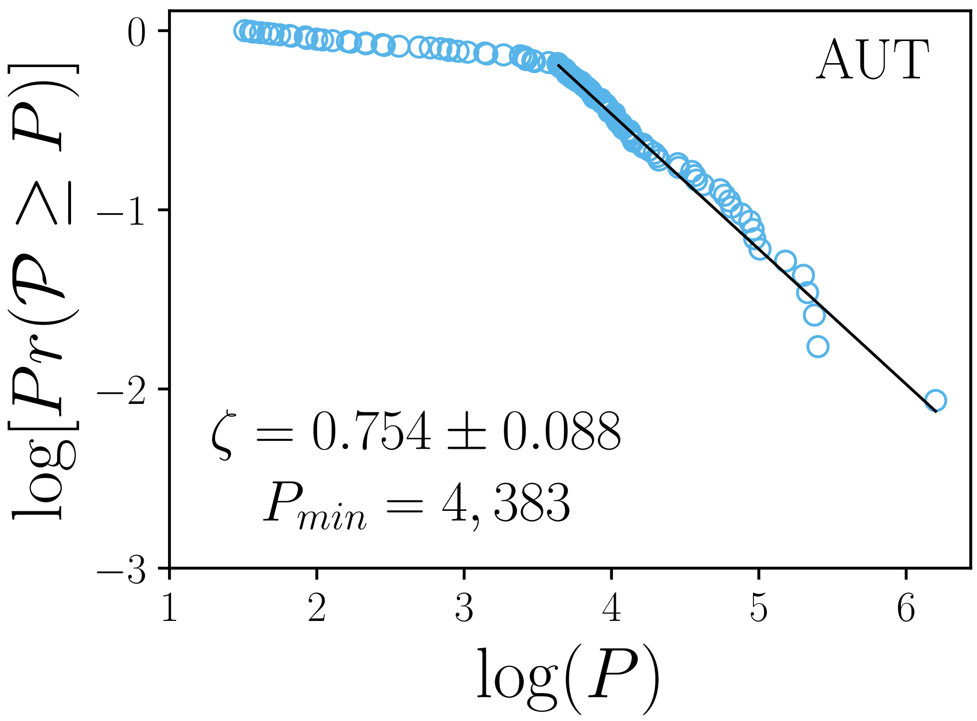

FIG. 97. **(Color online) Cumulative Distribution Function (CDF)** $Pr(\mathcal{P} \geq P)$ **versus population** P **in log-log scale.** Cities proposed by the City Local Clustering Algorithm (CLCA) are represented by light blue circles. The solid black line is the maximum likelihood power-law fit defined by the Maximum Likelihood Estimator (MLE) [50]. The value of the lower bound P_{min} and the exponent ζ are also shown. The CLCA parameters used were $D^{(min)} = 100$ people/km^2, $D^{(max)} = 1000$ people/km^2, $\delta = 10$ people/km^2, $\ell = 3$ km, $A^* = 50$ km^2 and $H^* = 0.50$.

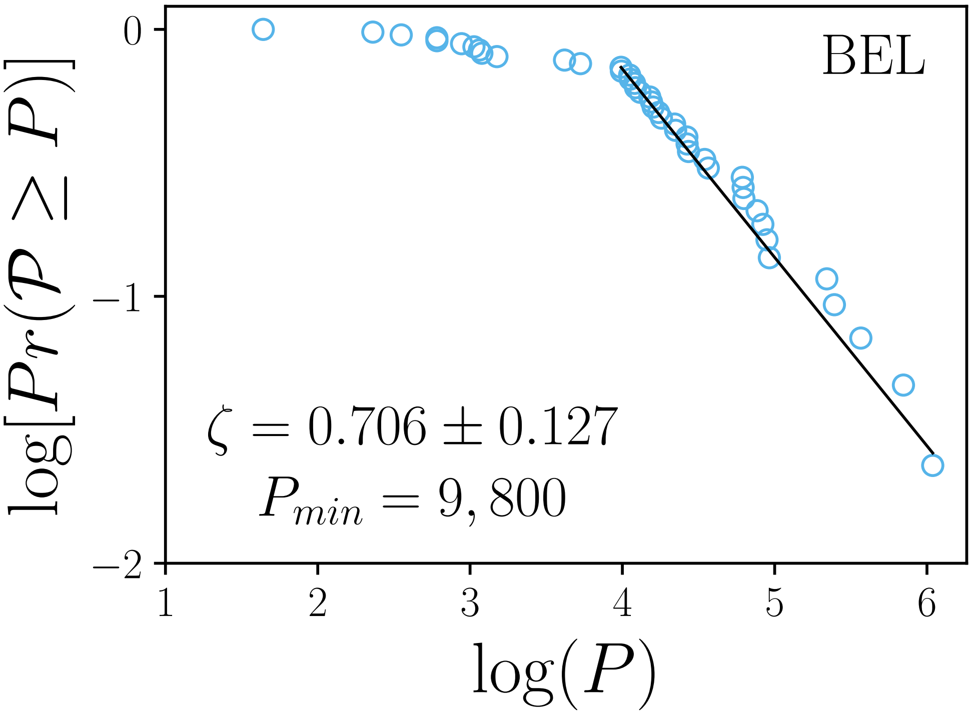

FIG. 98. **(Color online) Cumulative Distribution Function (CDF)** $Pr(\mathcal{P} \geq P)$ **versus population** P **in log-log scale.** Cities proposed by the City Local Clustering Algorithm (CLCA) are represented by light blue circles. The solid black line is the maximum likelihood power-law fit defined by the Maximum Likelihood Estimator (MLE) [50]. The value of the lower bound P_{min} and the exponent ζ are also shown. The CLCA parameters used were $D^{(min)} = 100$ people$/km^2$, $D^{(max)} = 1000$ people$/km^2$, $\delta = 10$ people$/km^2$, $\ell = 3$ km, $A^* = 50$ km^2 and $H^* = 0.50$.

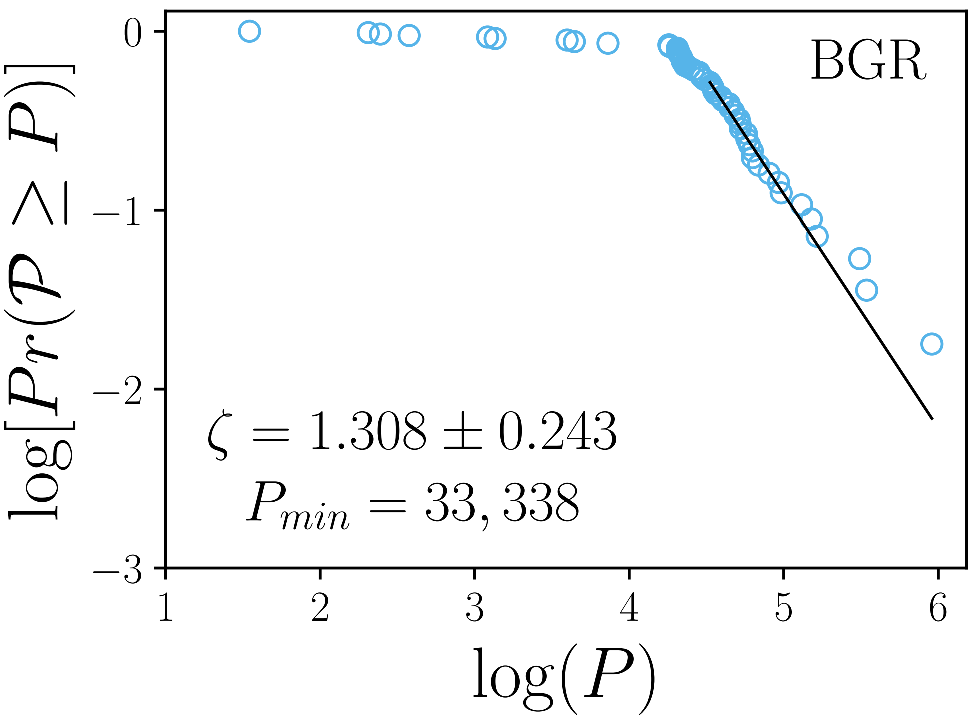

FIG. 99. **(Color online) Cumulative Distribution Function (CDF)** $Pr(\mathcal{P} \geq P)$ **versus population** P **in log-log scale.** Cities proposed by the City Local Clustering Algorithm (CLCA) are represented by light blue circles. The solid black line is the maximum likelihood power-law fit defined by the Maximum Likelihood Estimator (MLE) [50]. The value of the lower bound P_{min} and the exponent ζ are also shown. The CLCA parameters used were $D^{(min)} = 100$ people$/km^2$, $D^{(max)} = 1000$ people$/km^2$, $\delta = 10$ people$/km^2$, $\ell = 3$ km, $A^* = 50$ km^2 and $H^* = 0.50$.

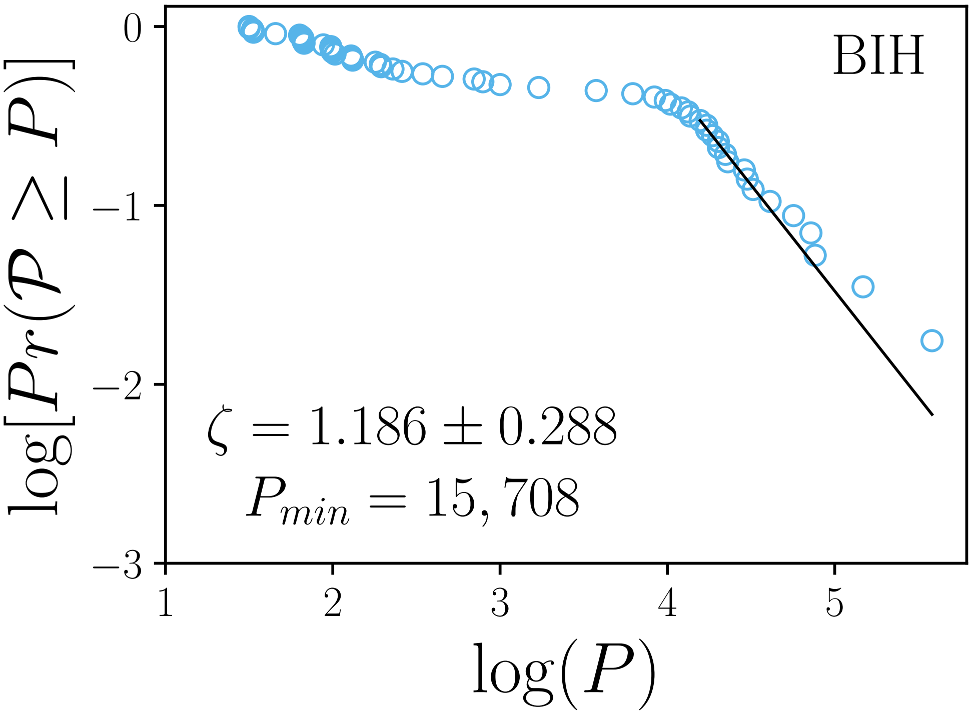

FIG. 100. **(Color online) Cumulative Distribution Function (CDF)** $Pr(\mathcal{P} \geq P)$ **versus population** P **in log-log scale.** Cities proposed by the City Local Clustering Algorithm (CLCA) are represented by light blue circles. The solid black line is the maximum likelihood power-law fit defined by the Maximum Likelihood Estimator (MLE) [50]. The value of the lower bound P_{min} and the exponent ζ are also shown. The CLCA parameters used were $D^{(min)} = 100$ people/km^2, $D^{(max)} = 1000$ people/km^2, $\delta = 10$ people/km^2, $\ell = 3$ km, $A^* = 50$ km^2 and $H^* = 0.50$.

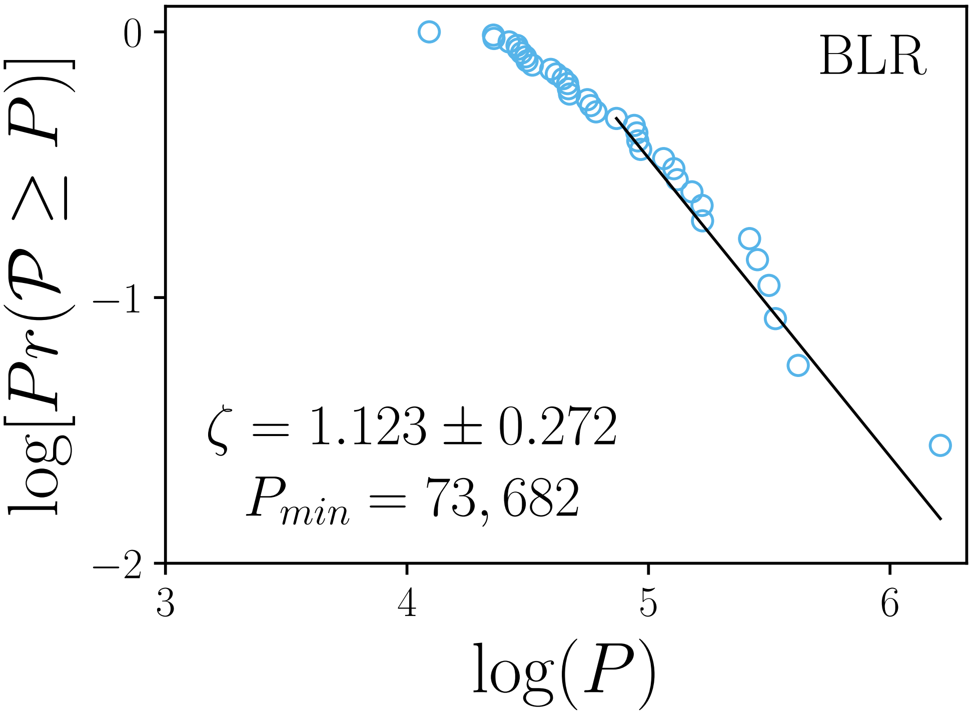

FIG. 101. **(Color online) Cumulative Distribution Function (CDF)** $Pr(\mathcal{P} \geq P)$ **versus population** P **in log-log scale.** Cities proposed by the City Local Clustering Algorithm (CLCA) are represented by light blue circles. The solid black line is the maximum likelihood power-law fit defined by the Maximum Likelihood Estimator (MLE) [50]. The value of the lower bound P_{min} and the exponent ζ are also shown. The CLCA parameters used were $D^{(min)} = 100$ people$/km^2$, $D^{(max)} = 1000$ people$/km^2$, $\delta = 10$ people$/km^2$, $\ell = 3$ km, $A^* = 50$ km^2 and $H^* = 0.50$.

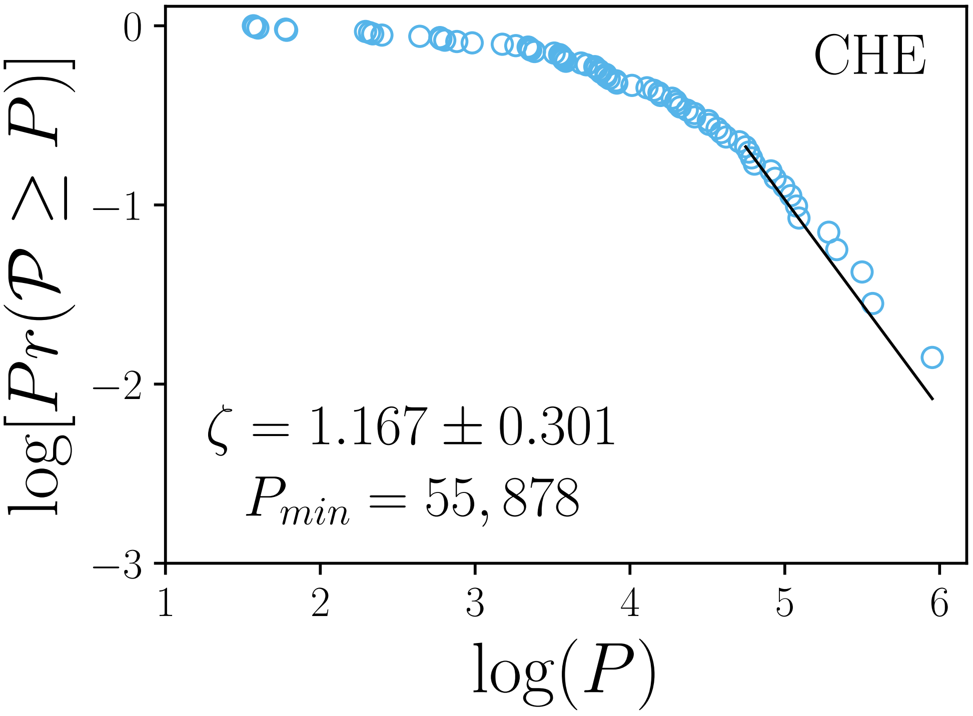

FIG. 102. **(Color online) Cumulative Distribution Function (CDF)** $Pr(\mathcal{P} \geq P)$ **versus population** P **in log-log scale.** Cities proposed by the City Local Clustering Algorithm (CLCA) are represented by light blue circles. The solid black line is the maximum likelihood power-law fit defined by the Maximum Likelihood Estimator (MLE) [50]. The value of the lower bound P_{min} and the exponent ζ are also shown. The CLCA parameters used were $D^{(min)} = 100$ people/km^2, $D^{(max)} = 1000$ people/km^2, $\delta = 10$ people/km^2, $\ell = 3$ km, $A^* = 50$ km^2 and $H^* = 0.50$.

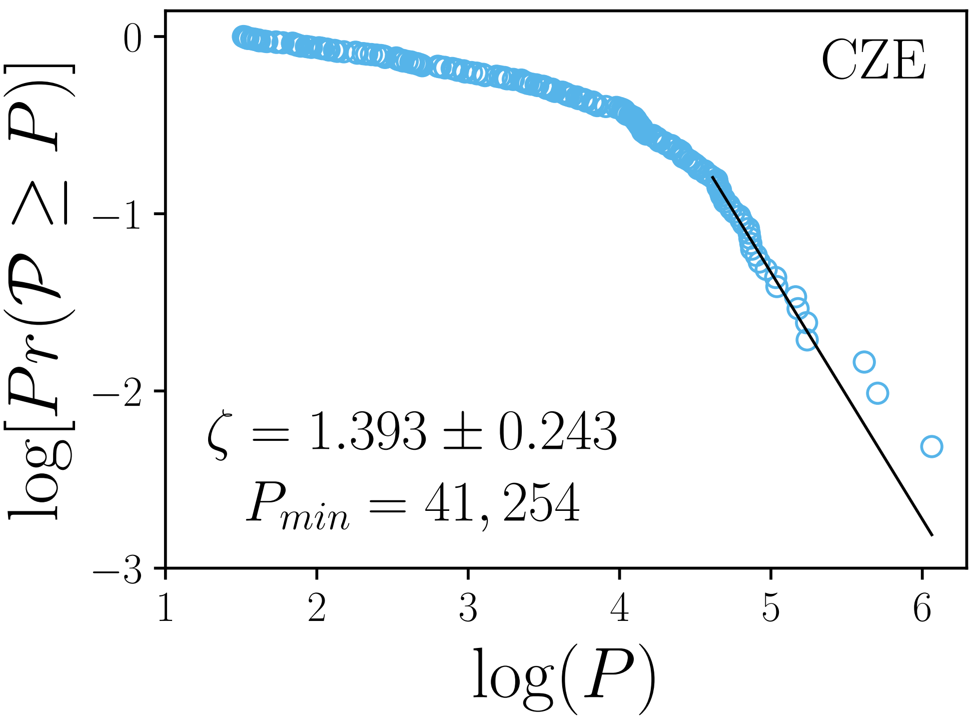

FIG. 103. **(Color online) Cumulative Distribution Function (CDF)** $Pr(\mathcal{P} \geq P)$ **versus population** P **in log-log scale.** Cities proposed by the City Local Clustering Algorithm (CLCA) are represented by light blue circles. The solid black line is the maximum likelihood power-law fit defined by the Maximum Likelihood Estimator (MLE) [50]. The value of the lower bound P_{min} and the exponent ζ are also shown. The CLCA parameters used were $D^{(min)} = 100$ people$/km^2$, $D^{(max)} = 1000$ people$/km^2$, $\delta = 10$ people$/km^2$, $\ell = 3\ km$, $A^* = 50\ km^2$ and $H^* = 0.50$.

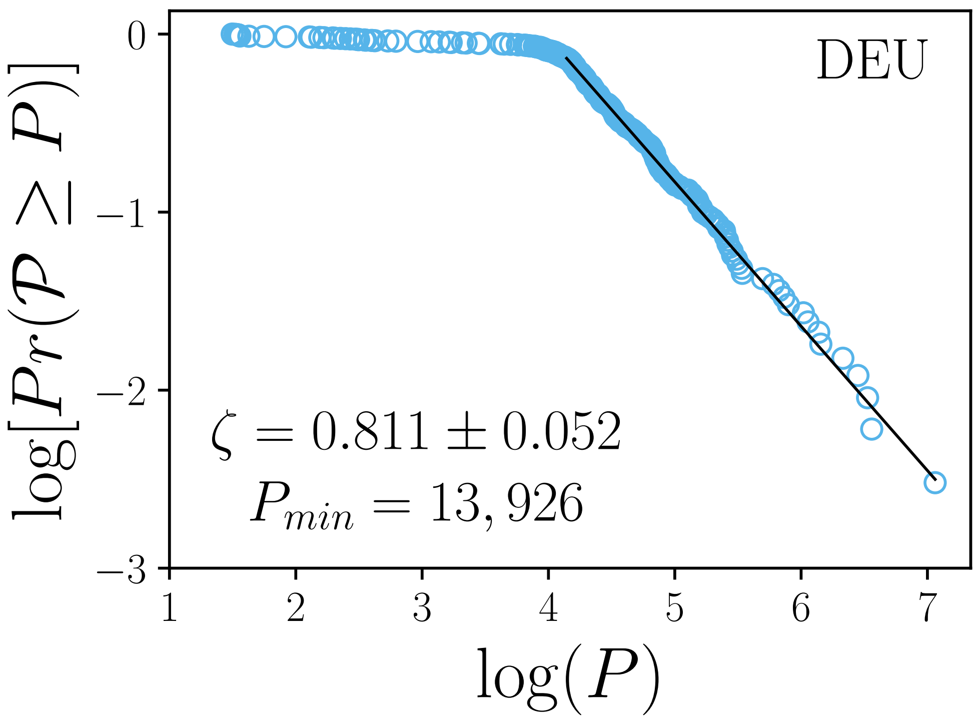

FIG. 104. **(Color online) Cumulative Distribution Function (CDF)** $Pr(\mathcal{P} \geq P)$ **versus population** P **in log-log scale.** Cities proposed by the City Local Clustering Algorithm (CLCA) are represented by light blue circles. The solid black line is the maximum likelihood power-law fit defined by the Maximum Likelihood Estimator (MLE) [50]. The value of the lower bound P_{min} and the exponent ζ are also shown. The CLCA parameters used were $D^{(min)} = 100$ people/km^2, $D^{(max)} = 1000$ people/km^2, $\delta = 10$ people/km^2, $\ell = 3$ km, $A^* = 50$ km^2 and $H^* = 0.50$.

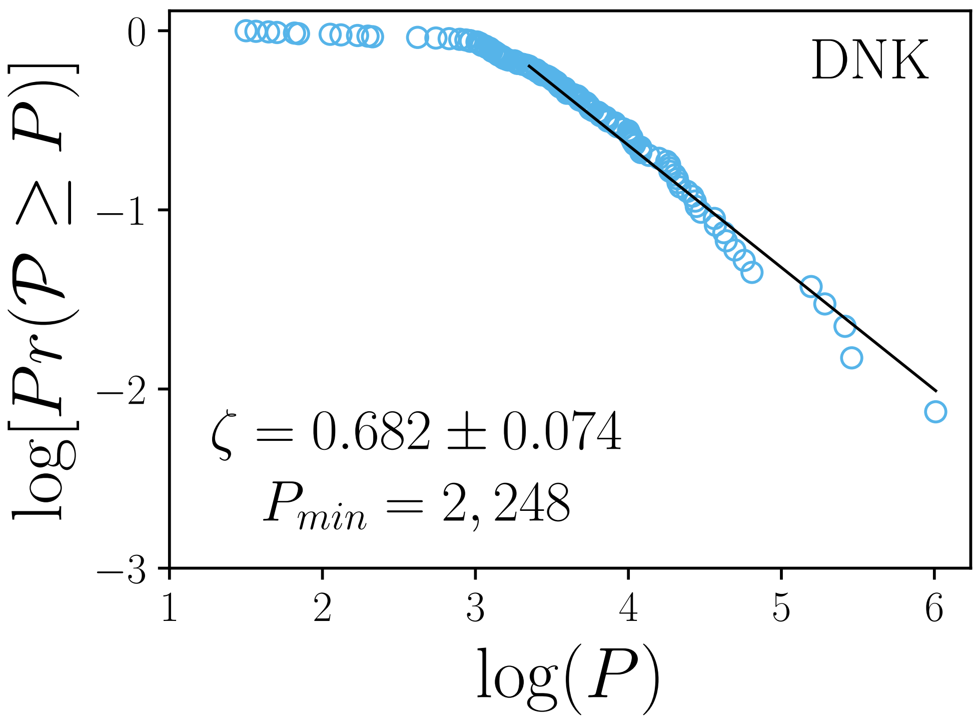

FIG. 105. **(Color online) Cumulative Distribution Function (CDF)** $Pr(\mathcal{P} \geq P)$ **versus population** P **in log-log scale.** Cities proposed by the City Local Clustering Algorithm (CLCA) are represented by light blue circles. The solid black line is the maximum likelihood power-law fit defined by the Maximum Likelihood Estimator (MLE) [50]. The value of the lower bound P_{min} and the exponent ζ are also shown. The CLCA parameters used were $D^{(min)} = 100$ people/km^2, $D^{(max)} = 1000$ people/km^2, $\delta = 10$ people/km^2, $\ell = 3$ km, $A^* = 50$ km^2 and $H^* = 0.50$.

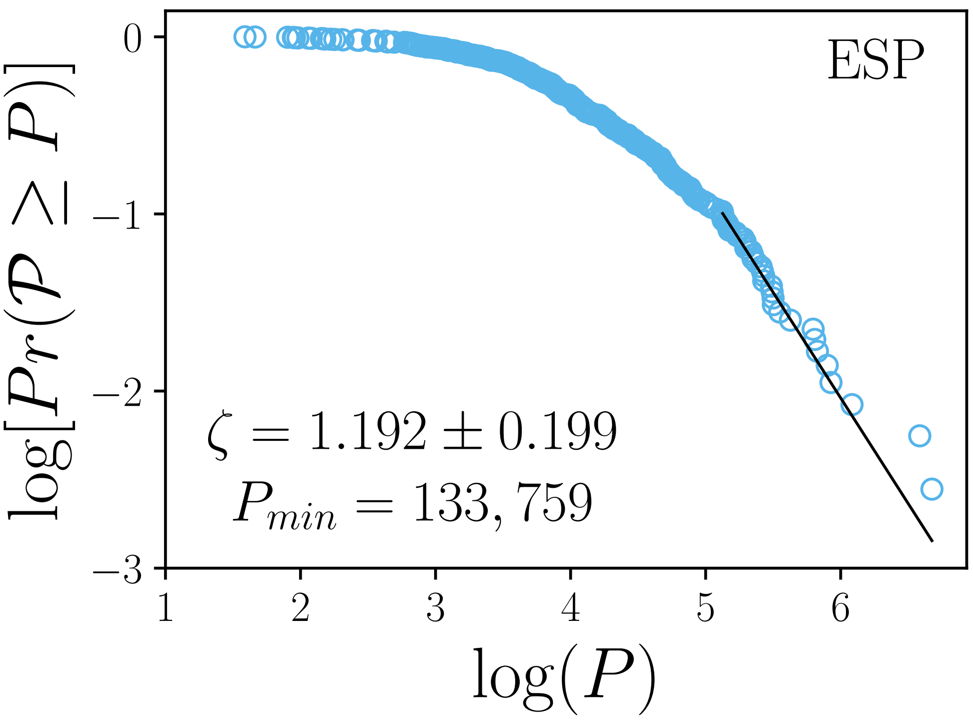

FIG. 106. **(Color online) Cumulative Distribution Function (CDF)** $Pr(\mathcal{P} \geq P)$ **versus population** P **in log-log scale.** Cities proposed by the City Local Clustering Algorithm (CLCA) are represented by light blue circles. The solid black line is the maximum likelihood power-law fit defined by the Maximum Likelihood Estimator (MLE) [50]. The value of the lower bound P_{min} and the exponent ζ are also shown. The CLCA parameters used were $D^{(min)} = 100$ people$/km^2$, $D^{(max)} = 1000$ people$/km^2$, $\delta = 10$ people$/km^2$, $\ell = 3$ km, $A^* = 50$ km^2 and $H^* = 0.50$.

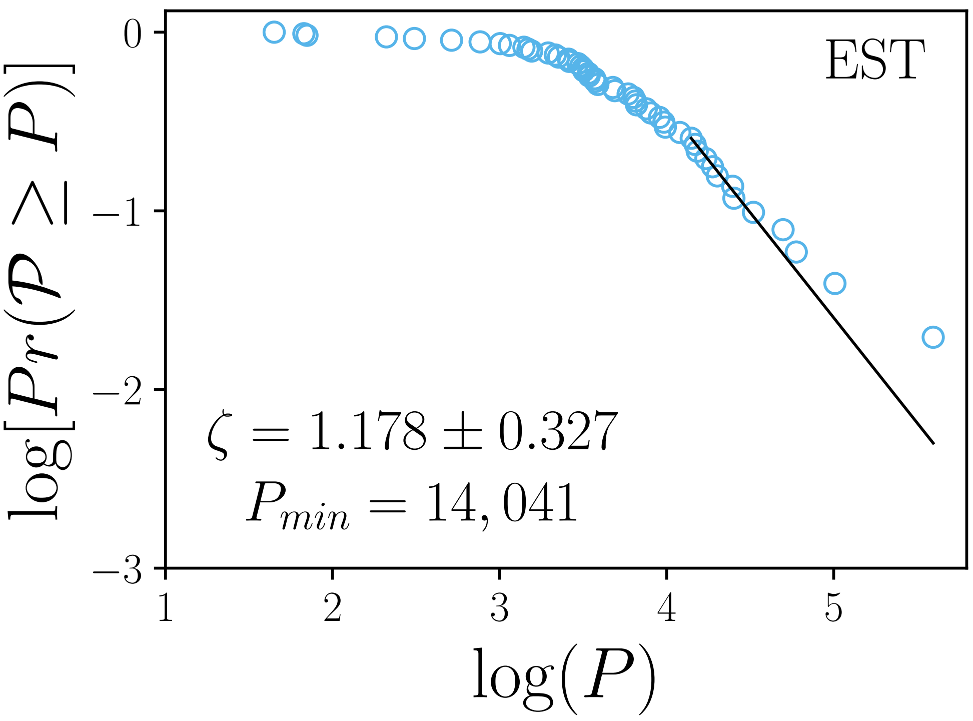

FIG. 107. **(Color online) Cumulative Distribution Function (CDF)** $Pr(\mathcal{P} \geq P)$ **versus population** P **in log-log scale.** Cities proposed by the City Local Clustering Algorithm (CLCA) are represented by light blue circles. The solid black line is the maximum likelihood power-law fit defined by the Maximum Likelihood Estimator (MLE) [50]. The value of the lower bound P_{min} and the exponent ζ are also shown. The CLCA parameters used were $D^{(min)} = 100$ people/km^2, $D^{(max)} = 1000$ people/km^2, $\delta = 10$ people/km^2, $\ell = 3$ km, $A^* = 50$ km^2 and $H^* = 0.50$.

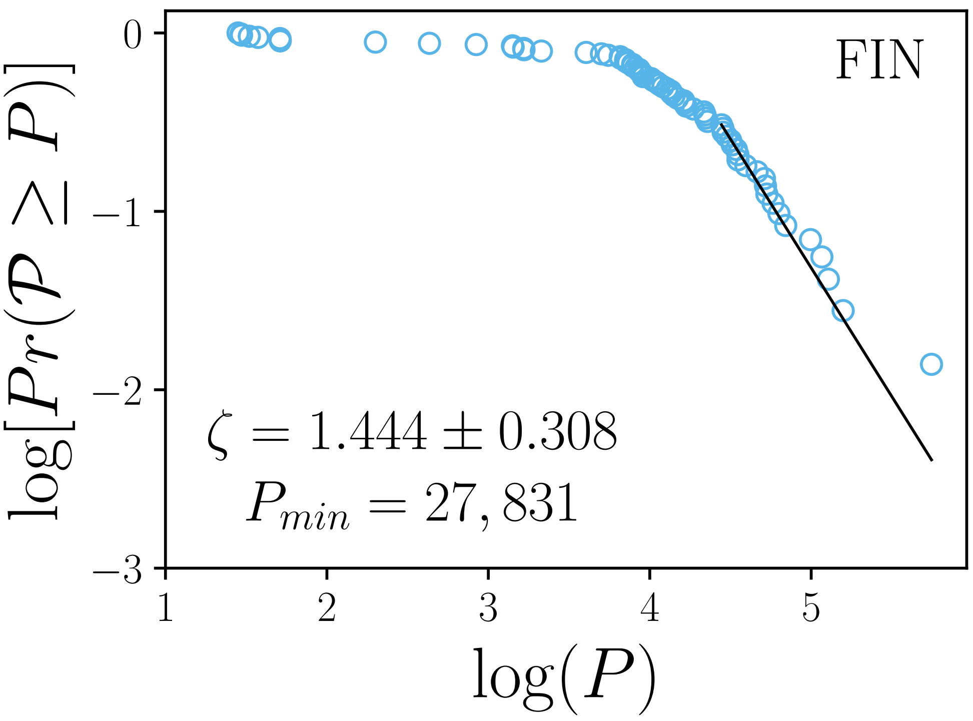

FIG. 108. **(Color online) Cumulative Distribution Function (CDF)** $Pr(\mathcal{P} \geq P)$ **versus population** P **in log-log scale.** Cities proposed by the City Local Clustering Algorithm (CLCA) are represented by light blue circles. The solid black line is the maximum likelihood power-law fit defined by the Maximum Likelihood Estimator (MLE) [50]. The value of the lower bound P_{min} and the exponent ζ are also shown. The CLCA parameters used were $D^{(min)} = 100$ people/km^2, $D^{(max)} = 1000$ people/km^2, $\delta = 10$ people/km^2, $\ell = 3$ km, $A^* = 50$ km^2 and $H^* = 0.50$.

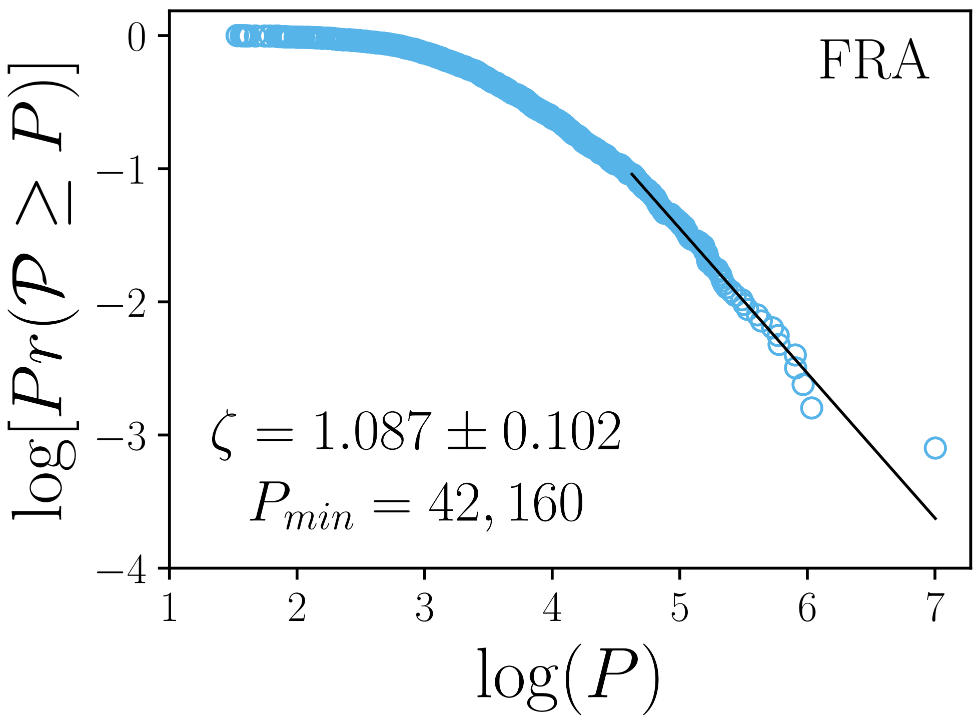

FIG. 109. **(Color online) Cumulative Distribution Function (CDF)** $Pr(\mathcal{P} \geq P)$ **versus population** P **in log-log scale.** Cities proposed by the City Local Clustering Algorithm (CLCA) are represented by light blue circles. The solid black line is the maximum likelihood power-law fit defined by the Maximum Likelihood Estimator (MLE) [50]. The value of the lower bound P_{min} and the exponent ζ are also shown. The CLCA parameters used were $D^{(min)} = 100$ people/km^2, $D^{(max)} = 1000$ people/km^2, $\delta = 10$ people/km^2, $\ell = 3$ km, $A^* = 50$ km^2 and $H^* = 0.50$.

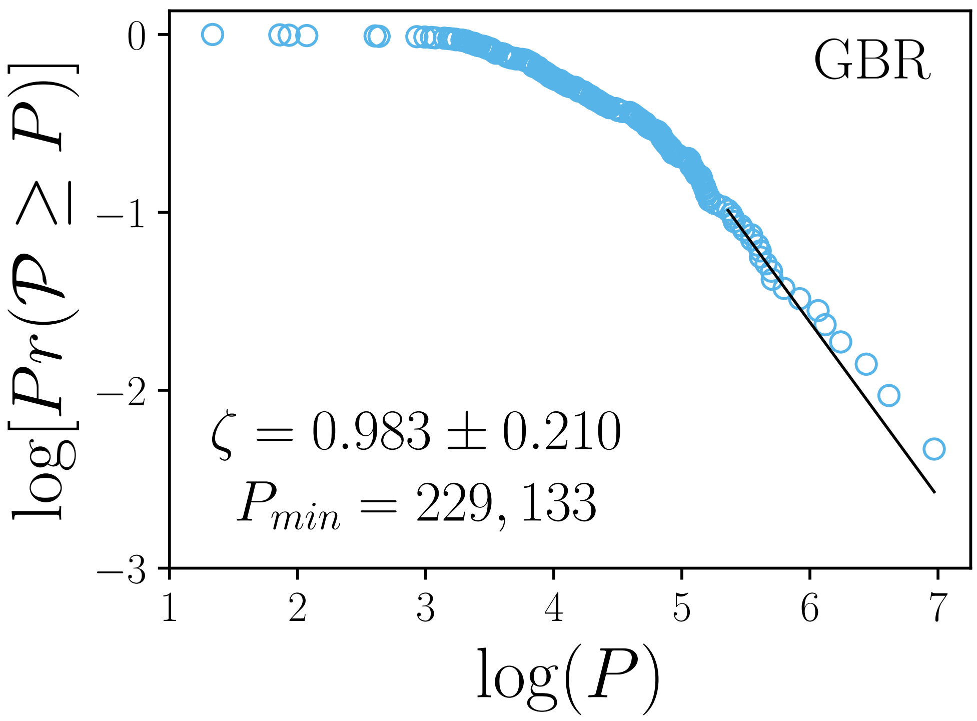

FIG. 110. **(Color online) Cumulative Distribution Function (CDF)** $Pr(\mathcal{P} \geq P)$ **versus population** P **in log-log scale.** Cities proposed by the City Local Clustering Algorithm (CLCA) are represented by light blue circles. The solid black line is the maximum likelihood power-law fit defined by the Maximum Likelihood Estimator (MLE) [50]. The value of the lower bound P_{min} and the exponent ζ are also shown. The CLCA parameters used were $D^{(min)} = 100$ people/km^2, $D^{(max)} = 1000$ people/km^2, $\delta = 10$ people/km^2, $\ell = 3$ km, $A^* = 50$ km^2 and $H^* = 0.50$.

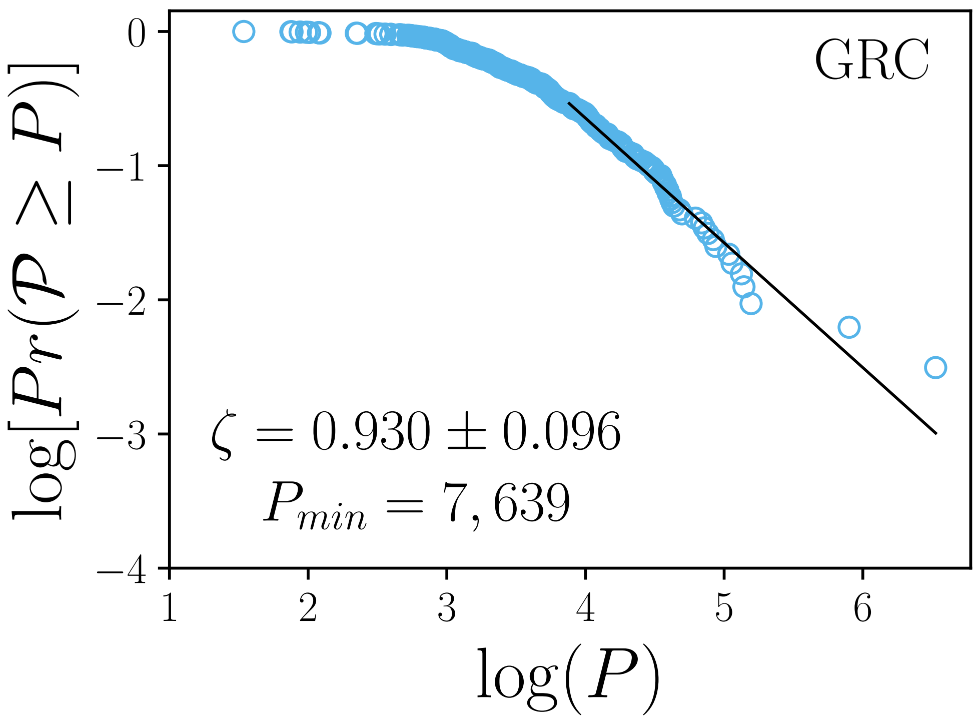

FIG. 111. **(Color online) Cumulative Distribution Function (CDF)** $Pr(\mathcal{P} \geq P)$ **versus population** P **in log-log scale.** Cities proposed by the City Local Clustering Algorithm (CLCA) are represented by light blue circles. The solid black line is the maximum likelihood power-law fit defined by the Maximum Likelihood Estimator (MLE) [50]. The value of the lower bound P_{min} and the exponent ζ are also shown. The CLCA parameters used were $D^{(min)} = 100$ people/km^2, $D^{(max)} = 1000$ people/km^2, $\delta = 10$ people/km^2, $\ell = 3$ km, $A^* = 50$ km^2 and $H^* = 0.50$.

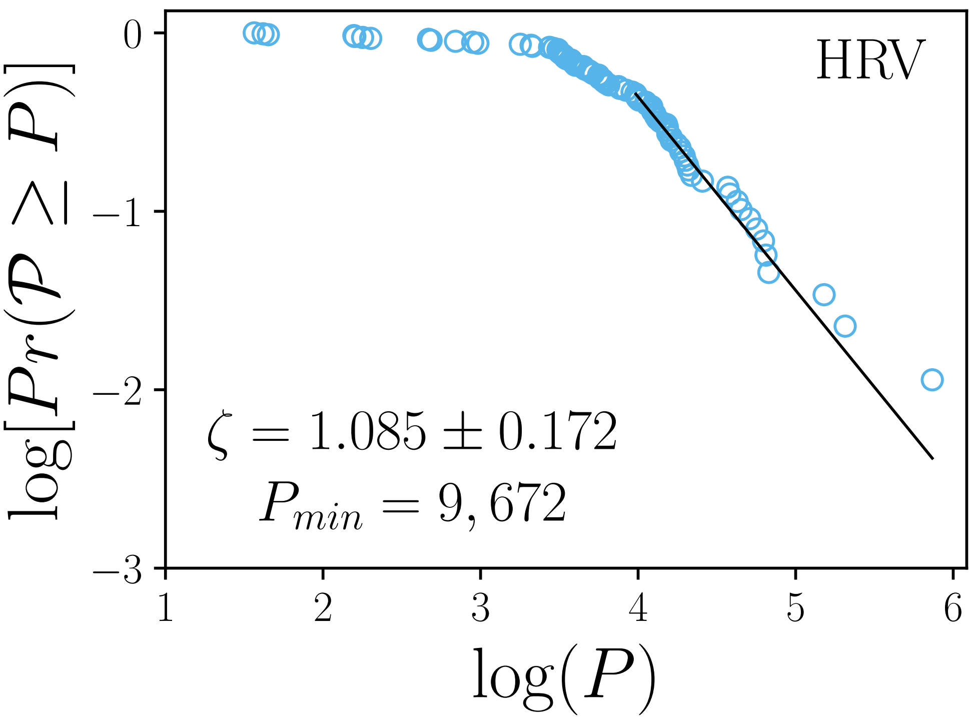

FIG. 112. **(Color online) Cumulative Distribution Function (CDF)** $Pr(\mathcal{P} \geq P)$ **versus population** P **in log-log scale.** Cities proposed by the City Local Clustering Algorithm (CLCA) are represented by light blue circles. The solid black line is the maximum likelihood power-law fit defined by the Maximum Likelihood Estimator (MLE) [50]. The value of the lower bound P_{min} and the exponent ζ are also shown. The CLCA parameters used were $D^{(min)} = 100$ people/km^2, $D^{(max)} = 1000$ people/km^2, $\delta = 10$ people/km^2, $\ell = 3$ km, $A^* = 50$ km^2 and $H^* = 0.50$.

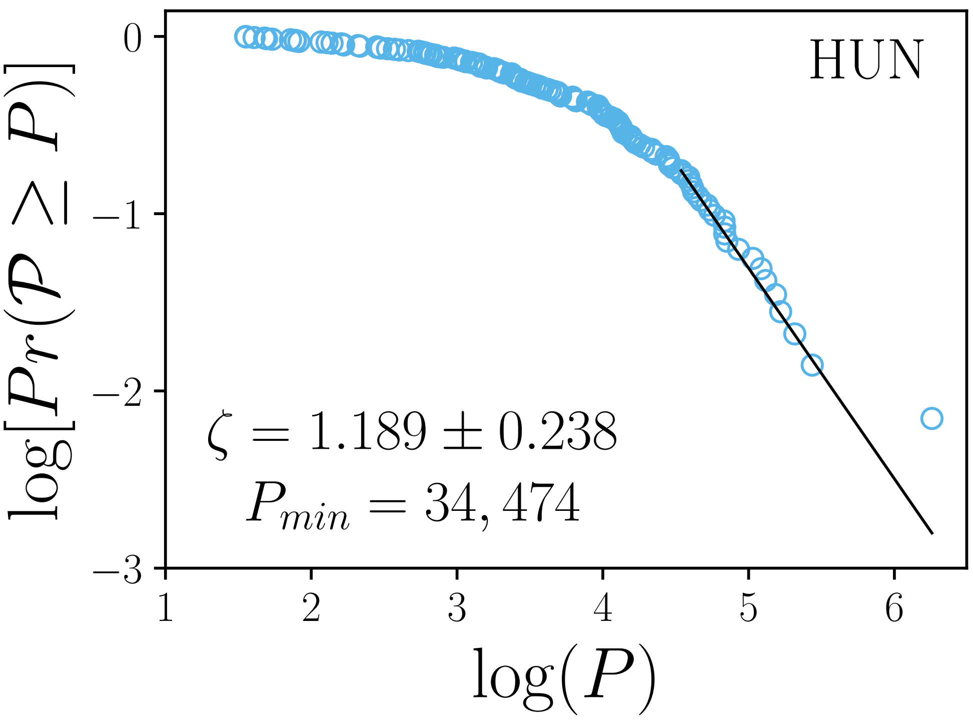

FIG. 113. **(Color online) Cumulative Distribution Function (CDF)** $Pr(\mathcal{P} \geq P)$ **versus population** P **in log-log scale.** Cities proposed by the City Local Clustering Algorithm (CLCA) are represented by light blue circles. The solid black line is the maximum likelihood power-law fit defined by the Maximum Likelihood Estimator (MLE) [50]. The value of the lower bound P_{min} and the exponent ζ are also shown. The CLCA parameters used were $D^{(min)} = 100$ people$/km^2$, $D^{(max)} = 1000$ people$/km^2$, $\delta = 10$ people$/km^2$, $\ell = 3\ km$, $A^* = 50\ km^2$ and $H^* = 0.50$.

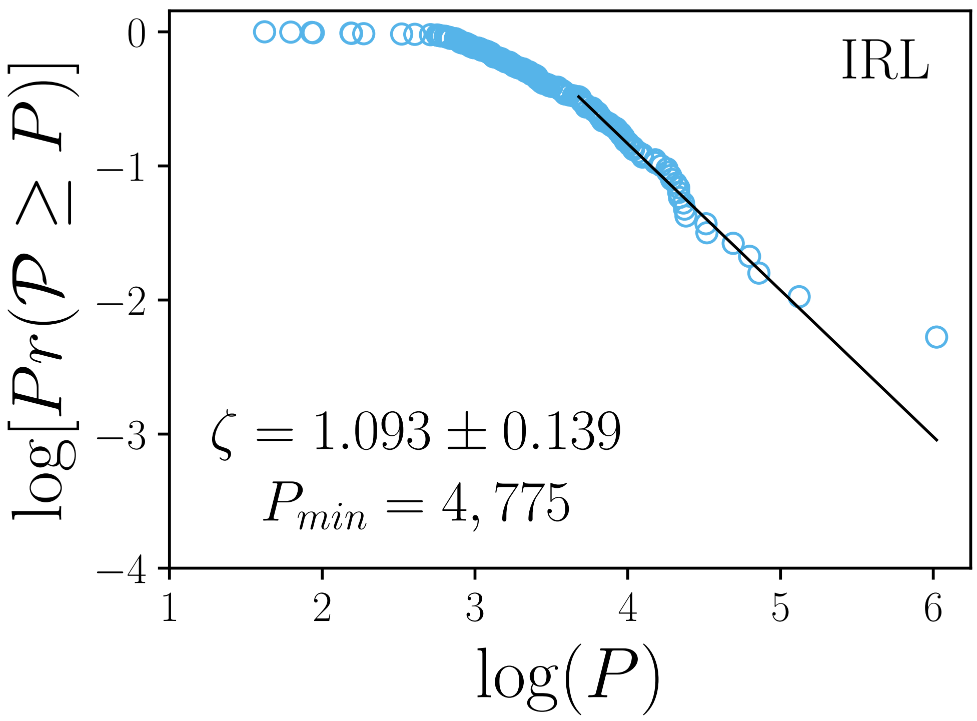

FIG. 114. **(Color online) Cumulative Distribution Function (CDF)** $Pr(\mathcal{P} \geq P)$ **versus population** P **in log-log scale.** Cities proposed by the City Local Clustering Algorithm (CLCA) are represented by light blue circles. The solid black line is the maximum likelihood power-law fit defined by the Maximum Likelihood Estimator (MLE) [50]. The value of the lower bound P_{min} and the exponent ζ are also shown. The CLCA parameters used were $D^{(min)} = 100$ people/km^2, $D^{(max)} = 1000$ people/km^2, $\delta = 10$ people/km^2, $\ell = 3$ km, $A^* = 50$ km^2 and $H^* = 0.50$.

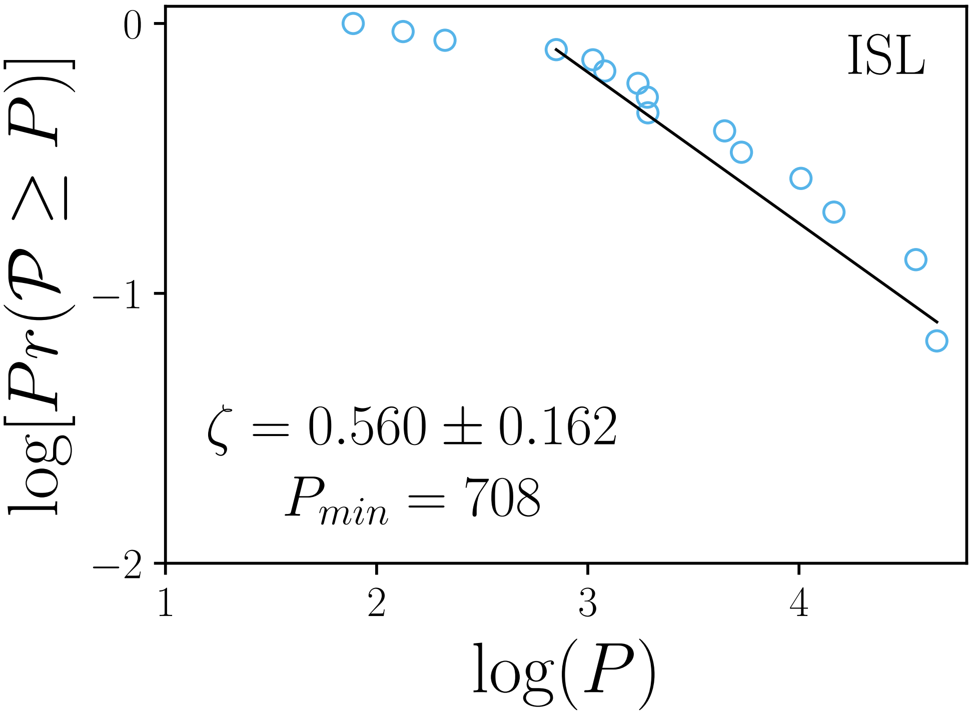

FIG. 115. **(Color online) Cumulative Distribution Function (CDF)** $Pr(\mathcal{P} \geq P)$ **versus population** P **in log-log scale.** Cities proposed by the City Local Clustering Algorithm (CLCA) are represented by light blue circles. The solid black line is the maximum likelihood power-law fit defined by the Maximum Likelihood Estimator (MLE) [50]. The value of the lower bound P_{min} and the exponent ζ are also shown. The CLCA parameters used were $D^{(min)} = 100$ people$/km^2$, $D^{(max)} = 1000$ people$/km^2$, $\delta = 10$ people$/km^2$, $\ell = 3$ km, $A^* = 50$ km^2 and $H^* = 0.50$.

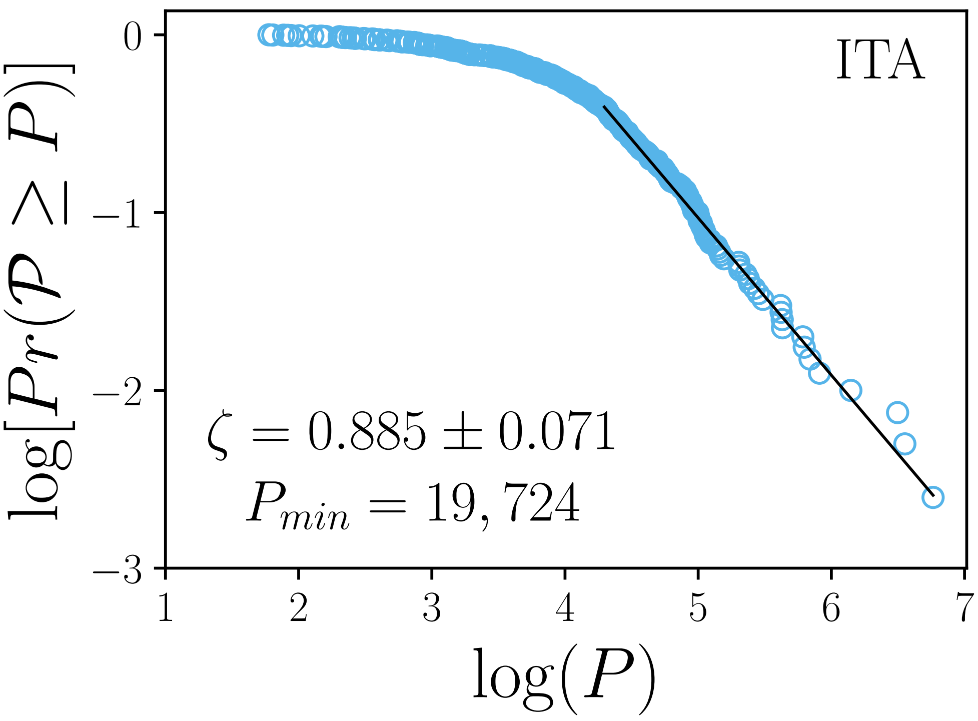

FIG. 116. **(Color online) Cumulative Distribution Function (CDF)** $Pr(\mathcal{P} \geq P)$ **versus population** P **in log-log scale.** Cities proposed by the City Local Clustering Algorithm (CLCA) are represented by light blue circles. The solid black line is the maximum likelihood power-law fit defined by the Maximum Likelihood Estimator (MLE) [50]. The value of the lower bound P_{min} and the exponent ζ are also shown. The CLCA parameters used were $D^{(min)} = 100$ people$/km^2$, $D^{(max)} = 1000$ people$/km^2$, $\delta = 10$ people$/km^2$, $\ell = 3\ km$, $A^* = 50\ km^2$ and $H^* = 0.50$.

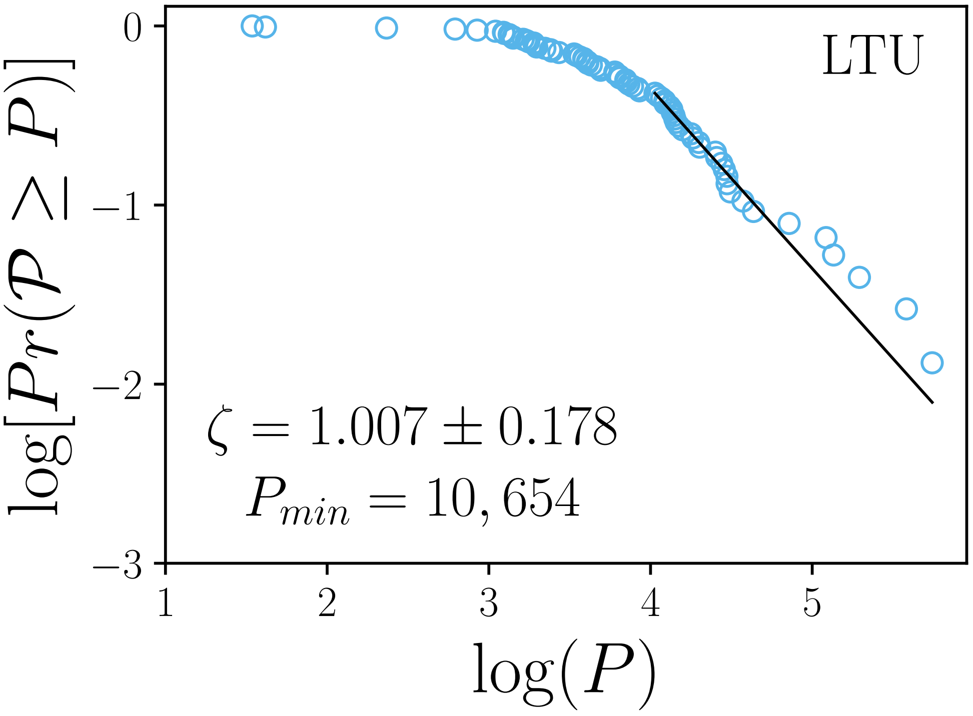

FIG. 117. **(Color online) Cumulative Distribution Function (CDF)** $Pr(\mathcal{P} \geq P)$ **versus population** P **in log-log scale.** Cities proposed by the City Local Clustering Algorithm (CLCA) are represented by light blue circles. The solid black line is the maximum likelihood power-law fit defined by the Maximum Likelihood Estimator (MLE) [50]. The value of the lower bound P_{min} and the exponent ζ are also shown. The CLCA parameters used were $D^{(min)} = 100$ people$/km^2$, $D^{(max)} = 1000$ people$/km^2$, $\delta = 10$ people$/km^2$, $\ell = 3$ km, $A^* = 50$ km^2 and $H^* = 0.50$.

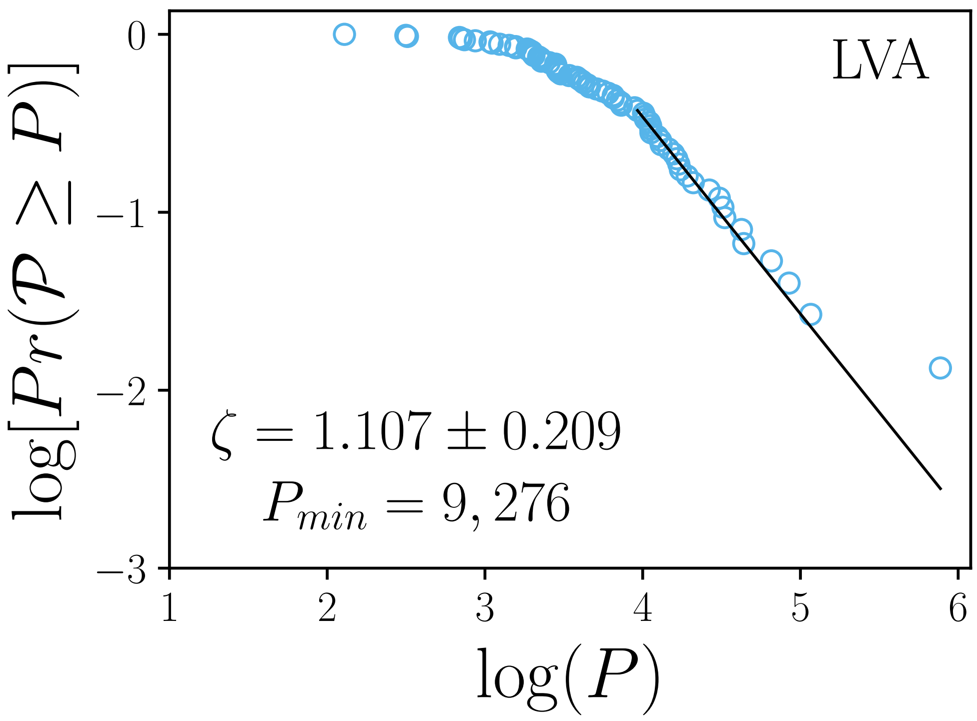

FIG. 118. **(Color online) Cumulative Distribution Function (CDF)** $Pr(\mathcal{P} \geq P)$ **versus population** P **in log-log scale.** Cities proposed by the City Local Clustering Algorithm (CLCA) are represented by light blue circles. The solid black line is the maximum likelihood power-law fit defined by the Maximum Likelihood Estimator (MLE) [50]. The value of the lower bound P_{min} and the exponent ζ are also shown. The CLCA parameters used were $D^{(min)} = 100$ people/km^2, $D^{(max)} = 1000$ people/km^2, $\delta = 10$ people/km^2, $\ell = 3$ km, $A^* = 50$ km^2 and $H^* = 0.50$.

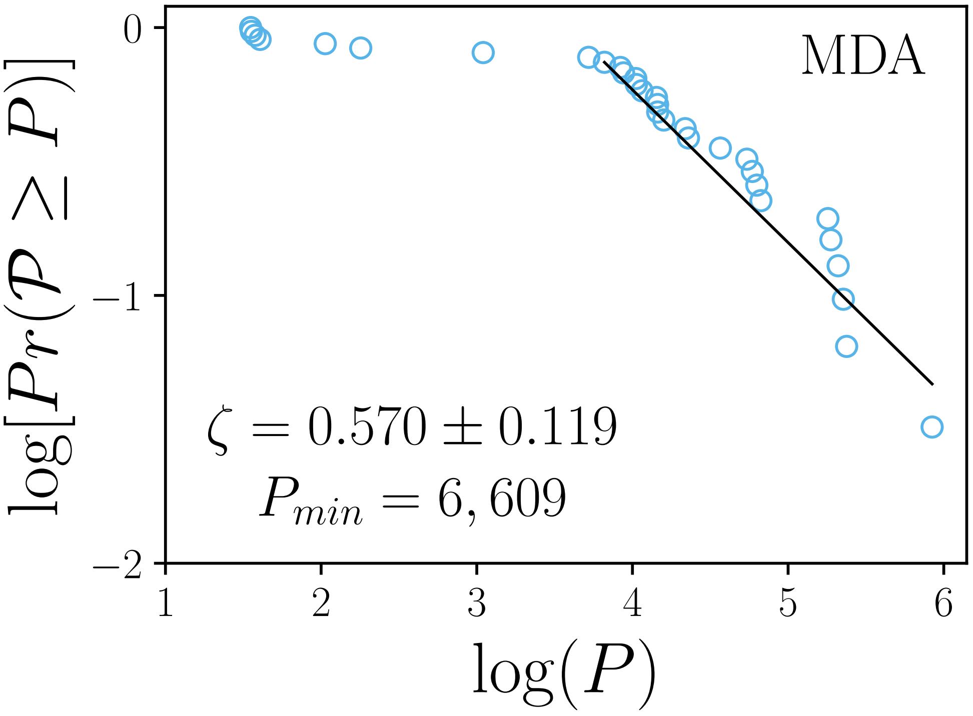

FIG. 119. **(Color online) Cumulative Distribution Function (CDF)** $Pr(\mathcal{P} \geq P)$ **versus population** P **in log-log scale.** Cities proposed by the City Local Clustering Algorithm (CLCA) are represented by light blue circles. The solid black line is the maximum likelihood power-law fit defined by the Maximum Likelihood Estimator (MLE) [50]. The value of the lower bound P_{min} and the exponent ζ are also shown. The CLCA parameters used were $D^{(min)} = 100$ people$/km^2$, $D^{(max)} = 1000$ people$/km^2$, $\delta = 10$ people$/km^2$, $\ell = 3\ km$, $A^* = 50\ km^2$ and $H^* = 0.50$.

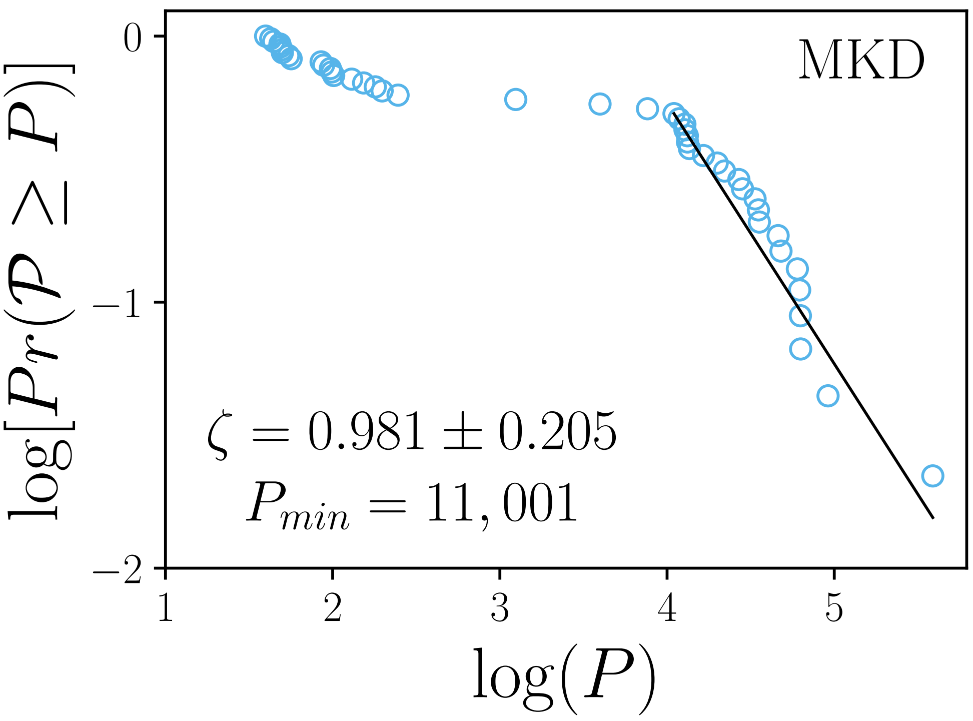

FIG. 120. **(Color online) Cumulative Distribution Function (CDF)** $Pr(\mathcal{P} \geq P)$ **versus population** P **in log-log scale.** Cities proposed by the City Local Clustering Algorithm (CLCA) are represented by light blue circles. The solid black line is the maximum likelihood power-law fit defined by the Maximum Likelihood Estimator (MLE) [50]. The value of the lower bound P_{min} and the exponent ζ are also shown. The CLCA parameters used were $D^{(min)} = 100$ people/km^2, $D^{(max)} = 1000$ people/km^2, $\delta = 10$ people/km^2, $\ell = 3$ km, $A^* = 50$ km^2 and $H^* = 0.50$.

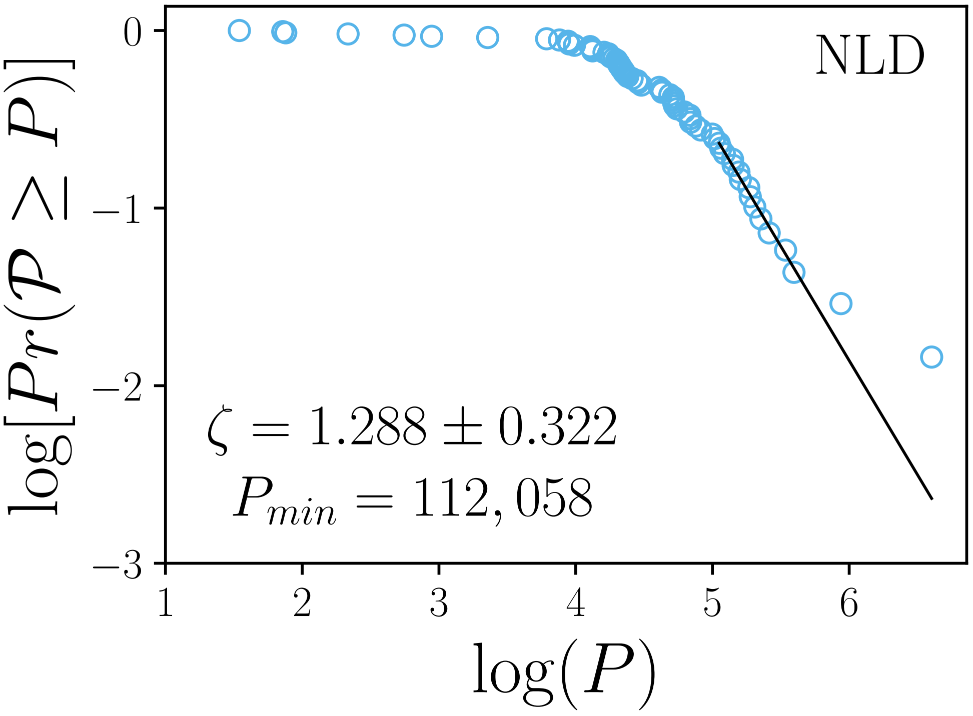

FIG. 121. **(Color online) Cumulative Distribution Function (CDF)** $Pr(\mathcal{P} \geq P)$ **versus population** P **in log-log scale.** Cities proposed by the City Local Clustering Algorithm (CLCA) are represented by light blue circles. The solid black line is the maximum likelihood power-law fit defined by the Maximum Likelihood Estimator (MLE) [50]. The value of the lower bound P_{min} and the exponent ζ are also shown. The CLCA parameters used were $D^{(min)} = 100$ people/km^2, $D^{(max)} = 1000$ people/km^2, $\delta = 10$ people/km^2, $\ell = 3$ km, $A^* = 50$ km^2 and $H^* = 0.50$.

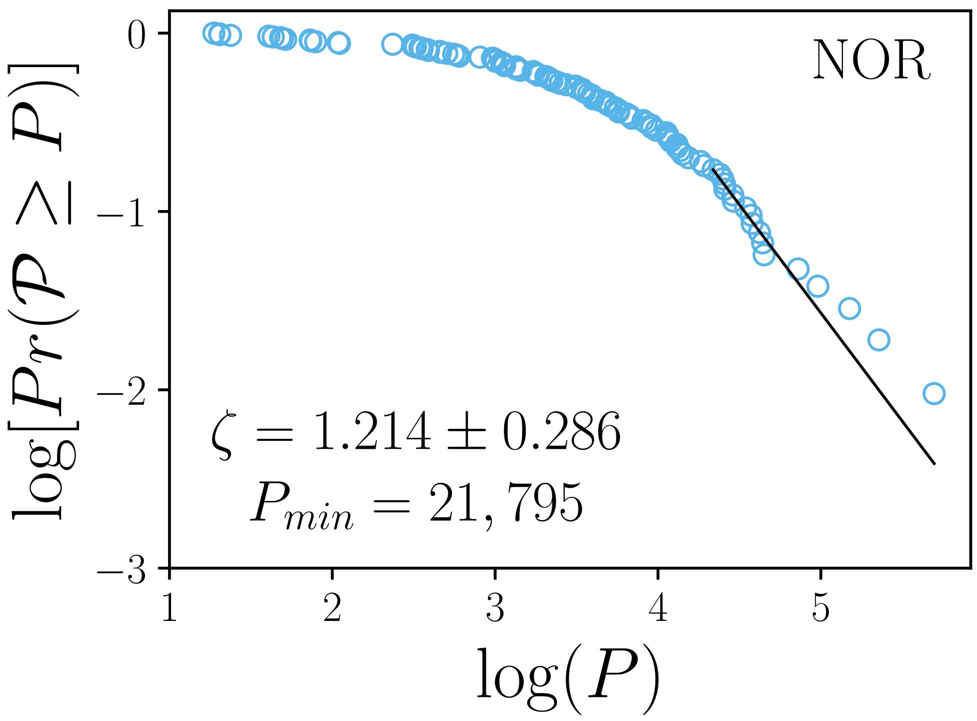

FIG. 122. **(Color online) Cumulative Distribution Function (CDF)** $Pr(\mathcal{P} \geq P)$ **versus population** P **in log-log scale.** Cities proposed by the City Local Clustering Algorithm (CLCA) are represented by light blue circles. The solid black line is the maximum likelihood power-law fit defined by the Maximum Likelihood Estimator (MLE) [50]. The value of the lower bound P_{min} and the exponent ζ are also shown. The CLCA parameters used were $D^{(min)} = 100$ people/km^2, $D^{(max)} = 1000$ people/km^2, $\delta = 10$ people/km^2, $\ell = 3$ km, $A^* = 50$ km^2 and $H^* = 0.50$.

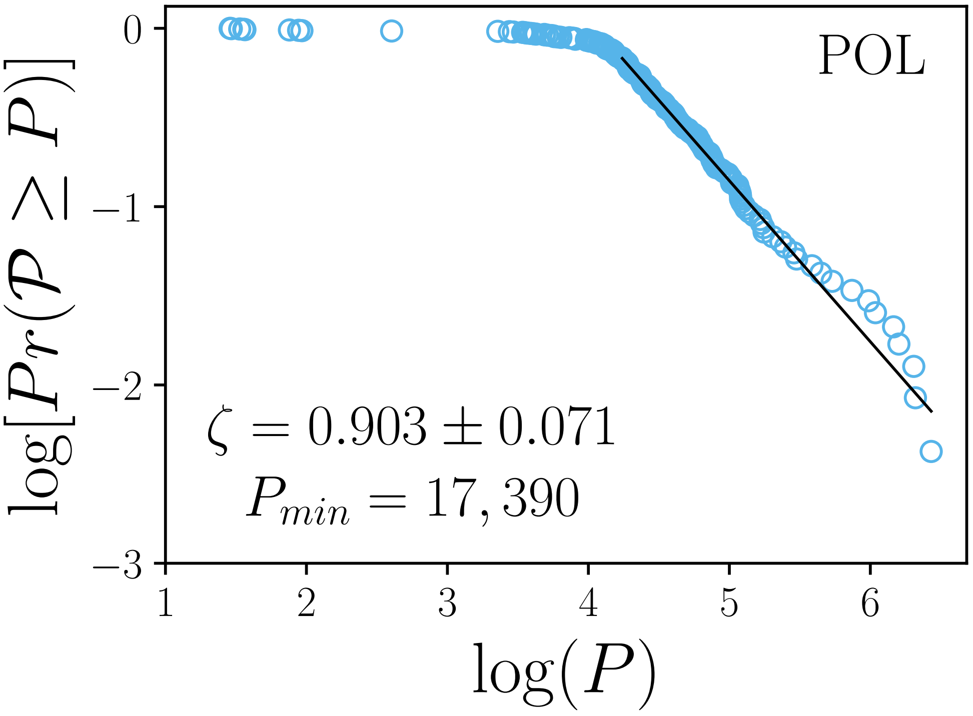

FIG. 123. **(Color online) Cumulative Distribution Function (CDF)** $Pr(\mathcal{P} \geq P)$ **versus population** P **in log-log scale.** Cities proposed by the City Local Clustering Algorithm (CLCA) are represented by light blue circles. The solid black line is the maximum likelihood power-law fit defined by the Maximum Likelihood Estimator (MLE) [50]. The value of the lower bound P_{min} and the exponent ζ are also shown. The CLCA parameters used were $D^{(min)} = 100$ people$/km^2$, $D^{(max)} = 1000$ people$/km^2$, $\delta = 10$ people$/km^2$, $\ell = 3$ km, $A^* = 50$ km^2 and $H^* = 0.50$.

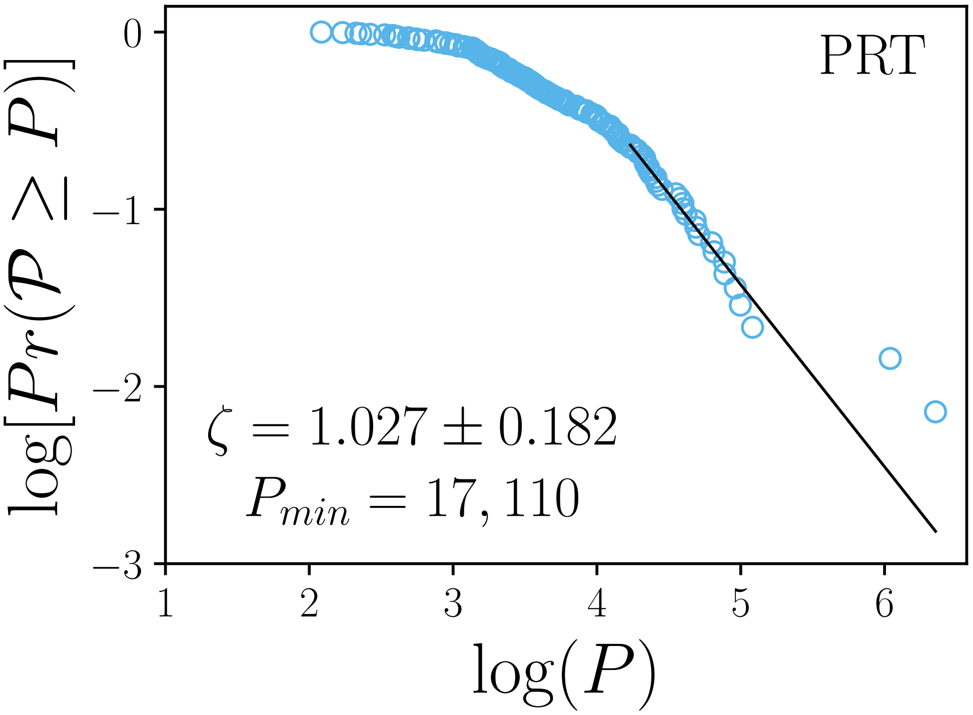

FIG. 124. **(Color online) Cumulative Distribution Function (CDF)** $Pr(\mathcal{P} \geq P)$ **versus population** P **in log-log scale.** Cities proposed by the City Local Clustering Algorithm (CLCA) are represented by light blue circles. The solid black line is the maximum likelihood power-law fit defined by the Maximum Likelihood Estimator (MLE) [50]. The value of the lower bound P_{min} and the exponent ζ are also shown. The CLCA parameters used were $D^{(min)} = 100$ people/km^2, $D^{(max)} = 1000$ people/km^2, $\delta = 10$ people/km^2, $\ell = 3$ km, $A^* = 50$ km^2 and $H^* = 0.50$.

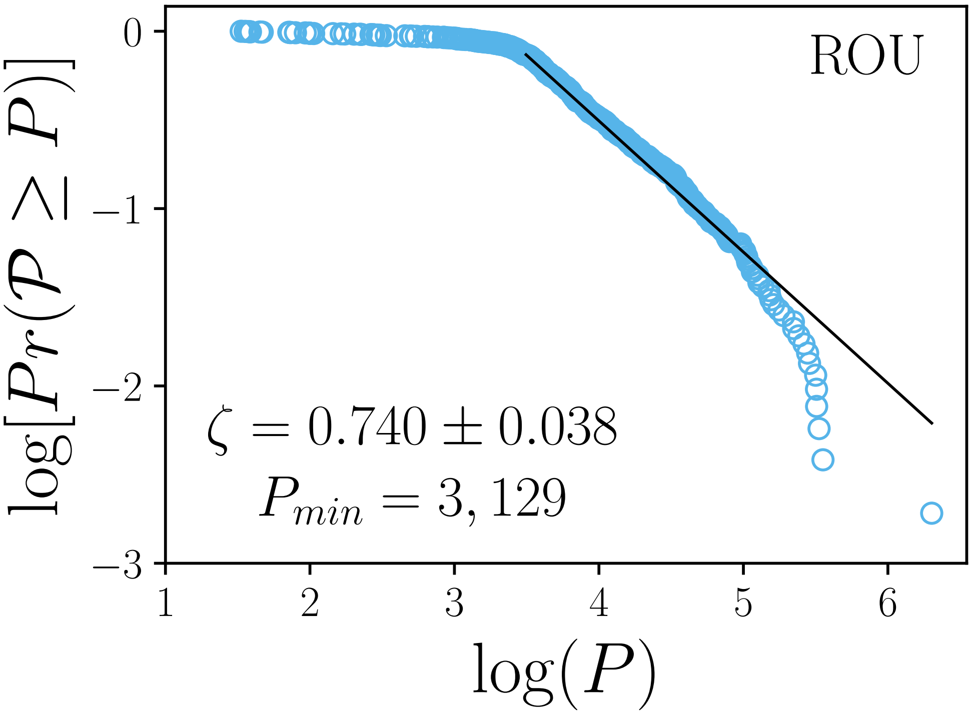

FIG. 125. **(Color online) Cumulative Distribution Function (CDF)** $Pr(\mathcal{P} \geq P)$ **versus population** P **in log-log scale.** Cities proposed by the City Local Clustering Algorithm (CLCA) are represented by light blue circles. The solid black line is the maximum likelihood power-law fit defined by the Maximum Likelihood Estimator (MLE) [50]. The value of the lower bound P_{min} and the exponent ζ are also shown. The CLCA parameters used were $D^{(min)} = 100$ people/km^2, $D^{(max)} = 1000$ people/km^2, $\delta = 10$ people/km^2, $\ell = 3$ km, $A^* = 50$ km^2 and $H^* = 0.50$.

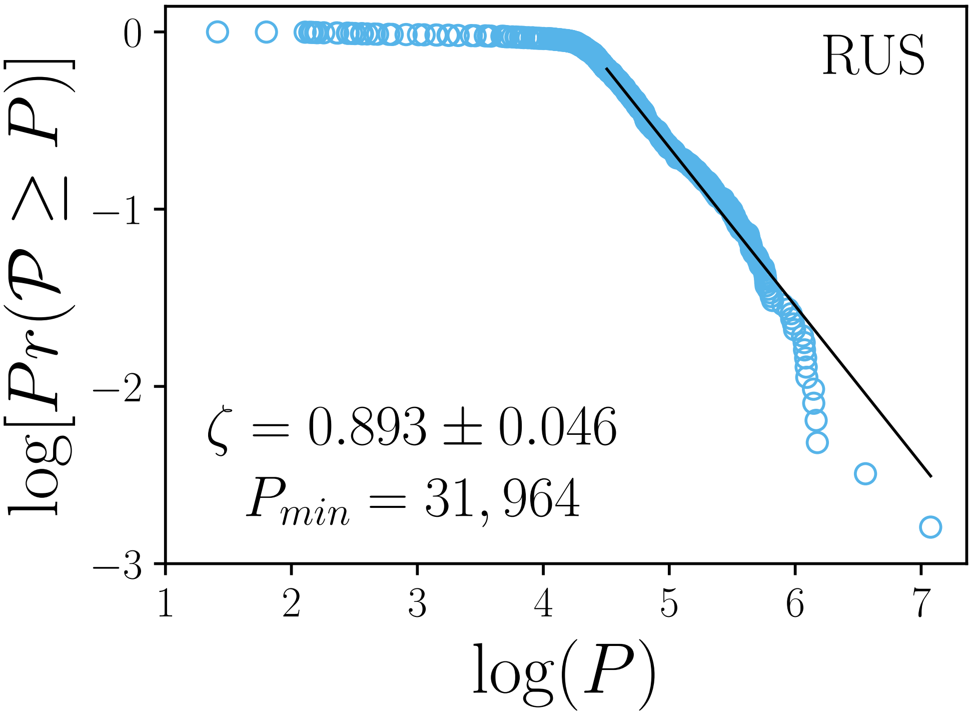

FIG. 126. **(Color online) Cumulative Distribution Function (CDF)** $Pr(\mathcal{P} \geq P)$ **versus population** P **in log-log scale.** Cities proposed by the City Local Clustering Algorithm (CLCA) are represented by light blue circles. The solid black line is the maximum likelihood power-law fit defined by the Maximum Likelihood Estimator (MLE) [50]. The value of the lower bound P_{min} and the exponent ζ are also shown. The CLCA parameters used were $D^{(min)} = 100$ people$/km^2$, $D^{(max)} = 1000$ people$/km^2$, $\delta = 10$ people$/km^2$, $\ell = 3$ km, $A^* = 50$ km^2 and $H^* = 0.50$.

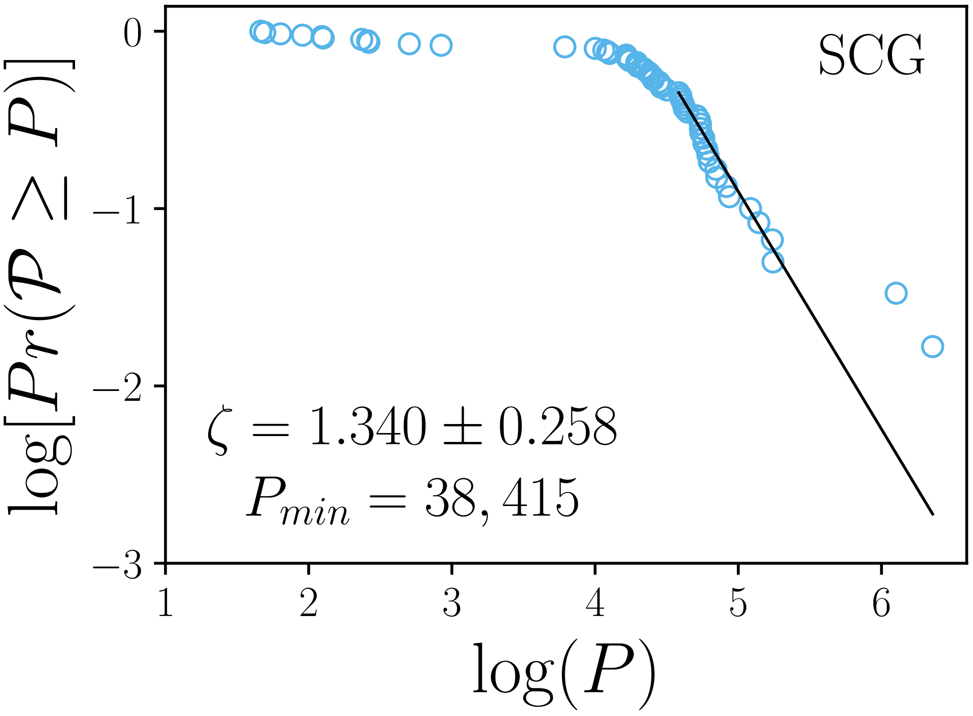

FIG. 127. **(Color online) Cumulative Distribution Function (CDF)** $Pr(\mathcal{P} \geq P)$ **versus population** P **in log-log scale.** Cities proposed by the City Local Clustering Algorithm (CLCA) are represented by light blue circles. The solid black line is the maximum likelihood power-law fit defined by the Maximum Likelihood Estimator (MLE) [50]. The value of the lower bound P_{min} and the exponent ζ are also shown. The CLCA parameters used were $D^{(min)} = 100$ people$/km^2$, $D^{(max)} = 1000$ people$/km^2$, $\delta = 10$ people$/km^2$, $\ell = 3$ km, $A^* = 50$ km^2 and $H^* = 0.50$.

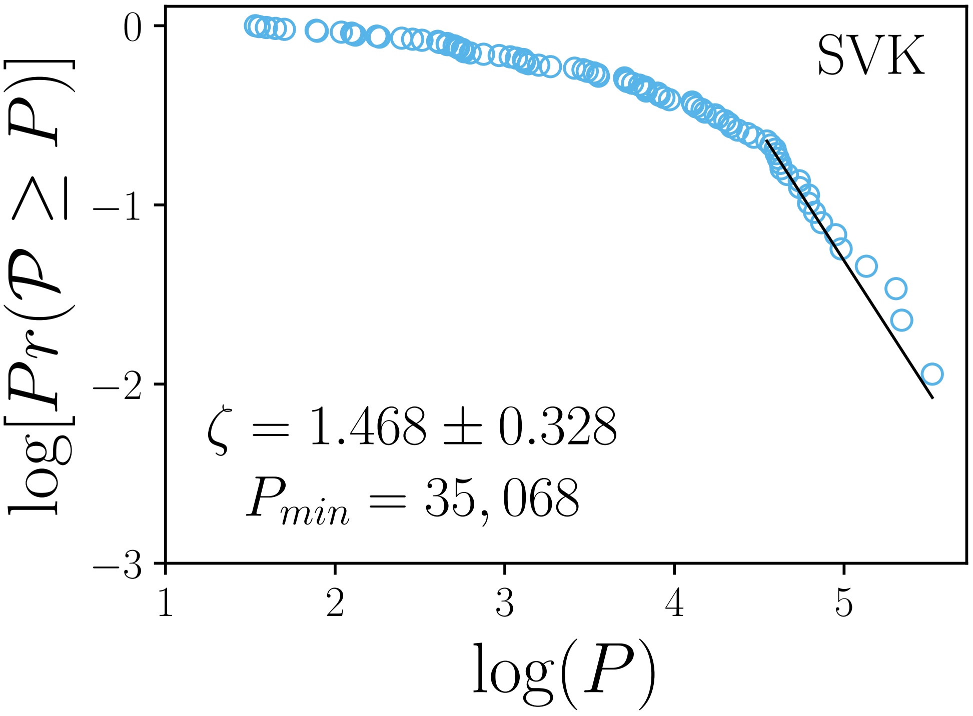

FIG. 128. **(Color online) Cumulative Distribution Function (CDF)** $Pr(\mathcal{P} \geq P)$ **versus population** P **in log-log scale.** Cities proposed by the City Local Clustering Algorithm (CLCA) are represented by light blue circles. The solid black line is the maximum likelihood power-law fit defined by the Maximum Likelihood Estimator (MLE) [50]. The value of the lower bound P_{min} and the exponent ζ are also shown. The CLCA parameters used were $D^{(min)} = 100$ people$/km^2$, $D^{(max)} = 1000$ people$/km^2$, $\delta = 10$ people$/km^2$, $\ell = 3$ km, $A^* = 50$ km^2 and $H^* = 0.50$.

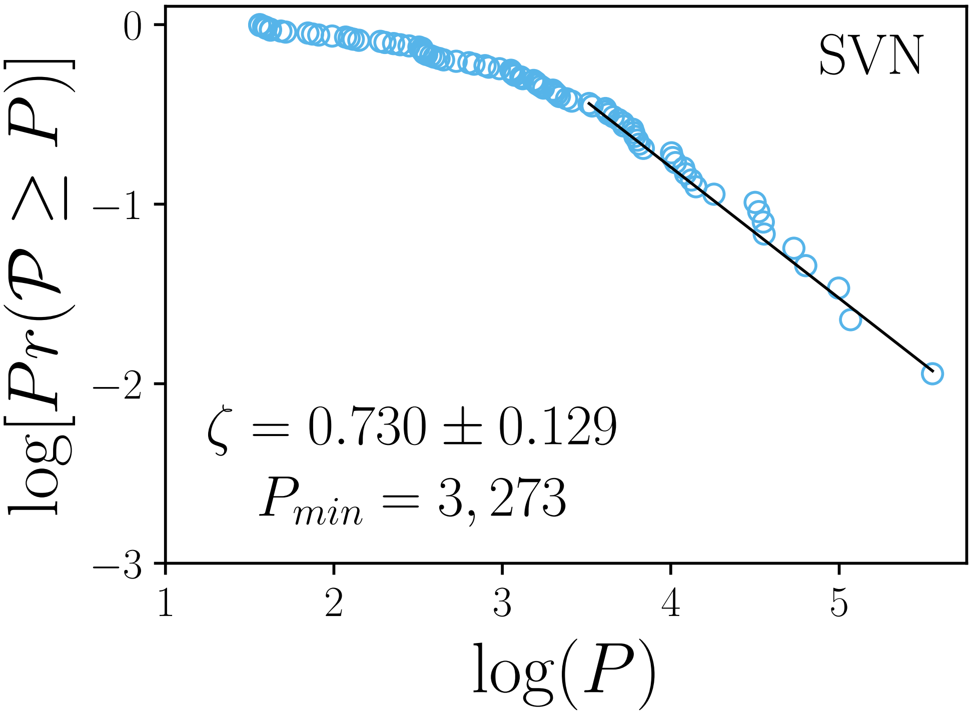

FIG. 129. **(Color online) Cumulative Distribution Function (CDF)** $Pr(\mathcal{P} \geq P)$ **versus population** P **in log-log scale.** Cities proposed by the City Local Clustering Algorithm (CLCA) are represented by light blue circles. The solid black line is the maximum likelihood power-law fit defined by the Maximum Likelihood Estimator (MLE) [50]. The value of the lower bound P_{min} and the exponent ζ are also shown. The CLCA parameters used were $D^{(min)} = 100$ people/km^2, $D^{(max)} = 1000$ people/km^2, $\delta = 10$ people/km^2, $\ell = 3$ km, $A^* = 50$ km^2 and $H^* = 0.50$.

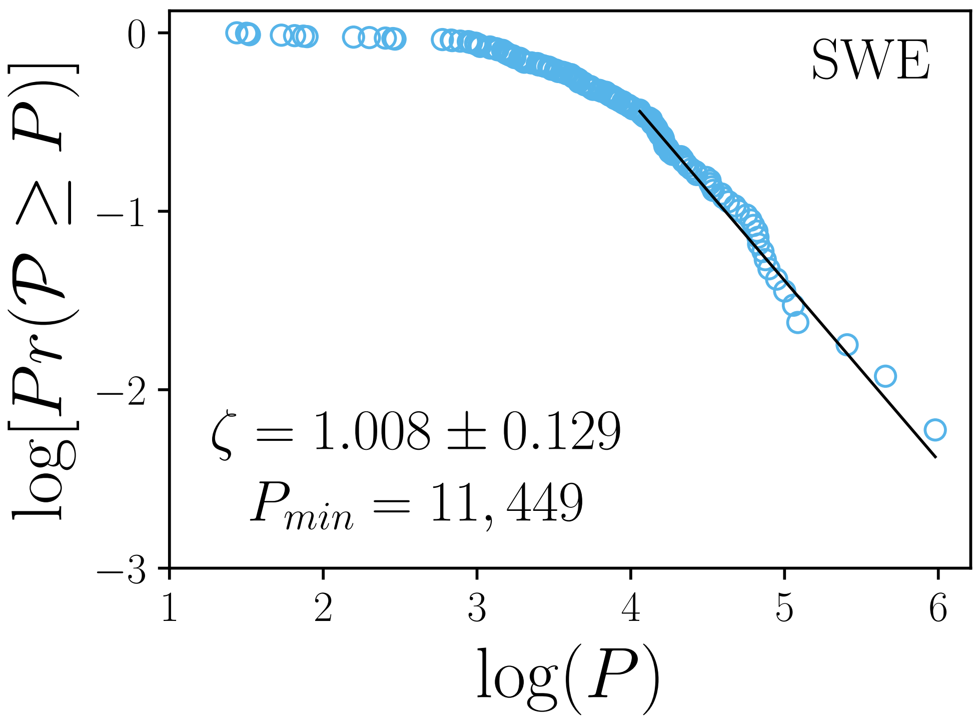

FIG. 130. **(Color online) Cumulative Distribution Function (CDF)** $Pr(\mathcal{P} \geq P)$ **versus population** P **in log-log scale.** Cities proposed by the City Local Clustering Algorithm (CLCA) are represented by light blue circles. The solid black line is the maximum likelihood power-law fit defined by the Maximum Likelihood Estimator (MLE) [50]. The value of the lower bound P_{min} and the exponent ζ are also shown. The CLCA parameters used were $D^{(min)} = 100$ people/km^2, $D^{(max)} = 1000$ people/km^2, $\delta = 10$ people/km^2, $\ell = 3$ km, $A^* = 50$ km^2 and $H^* = 0.50$.

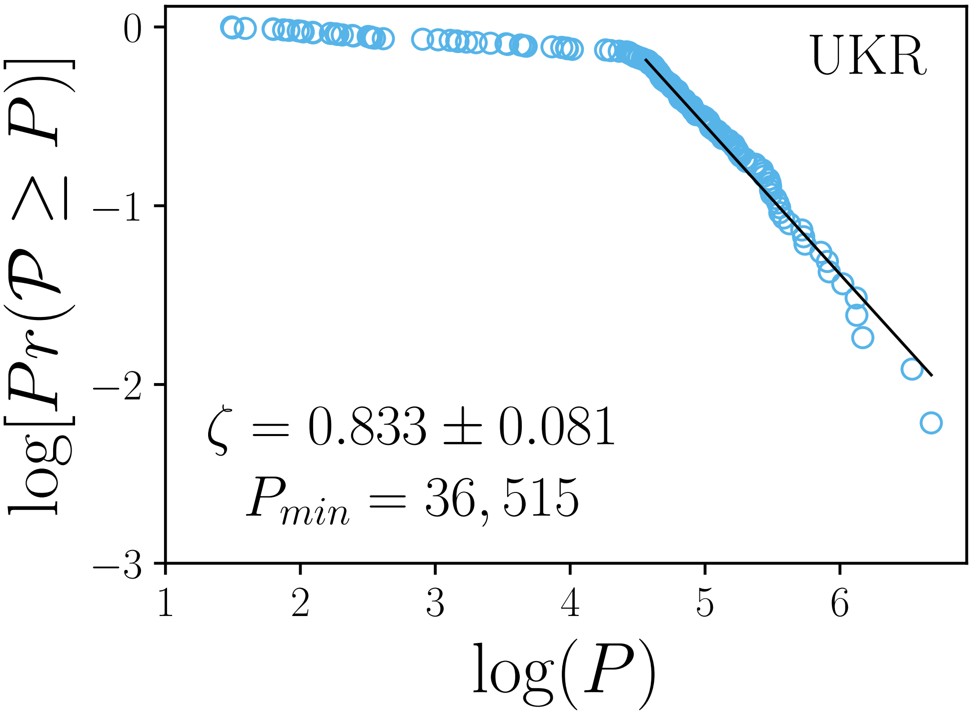

FIG. 131. **(Color online) Cumulative Distribution Function (CDF)** $Pr(\mathcal{P} \geq P)$ **versus population** P **in log-log scale.** Cities proposed by the City Local Clustering Algorithm (CLCA) are represented by light blue circles. The solid black line is the maximum likelihood power-law fit defined by the Maximum Likelihood Estimator (MLE) [50]. The value of the lower bound P_{min} and the exponent ζ are also shown. The CLCA parameters used were $D^{(min)} = 100$ people$/km^2$, $D^{(max)} = 1000$ people$/km^2$, $\delta = 10$ people$/km^2$, $\ell = 3\ km$, $A^* = 50\ km^2$ and $H^* = 0.50$.

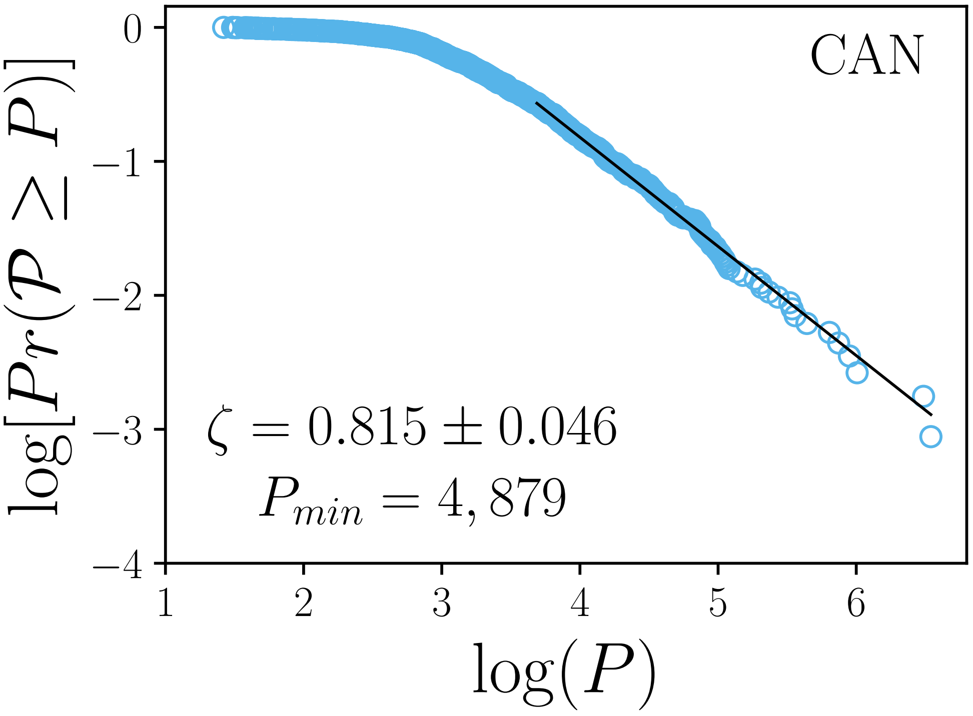

FIG. 132. **(Color online) Cumulative Distribution Function (CDF)** $Pr(\mathcal{P} \geq P)$ **versus population** P **in log-log scale.** Cities proposed by the City Local Clustering Algorithm (CLCA) are represented by light blue circles. The solid black line is the maximum likelihood power-law fit defined by the Maximum Likelihood Estimator (MLE) [50]. The value of the lower bound P_{min} and the exponent ζ are also shown. The CLCA parameters used were $D^{(min)} = 100$ people/km^2, $D^{(max)} = 1000$ people/km^2, $\delta = 10$ people/km^2, $\ell = 3$ km, $A^* = 50$ km^2 and $H^* = 0.50$.

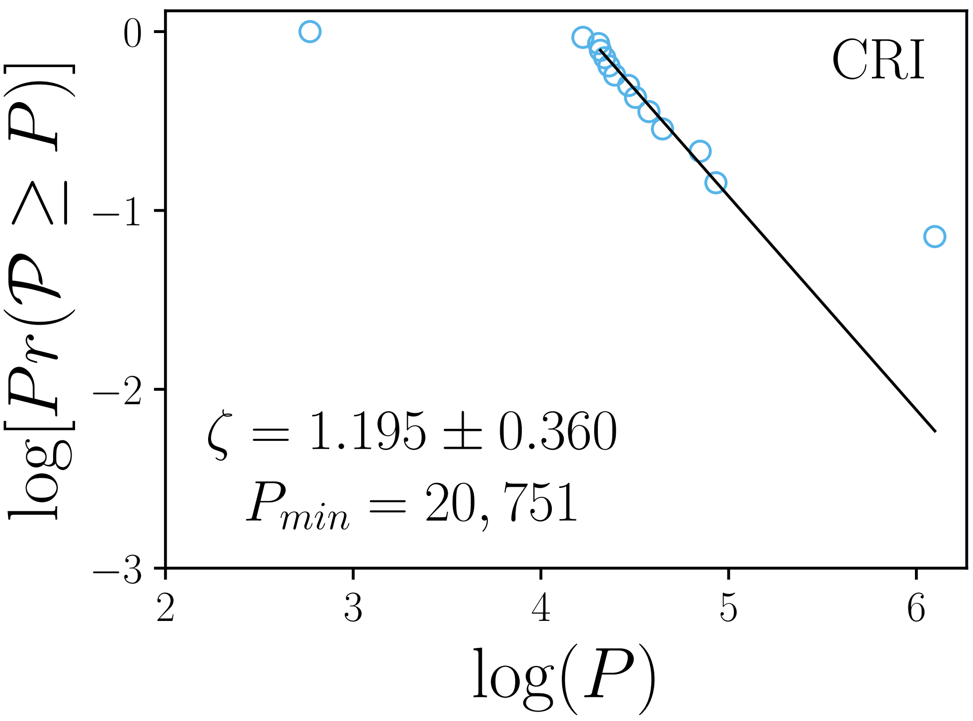

FIG. 133. **(Color online) Cumulative Distribution Function (CDF)** $Pr(\mathcal{P} \geq P)$ **versus population** P **in log-log scale.** Cities proposed by the City Local Clustering Algorithm (CLCA) are represented by light blue circles. The solid black line is the maximum likelihood power-law fit defined by the Maximum Likelihood Estimator (MLE) [50]. The value of the lower bound P_{min} and the exponent ζ are also shown. The CLCA parameters used were $D^{(min)} = 100$ people/km^2, $D^{(max)} = 1000$ people/km^2, $\delta = 10$ people/km^2, $\ell = 3$ km, $A^* = 50$ km^2 and $H^* = 0.50$.

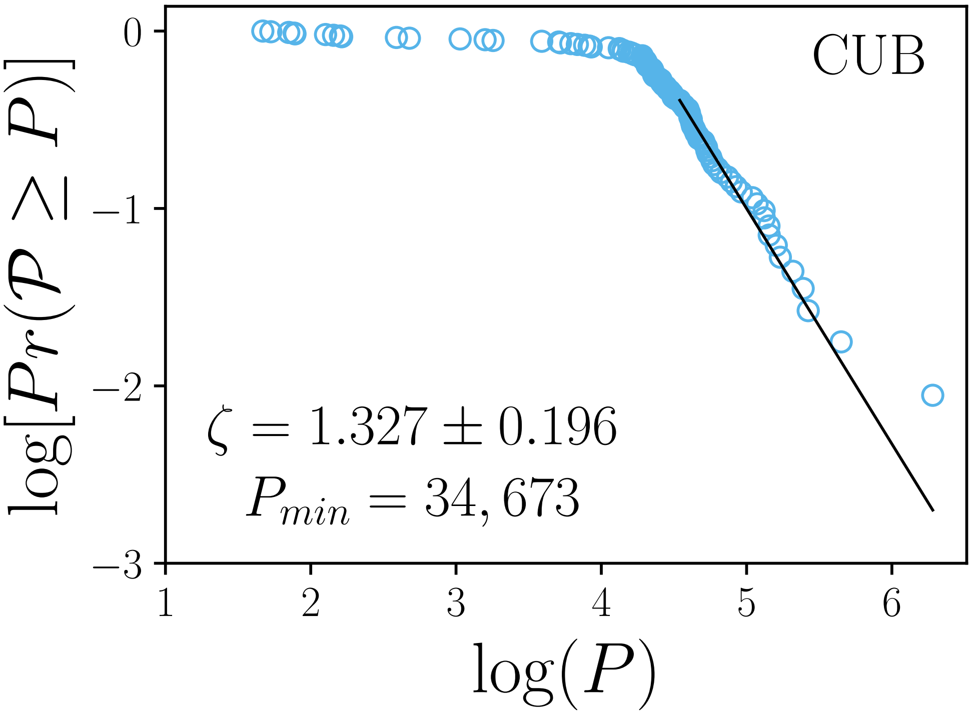

FIG. 134. **(Color online) Cumulative Distribution Function (CDF)** $Pr(\mathcal{P} \geq P)$ **versus population** P **in log-log scale.** Cities proposed by the City Local Clustering Algorithm (CLCA) are represented by light blue circles. The solid black line is the maximum likelihood power-law fit defined by the Maximum Likelihood Estimator (MLE) [50]. The value of the lower bound P_{min} and the exponent ζ are also shown. The CLCA parameters used were $D^{(min)} = 100$ people/km^2, $D^{(max)} = 1000$ people/km^2, $\delta = 10$ people/km^2, $\ell = 3$ km, $A^* = 50$ km^2 and $H^* = 0.50$.

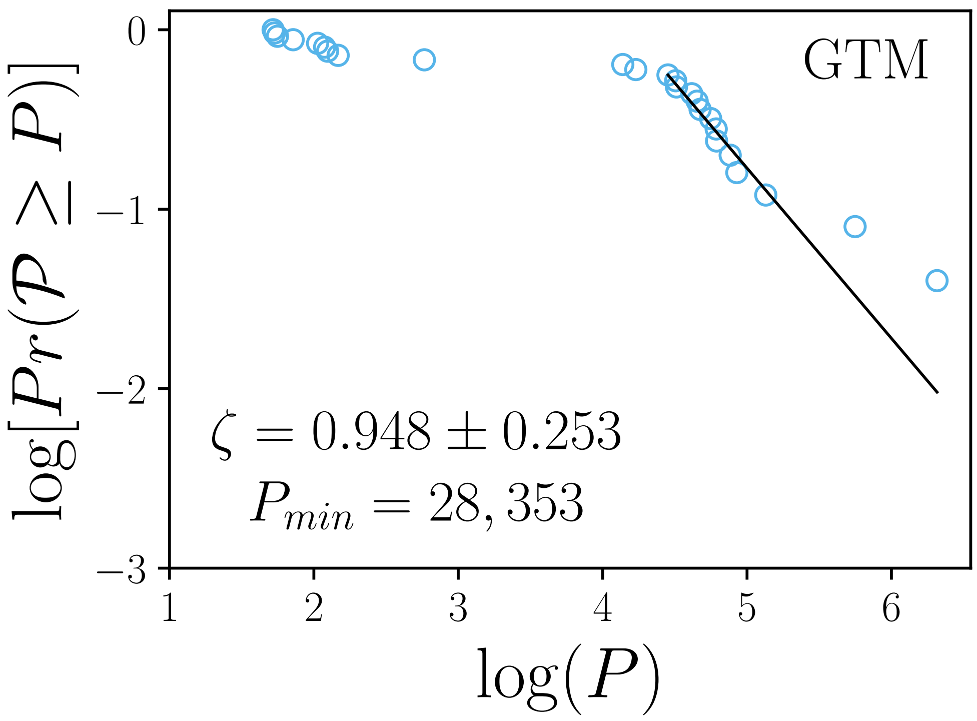

FIG. 135. **(Color online) Cumulative Distribution Function (CDF)** $Pr(\mathcal{P} \geq P)$ **versus population** P **in log-log scale.** Cities proposed by the City Local Clustering Algorithm (CLCA) are represented by light blue circles. The solid black line is the maximum likelihood power-law fit defined by the Maximum Likelihood Estimator (MLE) [50]. The value of the lower bound P_{min} and the exponent ζ are also shown. The CLCA parameters used were $D^{(min)} = 100$ people$/km^2$, $D^{(max)} = 1000$ people$/km^2$, $\delta = 10$ people$/km^2$, $\ell = 3$ km, $A^* = 50$ km^2 and $H^* = 0.50$.

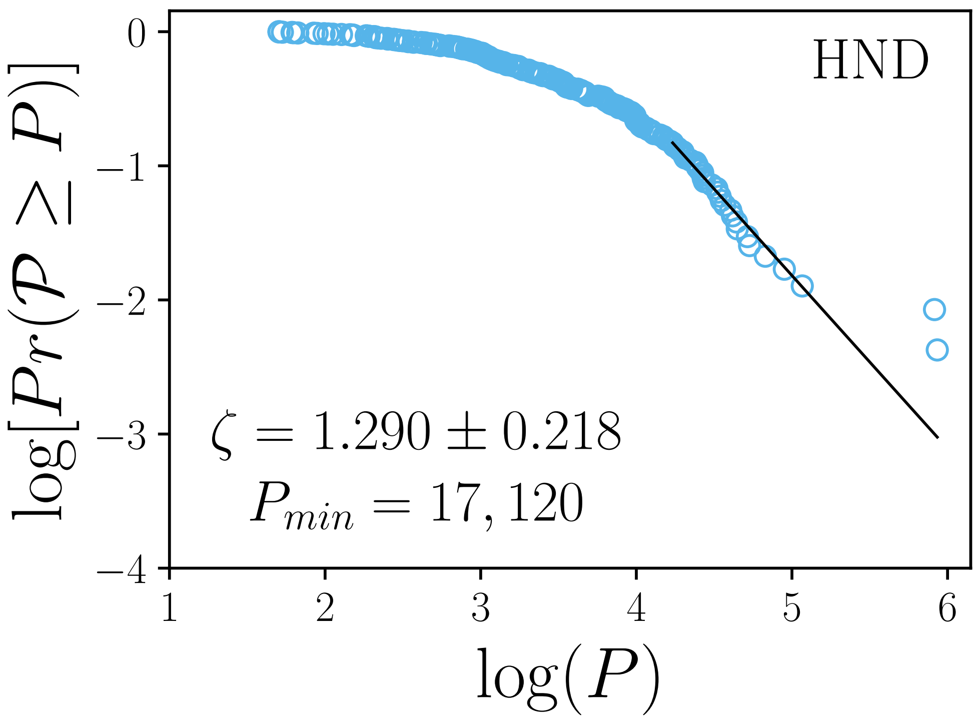

FIG. 136. **(Color online) Cumulative Distribution Function (CDF)** $Pr(\mathcal{P} \geq P)$ **versus population** P **in log-log scale.** Cities proposed by the City Local Clustering Algorithm (CLCA) are represented by light blue circles. The solid black line is the maximum likelihood power-law fit defined by the Maximum Likelihood Estimator (MLE) [50]. The value of the lower bound P_{min} and the exponent ζ are also shown. The CLCA parameters used were $D^{(min)} = 100$ people/km^2, $D^{(max)} = 1000$ people/km^2, $\delta = 10$ people/km^2, $\ell = 3$ km, $A^* = 50$ km^2 and $H^* = 0.50$.

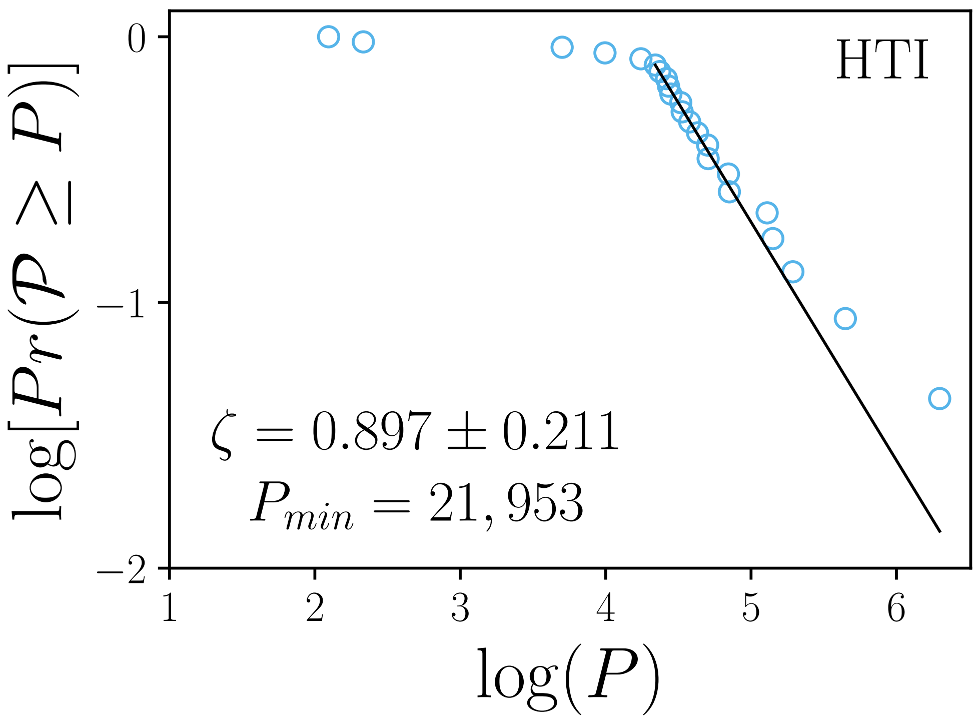

FIG. 137. **(Color online) Cumulative Distribution Function (CDF)** $Pr(\mathcal{P} \geq P)$ **versus population** P **in log-log scale.** Cities proposed by the City Local Clustering Algorithm (CLCA) are represented by light blue circles. The solid black line is the maximum likelihood power-law fit defined by the Maximum Likelihood Estimator (MLE) [50]. The value of the lower bound P_{min} and the exponent ζ are also shown. The CLCA parameters used were $D^{(min)} = 100$ people/km^2, $D^{(max)} = 1000$ people/km^2, $\delta = 10$ people/km^2, $\ell = 3$ km, $A^* = 50$ km^2 and $H^* = 0.50$.

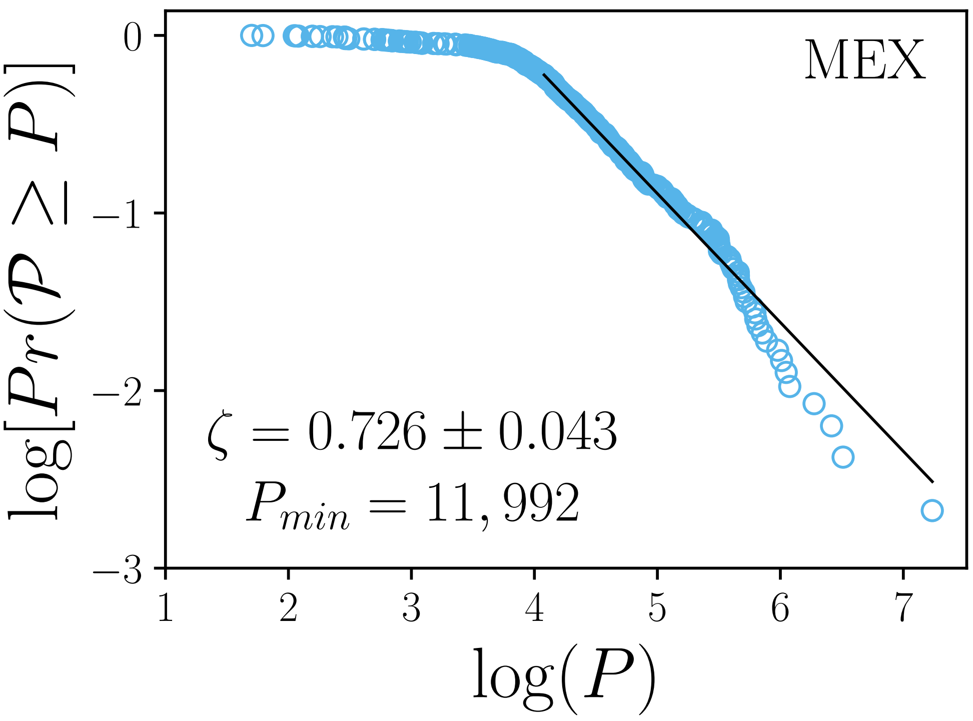

FIG. 138. **(Color online) Cumulative Distribution Function (CDF)** $Pr(\mathcal{P} \geq P)$ **versus population** P **in log-log scale.** Cities proposed by the City Local Clustering Algorithm (CLCA) are represented by light blue circles. The solid black line is the maximum likelihood power-law fit defined by the Maximum Likelihood Estimator (MLE) [50]. The value of the lower bound P_{min} and the exponent ζ are also shown. The CLCA parameters used were $D^{(min)} = 100$ people$/km^2$, $D^{(max)} = 1000$ people$/km^2$, $\delta = 10$ people$/km^2$, $\ell = 3$ km, $A^* = 50$ km^2 and $H^* = 0.50$.

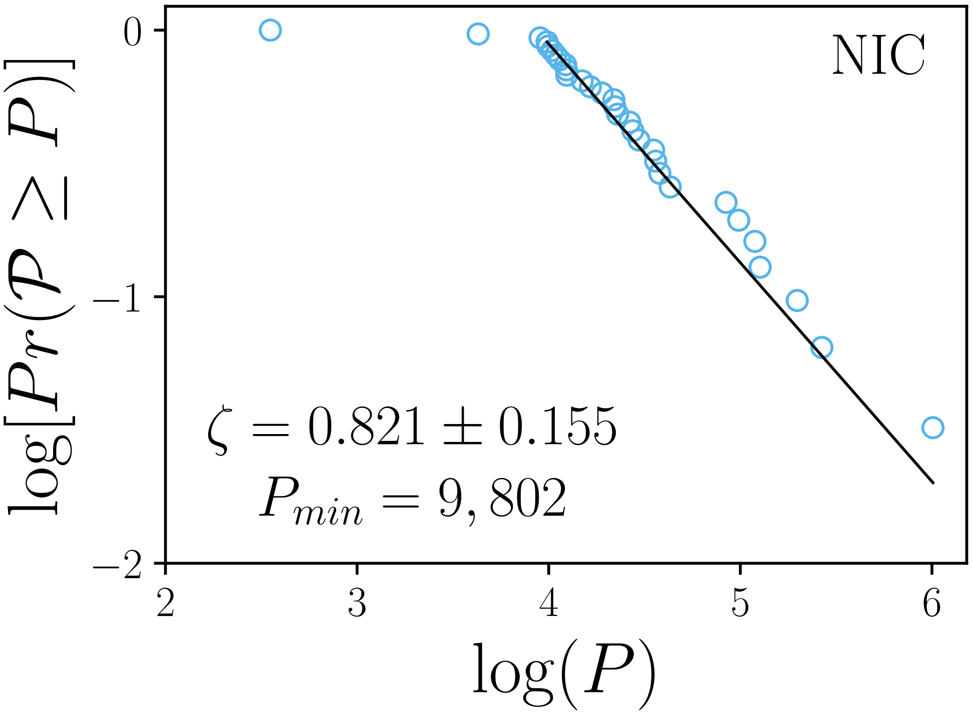

FIG. 139. **(Color online) Cumulative Distribution Function (CDF)** $Pr(\mathcal{P} \geq P)$ **versus population** P **in log-log scale.** Cities proposed by the City Local Clustering Algorithm (CLCA) are represented by light blue circles. The solid black line is the maximum likelihood power-law fit defined by the Maximum Likelihood Estimator (MLE) [50]. The value of the lower bound P_{min} and the exponent ζ are also shown. The CLCA parameters used were $D^{(min)} = 100$ people/km^2, $D^{(max)} = 1000$ people/km^2, $\delta = 10$ people/km^2, $\ell = 3$ km, $A^* = 50$ km^2 and $H^* = 0.50$.

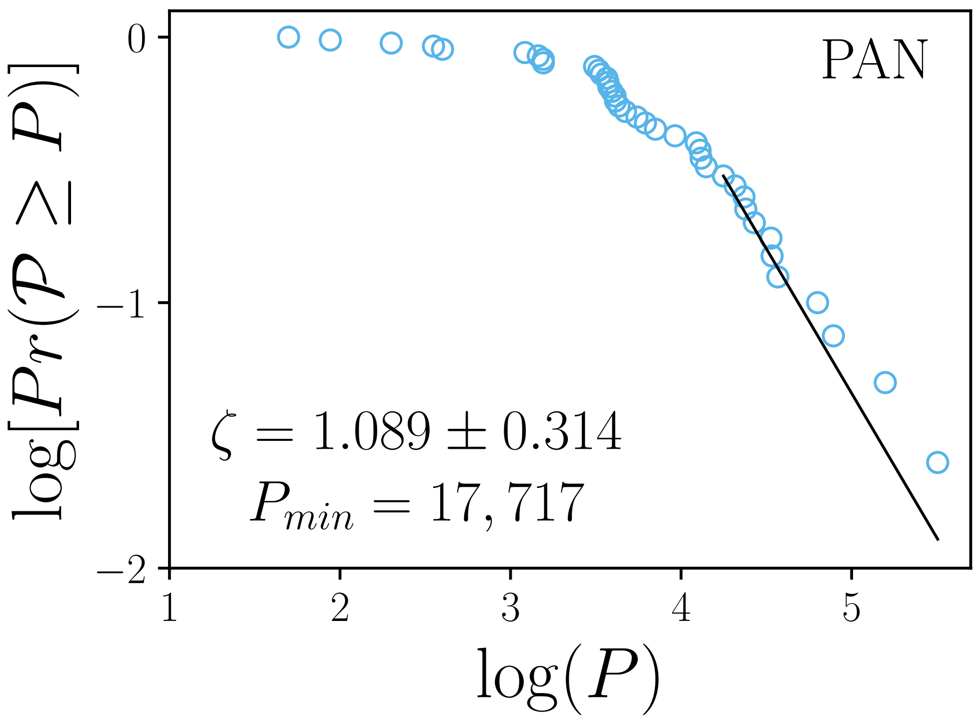

FIG. 140. **(Color online) Cumulative Distribution Function (CDF)** $Pr(\mathcal{P} \geq P)$ **versus population** P **in log-log scale.** Cities proposed by the City Local Clustering Algorithm (CLCA) are represented by light blue circles. The solid black line is the maximum likelihood power-law fit defined by the Maximum Likelihood Estimator (MLE) [50]. The value of the lower bound P_{min} and the exponent ζ are also shown. The CLCA parameters used were $D^{(min)} = 100$ people/km^2, $D^{(max)} = 1000$ people/km^2, $\delta = 10$ people/km^2, $\ell = 3$ km, $A^* = 50$ km^2 and $H^* = 0.50$.

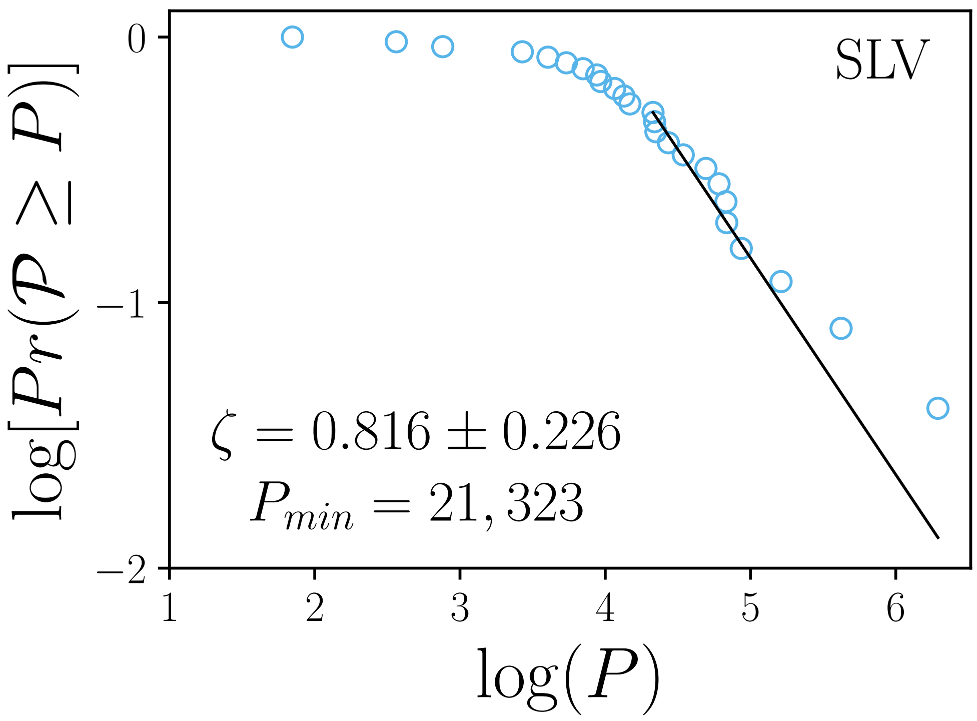

FIG. 141. **(Color online) Cumulative Distribution Function (CDF)** $Pr(\mathcal{P} \geq P)$ **versus population** P **in log-log scale.** Cities proposed by the City Local Clustering Algorithm (CLCA) are represented by light blue circles. The solid black line is the maximum likelihood power-law fit defined by the Maximum Likelihood Estimator (MLE) [50]. The value of the lower bound P_{min} and the exponent ζ are also shown. The CLCA parameters used were $D^{(min)} = 100$ people/km^2, $D^{(max)} = 1000$ people/km^2, $\delta = 10$ people/km^2, $\ell = 3\ km$, $A^* = 50\ km^2$ and $H^* = 0.50$.

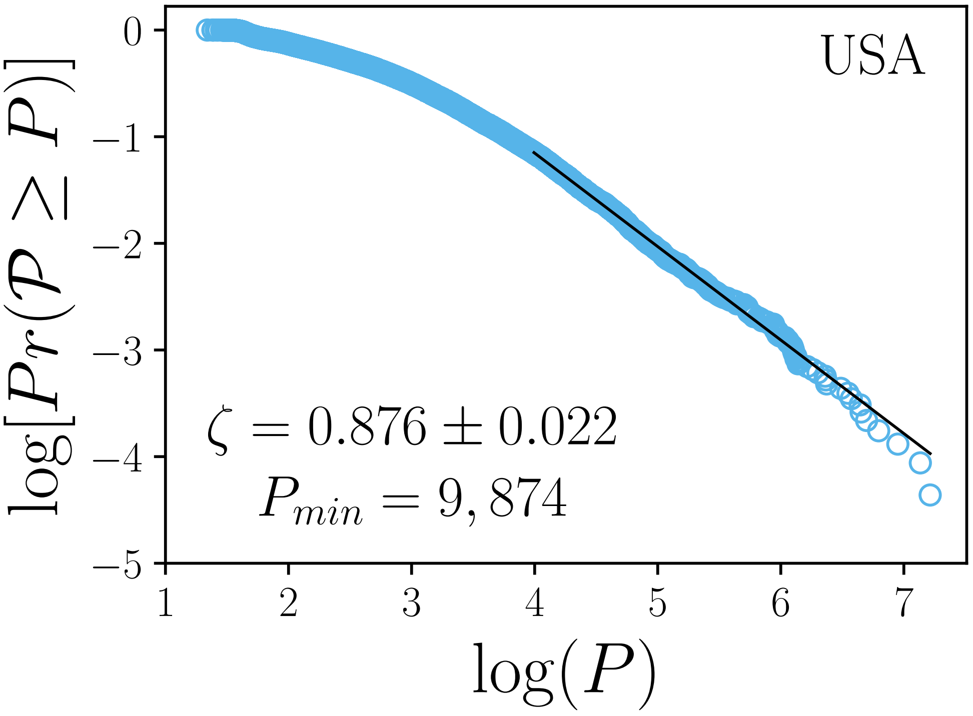

FIG. 142. **(Color online) Cumulative Distribution Function (CDF)** $Pr(\mathcal{P} \geq P)$ **versus population** P **in log-log scale.** Cities proposed by the City Local Clustering Algorithm (CLCA) are represented by light blue circles. The solid black line is the maximum likelihood power-law fit defined by the Maximum Likelihood Estimator (MLE) [50]. The value of the lower bound P_{min} and the exponent ζ are also shown. The CLCA parameters used were $D^{(min)} = 100$ people/km^2, $D^{(max)} = 1000$ people/km^2, $\delta = 10$ people/km^2, $\ell = 3$ km, $A^* = 50$ km^2 and $H^* = 0.50$.

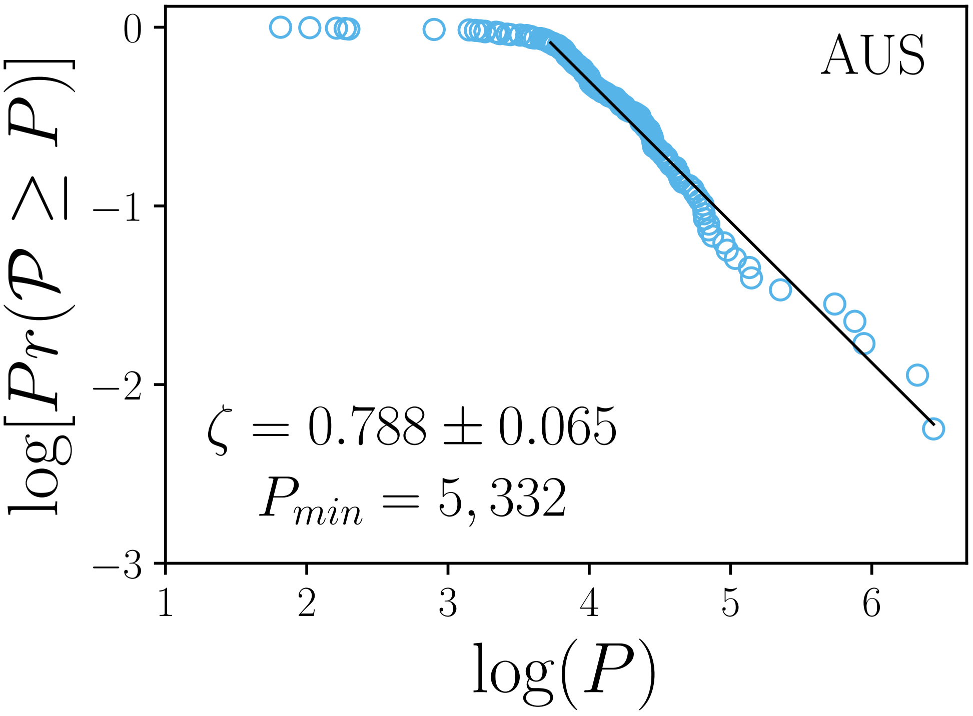

FIG. 143. **(Color online) Cumulative Distribution Function (CDF)** $Pr(\mathcal{P} \geq P)$ **versus population** P **in log-log scale.** Cities proposed by the City Local Clustering Algorithm (CLCA) are represented by light blue circles. The solid black line is the maximum likelihood power-law fit defined by the Maximum Likelihood Estimator (MLE) [50]. The value of the lower bound P_{min} and the exponent ζ are also shown. The CLCA parameters used were $D^{(min)} = 100$ people/km^2, $D^{(max)} = 1000$ people/km^2, $\delta = 10$ people/km^2, $\ell = 3$ km, $A^* = 50$ km^2 and $H^* = 0.50$.

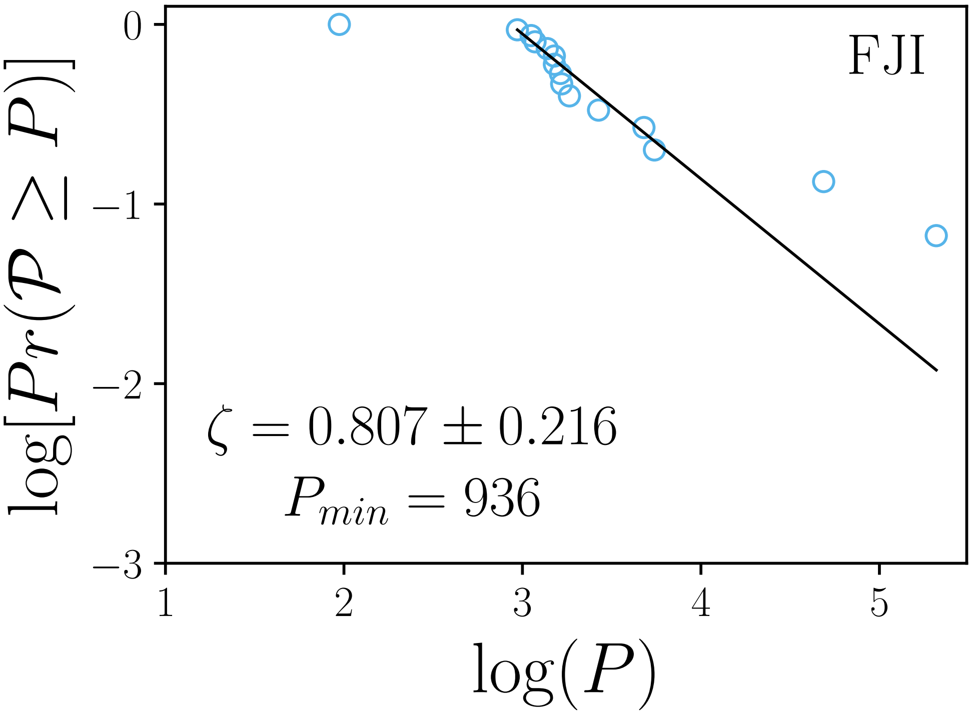

FIG. 144. **(Color online) Cumulative Distribution Function (CDF)** $Pr(\mathcal{P} \geq P)$ **versus population** P **in log-log scale.** Cities proposed by the City Local Clustering Algorithm (CLCA) are represented by light blue circles. The solid black line is the maximum likelihood power-law fit defined by the Maximum Likelihood Estimator (MLE) [50]. The value of the lower bound P_{min} and the exponent ζ are also shown. The CLCA parameters used were $D^{(min)} = 100$ people/km^2, $D^{(max)} = 1000$ people/km^2, $\delta = 10$ people/km^2, $\ell = 3\ km$, $A^* = 50\ km^2$ and $H^* = 0.50$.

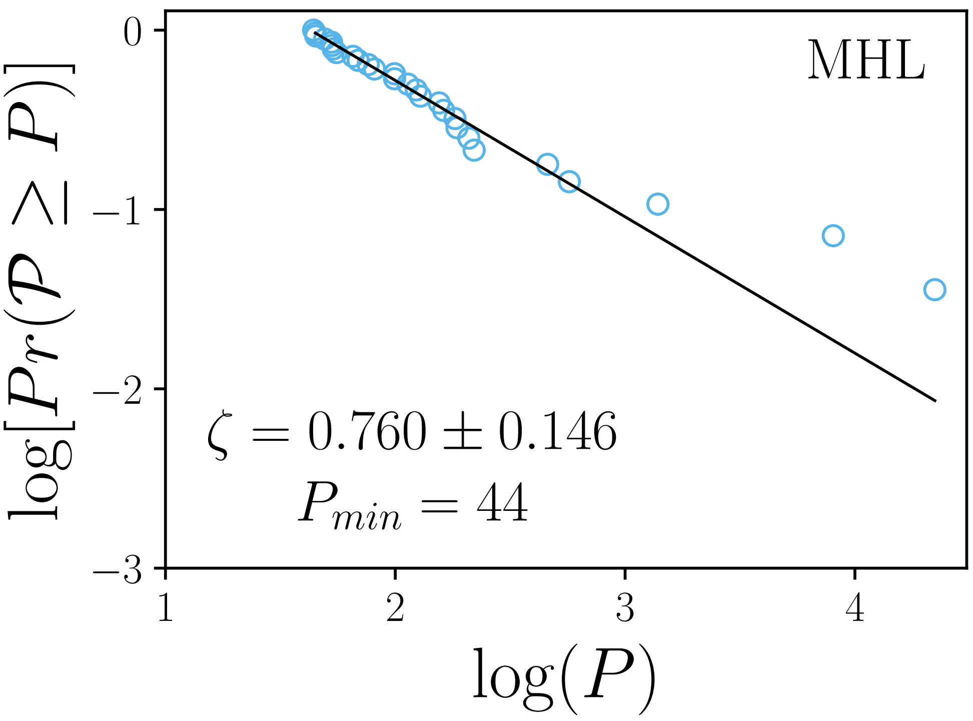

FIG. 145. **(Color online) Cumulative Distribution Function (CDF)** $Pr(\mathcal{P} \geq P)$ **versus population** P **in log-log scale.** Cities proposed by the City Local Clustering Algorithm (CLCA) are represented by light blue circles. The solid black line is the maximum likelihood power-law fit defined by the Maximum Likelihood Estimator (MLE) [50]. The value of the lower bound P_{min} and the exponent ζ are also shown. The CLCA parameters used were $D^{(min)} = 100$ people/km^2, $D^{(max)} = 1000$ people/km^2, $\delta = 10$ people/km^2, $\ell = 3$ km, $A^* = 50$ km^2 and $H^* = 0.50$.

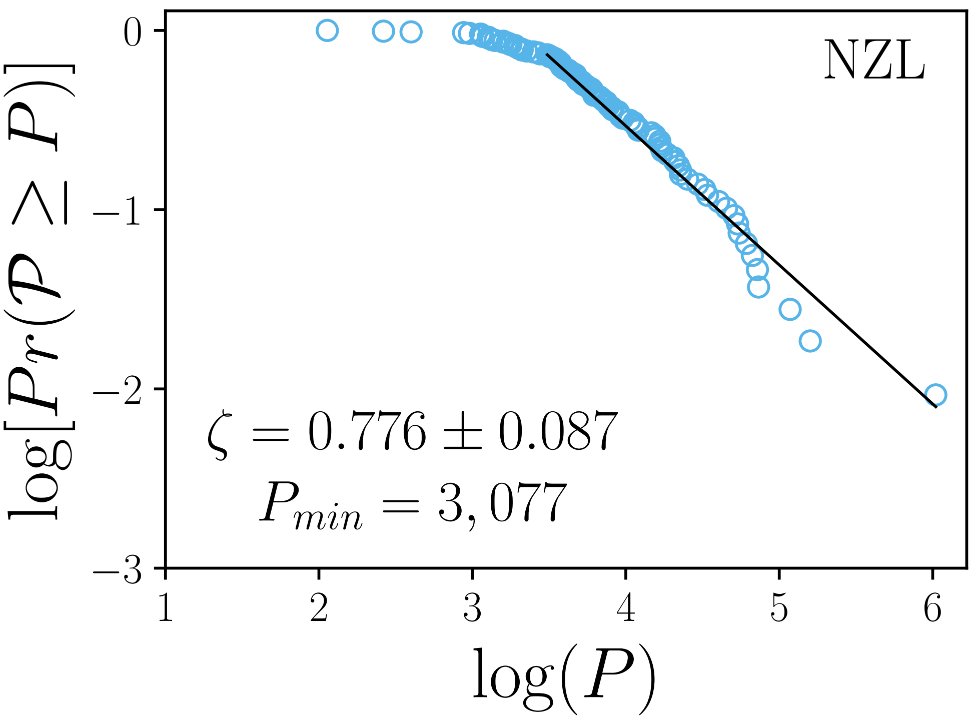

FIG. 146. **(Color online) Cumulative Distribution Function (CDF)** $Pr(\mathcal{P} \geq P)$ **versus population** P **in log-log scale.** Cities proposed by the City Local Clustering Algorithm (CLCA) are represented by light blue circles. The solid black line is the maximum likelihood power-law fit defined by the Maximum Likelihood Estimator (MLE) [50]. The value of the lower bound P_{min} and the exponent ζ are also shown. The CLCA parameters used were $D^{(min)} = 100$ people/km^2, $D^{(max)} = 1000$ people/km^2, $\delta = 10$ people/km^2, $\ell = 3$ km, $A^* = 50$ km^2 and $H^* = 0.50$.

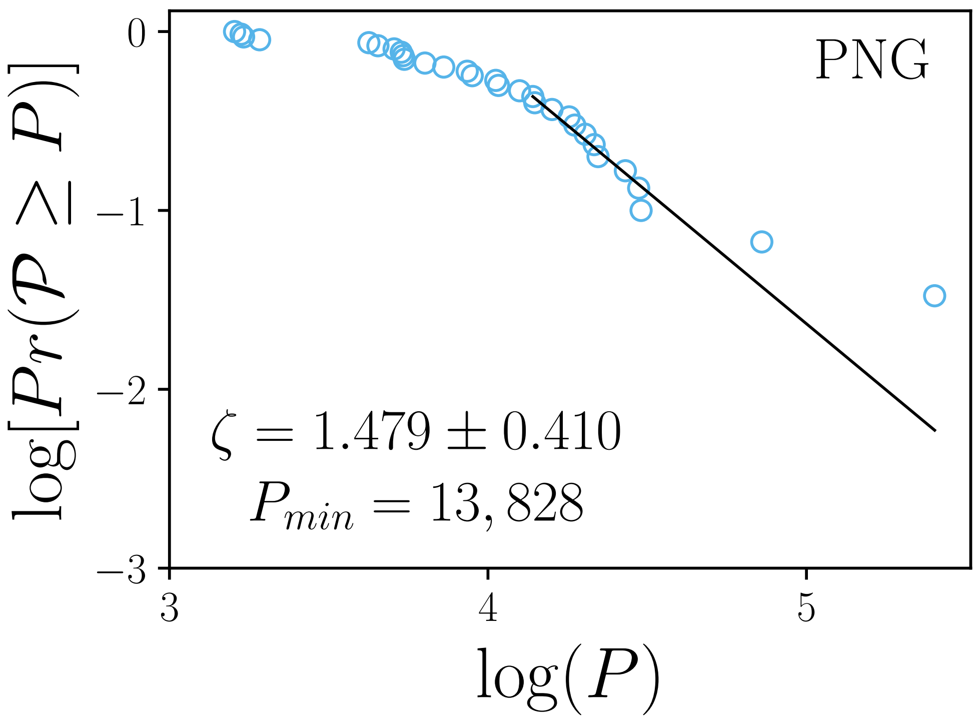

FIG. 147. **(Color online) Cumulative Distribution Function (CDF)** $Pr(\mathcal{P} \geq P)$ **versus population** P **in log-log scale.** Cities proposed by the City Local Clustering Algorithm (CLCA) are represented by light blue circles. The solid black line is the maximum likelihood power-law fit defined by the Maximum Likelihood Estimator (MLE) [50]. The value of the lower bound P_{min} and the exponent ζ are also shown. The CLCA parameters used were $D^{(min)} = 100$ people$/km^2$, $D^{(max)} = 1000$ people$/km^2$, $\delta = 10$ people$/km^2$, $\ell = 3\ km$, $A^* = 50\ km^2$ and $H^* = 0.50$.

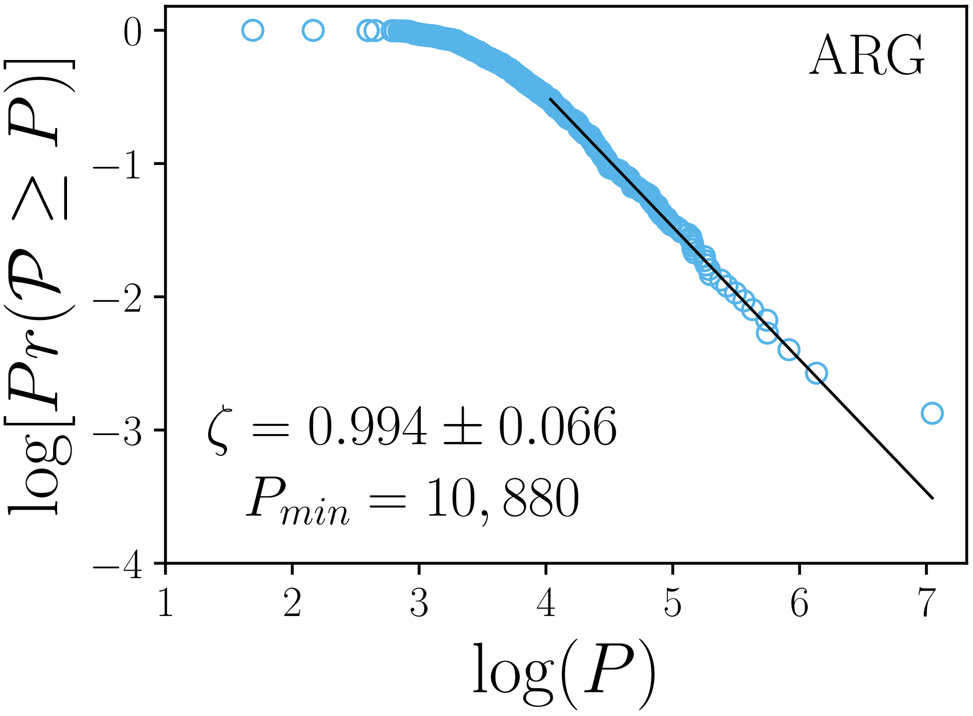

FIG. 148. **(Color online) Cumulative Distribution Function (CDF)** $Pr(\mathcal{P} \geq P)$ **versus population** P **in log-log scale.** Cities proposed by the City Local Clustering Algorithm (CLCA) are represented by light blue circles. The solid black line is the maximum likelihood power-law fit defined by the Maximum Likelihood Estimator (MLE) [50]. The value of the lower bound P_{min} and the exponent ζ are also shown. The CLCA parameters used were $D^{(min)} = 100$ people/km^2, $D^{(max)} = 1000$ people/km^2, $\delta = 10$ people/km^2, $\ell = 3$ km, $A^* = 50$ km^2 and $H^* = 0.50$.

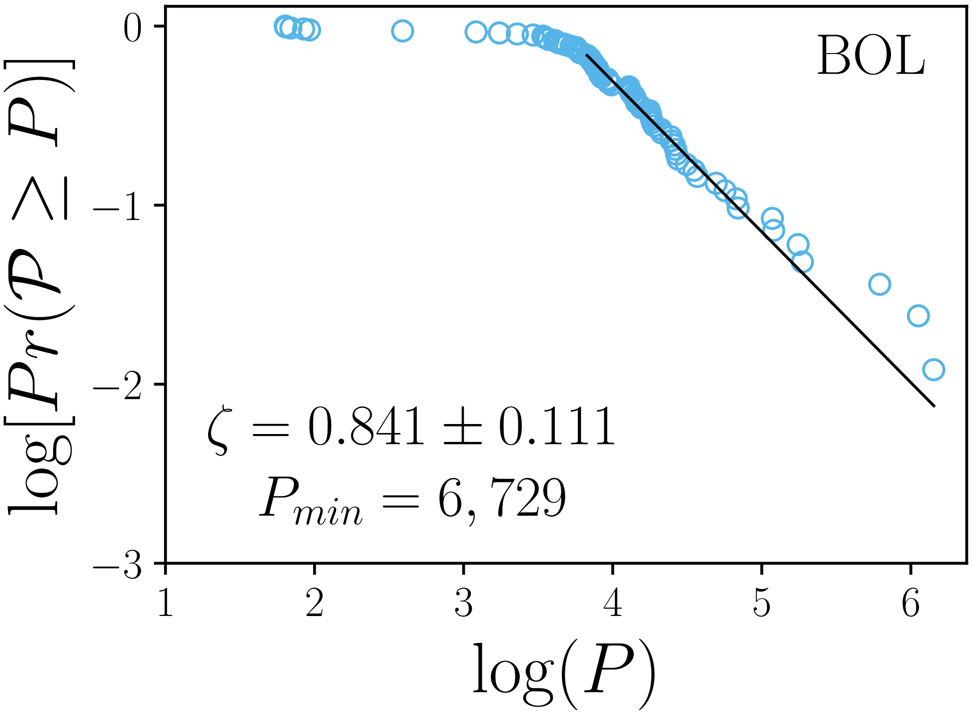

FIG. 149. **(Color online) Cumulative Distribution Function (CDF)** $Pr(\mathcal{P} \geq P)$ **versus population** P **in log-log scale.** Cities proposed by the City Local Clustering Algorithm (CLCA) are represented by light blue circles. The solid black line is the maximum likelihood power-law fit defined by the Maximum Likelihood Estimator (MLE) [50]. The value of the lower bound P_{min} and the exponent ζ are also shown. The CLCA parameters used were $D^{(min)} = 100$ people/km^2, $D^{(max)} = 1000$ people/km^2, $\delta = 10$ people/km^2, $\ell = 3$ km, $A^* = 50$ km^2 and $H^* = 0.50$.

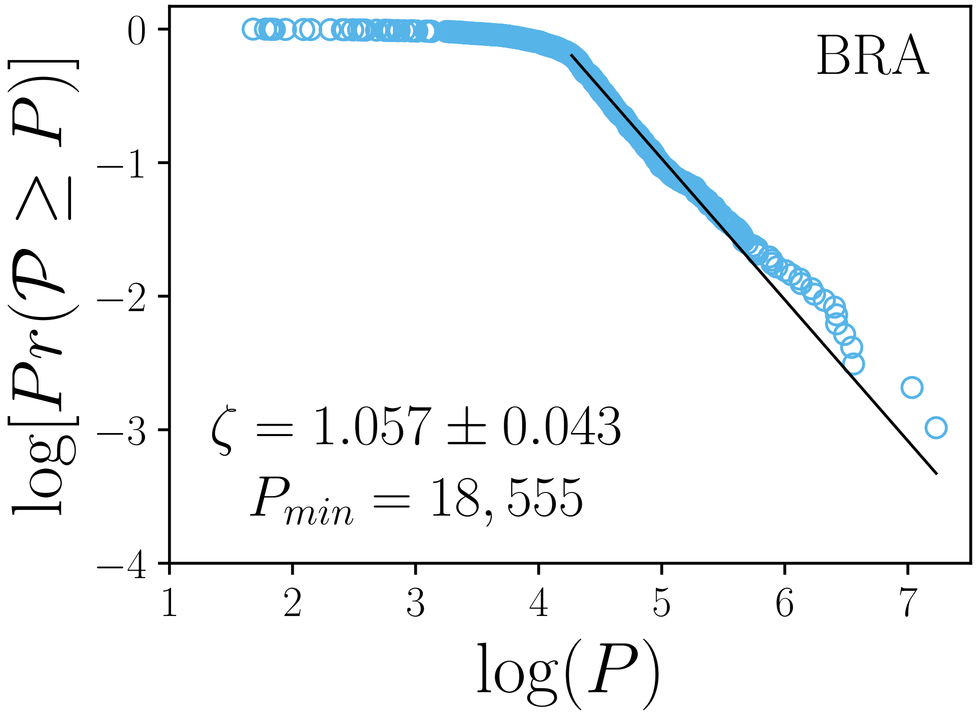

FIG. 150. **(Color online) Cumulative Distribution Function (CDF)** $Pr(\mathcal{P} \geq P)$ **versus population** P **in log-log scale.** Cities proposed by the City Local Clustering Algorithm (CLCA) are represented by light blue circles. The solid black line is the maximum likelihood power-law fit defined by the Maximum Likelihood Estimator (MLE) [50]. The value of the lower bound P_{min} and the exponent ζ are also shown. The CLCA parameters used were $D^{(min)} = 100$ people$/km^2$, $D^{(max)} = 1000$ people$/km^2$, $\delta = 10$ people$/km^2$, $\ell = 3\ km$, $A^* = 50\ km^2$ and $H^* = 0.50$.

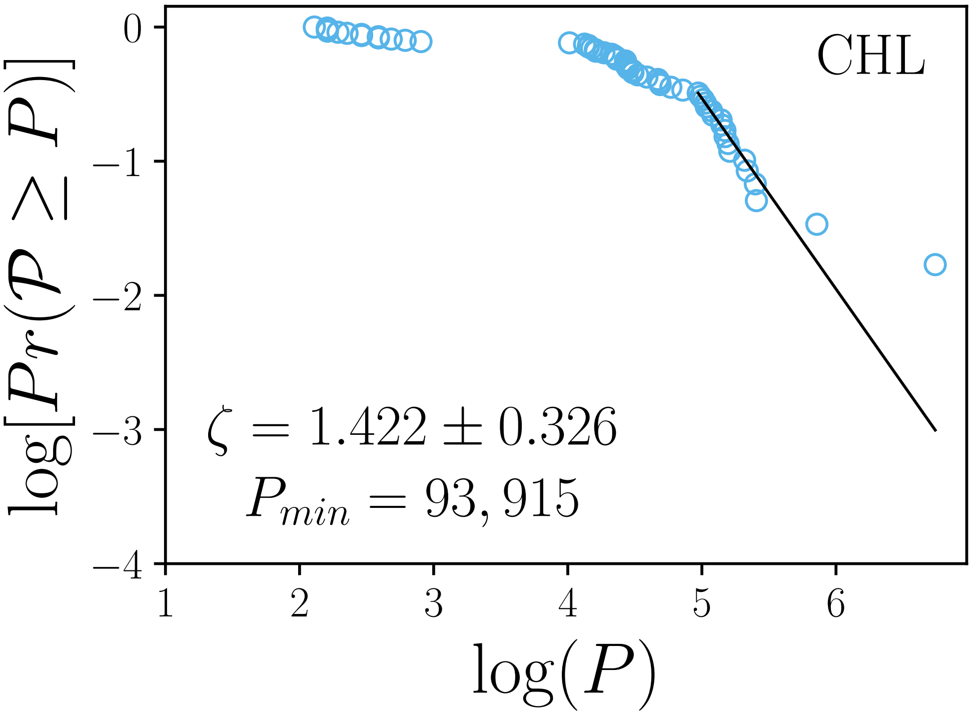

FIG. 151. **(Color online) Cumulative Distribution Function (CDF)** $Pr(\mathcal{P} \geq P)$ **versus population** P **in log-log scale.** Cities proposed by the City Local Clustering Algorithm (CLCA) are represented by light blue circles. The solid black line is the maximum likelihood power-law fit defined by the Maximum Likelihood Estimator (MLE) [50]. The value of the lower bound P_{min} and the exponent ζ are also shown. The CLCA parameters used were $D^{(min)} = 100$ people$/km^2$, $D^{(max)} = 1000$ people$/km^2$, $\delta = 10$ people$/km^2$, $\ell = 3$ km, $A^* = 50$ km^2 and $H^* = 0.50$.

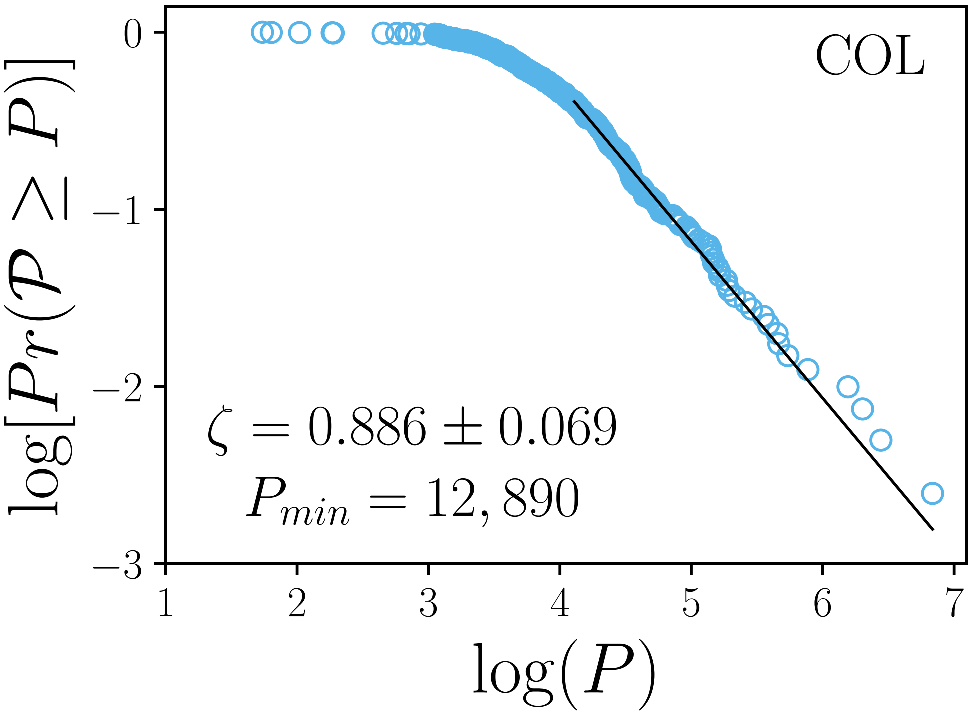

FIG. 152. **(Color online) Cumulative Distribution Function (CDF)** $Pr(\mathcal{P} \geq P)$ **versus population** P **in log-log scale.** Cities proposed by the City Local Clustering Algorithm (CLCA) are represented by light blue circles. The solid black line is the maximum likelihood power-law fit defined by the Maximum Likelihood Estimator (MLE) [50]. The value of the lower bound P_{min} and the exponent ζ are also shown. The CLCA parameters used were $D^{(min)} = 100$ people/km^2, $D^{(max)} = 1000$ people/km^2, $\delta = 10$ people/km^2, $\ell = 3$ km, $A^* = 50$ km^2 and $H^* = 0.50$.

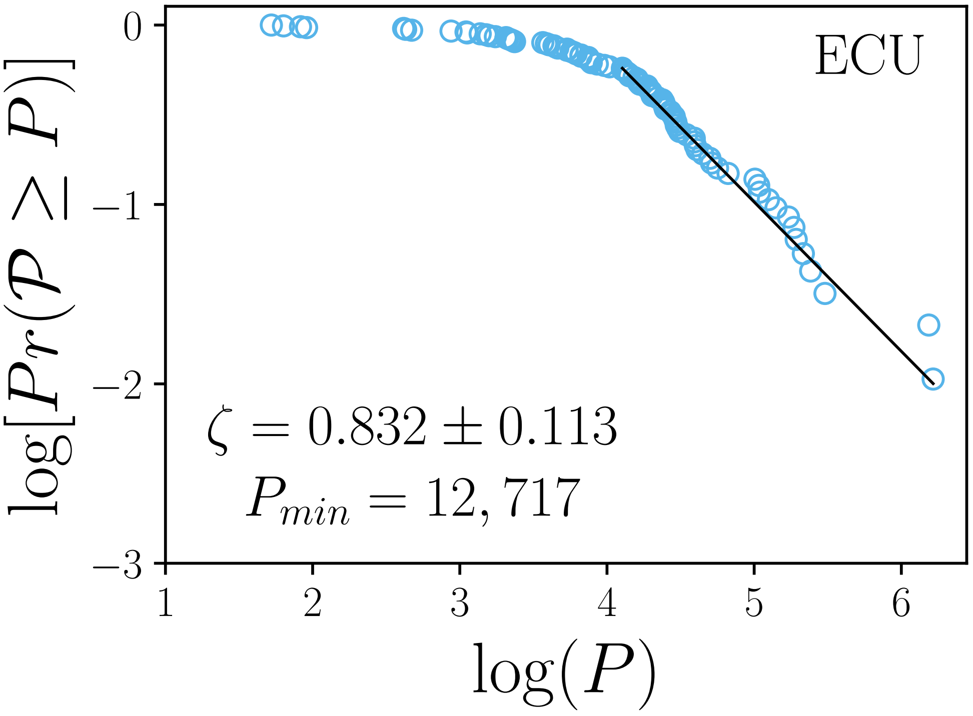

FIG. 153. **(Color online) Cumulative Distribution Function (CDF)** $Pr(\mathcal{P} \geq P)$ **versus population** P **in log-log scale.** Cities proposed by the City Local Clustering Algorithm (CLCA) are represented by light blue circles. The solid black line is the maximum likelihood power-law fit defined by the Maximum Likelihood Estimator (MLE) [50]. The value of the lower bound P_{min} and the exponent ζ are also shown. The CLCA parameters used were $D^{(min)} = 100$ people$/km^2$, $D^{(max)} = 1000$ people$/km^2$, $\delta = 10$ people$/km^2$, $\ell = 3$ km, $A^* = 50$ km^2 and $H^* = 0.50$.

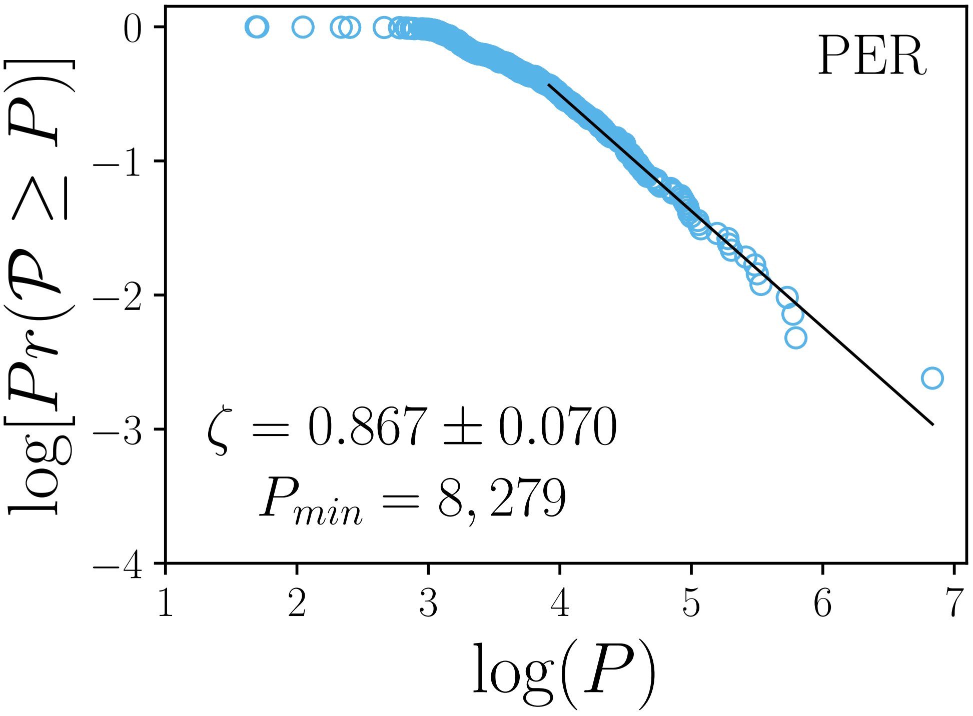

FIG. 154. **(Color online) Cumulative Distribution Function (CDF)** $Pr(\mathcal{P} \geq P)$ **versus population** P **in log-log scale.** Cities proposed by the City Local Clustering Algorithm (CLCA) are represented by light blue circles. The solid black line is the maximum likelihood power-law fit defined by the Maximum Likelihood Estimator (MLE) [50]. The value of the lower bound P_{min} and the exponent ζ are also shown. The CLCA parameters used were $D^{(min)} = 100$ people/km^2, $D^{(max)} = 1000$ people/km^2, $\delta = 10$ people/km^2, $\ell = 3\ km$, $A^* = 50\ km^2$ and $H^* = 0.50$.

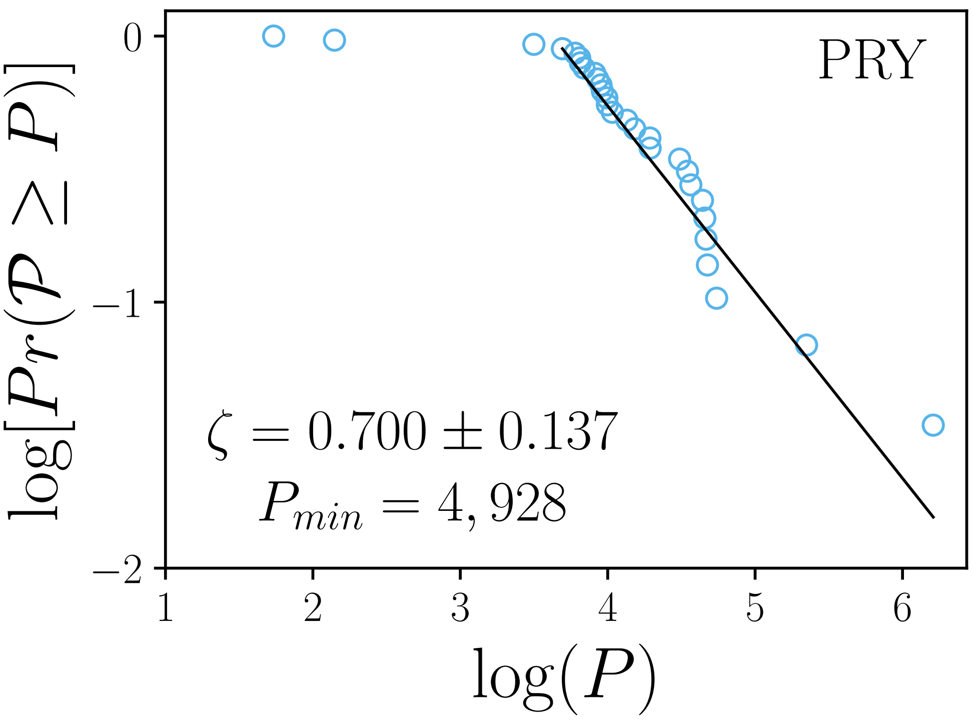

FIG. 155. **(Color online) Cumulative Distribution Function (CDF)** $Pr(\mathcal{P} \geq P)$ **versus population** P **in log-log scale.** Cities proposed by the City Local Clustering Algorithm (CLCA) are represented by light blue circles. The solid black line is the maximum likelihood power-law fit defined by the Maximum Likelihood Estimator (MLE) [50]. The value of the lower bound P_{min} and the exponent ζ are also shown. The CLCA parameters used were $D^{(min)} = 100$ people/km^2, $D^{(max)} = 1000$ people/km^2, $\delta = 10$ people/km^2, $\ell = 3$ km, $A^* = 50$ km^2 and $H^* = 0.50$.

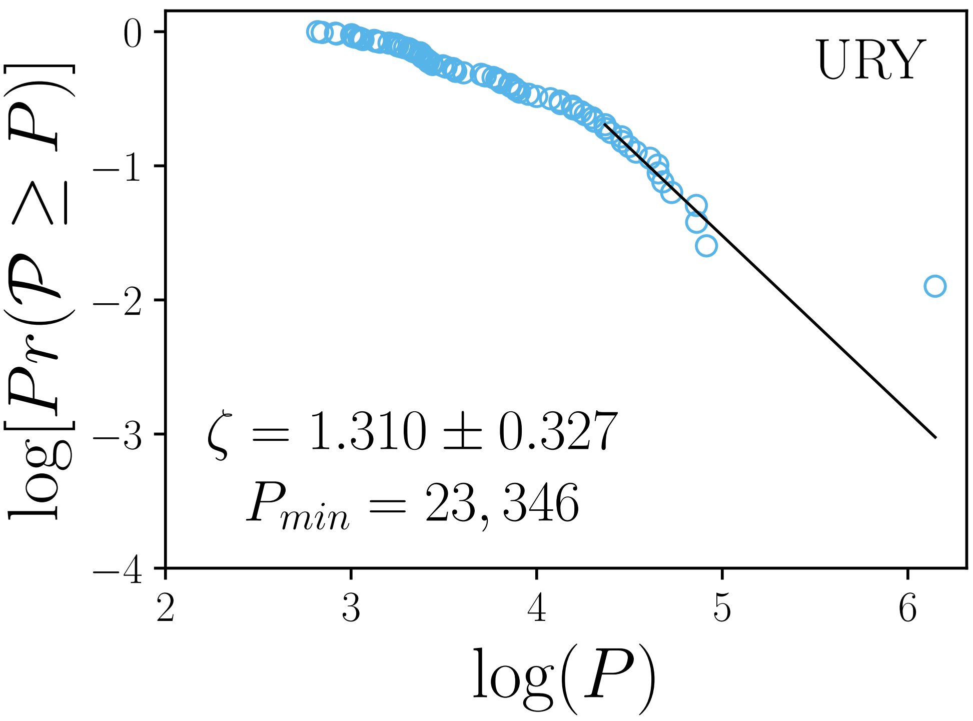

FIG. 156. **(Color online) Cumulative Distribution Function (CDF)** $Pr(\mathcal{P} \geq P)$ **versus population** P **in log-log scale.** Cities proposed by the City Local Clustering Algorithm (CLCA) are represented by light blue circles. The solid black line is the maximum likelihood power-law fit defined by the Maximum Likelihood Estimator (MLE) [50]. The value of the lower bound P_{min} and the exponent ζ are also shown. The CLCA parameters used were $D^{(min)} = 100$ people/km^2, $D^{(max)} = 1000$ people/km^2, $\delta = 10$ people/km^2, $\ell = 3$ km, $A^* = 50$ km^2 and $H^* = 0.50$.

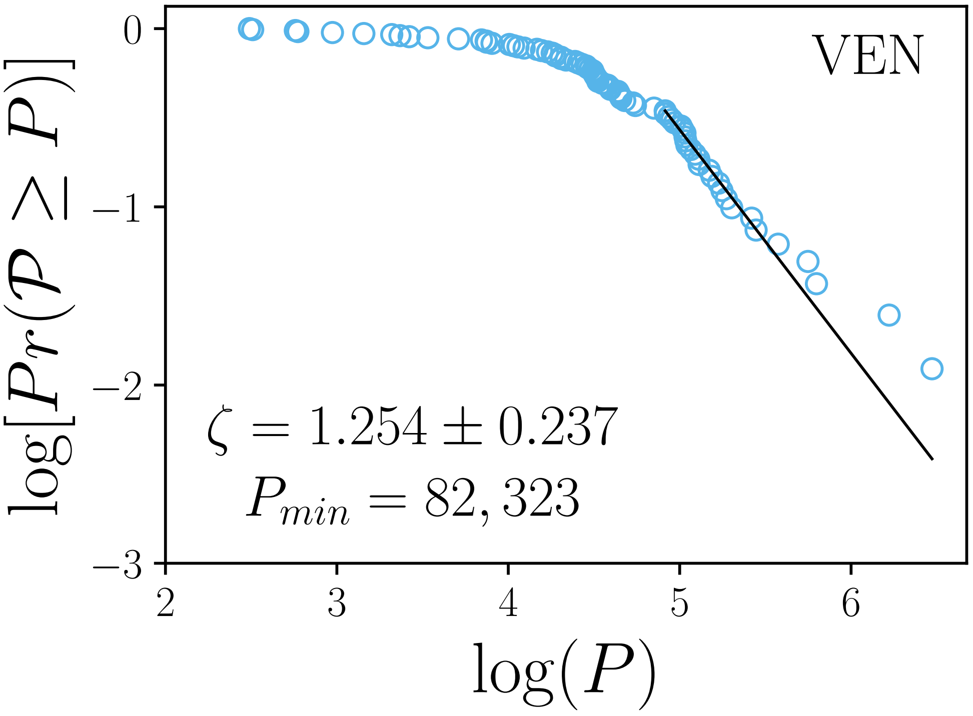

FIG. 157. **(Color online) Cumulative Distribution Function (CDF)** $Pr(\mathcal{P} \geq P)$ **versus population** P **in log-log scale.** Cities proposed by the City Local Clustering Algorithm (CLCA) are represented by light blue circles. The solid black line is the maximum likelihood power-law fit defined by the Maximum Likelihood Estimator (MLE) [50]. The value of the lower bound P_{min} and the exponent ζ are also shown. The CLCA parameters used were $D^{(min)} = 100$ people/km^2, $D^{(max)} = 1000$ people/km^2, $\delta = 10$ people/km^2, $\ell = 3$ km, $A^* = 50$ km^2 and $H^* = 0.50$.
